# Integrating Genome-Scale Metabolic Models with Patient Plasma Metabolome to Study Endothelial Metabolism In Situ

**DOI:** 10.3390/ijms25105406

**Published:** 2024-05-15

**Authors:** Fernando Silva-Lance, Isabel Montejano-Montelongo, Eric Bautista, Lars K. Nielsen, Pär I. Johansson, Igor Marin de Mas

**Affiliations:** 1 Novo Nordisk Foundation Center for Biosustainability, Danish Technical University, 2800 Lyngby, Denmark; 2 CAG Center for Endotheliomics, Copenhagen University Hospital, Rigshospitalet, 2100 Copenhagen, Denmark; 3 Department of Clinical Medicine, University of Copenhagen, 2200 Copenhagen, Denmark

**Keywords:** metabolic network analysis, genome-scale metabolic models, exo-metabolomics integration, sampling algorithms, endothelial cell metabolism

## Abstract

Patient blood samples are invaluable in clinical omics databases, yet current methodologies often fail to fully uncover the molecular mechanisms driving patient pathology. While genome-scale metabolic models (GEMs) show promise in systems medicine by integrating various omics data, having only exometabolomic data remains a limiting factor. To address this gap, we introduce a comprehensive pipeline integrating GEMs with patient plasma metabolome. This pipeline constructs case-specific GEMs using literature-based and patient-specific metabolomic data. Novel computational methods, including adaptive sampling and an in-house developed algorithm for the rational exploration of the sampled space of solutions, enhance integration accuracy while improving computational performance. Model characterization involves task analysis in combination with clustering methods to identify critical cellular functions. The new pipeline was applied to a cohort of trauma patients to investigate shock-induced endotheliopathy using patient plasma metabolome data. By analyzing endothelial cell metabolism comprehensively, the pipeline identified critical therapeutic targets and biomarkers that can potentially contribute to the development of therapeutic strategies. Our study demonstrates the efficacy of integrating patient plasma metabolome data into computational models to analyze endothelial cell metabolism in disease contexts. This approach offers a deeper understanding of metabolic dysregulations and provides insights into diseases with metabolic components and potential treatments.

## 1. Introduction

Genome-scale metabolic models (GEMs) have been widely used in systems medicine to unravel the behavior of metabolic pathways and their contributions to the development and progression of diseases [1,2]. These models provide a comprehensive representation of an organism’s metabolic network, capturing the intricate interconnectedness of biochemical reactions and the flow of metabolites [3]. This systems biology tool plays a crucial role in the study of diseases with a strong metabolic component, such as diabetes and cancer, where dysregulated metabolic pathways are key contributors to pathogenesis [4]. GEMs serve as a robust platform for integrating multiple omics data, including genomics, proteomics, and metabolic-related data, which allows researchers to gain a holistic view of the molecular changes and identify key metabolic alterations associated with a specific disease or patient [5].

Metabolic-related data, including metabolomics data, measurements of metabolite consumption/secretion rates, or time-course data, can be integrated into GEMs facilitating the identification of metabolic biomarkers, prediction of metabolic fluxes, and exploration of potential therapeutic targets [6]. Furthermore, GEMs can simulate the effect of genetic or environmental perturbations on the metabolic network, providing a framework to investigate the underlying mechanisms of disease development and progression [7].

However, studying the metabolism of endothelial cells (EC), which play a crucial role in many diseases, presents unique obstacles that hinder the effective utilization of these methodologies. Obtaining viable samples directly from the microvascular endothelium is challenging due to the tissue’s complexity, the fragility of EC, the need to preserve the endothelial phenotype, and the lack of suitable techniques [8].

Consequently, researchers often rely on in vitro cell culture models, such as Human Lung Microvascular ECs (HMVEC-L), to gain insights into endothelial biology and disease mechanisms. However, culturing HMVEC-L cells in vitro results in alterations compared to their in vivo counterparts due to the absence of physiological cues, including specific oxygen tensions, shear stress, and cell–cell interactions [9]. The two-dimensional monolayer culture fails to replicate the essential three-dimensional architecture observed in vivo, impacting cell behavior, gene expression, and endothelial barrier function. In vitro cultures’ nutrient and oxygen supply may not precisely match the in vivo environment, leading to metabolic differences and altered cellular responses. Moreover, the absence of immune cells and other cellular interactions in vitro hinders understanding of crucial physiological processes involving ECs, such as immune responses, inflammation, and coagulation [10,11].

Since ECs are the most abundant cell type in the vascular compartment and have a direct impact on plasma metabolome, analyzing plasma metabolome can help in the study of EC dysregulation and alterations in the context of pathogenesis [12].

To address the need for integrating patient plasma metabolome into computational models describing endothelial metabolism, we have developed a computational pipeline for the construction of case/patient-specific GEM of the endothelium by using patient-specific plasma metabolome data with a generic GEM of the endothelium.

The first step in our pipeline involves reconstructing case-specific EC GEMs. We constrain the boundaries of the generic reconstruction of EC metabolism using literature-based data. This step ensures that the model reflects the specific metabolic capabilities described for ECs. Subsequently, we determine the baseline model through flux sampling, allowing us to explore the feasible metabolic states of the cell [13]. Next, we define the minimum and maximum uptake/secretion rates of exchange reactions based on a Rational Exploration of Space Of Solutions (RESOS) of their respective flux distributions [14]. By re-scaling the baseline GEM thresholds using case-specific relative metabolomics in combination with flux sampling [13], we construct case/patient-specific GEMs. These case-specific models provide a more accurate representation of the metabolic capabilities of the ECs under specific conditions [15].

Next, we investigate the cellular capabilities of the case-specific EC GEMs. This involves applying a task analysis of a set of manually curated metabolic tasks [16], which allows us to assess the cell’s ability to perform specific metabolic functions and identify any aberrations or limitations in the case-specific models [17].

To facilitate the interpretation of the results and identify patterns within the data, we employ clustering analysis in combination with heat map representation and multi-variate analysis [18]. This allows for the stratification of patients based on their metabolic profiles and helps uncover distinct subgroups or phenotypes within the cohort [18,19].

In our pipeline, we have integrated novel computational methods that enhance the accuracy of exometabolomics data integration. Moreover, our approach considers the variability observed in the control group, allowing for the construction of patient-specific GEMs. This inclusion of patient-specific information ensures a more realistic representation of the metabolic profiles and enhances the precision of our models.

To address computational challenges, we have implemented a novel sampling algorithm called Adaptive Directions Sampling on a Box (ADSB) [20]. This algorithm significantly reduces the computational time required for exploring the solution space, enabling faster analysis of the metabolic models.

Furthermore, we have developed and incorporated a novel algorithm for RESOS. This algorithm excels at pinpointing the regions within the feasible flux solution space that most accurately represent the metabolic network. Its effectiveness is evidenced by its systematic exploration of diverse parameter configurations, leading to the identification of optimal solutions and thereby enhancing the characterization of GEMs. This enhances our understanding of the metabolic network and provides valuable insights into the system behavior.

As a proof of concept, we apply this method to a cohort of 95 trauma patients, previously clustered into four groups (metabo-groups) based on their metabolic profiles in blood samples, which correlate with increasing mortality rates [17]. Trauma represents one of the leading causes of death globally, with significant morbidity and mortality rates [21]. Our previous research has linked poor prognosis in trauma patients to EC membrane damage caused by the over-activation of the sympathetic system, resulting in a condition known as shock-induced endotheliopathy (SHINE) [22]. SHINE leads to increased extravasation, tissue pressure, microvascular thrombus formation, impaired oxygen delivery, multiple organ failure (MOF), and ultimately, death [23].

By integrating patient plasma metabolome data into the EC-GEM, we aim to gain a deeper understanding of the metabolic alterations associated with SHINE and identify potential therapeutic interventions. Our comprehensive pipeline, encompassing case-specific GEM, task analysis, and results visualization, allows us to explore the intricate metabolic network of ECs in the context of the disease.

The presented pipeline offers a novel computational method to analyze patient plasma metabolome and investigate EC metabolism. By uncovering metabolic dysregulations and identifying critical substrates, this approach holds promise for improving our understanding of diseases with a metabolic component and developing targeted therapeutic strategies to mitigate their effects.

## 2. Results

### 2.1. Assessing RESOS Algorithm

The RESOS algorithm proposed in this study harnesses the non-uniform distribution of solutions across the feasible flux solution space, emphasizing that solutions clustered in denser regions offer richer insights into the metabolic system. Put simply, RESOS leverages Euclidean distances between solutions to pinpoint these densest regions, where shorter distances signify higher density, and thus, greater metabolic characterization. In the upcoming subsections, this algorithm will undergo evaluation at various levels. Initially, we will compare two methods for identifying the densest region: the continuous and discrete approaches. Subsequently, we will assess the effectiveness of the dynamic threshold method in determining the extent of the densest solution region. Finally, we will examine the enhanced predictive capabilities of the model when RESOS is applied.

#### 2.1.1. Assessing RESOS Algorithm: Continuous vs. Discrete Approach

The RESOS algorithm allows for a discrete or continuous approach (see Section 4, Methods). The discrete method is faster, since it depends on the number of reactions in the model rather than the number of valid solutions. However, the binning of values in histograms reduces the resolution and similar solutions may have identical Euclidean distance value.

**Figure 1:**
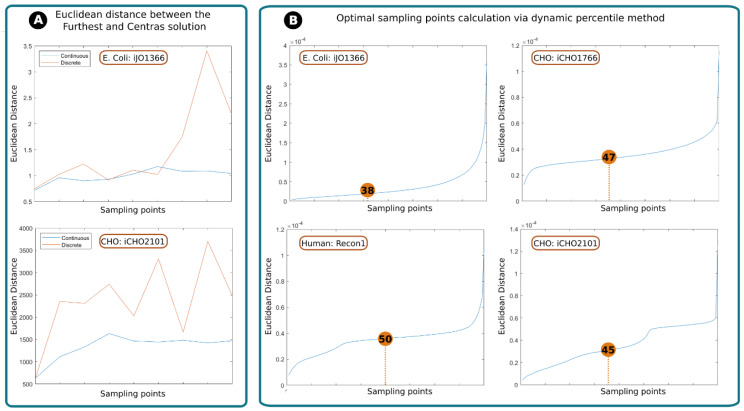
(**A**). Euclidean distance between the “furthest” and “central” solutions: evolution of the distance between the central and furthest solution as sampling points increase using *E. coli* iJO1366 and CHO iCHO2101 GEMs. Blue lines represent the continuous approach, while red line represents the discrete approach. (**B**). Optimal sampling point calculation via dynamic percentile method: computed inflexion points as the second derivative of the curve representing the number of sampling points and distance between the central and furthest solutions. This evolution is represented by a blue line and the inflexion point with an orange circle with a number that indicates the percentile. The assessed GEMs are (from up to down and from left to right): *E. coli* (iJO1366), CHO (iCHO1766), Human (Recon1), and CHO (iCHO2101).

These two approaches were compared using two models, *E. coli* [24] and CHO [25], to determine the most appropriate strategy to explore the space of feasible flux solutions of a Flux Balance Analysis (FBA). The models were sampled with fixed step lengths up to a total of 16,000 points. Next, the Euclidean distances [26] between the “furthest” and “central” solution were computed as a function of the number of points sampled (Figure 1A). In both cases, the discrete method shows inconsistency between the number of sampling points and the distance between the central and furthest solutions. On the other hand, the continuous method eventually reaches a stable state, meaning that, no matter how many points are sampled, the distance between the furthest and central solution will not tend to vary significantly after a determined number of points. This behavior can be attributed to the dynamic percentile, as it ensures that no outliers are considered.

In conclusion, despite the fact that the discrete method has shown to be faster, the continuous method is more accurate and reliable.

#### 2.1.2. Assessing RESOS Algorithm: Dynamic Threshold and Determining the Optimal Number of Sampling Points

The standard approach to mitigate the effect of extreme solutions is to establish a threshold that defines a limit between the sampled solutions that will be considered for the analysis and those that will be rejected. For example, one might reject the first and last quartiles of a population of solutions for a given reaction and only retain the central solutions, corresponding to the second and third quartiles [19]. In our case, one logical solution would be to establish a fixed percentile for every sampling, for example, eliminate those reactions whose total Euclidean distance value is above the 90th percentile. However, this does not guarantee that the solutions recovered are, indeed, those belonging to the densest space of solutions. Instead of using an arbitrary threshold, the RESOS algorithm implements a dynamic non-arbitrary percentile method to identify the set of relevant solutions.

The dynamic non-arbitrary percentile method was applied to different models to determine the threshold for relevant solutions for each model. The assessed GEMs *E. coli* (iJO1366) [24], CHO (iCHO1766) [27], Human (Recon1) [28] and CHO (iCHO2101) [25] represent different organisms with different model size and different levels of complexity. This heterogeneity in the models allows us to evaluate the robustness of the approach to changing conditions.

We found the optimal thresholds for *E. coli*, CHO (iCHO1766 and iCHO2101), and Human GEMs to be the 38th, 47th, 45th, and 50th percentiles, respectively. The algorithm discards solutions above these thresholds as outliers.

The algorithm determines the optimal distance between the central and farthest solutions, along with the number of solutions in between, to establish a threshold (percentile). These values can subsequently streamline computational analyses of the same network (e.g., simulating knockouts), as they are fewer and more precise than arbitrary thresholds.

#### 2.1.3. Assessing the Improvement of the Model’s Predictive Capabilities by Applying RESOS Algorithm

The RESOS algorithm aims to refine metabolic system characterization by pinpointing the densest region within the feasible flux solution space of a GEM, thereby influencing the model’s predictive capabilities. We assess how RESOS compares to the ROOM method, commonly used for metabolic flux prediction post-perturbation, such as gene or reaction knockouts [29]. Both algorithms were applied to predict gene essentiality and synthetic lethality in *E. coli* K12 using the iJO1366 GEM, with results compared to experimental data from the literature [30,31,32].

Synthetic lethality describes an interaction where two separate events, individually harmless to cell viability, together cause cell death. This phenomenon is studied to understand metabolic network interactions and potential drug targets, particularly in diseases like cancer [33].

To enhance analysis performance, ROOM and RESOS were integrated into the ParallelSL algorithm, a parallelized version of FastSL that employs FBA to predict essential reactions, pairs (synthetic lethals), and triplets [15]. A systematic comparison between ROOM and RESOS methods was conducted using the *E. coli* iJO1366 GEM (refer to Section 4, Methods). The model’s original boundaries remained unaltered, except for adjusting the lower bounds of oxygen and glucose exchange reactions to match experimental data [24]. Exchange reactions and the objective function (biomass) were excluded from analysis.

To validate the predictions, essential genes and synthetic lethals for the *E. coli* K12 strain were obtained from [30,31,32]. Genes were mapped to associated reactions using the findRxnsFromGenes() function in the Matlab R2022b COBRA Toolbox [34]. Since this function retrieves all reactions involving a gene, the resulting list required further filtering based on logical relations between genes, using the following criteria:

If gene_1_ encodes reaction_1_:

if gene_1_ ∈ essential genes → reaction_1_ ∈ essential reactions

If gene_1_ or gene_2_ encode reaction_1_:

if gene_1_ and gene_2_ ∈ essential genes → reaction_1_ ∈ essential reactions

If gene_1_ and gene_2_ encode reaction_1_:

if gene_1_ or gene_2_ ∈ essential genes → reaction_1_ ∈ essential reactions

A similar approach was used for synthetic lethals (SLs), where reactions were identified for each gene in a set. These reactions were then combined into pairs to obtain the final set of SLs.

**Figure 2:**
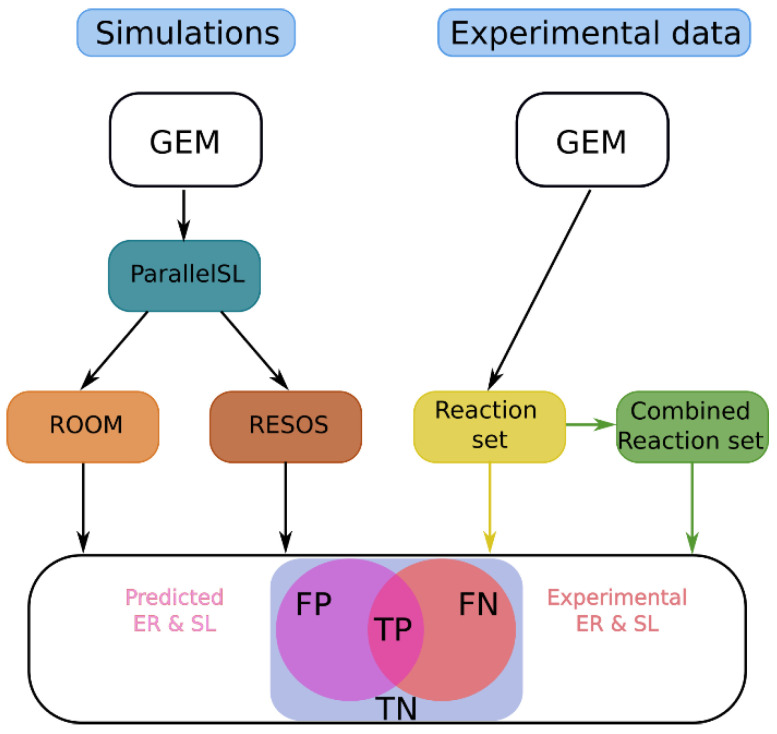
Schematic representation of the algorithm comparison: The color of the arrows indicates whether a particular step was performed to predict only ERs (yellow), SLs (green), or both (black).

Finally, the reaction set and their combinations corresponding to experimentally measured essential reactions and synthetic lethals, respectively, were compared against the predicted ones (Figure 2). The reaction set comprised a total of 2583 reactions, encompassing all reactions contained in the model. For synthetic lethals, the total considered was 17,159 combinations of the reaction set.

The prediction ratio was calculated using Equation (1):

(1) $$Prediction\;ratio=\frac{TP+TN}{TP+TN+FP+FN}$$

where TP, TN, FP, and FN are respectively “true positive”, “true negative”, “false positive”, and “false negative”. The analysis examines whether the deletion of a single or pair of reactions diminishes biomass production below a specified threshold, indicating compromised cell viability. In our study, this threshold was set at 5% of the optimal biomass production. Results demonstrate that implementing the RESOS algorithm enhances the model’s predictive capabilities by one percentage point. It is important to note that the iJO1366 GEM is a relatively compact model with a well-characterized metabolism focused on biomass production, rendering its predictions highly reliable regardless of the method employed, leaving little room for improvement. Nevertheless, even under these conditions, RESOS displayed notable performance enhancement, reducing error in essential reaction (ER) identification by 8.21%. Furthermore, the utilization of the RESOS algorithm substantially strengthens predictive abilities, reducing error in synthetic lethal predictions by 57.22%. Complete contingency tables are provided in Supplementary Material S1.

### 2.2. Assessing Sampling Algorithm Performance

Sampling feasible flux solutions in GEMs is essential for understanding system metabolism. However, the time required for this task grows exponentially with model size or when conducting a systematic analysis with numerous simulations, posing a significant computational challenge. To mitigate this issue, the presented pipeline incorporates the ADSB algorithm, aimed at drastically reducing computational time [20]. The performance of ADSB was assessed through a comparative analysis with the gpSampler algorithm, a well-established and widely used method implemented in Matlab [17]. Both ADBS and gpSampler algorithms were applied on two GEMs: the *E. coli* GEM EcoliCore [35] and the EC GEM iEC3006 [19]. These GEMs represent two different organisms with metabolic networks of different sizes and complexity offering a broader overview of the capabilities of both sampling algorithms. The *E. coli* model accounts for 95 reactions and 72 metabolites, while the EC model accounts for 3006 reactions and 2114 metabolites.

The computational time required for a specific process can be influenced by various external factors, which poses challenges when comparing simulations. To evaluate this, we compared the time taken to load the COBRA toolbox before each simulation, revealing nearly identical durations using the same machine. This observation indicates that the computational times for each calculation are indeed comparable and do not significantly impact the overall trend and conclusions drawn from the analysis.

Here, the number of sampling points was set to 10,000. The sampling time (in seconds) for the EcoliCore model was 45 and 72 for gpSampler and ADSB algorithms, respectively, while for the iEC3006 model, the sampling times were 8456 and 1056 for gpSampler and ADSB.

The sampling time of the *E. coli* model was 14-fold lower (1056/72) than the EC model with the ADSB sampling algorithm, and it was 188-fold lower (8457/45) when using the gpSampler algorithm. This shows that the running time of the gpSampler scaling is worse than ADSB when model complexity is increased (i.e., when analyzing a model with more reactions and metabolites).

When comparing the sampling time between algorithms applied to the same model, for the EC model, the ADSB is 8 times faster (8457/1056) than the gpSampler, and for the *E. coli* model, the gpSampler is 1.6 times faster (72/45) than the other sampling method. This suggests that the ADSB algorithm is best suited for complex models and that gpSampler performs best with simpler models. However, it is worth noting that the EcoliCore model is extremely small and simple compared to other GEMs. Considering this, while gpSampler has demonstrated to be only 60% faster than ADSB, it is crucial to recognize that the majority of cases involve much larger GEMs. Consequently, the ADSB algorithm remains the preferred choice due to its efficiency, especially when dealing with medium size and larger models.

### 2.3. Applying the Protocol to the Trauma Patient Cohort

#### 2.3.1. Patient-Specific metabolic Model Characterization

The protocol developed here was implemented on a cohort of 95 trauma patients [19]. Initially, we constructed a baseline model for EC metabolism using the iEC3006 model [19]. This EC-specific model, derived from Recon 1, incorporated observed metabolic capabilities gathered from literature references and databases [19].

Activation of the biomass reaction was enforced by setting its lower boundary to 50% of the optimal value. Furthermore, the RESOS algorithm determined an optimal threshold, resulting in a 50% reduction in sampling for subsequent patient-specific models.

Subsequently, relative metabolomic data from each patient were integrated by normalizing measured blood sample concentrations against control group metabolomics data. To address control group heterogeneity, three ratios—minimum, mean, and maximum—were calculated for each patient. These ratios were used to reconstruct three GEMs per patient (minimum, mean, and maximum), and the RESOS and ADBS algorithms were employed to characterize each patient’s metabolic flux profile.

**Figure 3:**
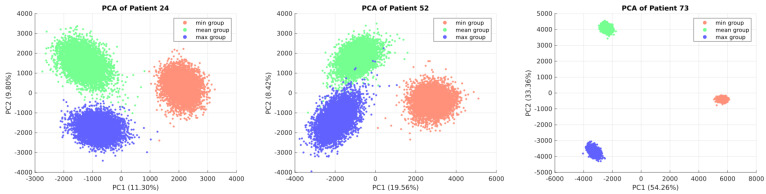
PCA of the minimum, mean, and maximum GEMs. The figure depicts PCAs illustrating the distribution of solutions obtained from three models employed to characterize the metabolic flux profiles of each patient (minimum, mean, and maximum). The plot presents PC1 and PC2 for patients 24, 52, and 73, serving as representative examples of the cohort.

The appropriateness of this approach was evaluated through PCA analysis of each patient’s metabolic flux profile. Figure 3 illustrates the distinct clustering of sampling points for patient numbers 24, 52, and 73 among models (minimum, mean, and maximum), highlighting an improved description of each patient’s EC metabolic capabilities through the integration of these three models. PCA for all patients’ flux profiles can be found in Supplementary Material S2.

#### 2.3.2. Validation of Patient-Specific GEMs

To assess the reliability of the patient-specific GEMs, cross-validation analysis was conducted. This involved reconstructing each patient-specific unconstrained model for each exchange reaction, calculating the population of feasible flux values using sampling analysis. Subsequently, the goodness of the predictions of each patient-specific model was determined by comparing the solutions from the unconstrained models with the initial constrained models, with significance assessed via t-test and FDR adjustment. In total, 5035 points were tested (53 measured metabolites × 95 patients). The analysis demonstrated high overall predictive accuracy, with 86.71% of metabolite uptake/secretion rates correctly predicted at a significance level of *p* < 0.01 (Supplementary Material S3).

### 2.4. Identify Common Metabolic Features in Trauma Patients Associated with Metabo-Groups with Different Mortality Rate

Once the patient-specific GEMs were characterized, we aimed to identify common metabolic features in trauma patients associated with metabo-groups exhibiting different mortality rates. The methodology utilized is outlined in detail below.

Drawing upon the characterized metabolic flux profiles of each patient, defined as the amalgamation of sampled solutions from the corresponding minimum, mean, and maximum models, we imposed these profiles as the metabolic boundaries of each patient-specific GEM. The patient-specific GEMs can be found in Supplementary Material S3.

**Figure 4:**
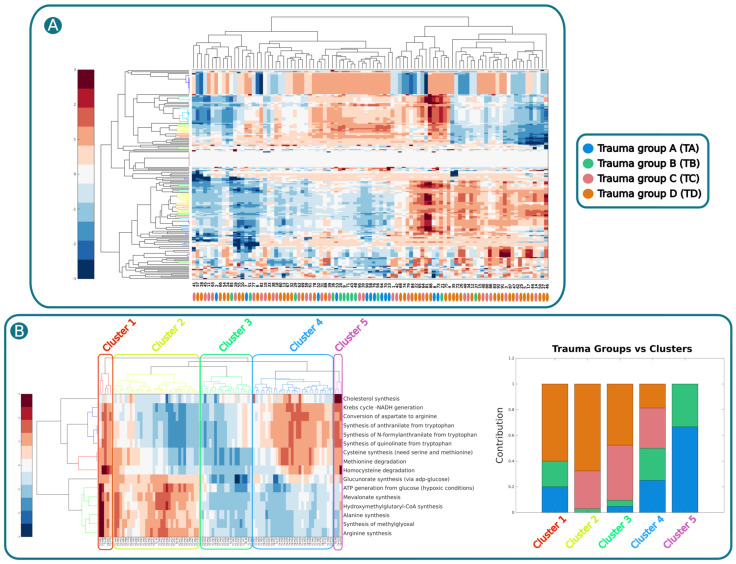
Clustering analysis. (**A**). Clustering analysis of the 190 task activities calculated for each patient-specific GEM. In the heatmap, red tones indicate overactivation, blue tones down activation, and white inactivity. Patient-specific GEMs are represented by the columns and the metabolic tasks in the row. Metabo-groups A, B, C, and D are represented by blue, green, pink, and yellow, respectively (heat map and metabo-group are interpreted in the same way in this figure). (**B**). From left to right: i. clustering analysis on all the patient-specific GEMs and the relevant metabolic tasks identified by the metabo-group-specific clustering analysis and five relevant column clusters; ii. bar plot representing the proportion of each metabo-group in each cluster.

Following this, the metabolic capabilities of each patient were inferred by applying a task analysis to the individual patient-specific GEMs with the sampling-based imposed boundaries. The task analysis involved a meticulous evaluation of the relevance of 190 manually curated metabolic tasks [16] on each patient-specific GEM, in accordance with the methodology outlined in Henriksen et al. [19]. The results were visually represented through heatmaps with dendrograms, applying Euclidean distance and the complete cluster algorithm [36] (Figure 4).

The initial analysis failed to cluster patients into the four metabo-groups as described in Henriksen et al., 2023 [17] (see Figure 4A). To gain deeper insights, we conducted the same clustering analysis on the same GEMs but now separated by metabo-groups and standardizing by row (See Figure 4B). This process internally standardizes the task activity values for each patient, positioning highly active tasks at the bottom of the heatmap and those with the lowest activity at the top. Next, we filtered metabolic tasks based on their consistent rank changes across metabo-groups categorized by mortality rate (Groups A, B, C, and D), as outlined by Henriksen et al. (2022) [19], ensuring a minimum 10-position difference between Groups A and D.

As a result, 16 metabolic tasks were identified as relevant, exhibiting changes in activity that correlated with mortality rate across different metabo-groups. The individual group clustering analysis revealed notable differences in the activity states of metabolic tasks, reminiscent of those described in Henriksen et al., 2022 [19]. For instance, an imbalance in ATP generation between glycolytic and oxidative phosphorylation pathways, as observed in the metabo-groups outlined by Henriksen et al., 2023, was also evident in this analysis [17]. Other relevant tasks that were identified were the synthesis of cholesterol, arginine, methiylglyoxal, alanine, hydroxymethylglutaryl-CoA, mevalonate, glucuronate, and cysteine, as well as the synthesis of anthranilate, N-formylanthranilate, and quinolinate from tryptophan and the degradation of methionine and homocysteine (Figure 4B).

To validate these findings, a new clustering analysis was performed, incorporating all patients but focusing solely on the 16 relevant metabolic tasks. This analysis revealed five clusters for columns (patients). Each cluster was then analyzed to determine the contribution of each metabo-group, with values ranging from 0 (no contribution) to 1 (full contribution) (Figure 4B).

Anomalies were observed in the first cluster, likely attributed to its small size. However, a clear trend emerged from Cluster 2 onwards. In metabo-group A, the abundance increased from 0 in Cluster 2 to 0.66 in Cluster 5. Similarly, metabo-group B exhibited an increase from 0.029 in Cluster 2 to 0.33 in Cluster 5. Group C displayed more oscillating behavior, likely due to its intermediate phenotype. Conversely, Group D, with the highest mortality rate, demonstrated a nearly identical trend but in the opposite direction to Group A (the group with the lowest mortality rate), decreasing from 0.68 in Cluster 2 to 0 in Cluster 5. Details of this analysis can be found in Supplementary Material S4.

### 2.5. Linking Plasma Metabolome with Endothelial Cell Metabolic Tasks for Biomarker and Target Discovery

**Figure 5:**
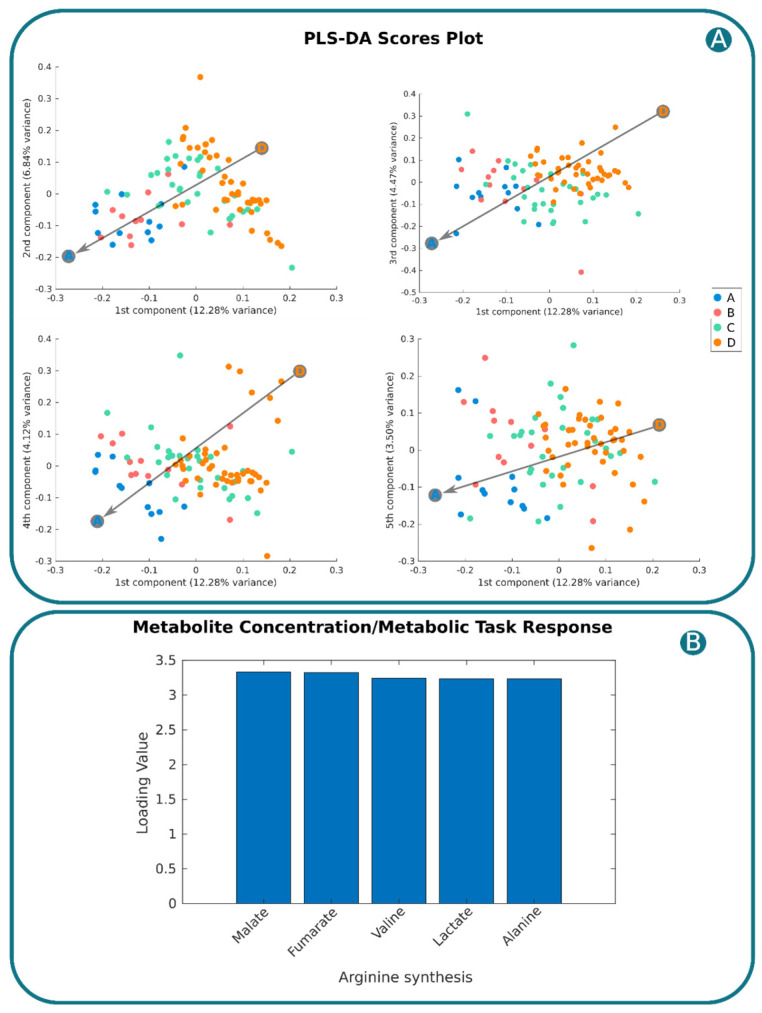
PLSA-DA. (**A**). Score plot of the PLSDA representing the 95 patients in Component 1 vs. Components 2, 3, 4. and 5. Metabo-groups A, B, C, and D are represented by blue, green, pink, and yellow, respectively. Grey arrow represents trend in the distribution of patients along the first component from group D to group A. (**B**). Top five task responses to plasma metabolite perturbation from the first 20 components of the PLSDA. Here is represented the loadings corresponding to the sensitivity of arginine synthesis towards perturbations of malate, fumarate, valine, lacatate, and alanine.

Given the direct interaction between endothelial cells and plasma, changes in plasma and endothelial cell metabolism are intimately connected. A sensitivity analysis evaluated how measured metabolite perturbations impact the 16 relevant metabolic tasks previously identified (see Section 4, Methods). Subsequently, clustering analysis, visualized in a heatmap with a dendrogram (Supplementary Material S5), yielded a cubic matrix containing responses of all 16 tasks to plasma metabolite perturbations across patients (16 tasks × 53 metabolites × 95 patients). This matrix was flattened to a 2D matrix (95 patients × 848 columns) for PLSDA, revealing patient stratification along the first principal component (Figure 5A). The first 20 components explained 62% of variance between groups. By analyzing these components, we identified the top five metabolic responses to plasma changes, with arginine synthesis showing the highest variance across groups. Notably, this metabolic function was found to be highly sensitive to changes in plasma levels of malate, fumarate, valine, lacatate, and alanine (Figure 5B). These findings suggest arginine synthesis and plasma malate, fumarate, valine, lacatate, and alanine as potential targets or biomarkers for improving patient outcomes.

## 3. Discussion

In this study, we undertook a comprehensive exploration of exo-metabolomics data integration and the development and implementation of novel algorithms that enhance the predictive capabilities of metabolic networks as well as improve the analysis performance in terms of computational time. More specifically, we have developed a novel algorithm to explore more efficiently the space of feasible flux solutions (RESOS) and have adapted and implemented the fast-sampling ADSB method. The pipeline development encompassed the use of exo-metabolomics and bibliomics from a number of sources as well as diverse models from a variety of organisms, including *E. coli*, humans, and Chinese Ovarian Hamster, evaluating the impact of RESOS and ADSB application on the analysis performance and model’s predictive capabilities.

The RESOS algorithm enables two approaches: continuous and discrete. Both approaches were assessed, unveiling the superiority of the continuous method in terms of accuracy, while the discrete approach required shorter computational time. This algorithm also enables us to dynamically determine the optimal number of sampling points. RESOS showcased its ability to characterize the metabolic flux profile efficiently while reducing the number of required sampling points between 50 and 62% which implies an important decrease in the computational time, which is especially crucial in studies where a large amount of simulations need to be carried out on the same GEM. Moreover, the algorithm integration significantly improved the predictive capabilities of the metabolic model, as evidenced by the comparison with the ROOM method in predicting gene essentiality and synthetic lethality in *E. coli* K12.

Our investigation also extended to the comparative analysis of two sampling algorithms: ADSB and gpSampler. Notably, ADSB showcased superior efficiency, particularly in complex models; it resulted in a significant 8.5-fold reduction in computational time, providing a substantial improvement in the overall efficiency of the metabolic network analysis. In addition, when considered in tandem with RESOS, which independently reduces computational time by 50–62%, the cumulative reduction in computational time can be between 17- and 22.4-fold, which is a substantial time reduction.

Both RESOS and ADSB demonstrate superior performance compared to similar existing methodologies, effectively reducing computational time and enhancing the reliability of model predictions. This is particularly crucial when studying cell metabolism using patients’ clinical data, where inherent noise in clinical samples can hinder model accuracy. Improving the reliability of model predictions enables the extraction of meaningful information from clinical datasets. Moreover, as this analysis entails sampling GEMs with increasing sizes [37], which demands more computational time, employing a rapid sampling algorithm like ADSB allows for the study of large datasets without compromising model quality.

In systems medicine, understanding ECs is crucial due to their central role in physiological processes and their implication in numerous diseases. The dynamic nature of EC metabolism poses a challenge in comprehensively characterizing their functions. This complexity arises from their heterogeneity, responsiveness to environmental cues, and involvement in processes like angiogenesis, inflammation, and tissue repair [22].

ECs are relevant in systems medicine, particularly in maintaining vascular homeostasis and contributing to diseases such as cardiovascular disorders, inflammatory conditions, and cancer. Dysregulation of endothelial metabolism is linked to endothelial dysfunction, a hallmark of various diseases. Therefore, unraveling EC metabolism is imperative for understanding disease mechanisms, identifying therapeutic targets, and advancing personalized medicine [10,11,12]. Exploring endothelial metabolism presents significant challenges. Obtaining viable samples from microvascular endothelium remains difficult due to tissue complexity, cell fragility, and the need to maintain the endothelial phenotype in vitro. Additionally, the scarcity of suitable techniques can lead to deviations from in vivo conditions [8].

Our novel computational pipeline addresses these challenges. Implemented on a trauma patient cohort, it integrates metabolomics data with RESOS and ABSD sampling algorithms [20], providing a nuanced understanding of patients’ EC metabolic capabilities. This application in trauma patients demonstrates the pipeline’s utility in a clinical context, elucidating metabolic alterations associated with critical conditions.

We successfully applied the pipeline to integrate metabolic measurements from patient blood samples, generating patient-specific GEMs to elucidate metabolic processes underlying different metabolic groups with significant differences in mortality rate.

The integration of exometabolomics data is crucial in trauma patients, allowing measurement of extracellular metabolites for insights into EC responses to traumatic stress. Our pipeline facilitates efficient and accurate integration of exometabolomics data, enabling precise characterization of metabolic flux profiles in trauma patients’ ECs.

In this context, our computational analysis revealed significant differences in the activity states of metabolic functions between metabo-groups. Some of these functions are already reported by Henriksen et al. (2023) [17] (see Figure 4B). For example, an imbalance in ATP generation between glycolytic and oxidative phosphorylation pathways, as observed in the metabo-groups outlined by Henriksen et al. (2023), was also evident in our analysis [17].

Other relevant metabolic functions with significant differences between metabo-groups are cysteine, alanine, glucuronate, mevalonate, methylglyoxal, and arginine synthesis and methionine degradation.

In trauma patients, cysteine synthesis is pivotal due to its role as a precursor of glutathione, a major antioxidant in the body. Given the heightened oxidative stress in trauma patients resulting from tissue injury and inflammation, maintaining sufficient cysteine levels is essential for preserving endothelial function and preventing vascular complications [38].

Glucuronate synthesis plays a vital role in detoxification processes. Trauma patients, exposed to numerous medications and endogenous metabolites requiring elimination, experience increased demand for glucuronate. Additionally, glucuronate contributes to the synthesis of hyaluronic acid, supporting endothelial integrity and wound healing [39].

Mevalonate synthesis is essential for sustaining endothelial viability and responsiveness to stimuli. Mevalonate serves as a precursor of cholesterol and other critical molecules, including geranylgeranyl pyrophosphate (GGPP), involved in various cellular processes crucial for endothelial function [40].

Methionine degradation yields homocysteine, which can cause endothelial damage and thrombosis; it also produces cysteine and glutathione, countering oxidative stress [41,42].

Methylglyoxal synthesis contributes to oxidative stress and inflammation in the endothelium by forming advanced glycation end products (AGEs). Elevated levels of methylglyoxal in trauma patients may lead to endothelial dysfunction and vascular complications [43].

Furthermore, our pipeline facilitates sensitivity analysis of patient-specific GEMs to elucidate the influence of metabolic blood composition on EC metabolism, revealing potential therapeutic targets and biomarkers. Notably, this analysis highlighted again the relevance of arginine synthesis in trauma patients, as this function presents the highest variation of sensitivity towards perturbations in plasma metabolome along the different metabo-groups, suggesting its potential role in patient outcomes [44].

Arginine is vital for endothelial health, serving as a substrate for nitric oxide (NO) synthesis, which regulates vascular tone and inflammation. However, trauma-induced upregulation of arginase-1 expression may reduce arginine availability for NO synthesis, impacting endothelial relaxation and protection [44].

Moreover, the significance of arginine in promoting NO production within ECs and its correlation with endothelial dysfunction [45,46,47] are particularly relevant in trauma patients, where this is a characteristic feature of shock-induced endotheliopathy (SHINE) [17,48].

Moreover, our analysis identified a high sensitivity of this metabolic function to alterations in malate, fumarate, valine, lactate, and alanine levels in the plasma metabolome. Several studies underscore the role of malate and fumarate in arginine metabolism, particularly in blood pressure regulation [49]. Deficiencies in fumarase and decreased malate levels can lead to reduced aspartate levels, subsequently impairing L-arginine regeneration and NO production [49]. This metabolic pathway suggests a connection between malate and hypertension [50]. Notably, L-arginine plays a crucial role in NO synthesis, a major vasodilator that enhances tissue blood flow [51].

Fumarate, another key player in arginine metabolism, has been implicated in inhibiting erastin-induced ferroptosis, a form of regulated cell death, by sustaining glutathione (GSH) biosynthesis through arginine deprivation [52]. The accumulation of fumarate in the cytosol stems from argininosuccinate cleavage due to the overexpression of argininosuccinate synthase (Ass1).

Valine, a branched-chain amino acid (BCAA), is recognized as a potent arginase inhibitor, exerting a significant regulatory role in arginine metabolism and demonstrating notable effects on the growth and differentiation of HT-29 cells [53,54].

The successful application of these methodologies in a clinical context, exemplified by the trauma patient cohort, underscores their potential for transformative contributions to personalized medicine and therapeutic interventions. This study lays the foundation for future research refining our understanding of cellular metabolism in health and disease, paving the way for innovative insights and applications in metabolic network analysis. Despite these results, the lack of correlation between all metabolic tasks and mortality rates suggests a potential absence of a linear relationship between metabo-groups and metabolism. Exploring machine learning analyses may unveil non-linear relationships, enabling nuanced patient stratification based on metabolic capabilities. Further clinical validation is crucial. Furthermore, scaling up GEMs and expanding the study to larger cohorts may pose challenges to scalability. However, these challenges could be addressed by implementing process parallelization, which could be further optimized by adapting the pipeline for GPU computing. Our computational analysis offers valuable insights into endothelial metabolism, but translating these into clinical practice demands rigorous experimental validation with patient cohorts, including clinical trials and longitudinal studies to confirm metabolic alterations in trauma patients and their clinical implications.

In conclusion, our computational pipeline advances understanding of EC metabolism in real-world clinical scenarios. The integration of exometabolomic data, coupled with advanced sampling algorithms, overcomes challenges posed by endothelial metabolism complexity, opening avenues for personalized medicine and therapeutic interventions. This work sets the stage for future investigations into EC metabolism in health and disease, contributing to the broader landscape of systems medicine research.

## 4. Materials and Methods

### 4.1. Protocol Overview

The presented protocol introduces a combination of existing and novel algorithms to effectively integrate exometabolomics data (i.e., patients’ plasma metabolome) into GEMs, enabling the elucidation of metabolic alterations.

The different approaches developed here have been tested with a diversity of GEMs as a proof of concept, to test and validate their capabilities. This subsection provides a general overview of the pipeline, while the subsequent subsections offer a more detailed description of each step.

Step 1: Baseline model characterization

**Figure 6:**
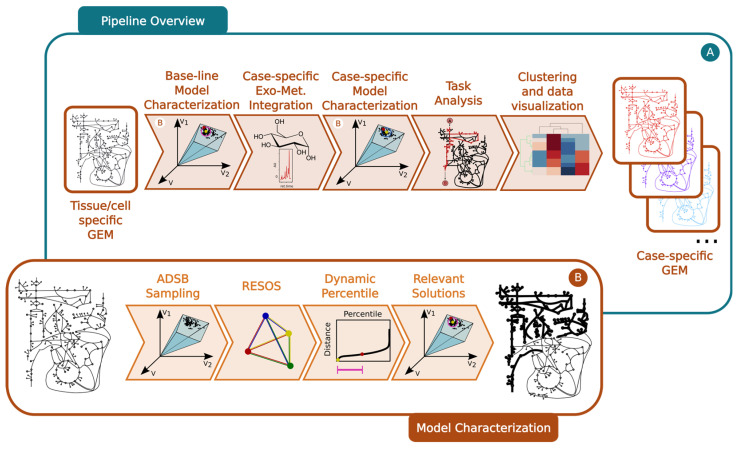
General overview of the developed pipeline: (**A**). Pipeline overview: i. Using a tissue/cell-specific GEM integrating the metabolic capabilities of the tissue/cell, a baseline model is characterized. This characterization includes maximum and minimum flux capabilities for each metabolic reaction in the model as well as RESOS parameters to identify the relevant optimal space of feasible flux solutions. ii. Case-specific exometabolomic data are integrated to modify the boundaries of the baseline model to reflect the case-specific metabolic capabilities. iii. Model characterization of the case-specific GEM by using RESOS parameters determined in the previous step. iv. Task analysis to identify the metabolic capabilities of each case-specific model. v. Clustering and visualization of task analysis results. (**B**). Detail on the model characterization process: i. ADSB Sampling algorithm is applied to identify solutions within the space of feasible optimal flux solutions of an FBA performed on a GEM. Next, the RESOS algorithm is applied to i. determine the sum of Euclidean distances between all the sampled solutions, ii. identify the set of relevant sampled solutions that better characterize the metabolic system by implementing the dynamic percentile approach, and iii. apply the parameters defined by the dynamic percentile to filter the relevant solutions from the space of feasible optimal flux solutions.

First, a baseline model is reconstructed and characterized to determine the maximum and minimum flux allowed for all the metabolic reactions. This process involves the following:

- Omics integration: The cell/organism physiological information is embedded into the baseline GEM using literature-based data, which sets boundaries on the exchange reactions (Figure 6A).
- Sampling space of solutions: The pipeline incorporates the ADSB algorithm, developed in 2024 by Saa et al. [20]. “This algorithm adeptly samples the space of feasible flux solutions for a given GEM, leading to a substantial reduction in computational time” (Figure 6B).
- Exploration of the space of feasible flux solutions using the RESOS algorithm: An in-house-developed algorithm is applied to analyze the sampled solutions and define the maximum and minimum flux allowed for each metabolic reaction. This algorithm identifies relevant regions and characterizes the metabolic flux spectrum without imposing arbitrary boundaries. Furthermore, the algorithm determines both the size and the number of solutions within the solution space to accurately profile the metabolic flux of a given network. These sampling parameters will be utilized in subsequent analyses, effectively reducing computational time.
- Outcome: The final outcome of this step is a characterization of the baseline GEM that includes the maximum and minimum flux allowed for all metabolic reactions (Figure 6B).

Step 2: Case-specific model characterization

- Here, the protocol integrates case-specific metabolomic data into the baseline model (Figure 6A). The following steps are involved:
- Omics integration: Relative exometabolomic data are integrated by calculating the ratio T_Mi_/C_Mi_, where T_Mi_ represents the concentration of the i^th^ metabolite in the T^th^ condition (e.g., disease group, treated group, or individual patient) and C_Mi_ represents the concentration of the same metabolite in the control group. To accommodate control group heterogeneity, three models are reconstructed for each condition, using either the minimum, maximum, or mean value of the i^th^ metabolite in the control group (C_Mi_) to calculate the relative metabolomic data (Figure 6A).
- Sampling: The same sampling algorithm employed in the baseline model is applied, using the sampling parameters previously determined by the RESOS algorithm in step 1 (Figure 6B).
- RESOS: The same algorithm used in the baseline model is applied for the case-specific analysis (Figure 6B). Outcome: As a result, three distinct GEMs are reconstructed for each case, and the case is characterized by combining the sampled solutions from the three models. This comprehensive approach provides a robust representation of the metabolic landscape for each specific case.

Step 3: Determining the metabolic capabilities of case-specific GEMs

- Generate a consensus case-specific GEM: First, the three previously reconstructed GEMs (minimum, mean, and maximum) are combined into a single case-specific GEM. This is achieved by adjusting the boundaries of the new GEM using the RESOS algorithm’s output, applied to the fusion of solutions from the three individual case-specific GEMs. The reliability of model reconstruction is ultimately assessed by cross-validating the exo-metabolomic experimental data with the model predictions [15].
- Task analysis: Then, the metabolic capabilities of the consensus case-specific GEMs are investigated by performing a task analysis [12] on a set of manually curated metabolic tasks [16] (Figure 6A).

Step 4: Results visualization and interpretation

To identify patterns and stratify the cases under study, a clustering analysis [18] in combination with a heat map representation and multivariate analysis is applied to the results obtained from the task analysis [19] (Figure 6A).

### 4.2. Main Case of Study

As a proof of concept, the pipeline outlined in the previous section was applied to a cohort of 95 critically ill adult trauma patients admitted directly from the scene of injury to the Red Duke Trauma Institute at Memorial Hermann Hospital, Houston, TX, USA, between March 2013 and February 2018. Blood samples were collected upon patient arrival. Patient selection was based on the Injury Severity Score (ISS) from a biorepository of over 6500 patients requiring the highest level of trauma activation. Patients with moderate to severe traumatic brain injury were excluded. Additionally, 20 healthy volunteers from Denmark were included to establish normal plasma metabolic variance for comparison with patient data [17].

### 4.3. Experimental Data

Throughout the development and application of the tools and algorithms developed and applied in the presented pipeline, different omics data from diverse sources were employed. The following provides an overview of the data utilized:

- Transcriptomics: *E. coli* essential and synthetic lethal genes: These data were used to evaluate the reliability of model predictions when applying the RESOS algorithm [30,31,32].
- Exometabolomics: Blood samples from a cohort of 95 trauma patients [17] and a control group of 20 healthy individuals were used for exometabolomic analysis. These data were integrated into the EC GEM iEC3006 to generate trauma patient-specific GEMs.

### 4.4. Genome-Scale Metabolic Network Models

Throughout the development and implementation of the diverse tools and algorithms within the presented pipeline, multiple GEMs were utilized to serve distinct purposes. The following GEMs were enlisted:

- EC GEM iEC3006 (latest reconstruction of EC metabolism) [19]:
  ○ To construct trauma patient-specific GEMs.
  ○ To assess the performance of the ADSB sampling algorithm.
- *E. coli* EcoliCore [35]:
  ○ To assess the performance of the ADSB sampling algorithm.
- *E. coli* K12 iJO1366 [24]:
  ○ To evaluate the enhancement in the reliability of model predictions when applying the RESOS algorithm.
  ○ To evaluate the dynamic percentile method implemented in the RESOS algorithm.
- General Human GEM Recon1 [28]:
  ○ To evaluate the dynamic percentile method implemented in the RESOS algorithm.
- CHO GEMs iCHO1766 [27] and iCHO2101 [25]:
  ○ To evaluate the dynamic percentile method implemented in the RESOS algorithm.

By utilizing these diverse GEMs, the pipeline was able to comprehensively assess the performance and reliability of the implemented algorithms across different biological systems, providing valuable insights into their effectiveness and applicability.

### 4.5. Exometabolomics Data Integration

Exometabolomics data from patients’ blood samples are often the only source of omics data available to study endothelium metabolism. In addition, these data are collected from a single time point, which makes the calculation of consumption/secretion rates impossible. To enable the integration of this source of information into a case-specific GEM reconstruction analysis, we have developed a computational approach to integrate relative exometabolomics data.

The process starts by reconstructing a baseline model, as previously explained. The baseline model is characterized by a sampling analysis using the ADSB algorithm [20], and the upper and lower thresholds of the exchange reactions are determined by applying the RESOS algorithm. This method also identifies the parameters required to characterize all of the following case-specific models.

Once the baseline model is characterized, and its exchange reaction boundaries are defined, we integrate the case-specific exometabolomic data by modifying the boundaries of the corresponding exchange reactions defined in the baseline model, as described in Equation (2).

(2) $${boundary}_{{Met}^{j^{th}},{Condition}^{i^{th}}}={boundary}_{{Met}^{j^{th}},Control}+\frac{{boundary}_{{Met}^{j^{th}},Control}}{\left|\left({boundary}_{{Met}^{j^{th}},Control}\right)\right|}*\;{boundary}_{{Met}^{j^{th}},Control}*\left(\frac{C_{{Met}^{j^{th}},{Condition}^{i^{th}}}}{C_{{Met}^{j^{th}},Control}}-1\right)$$

Here, the boundaries of the exchange reaction associated with the *j*^th^ measured metabolite in the *i*^th^ condition (${boundary}_{{Met}^{j^{th}},{Condition}^{i^{th}}}$) are defined as a function of the boundary of the same exchange reaction defined in the baseline model (${boundary}_{{Met}^{j^{th}},Control}$) and the concentration of the *j*^th^ metabolite in both the *i*^th^ condition and in a control group ($C_{{Met}^{j^{th}},{Condition}^{i^{th}}}$ and $C_{{Met}^{j^{th}},Control}$, respectively). In case the predicted exchange reaction flux in the baseline model is zero, upper and lower boundaries of 10 and −10 are set for the characterization of the case-specific GEM. The applicability of this approach can extend to the integration of other types of metabolomic data, including intracellular metabolomics or labeled metabolomics [54].

### 4.6. Sampling Algorithm

In this pipeline, we have incorporated a fast-sampling algorithm based on the Adaptive Directions Sampling on a Box (ADSB) algorithm, developed by Saa and Nielsen [20], which samples points from the feasible non-convex space. The algorithm employs a “shrinking” technique, gradually reducing the sampling space until a feasible flux distribution is obtained. This ensures a uniform sampling throughout the space, allowing for comprehensive exploration.

### 4.7. Exploring the Space of Feasible Flux Solutions Using RESOS Algorithm

The solutions obtained from sampling are all valid; however, it is unlikely to observe a uniform distribution within the solution space, and the presence of outlier solutions cannot be ignored. To this end, a novel algorithm for the Rational Exploration of Space Of Solutions (RESOS) is developed. In this subsection, we outline the methodology for assessing the density of the space of feasible flux solutions, while the subsequent subsection details the process for delineating the densest region, thereby providing a more accurate description of the metabolic system.

The algorithm identifies the most representative set of solutions corresponding to the densest region of solutions within the space of feasible flux solutions. The set of solutions is between two representative solutions, namely the “central” and “furthest” solutions.

The RESOS algorithm calculates the sum of distances among all sampled solutions, where a smaller overall sum indicates a denser region where the solution falls. Here, the central solution corresponds to the solution with the smallest sum of Euclidean distances within the same space of solutions. The furthest solution refers to the solution with the highest sum of Euclidean distances with the other solutions within the space of feasible flux solutions.

To identify the region with the highest concentration of solutions, two methods have been developed: continuous and discrete.

The discrete method is based on the frequency of reaction flux values within the sampled solutions. Solutions located in the densest regions tend to exhibit similar flux values for reactions compared to solutions in less dense areas. By comparing the frequency of possible values for each reaction, outliers can be identified. This method involves generating a histogram for each reaction and summing the frequencies across all solutions. Solutions with the highest total frequency values are located in the densest regions. Normalization is applied to ensure that the sum of the areas of each histogram bin always adds up to one.

On the other hand, the continuous method provides a more straightforward approach by calculating the Euclidean distance of each solution against every other solution. This allows for a direct comparison of distances and helps identify the central and furthest solutions within the solution space.

By employing both the discrete and continuous methods, the pipeline enables the identification of regions with the highest concentration of solutions. These methods provide complementary insights into the structure and characteristics of the solution space, facilitating the recovery of representative solution sets for further analyses. The distance between two solutions (vectors) is computed by applying Equation (3).

(3) $$d\bar{AB}=\sqrt{\sum_{i=1}^{n}{\left(A_{i}-B_{i}\right)}^{2}}$$

where n represents the number of reactions, and A and B are two different flux vectors corresponding to two different sampled solutions. The outcome is a squared symmetrical matrix in which the number of rows and columns corresponds to the number of sampled solutions. The sum of distances in each solution shows how far or close it is with respect to the other solutions. An example of a symmetrical matrix produced by the continuous method can be found in Table 1. This method also incorporates normalization, where the total Euclidean distance per solution is divided by the sum of all Euclidean distances.

In both methods, once frequencies or distances are computed, the next step is to filter the solutions to determine the set of relevant solutions that characterize the metabolic system.

### 4.8. Identifying the Set of Representative Flux Solutions by Applying a Dynamic Non-Arbitrary Percentile Threhold Method

The set of relevant solutions is defined as the set between the central and the furthest solutions. While the central solution is defined as the solution with the lowest sum of Euclidean distances with the other solutions, to define the furthest solution, a threshold is required.

To this end, the RESOS algorithm incorporates a dynamic non-arbitrary percentile threshold method. The dynamic percentile is based on the difference between the total Euclidean distances in the furthest and central solutions. In other words, it is based on how far the central and furthest solutions are from each other.

To obtain a dynamic percentile, the algorithm tests every possible percentile from the 1st to 100th. Within each test, the algorithm recovers the number of solutions generated and computes the difference between the total Euclidean distance or total frequency between the furthest and central solution, depending on whether the used method is the continuous or discrete approach, respectively. These results can be represented graphically in a curve that represents its second derivative, the inflection point where the curve’s concavity changes. This change in the curve’s trend indicates that after that point, the distance between the central and furthest solution will tend to increase. Thus, after the inflection point, the risk of considering outliers increases continuously. As a consequence, the percentile closest to the inflection point is chosen as the threshold, and all the solutions after this point are considered outliers.

After the dynamic threshold is defined, it is saved for subsequent analyses with the same model. The data recovered are as follows: i. Euclidean distance between furthest and central solution, and ii. number of valid solutions returned by the dynamic percentile. In other words, the attributes that the algorithm uses to define a representative set of solutions for a given model are the size of the space containing the solutions and its density. In subsequent sampling analyses with the same model (i.e., in a knockout analysis or characterizing case-specific models relative to a baseline model), sampling is performed until the values for the sampling parameters previously described are reached (distance between central and furthest solution and density of solutions between these points).

### 4.9. Task Analysis

To explore the metabolic capabilities of the reconstructed GEMs, we utilized a collection of 190 manually curated metabolic tasks [16]. These tasks represent specific goals that cells must achieve by producing certain metabolic products from a set of substrates. Unlike predefined metabolic pathways that include all the reactions, here we focus on defining the source substrates and the final products.

To investigate the metabolic task, FBA was applied [55]. FBA allows us to measure the flow of substances within the cell’s metabolic network by quantifying the maximum number of substances that can be exchanged between different groups of metabolites, considering specific constraints. This analysis was performed using the testPathway function within the Matlab COBRA toolbox.

### 4.10. Clustering Analysis

Task analysis results are represented graphically in heatmaps with dendrograms by applying Euclidian distance and the complete cluster algorithm using the clustergram package in MATLAB R2022b [18].

### 4.11. Unveiling Potential Therapeutic Targets by Applying Sensitivity Analysis

To understand how changes in plasma metabolic composition impact EC metabolism, we perform a sensitivity analysis. This entails a comprehensive task analysis for each measured plasma metabolite, covering the flux spectrum of corresponding exchange reactions. We calculate flux through cellular tasks while varying flux through individual exchange reactions within their boundaries (totaling 100 iterations). Then, the resulting table, indicating each metabolic task’s response to different metabolic levels, is normalized, rescaled for gradient slope calculation, clustered, and visually represented using MATLAB’s (R2022b) clustergram function [18]. This process is repeated for each patient-specific GEM, resulting in a cubic matrix containing the response of all metabolic tasks to alterations in all measured plasma metabolites across all patients (metabolic task × extracellular metabolites × patients). Subsequently, the cubic matrix is flattened to a 2D matrix, and PLS-DA is applied to identify relevant tasks and metabolites that best explain the differences in metabolic task responses to plasma metabolite perturbations.

## 5. Conclusions

In this study, we present a computational pipeline that offers a robust and innovative approach, enabling efficient integration of exometabolomic data into GEM analyses, thus providing a comprehensive understanding of cell metabolism.

Key novelties include the development of a pipeline for integrating patients’ blood sample metabolomics, along with the creation and adaptation of computational tools such as the RESOS algorithm and the general adaptation of the ADSB algorithm. By considering control group variability and providing an efficient method for integrating blood sample metabolomic data into GEM analyses, our pipeline enhances the accuracy of metabolism depiction.

Our approach offers a practical solution for constructing patient-specific GEMs, enabling the accurate characterization of metabolic flux profiles in trauma patients’ endothelial cells (ECs). We identified significant differences in the activity states of relevant metabolic functions, indicative of underlying metabolic dysregulations associated with traumatic stress. Arginine synthesis has emerged as a potential therapeutic target for improving patient outcomes, with sensitivity analysis confirming this finding and identifying plasma metabolites with high differences in their capability to modulate arginine synthesis activity between metabo-groups. These metabolites could serve as potential biomarkers for patient stratification or as effective modulators of arginine metabolism aiming to improve patient outcomes.

While certain metabolic tasks show promising correlations with mortality rates, the lack of a consistent linear relationship across all tasks suggests the potential presence of non-linear interactions. Further exploration, such as machine learning analysis, could provide valuable insights into nuanced patient stratification based on metabolic capabilities.

The successful application of our methodologies in a clinical context, exemplified by the trauma patient cohort, underscores their transformative potential in personalized medicine and more efficient therapeutic interventions.

Overall, our work contributes to advancing understanding of cellular metabolism in health and disease. By integrating exometabolomic data into GEM analyses, we establish a robust foundation for future research in systems medicine [56]. This interdisciplinary effort relies on iterative collaboration among clinical investigations, experimental models, and computational analyses, with the potential to revolutionize metabolic network analysis and pave the way for innovative approaches to personalized disease management and treatment.

## Figures and Tables

**Table 1:** Example of a symmetrical matrix when four valid solutions are given by the sampling. Here, the Euclidean distance is calculated between all the pairs (i.e., *d*12, *d*13, …) and the overall distance for each solution is calculated by summing all the terms in the corresponding column.

	1	2	3	4
1	0	$d\bar{12}$	$d\bar{13}$	$d\bar{14}$
2	$d\bar{12}$	0	$d\bar{23}$	$d\bar{24}$
3	$d\bar{13}$	$d\bar{23}$	0	$d\bar{34}$
4	$d\bar{14}$	$d\bar{24}$	$d\bar{34}$	0

## Data Availability

https://github.com/biosustain/GEM-Exometabolome, accessed on 1 March 2024.

## Supplementary Materials

The supporting information can be downloaded at: https://www.mdpi.com/article/10.3390/ijms25105406/s1.
