# Supplementary material for: Integrating Genome-Scale Metabolic Models with Patient Plasma Metabolome to Study Endothelial Metabolism In Situ
Source: Int J Mol Sci. 2024 May 15;25(10):5406. doi: 10.3390/ijms25105406 (PMC11121795; doi:10.3390/ijms25105406)
Supplement: Supplementary file 1 [file ijms-25-05406-s001.zip › Silva-Lance et al-Supplementary Material 2.pdf]

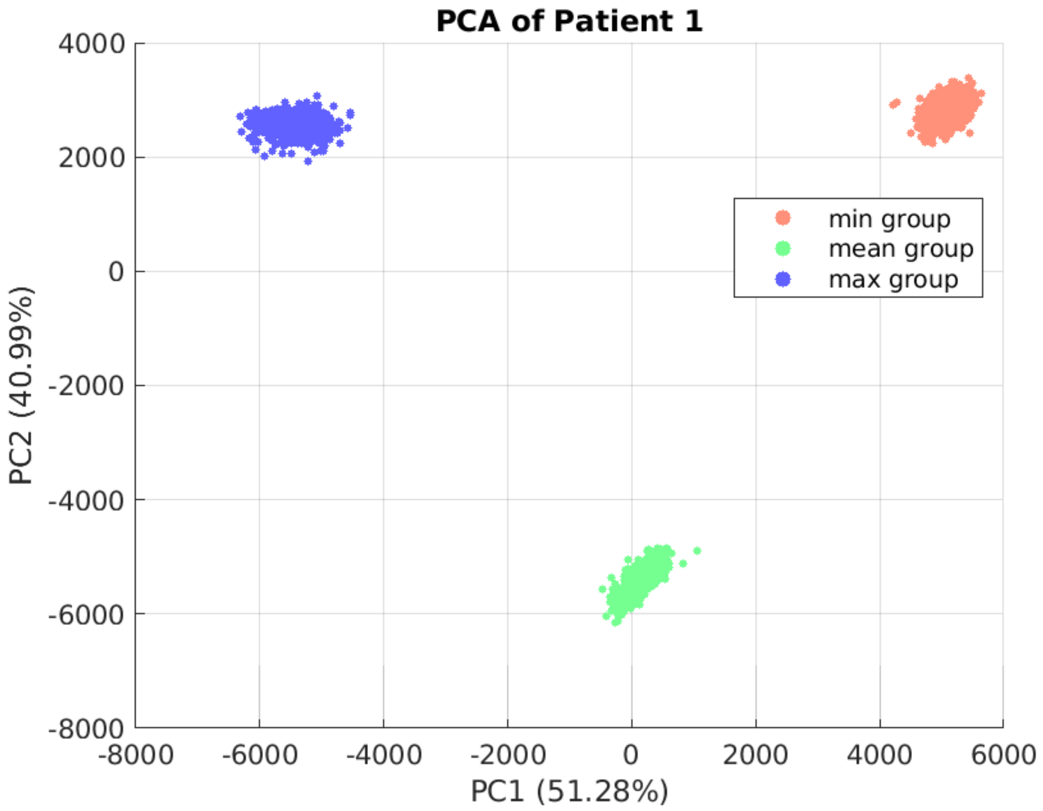
PCA of Patient 1
4000
2000
0
-2000
-4000
-6000
-8000
-8000
-6000
-4000
-2000
0
2000
4000
6000
PC2 (40.99%)
PC1 (51.28%)
min group
mean group
max group

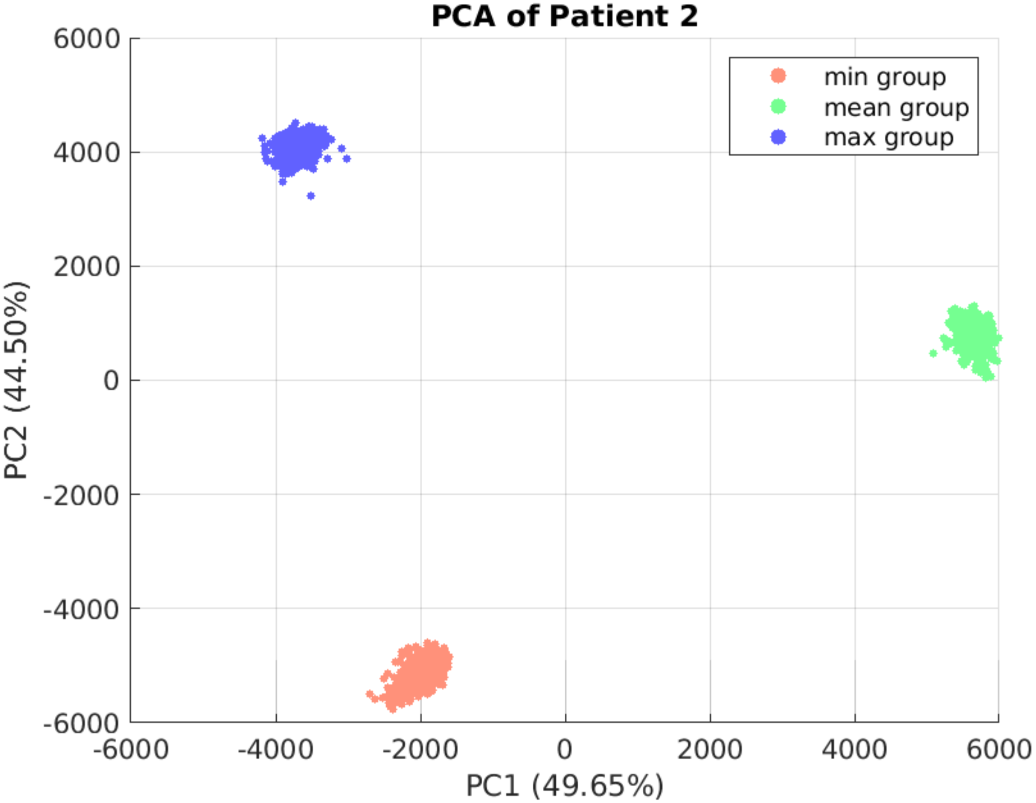

PCA of Patient 2
min group
mean group
max group
6000
4000
2000
0
-2000
-4000
-6000
-6000
-4000
-2000
0
2000
4000
6000
PC1 (49.65%)
PC2 (44.50%)

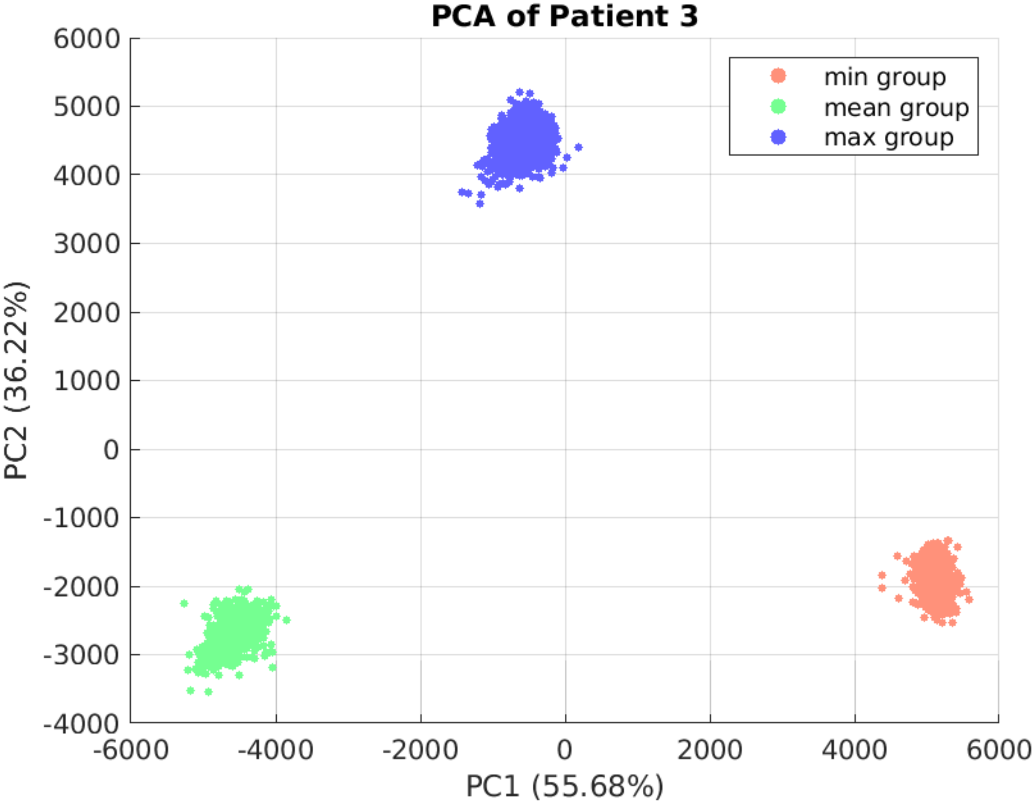
PCA of Patient 3
min group
mean group
max group
PC1 (55.68%)
PC2 (36.22%)
6000
5000
4000
3000
2000
1000
0
-1000
-2000
-3000
-4000
-6000
-4000
-2000
0
2000
4000
6000

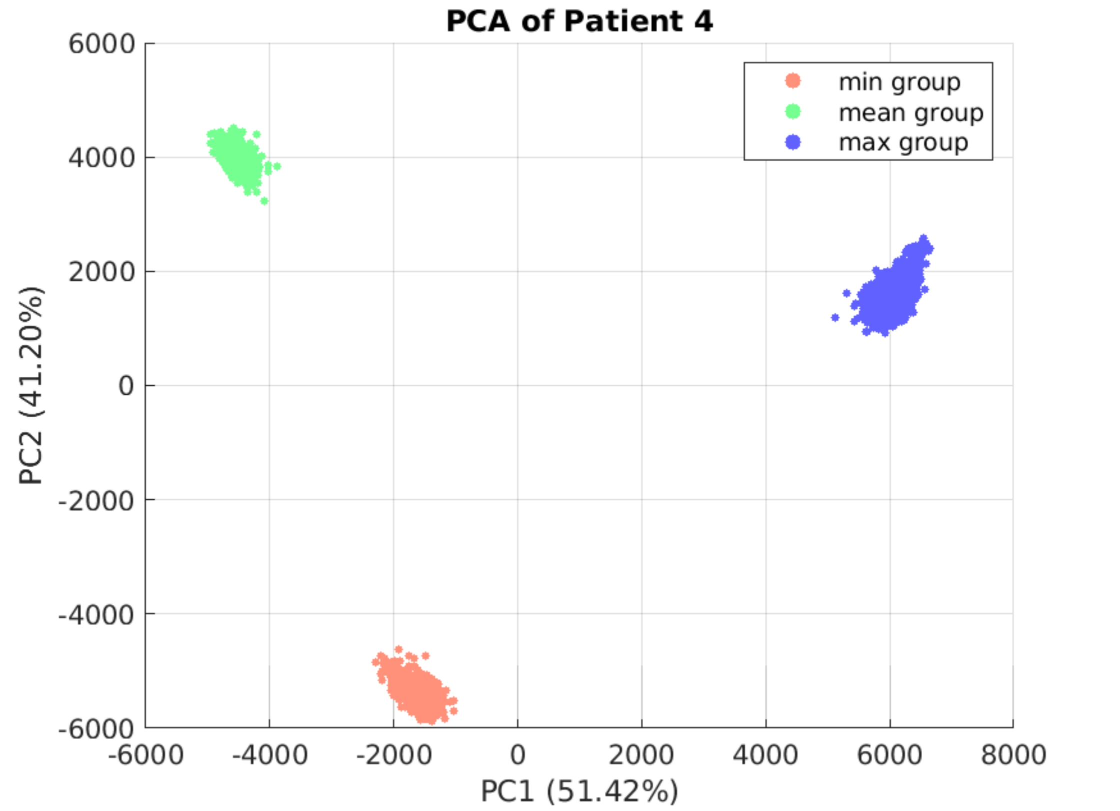
PCA of Patient 4
min group
mean group
max group
PC1 (51.42%)
PC2 (41.20%)
6000
4000
2000
0
-2000
-4000
-6000
-6000
-4000
-2000
0
2000
4000
6000
8000

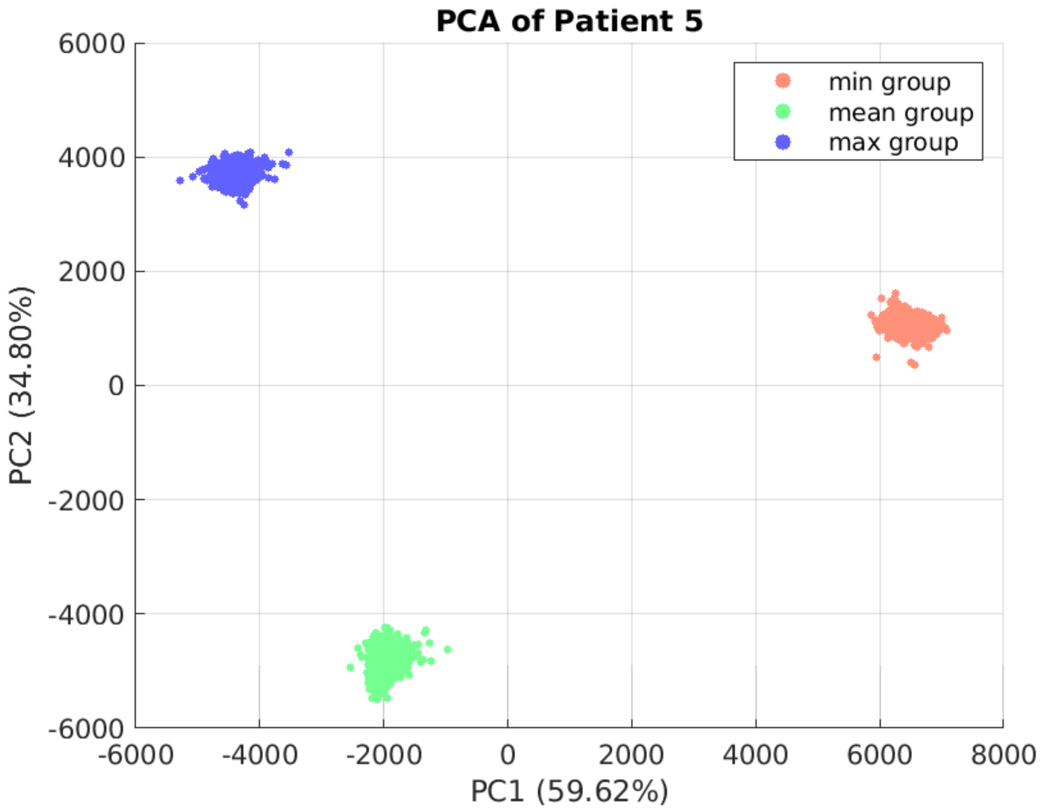
PCA of Patient 5
min group
mean group
max group
6000
4000
2000
0
-2000
-4000
-6000
-6000
-4000
-2000
0
2000
4000
6000
8000
PC1 (59.62%)
PC2 (34.80%)

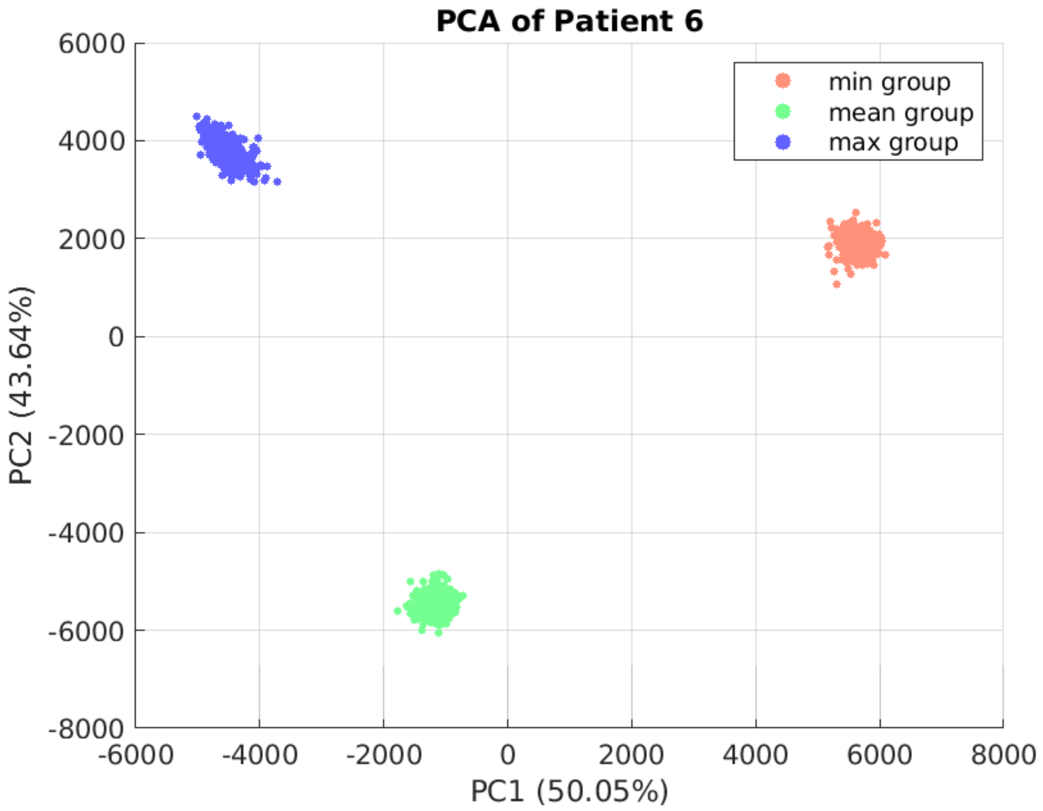
PCA of Patient 6
min group
mean group
max group
PC1 (50.05%)
PC2 (43.64%)
6000
4000
2000
0
-2000
-4000
-6000
-8000
-6000
-4000
-2000
0
2000
4000
6000
8000

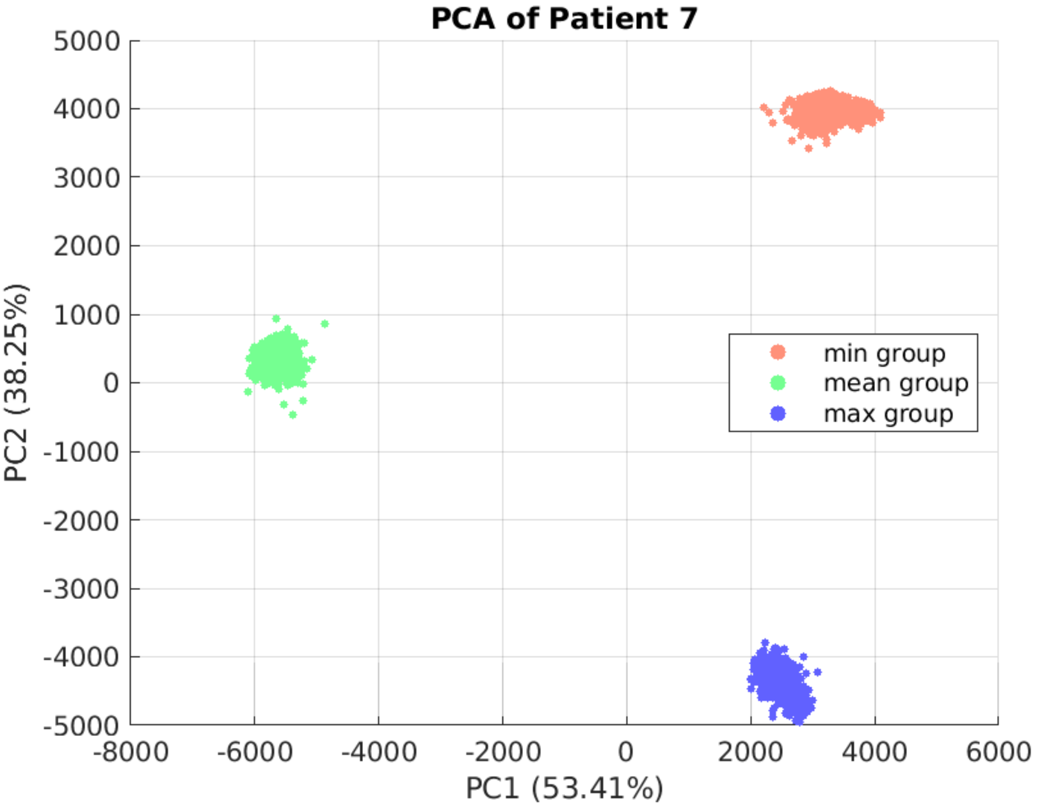
PCA of Patient 7
5000
4000
3000
2000
1000
0
-1000
-2000
-3000
-4000
-5000
-8000
-6000
-4000
-2000
0
2000
4000
6000
PC1 (53.41%)
PC2 (38.25%)
min group
mean group
max group

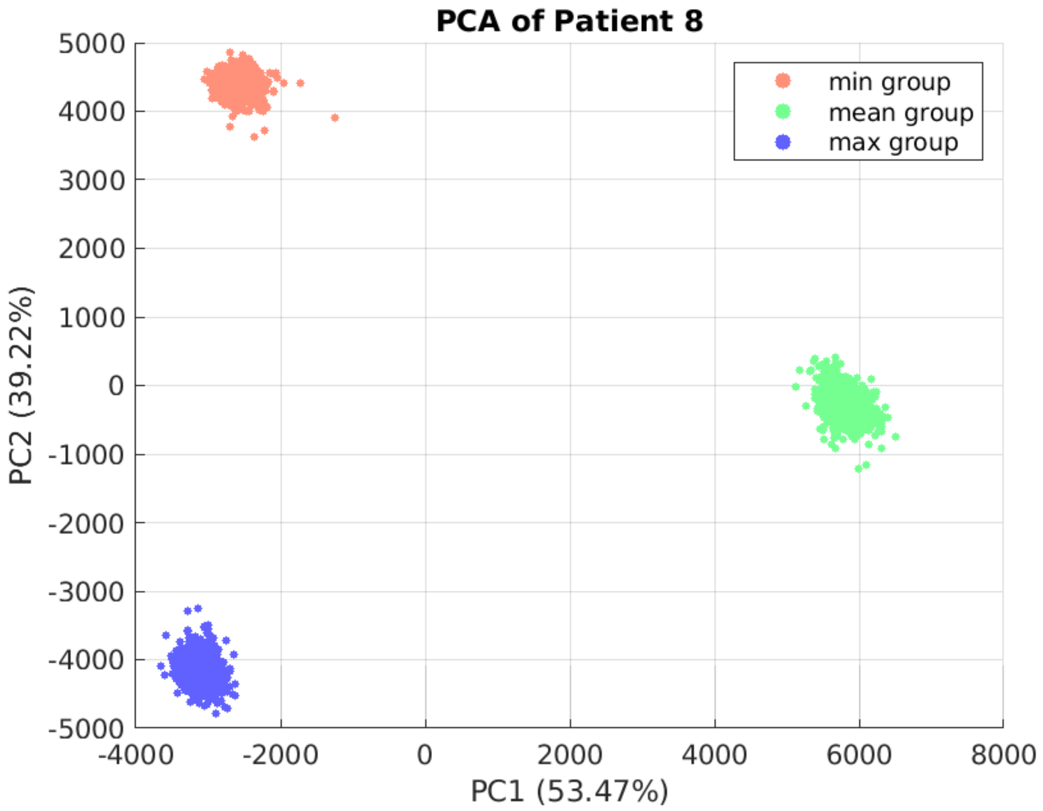
PCA of Patient 8
min group
mean group
max group
5000
4000
3000
2000
1000
0
-1000
-2000
-3000
-4000
-5000
-4000
-2000
0
2000
4000
6000
8000
PC2 (39.22%)
PC1 (53.47%)

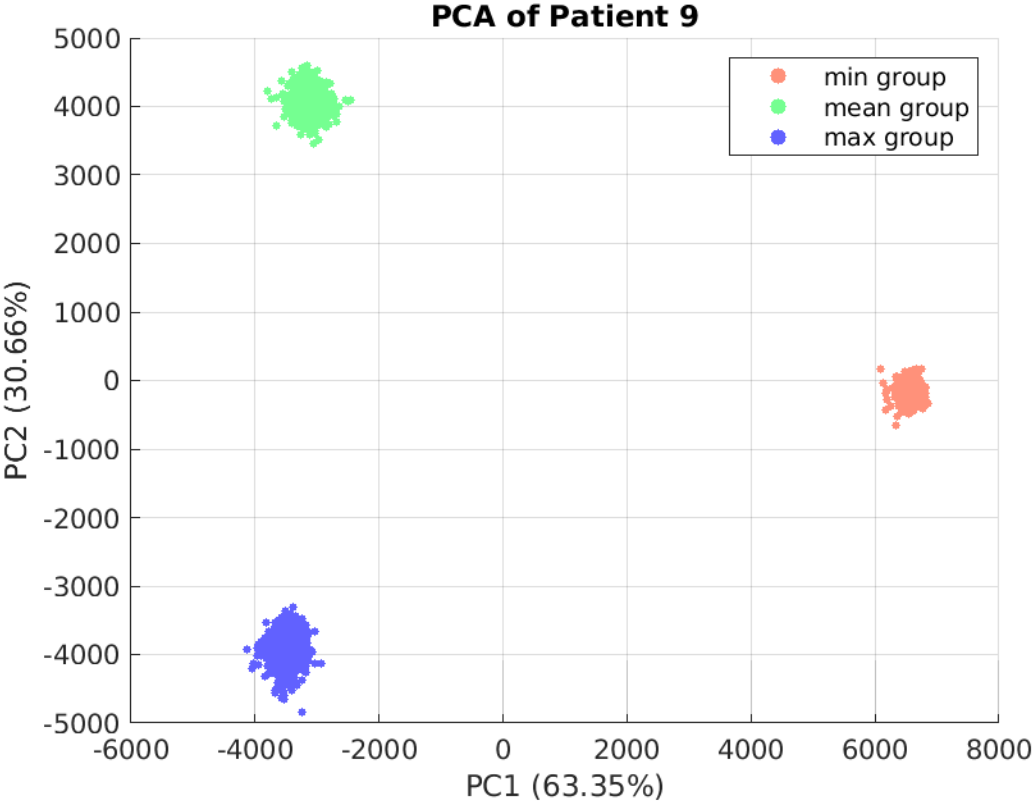
PCA of Patient 9
min group
mean group
max group
PC1 (63.35%)
PC2 (30.66%)
5000
4000
3000
2000
1000
0
-1000
-2000
-3000
-4000
-5000
-6000
-4000
-2000
0
2000
4000
6000
8000

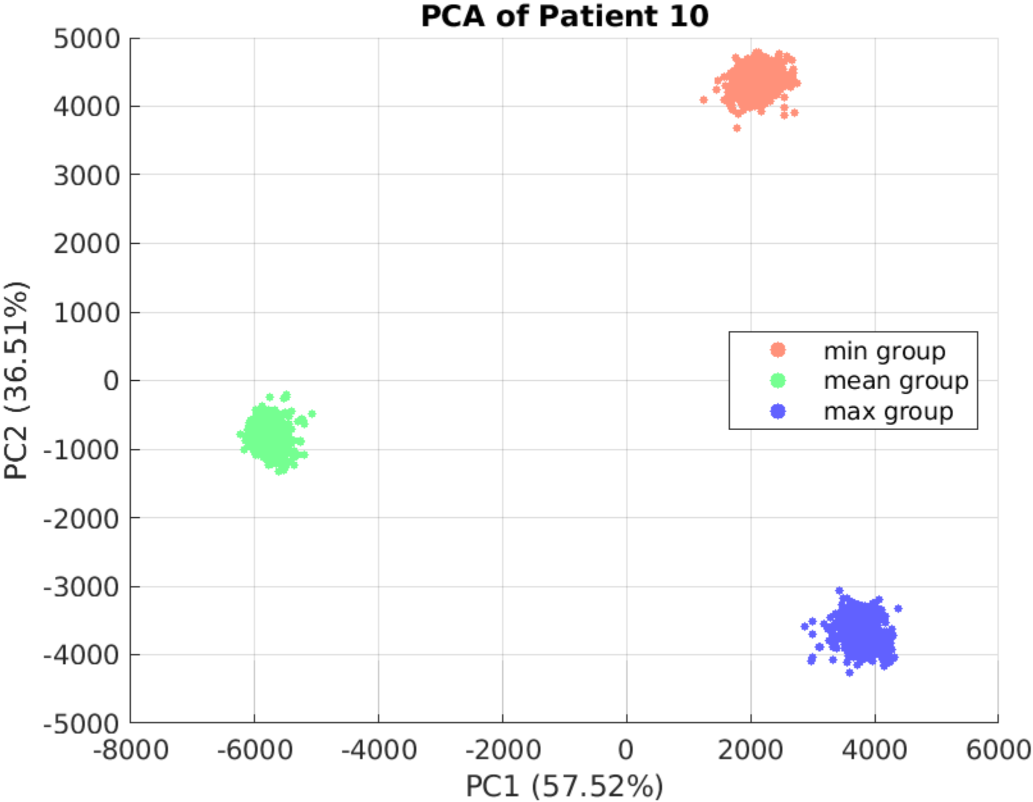
PCA of Patient 10
PC2 (36.51%)
PC1 (57.52%)
5000
4000
3000
2000
1000
0
-1000
-2000
-3000
-4000
-5000
-8000
-6000
-4000
-2000
0
2000
4000
6000
min group
mean group
max group

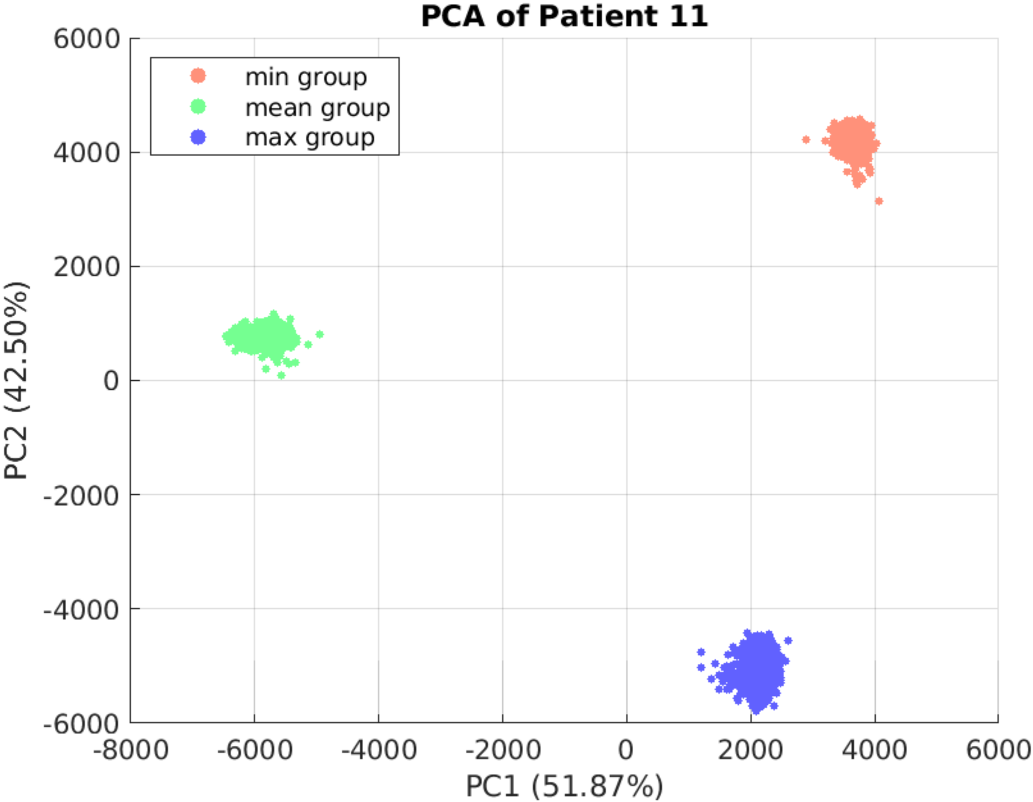
PCA of Patient 11
min group
mean group
max group
6000
4000
2000
0
-2000
-4000
-6000
-8000
-6000
-4000
-2000
0
2000
4000
6000
PC1 (51.87%)
PC2 (42.50%)

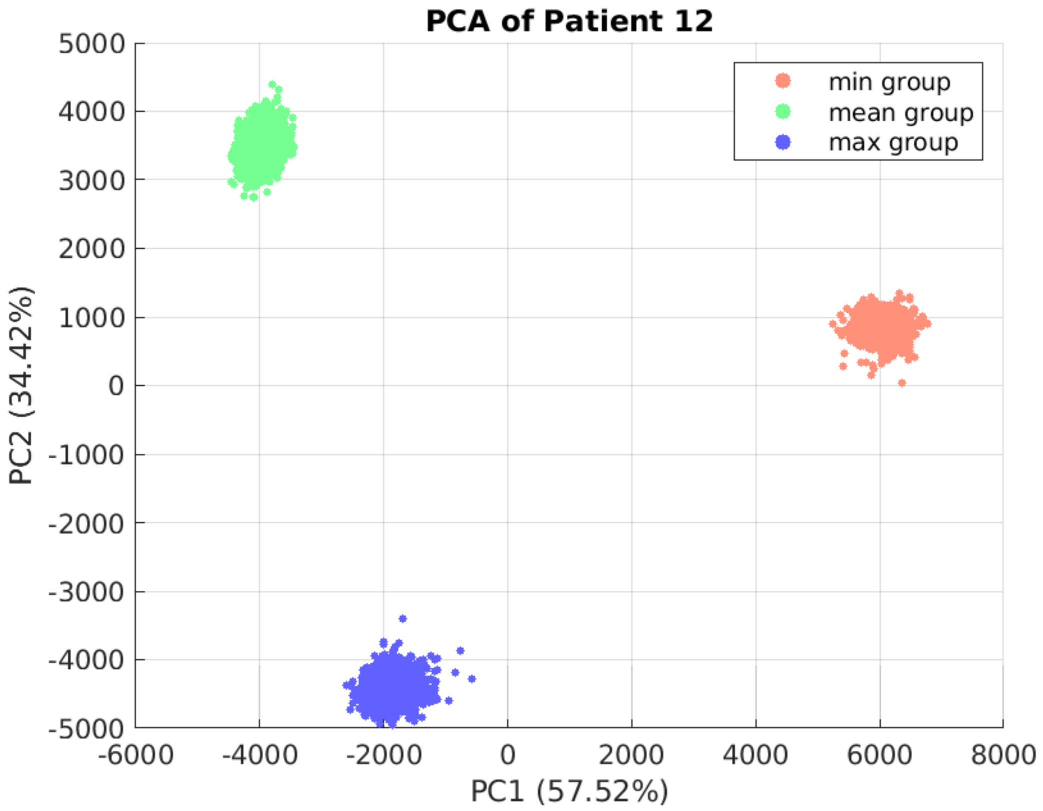
PCA of Patient 12
min group
mean group
max group
PC1 (57.52%)
PC2 (34.42%)
-6000
-4000
-2000
0
2000
4000
6000
8000
5000
4000
3000
2000
1000
0
-1000
-2000
-3000
-4000
-5000

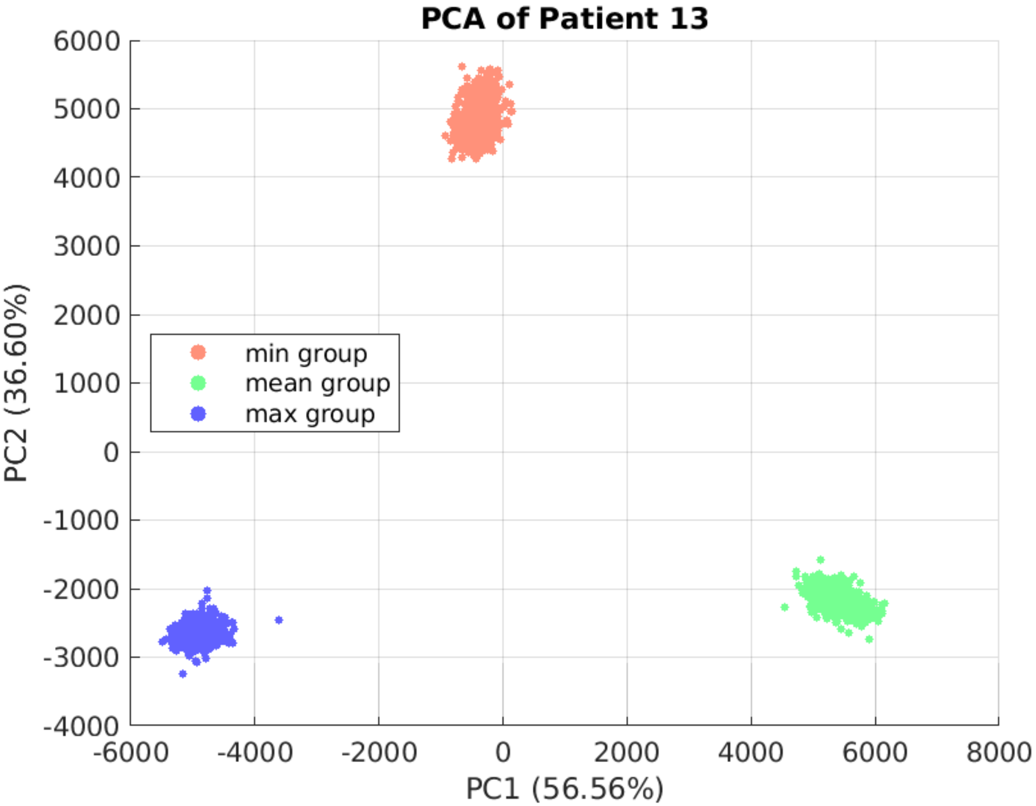
PCA of Patient 13
min group
mean group
max group
PC1 (56.56%)
PC2 (36.60%)
6000
5000
4000
3000
2000
1000
0
-1000
-2000
-3000
-4000
-6000
-4000
-2000
0
2000
4000
6000
8000

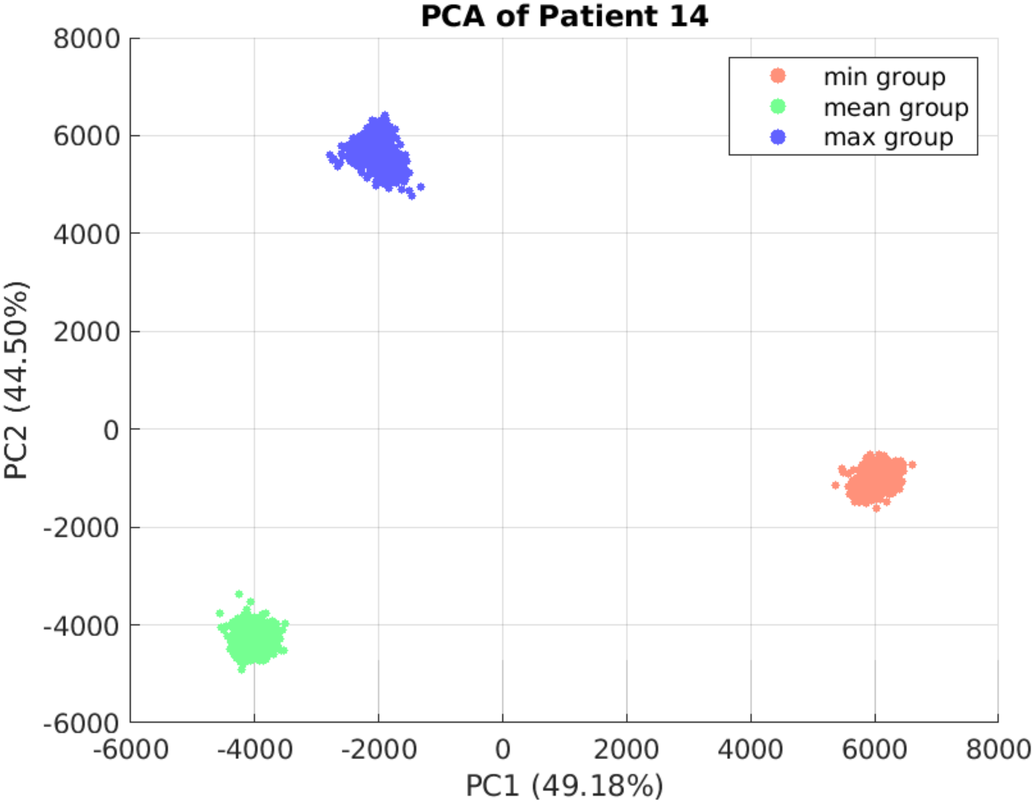
PCA of Patient 14
min group
mean group
max group
PC1 (49.18%)
PC2 (44.50%)
8000
6000
4000
2000
0
-2000
-4000
-6000
-6000
-4000
-2000
0
2000
4000
6000
8000

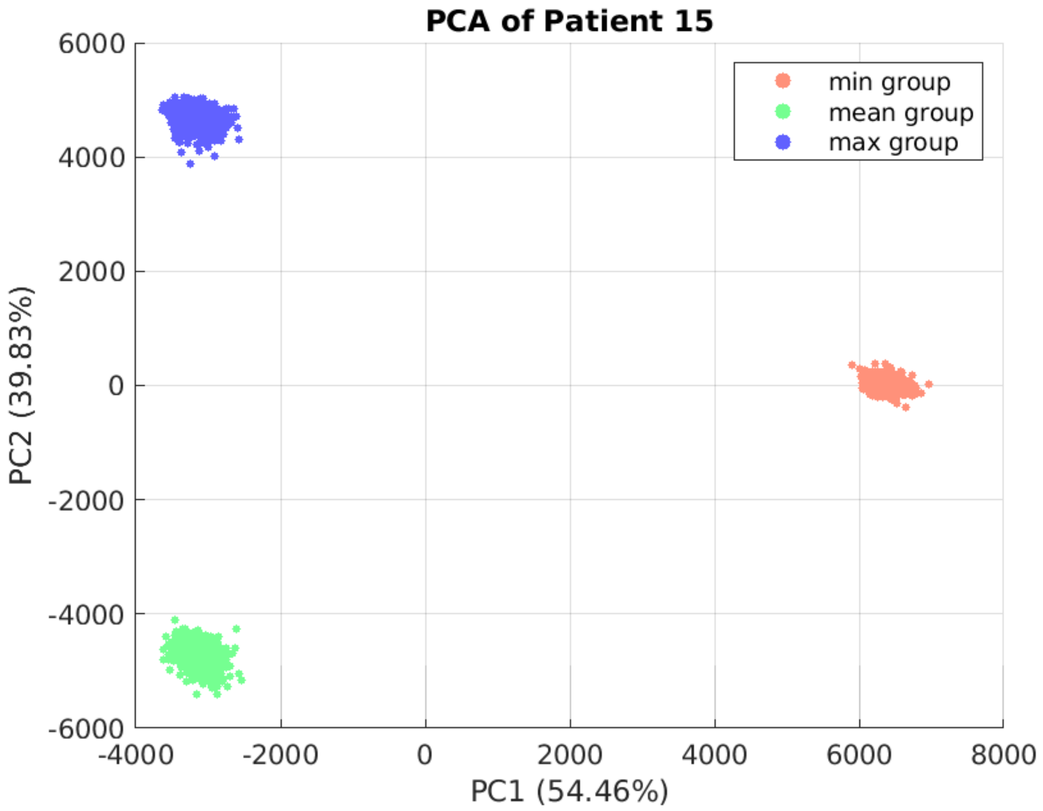

PCA of Patient 15
min group
mean group
max group
6000
4000
2000
0
-2000
-4000
-6000
-4000
-2000
0
2000
4000
6000
8000
PC2 (39.83%)
PC1 (54.46%)

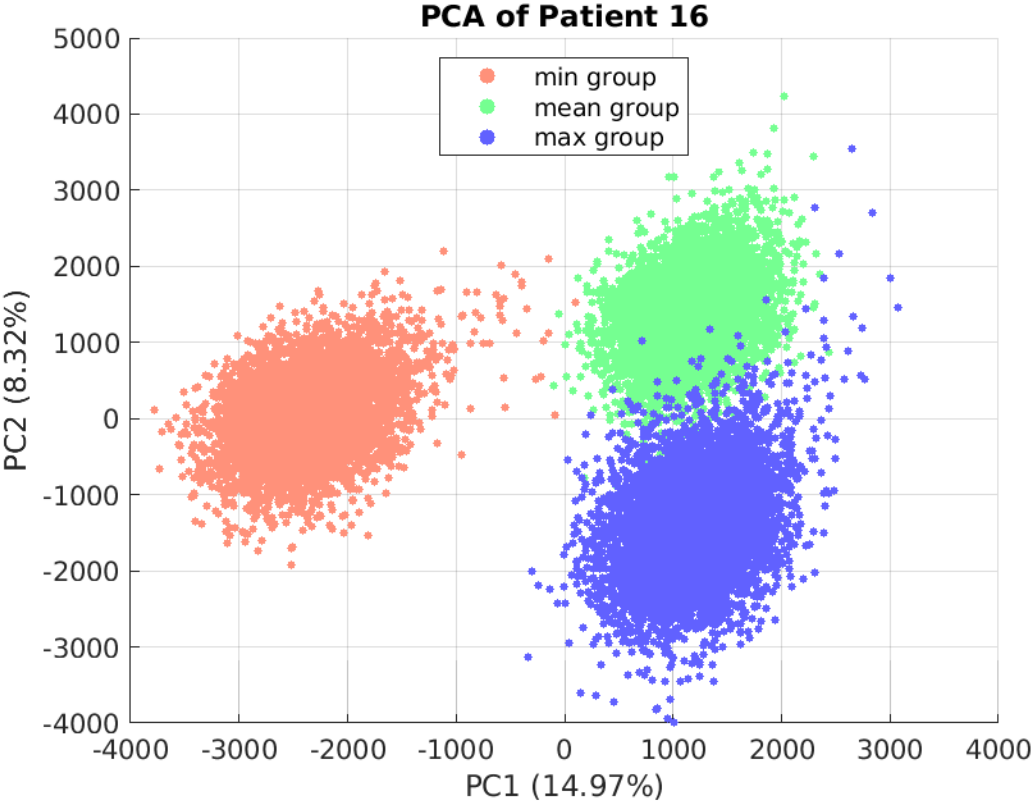

PCA of Patient 16
min group
mean group
max group
PC2 (8.32%)
PC1 (14.97%)
5000
4000
3000
2000
1000
0
-1000
-2000
-3000
-4000
-4000
-3000
-2000
-1000
0
1000
2000
3000
4000

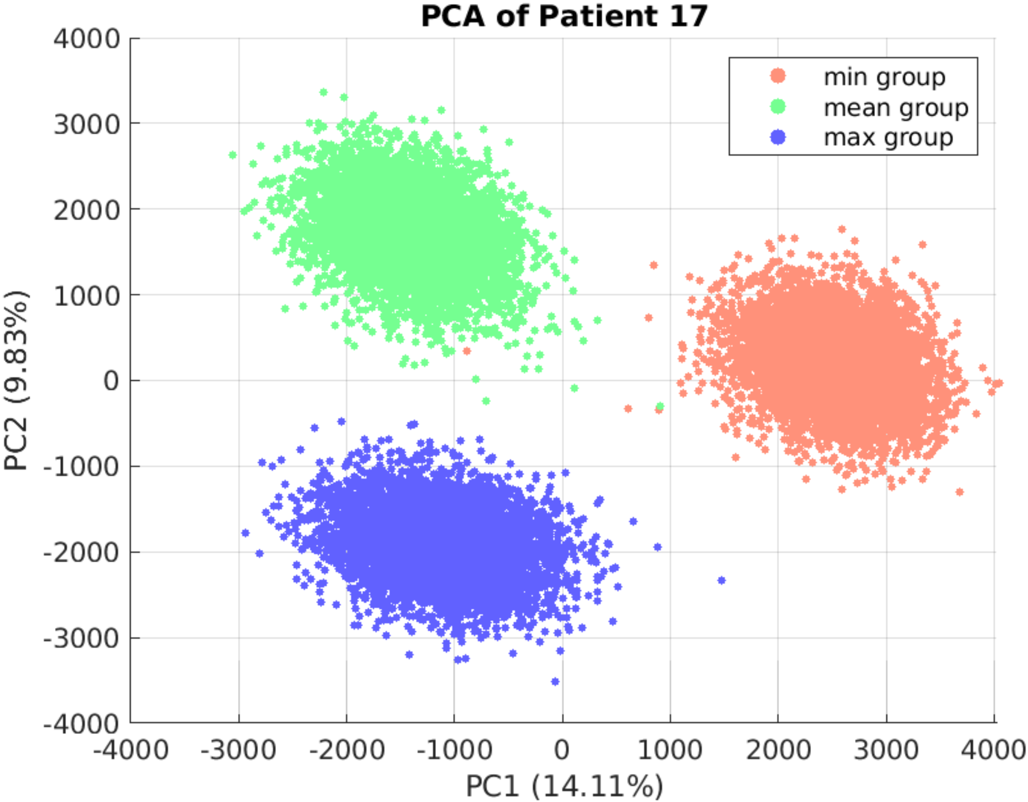
PCA of Patient 17
min group
mean group
max group
PC1 (14.11%)
PC2 (9.83%)
4000
3000
2000
1000
0
-1000
-2000
-3000
-4000

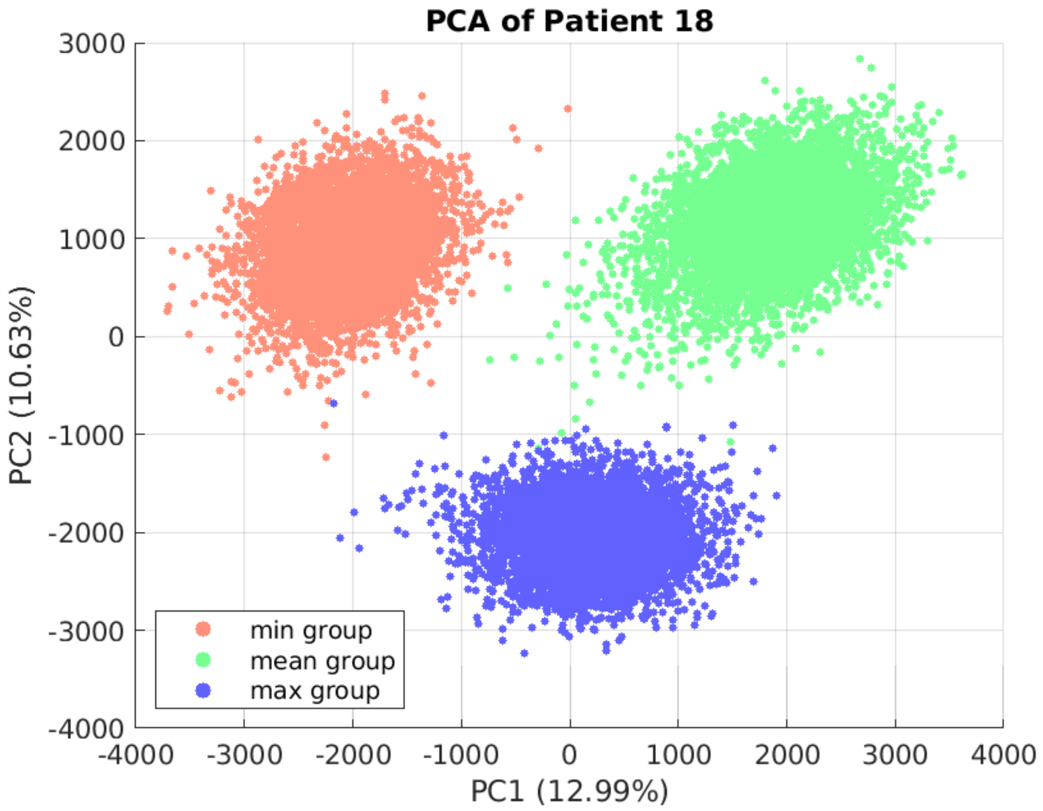

PCA of Patient 18
3000
2000
1000
0
-1000
-2000
-3000
-4000
-4000
-3000
-2000
-1000
0
1000
2000
3000
4000
PC1 (12.99%)
PC2 (10.63%)
min group
mean group
max group

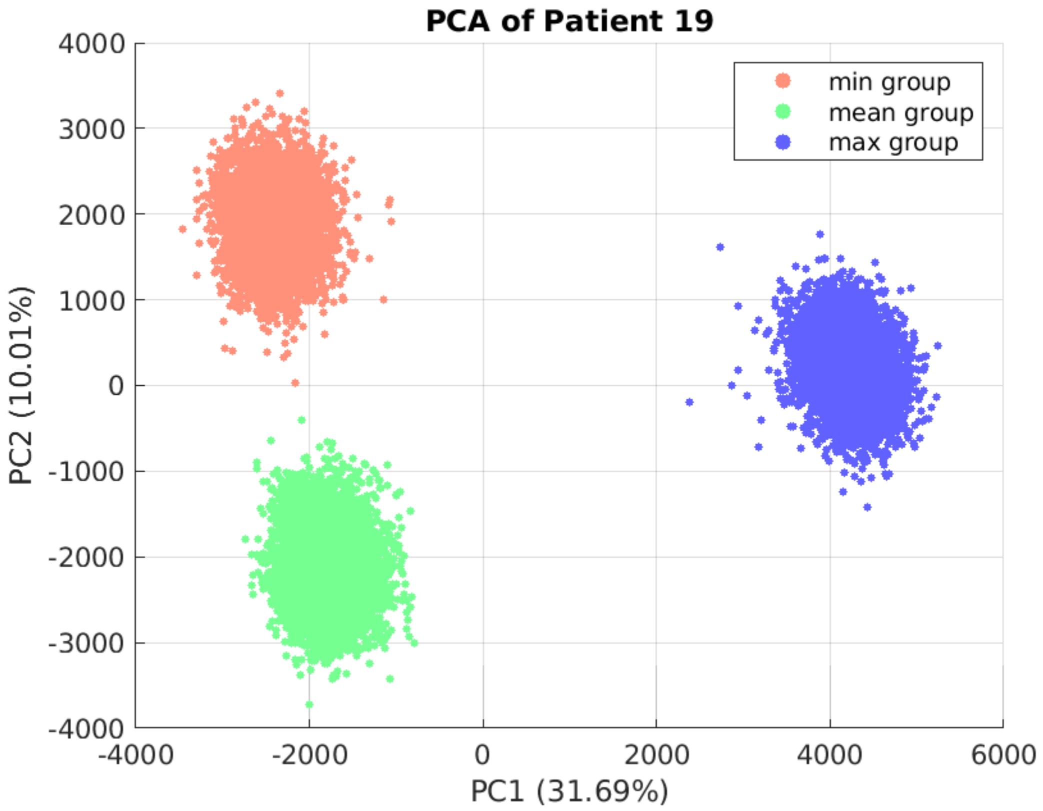

PCA of Patient 19
min group
mean group
max group
4000
3000
2000
1000
0
-1000
-2000
-3000
-4000
-4000
-2000
0
2000
4000
6000
PC1 (31.69%)
PC2 (10.01%)

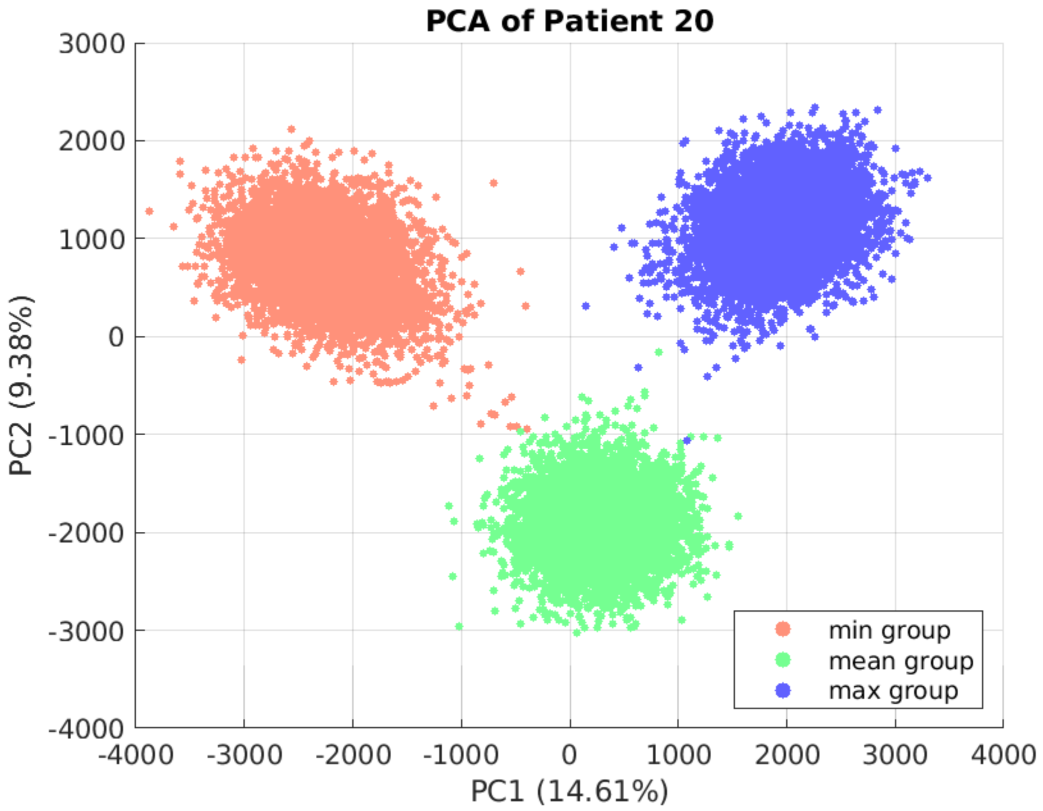

PCA of Patient 20
3000
2000
1000
0
-1000
-2000
-3000
-4000
-4000
-3000
-2000
-1000
0
1000
2000
3000
4000
PC2 (9.38%)
PC1 (14.61%)
min group
mean group
max group

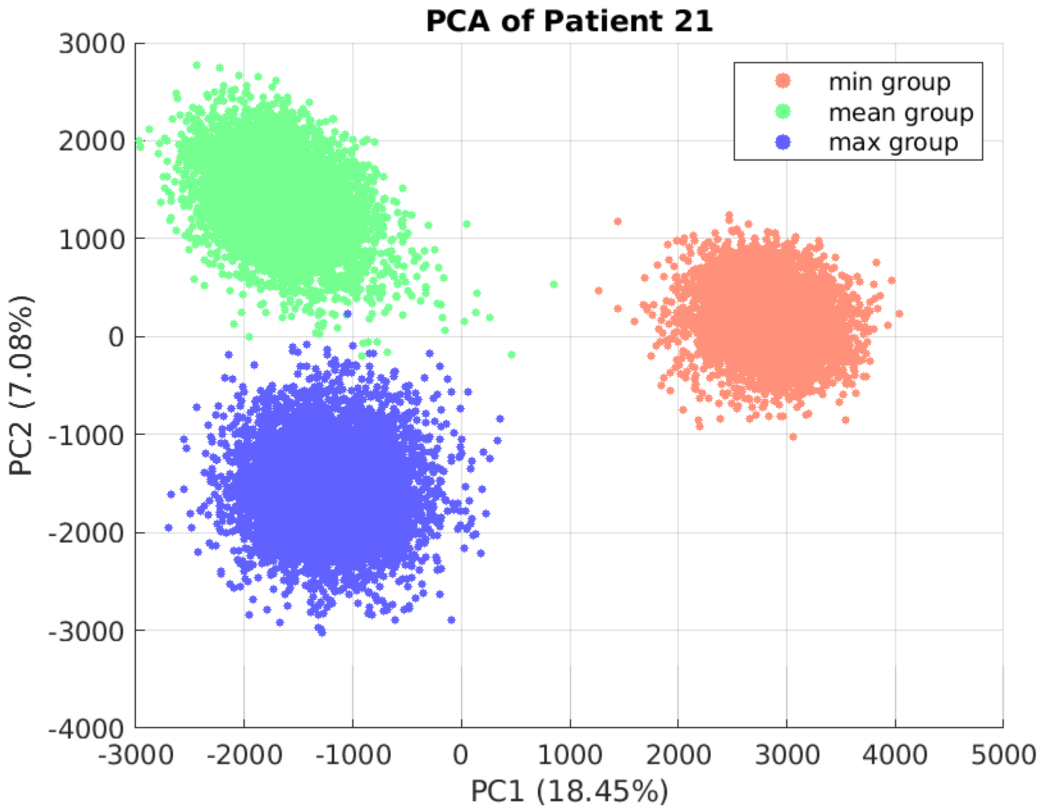

PCA of Patient 21
min group
mean group
max group
PC1 (18.45%)
PC2 (7.08%)
3000
2000
1000
0
-1000
-2000
-3000
-4000
-3000
-2000
-1000
0
1000
2000
3000
4000
5000

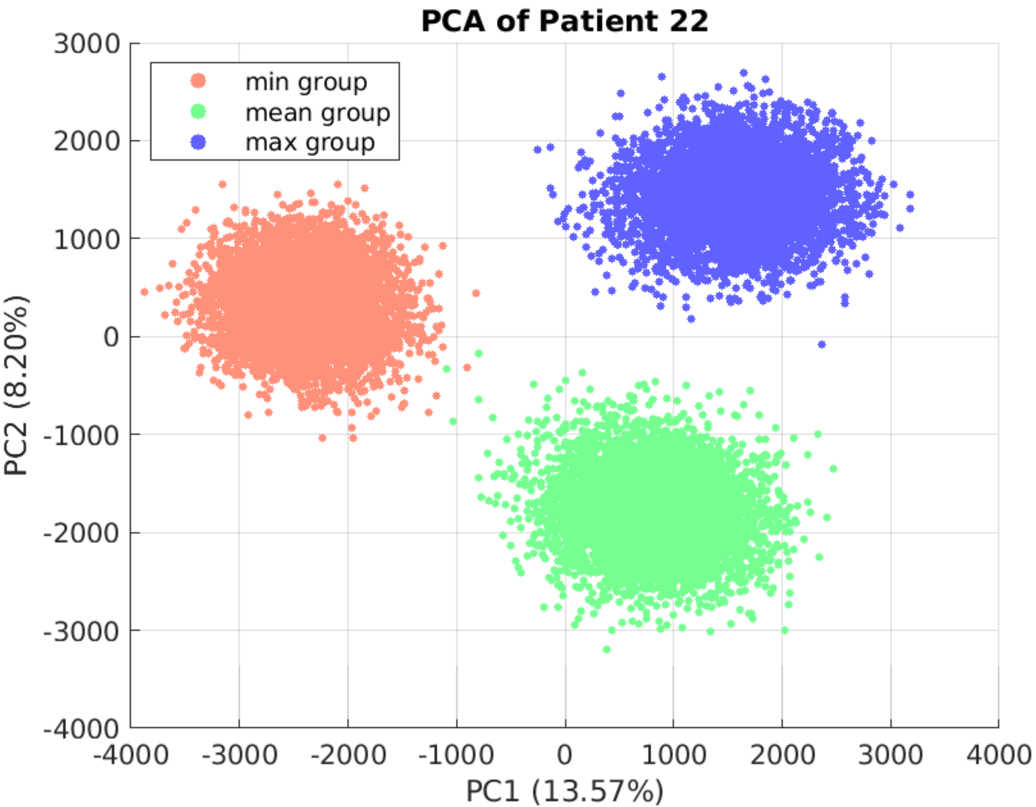
PCA of Patient 22
min group
mean group
max group
PC2 (8.20%)
PC1 (13.57%)
3000
2000
1000
0
-1000
-2000
-3000
-4000
-4000
-3000
-2000
-1000
0
1000
2000
3000
4000

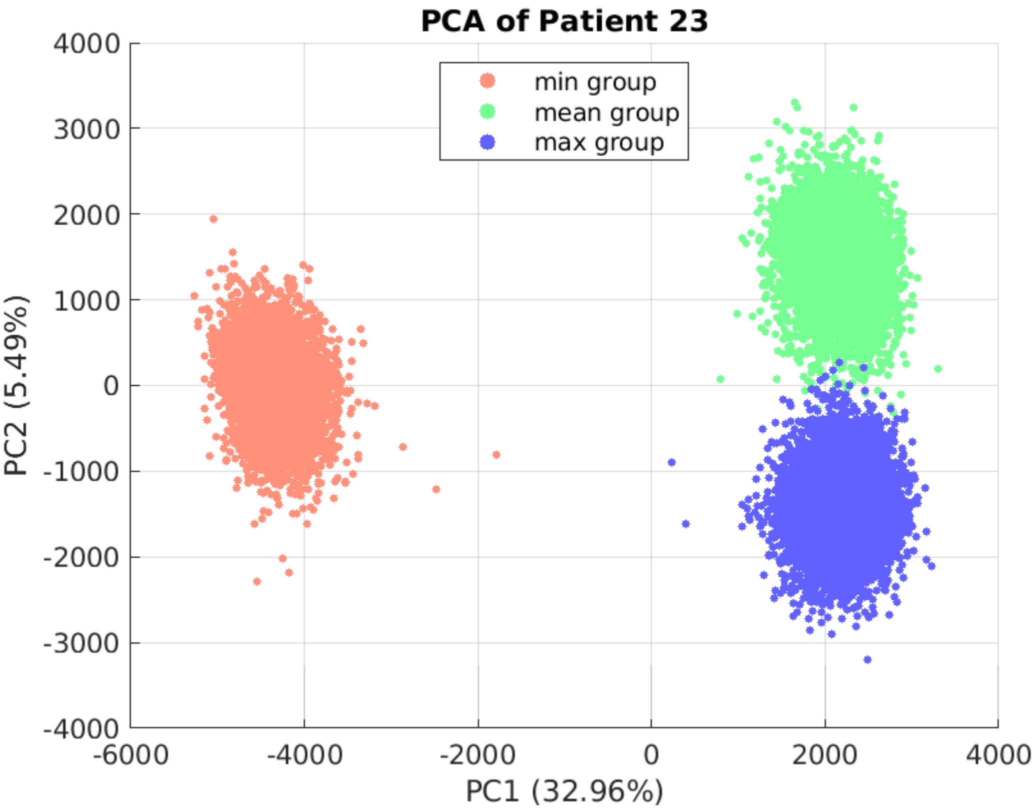

PCA of Patient 23
min group
mean group
max group
PC2 (5.49%)
PC1 (32.96%)
4000
3000
2000
1000
0
-1000
-2000
-3000
-4000
-6000
-4000
-2000
0
2000
4000

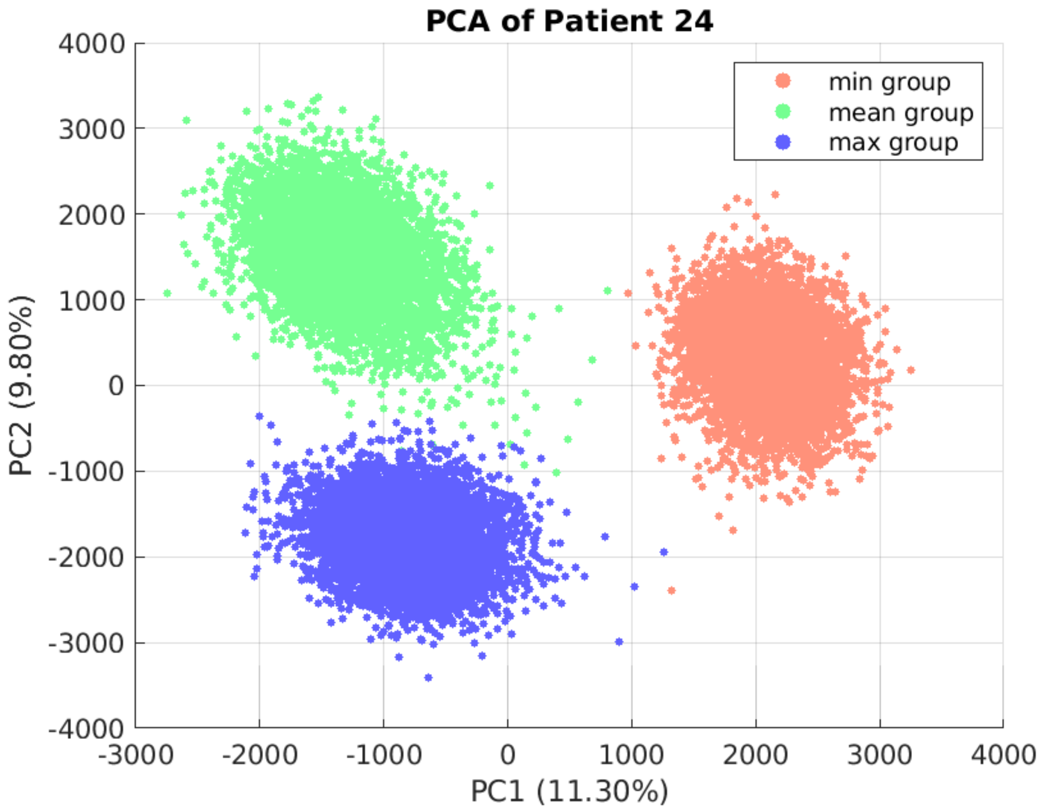
PCA of Patient 24
min group
mean group
max group
PC2 (9.80%)
PC1 (11.30%)
4000
3000
2000
1000
0
-1000
-2000
-3000
-4000
-3000
-2000
-1000
0
1000
2000
3000
4000

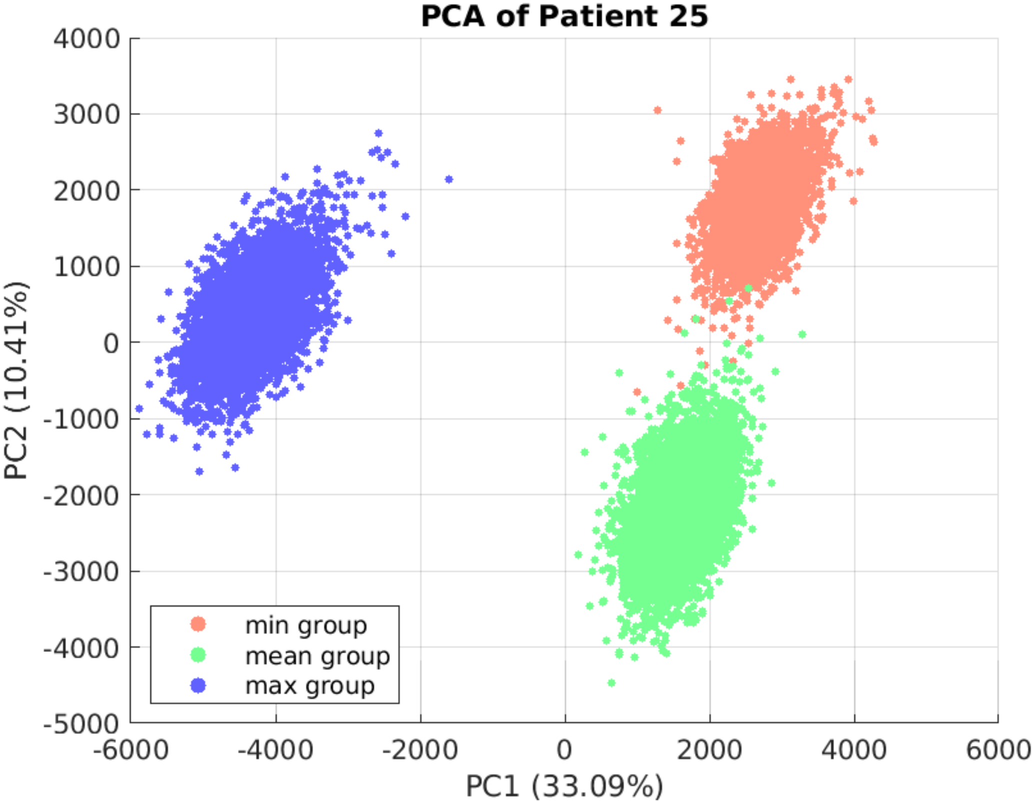

PCA of Patient 25
4000
3000
2000
1000
0
-1000
-2000
-3000
-4000
-5000
-6000
-4000
-2000
0
2000
4000
6000
PC1 (33.09%)
PC2 (10.41%)
min group
mean group
max group

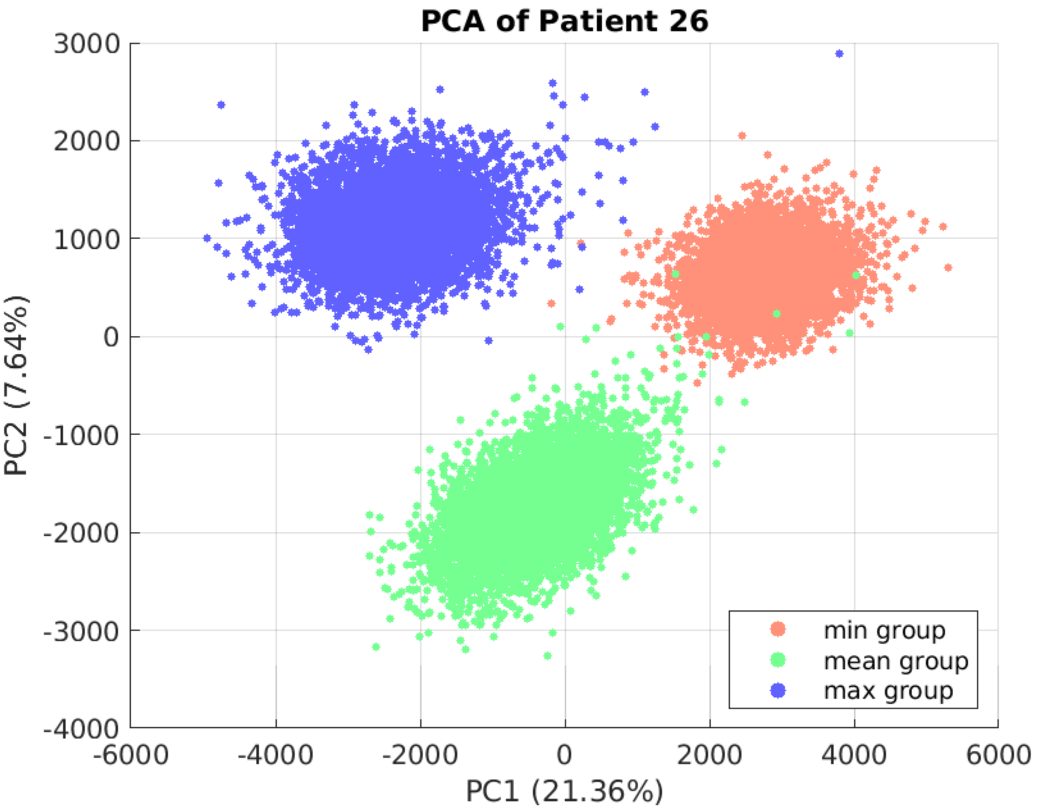

PCA of Patient 26
3000
2000
1000
0
-1000
-2000
-3000
-4000
-6000
-4000
-2000
0
2000
4000
6000
PC1 (21.36%)
PC2 (7.64%)
min group
mean group
max group

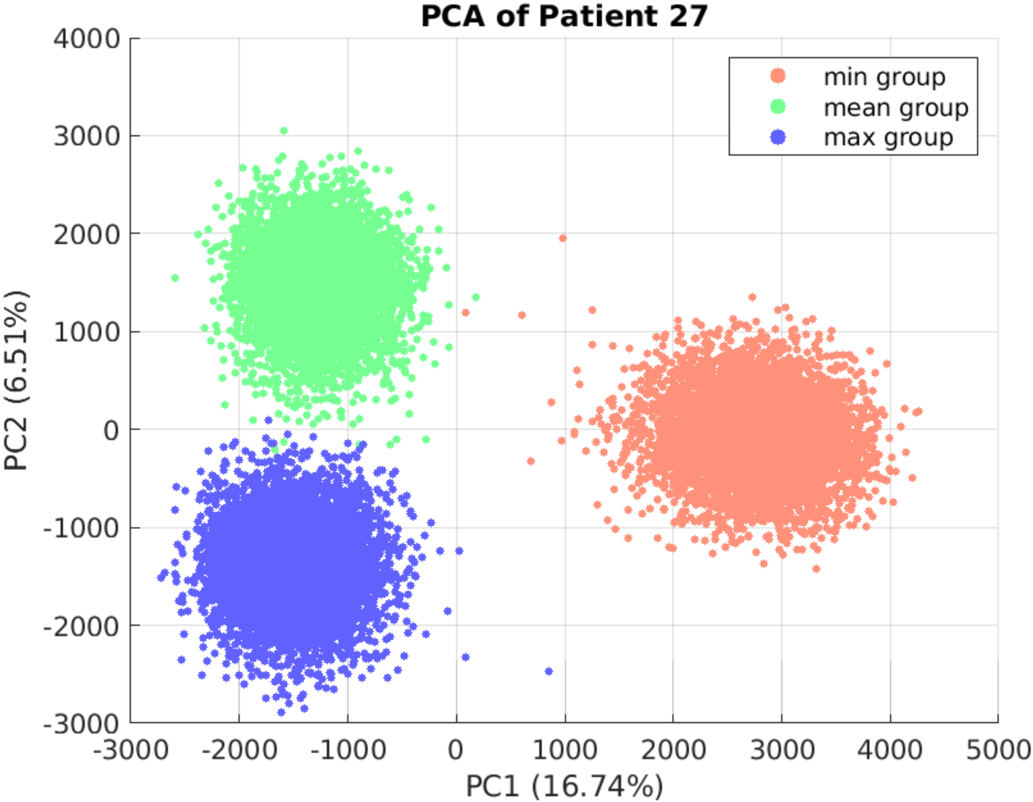
PCA of Patient 27
min group
mean group
max group
PC1 (16.74%)
PC2 (6.51%)
4000
3000
2000
1000
0
-1000
-2000
-3000
-3000
-2000
-1000
0
1000
2000
3000
4000
5000

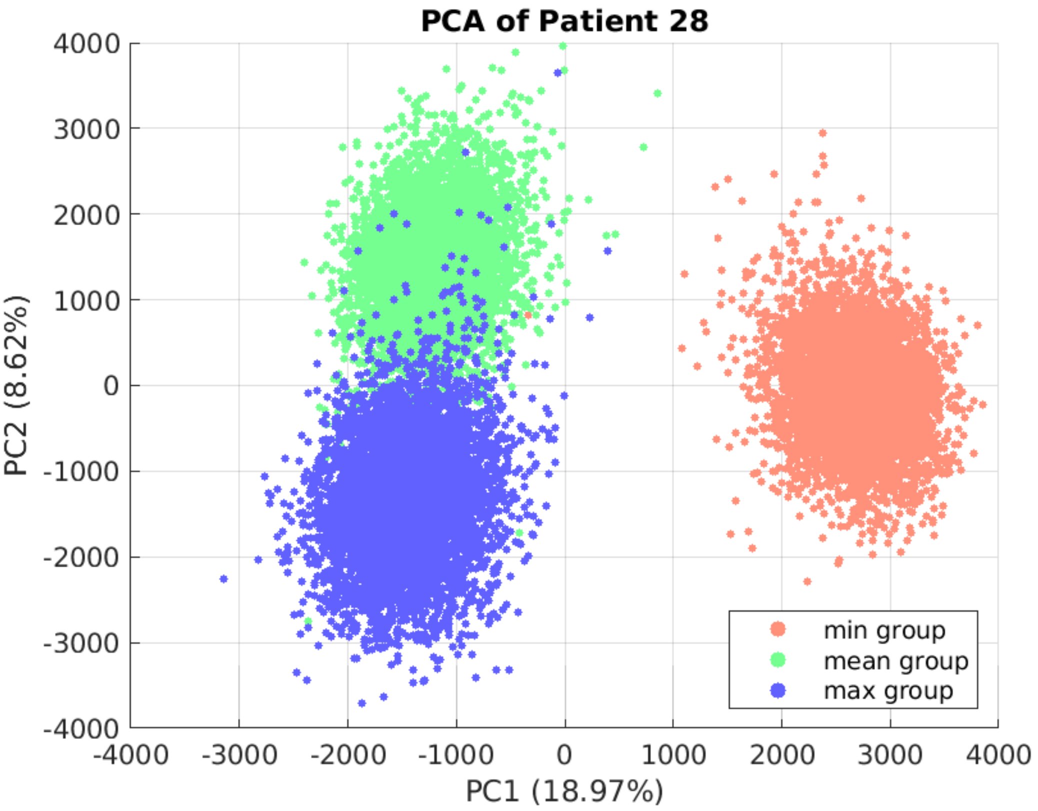

PCA of Patient 28
PC2 (8.62%)
PC1 (18.97%)
4000
3000
2000
1000
0
-1000
-2000
-3000
-4000
-4000
-3000
-2000
-1000
0
1000
2000
3000
4000
min group
mean group
max group

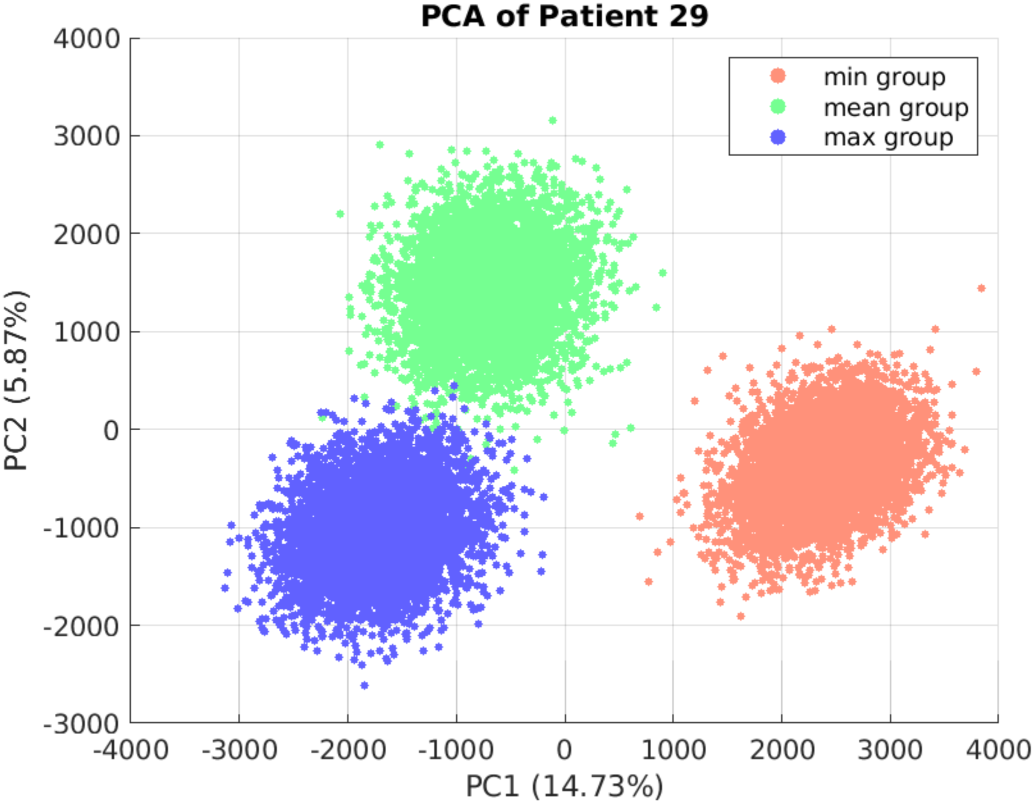
PCA of Patient 29
min group
mean group
max group
PC1 (14.73%)
PC2 (5.87%)
4000
3000
2000
1000
0
-1000
-2000
-3000
-4000

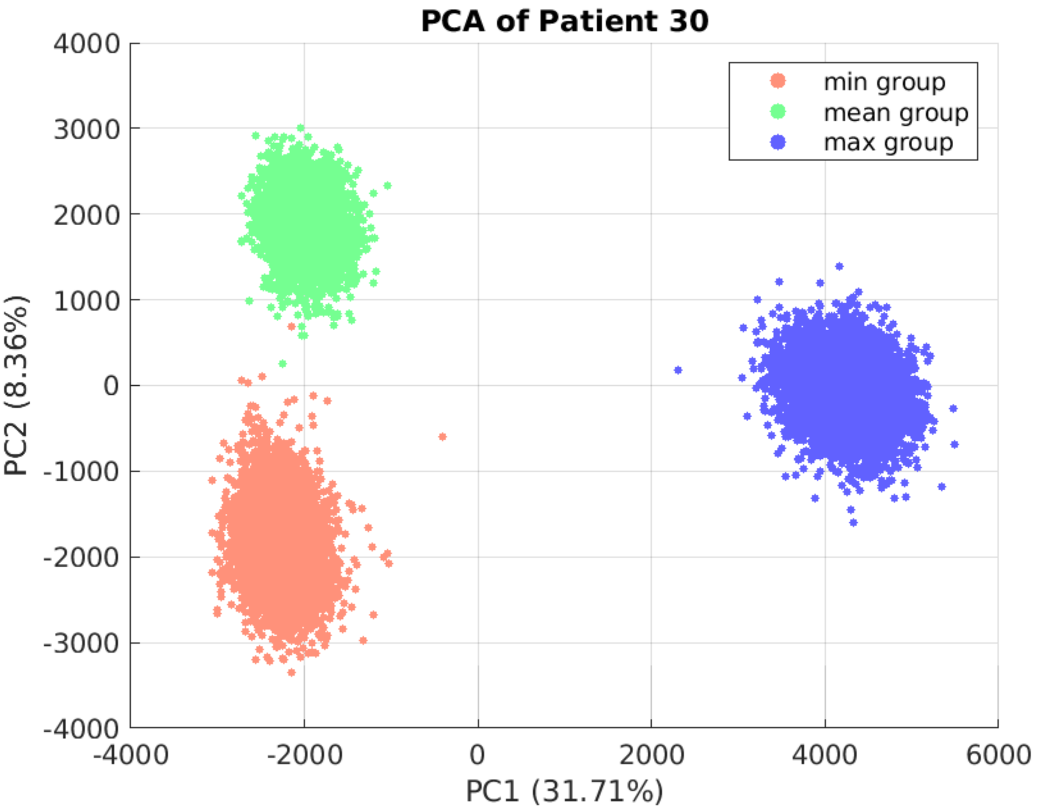
PCA of Patient 30
min group
mean group
max group
PC2 (8.36%)
PC1 (31.71%)
4000
3000
2000
1000
0
-1000
-2000
-3000
-4000
-4000
-2000
0
2000
4000
6000

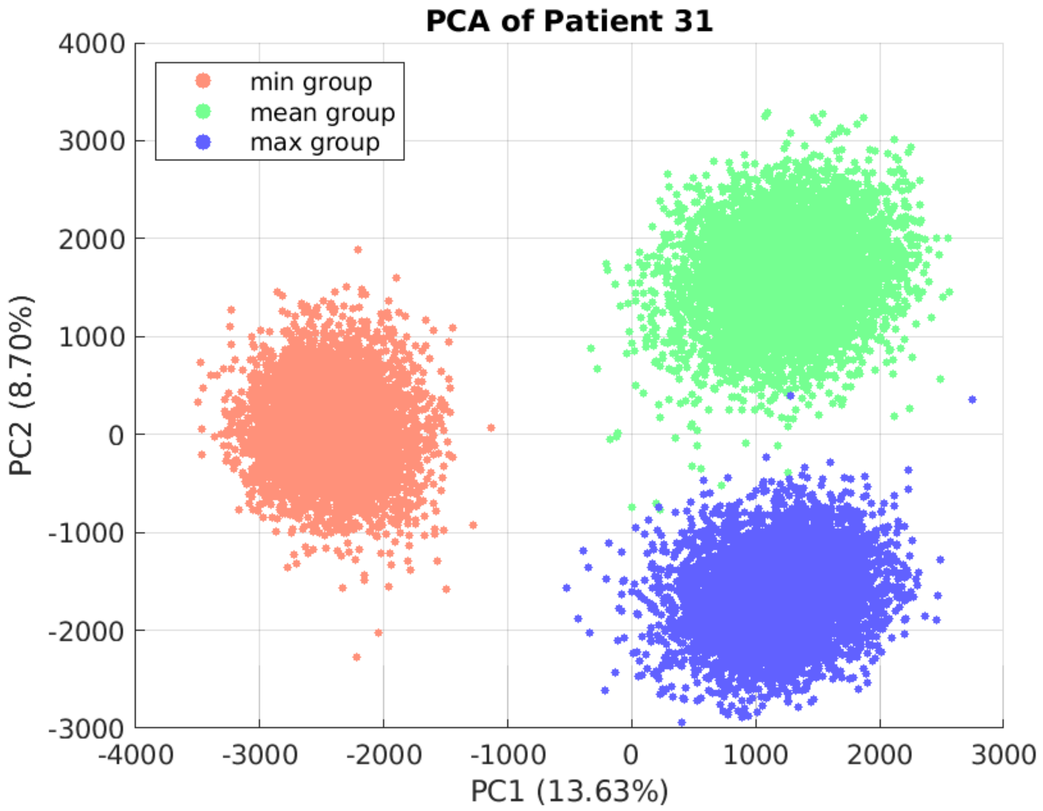
PCA of Patient 31
min group
mean group
max group
PC1 (13.63%)
PC2 (8.70%)
-4000
-3000
-2000
-1000
0
1000
2000
3000
4000

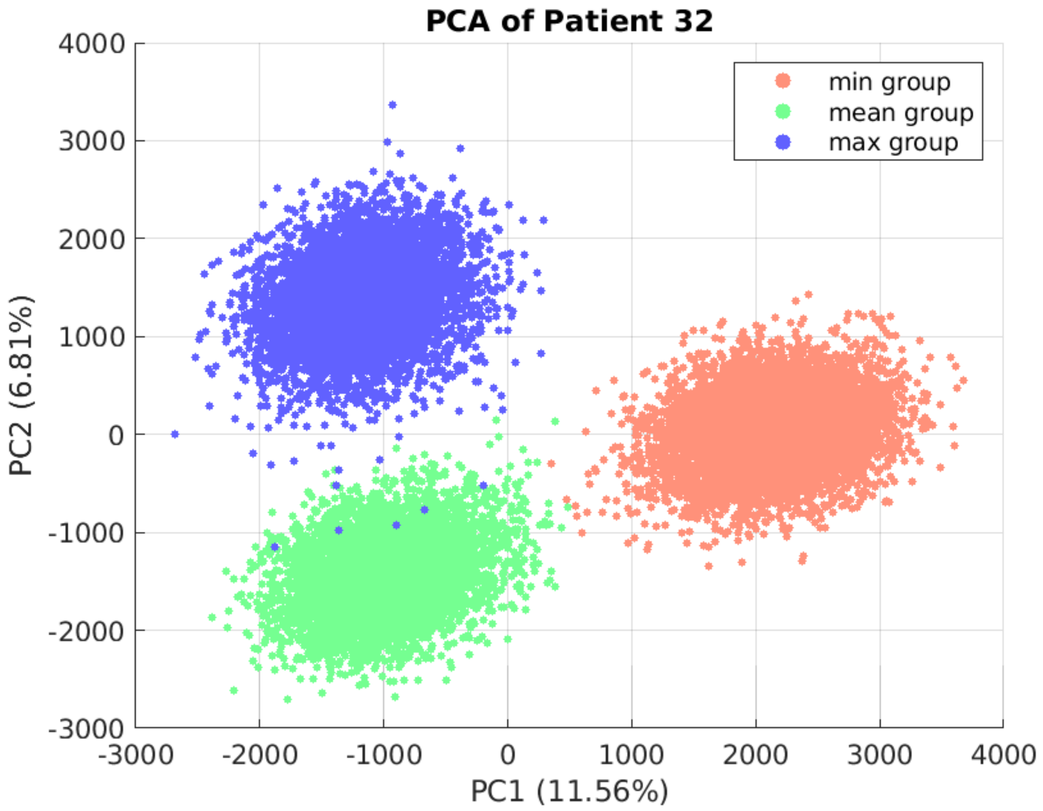
PCA of Patient 32
min group
mean group
max group
4000
3000
2000
1000
0
-1000
-2000
-3000
-3000
-2000
-1000
0
1000
2000
3000
4000
PC1 (11.56%)
PC2 (6.81%)

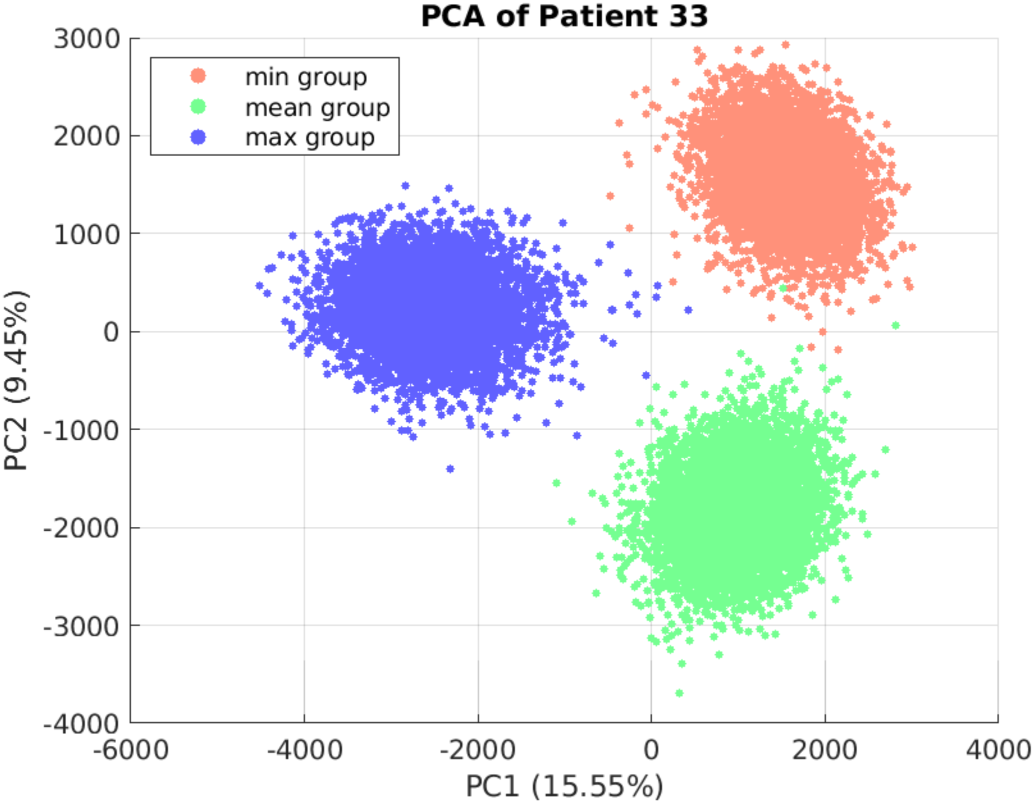
PCA of Patient 33
min group
mean group
max group
PC2 (9.45%)
PC1 (15.55%)
3000
2000
1000
0
-1000
-2000
-3000
-4000
-6000
-4000
-2000
0
2000
4000

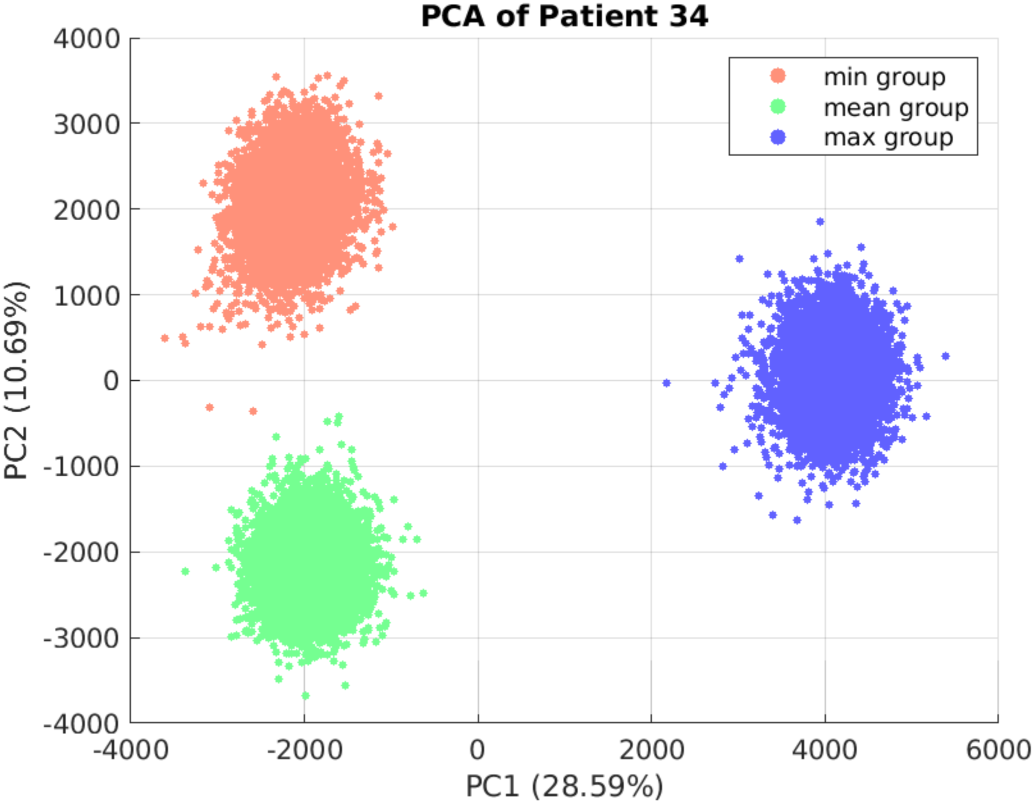

PCA of Patient 34
min group
mean group
max group
PC2 (10.69%)
PC1 (28.59%)
4000
3000
2000
1000
0
-1000
-2000
-3000
-4000
-4000
-2000
0
2000
4000
6000

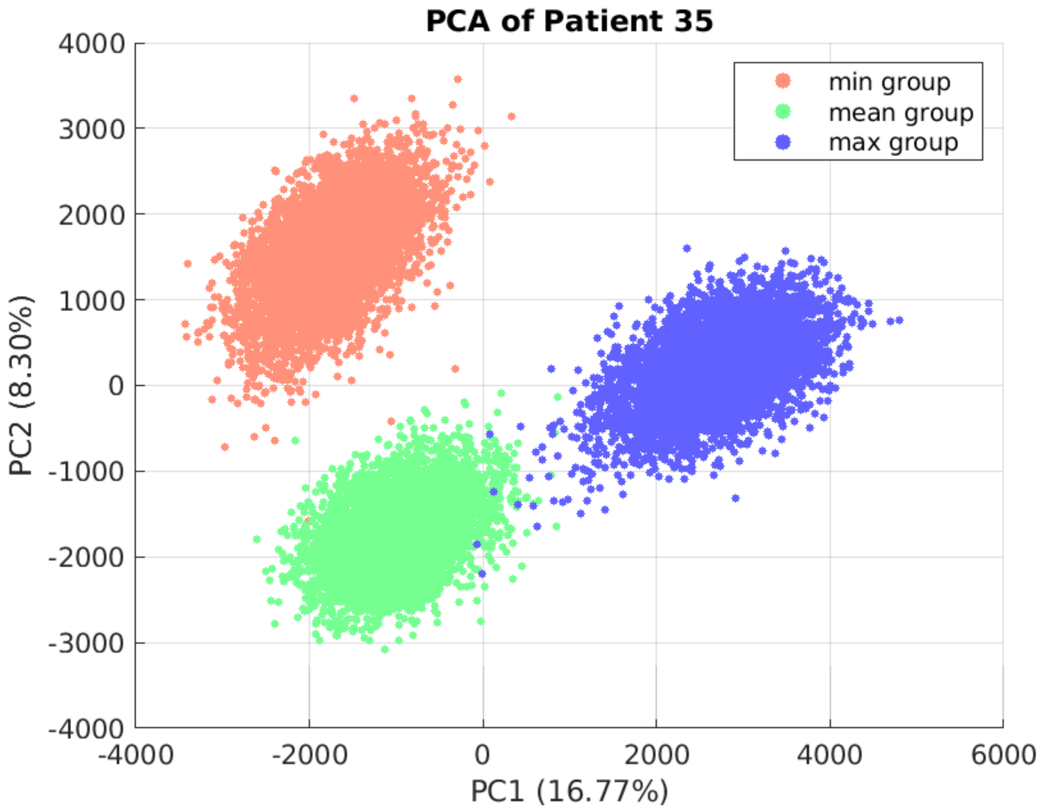
PCA of Patient 35
min group
mean group
max group
PC2 (8.30%)
PC1 (16.77%)
4000
3000
2000
1000
0
-1000
-2000
-3000
-4000
-4000
-2000
0
2000
4000
6000

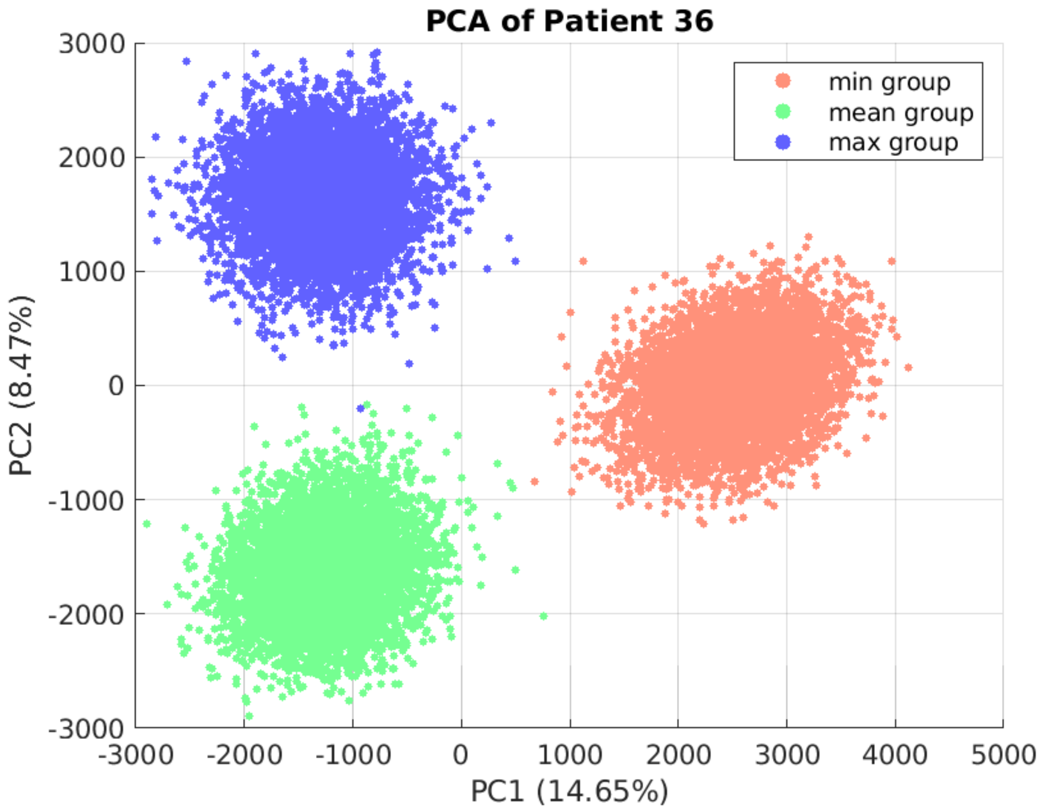

PCA of Patient 36
min group
mean group
max group
PC2 (8.47%)
PC1 (14.65%)
3000
2000
1000
0
-1000
-2000
-3000
-3000
-2000
-1000
0
1000
2000
3000
4000
5000

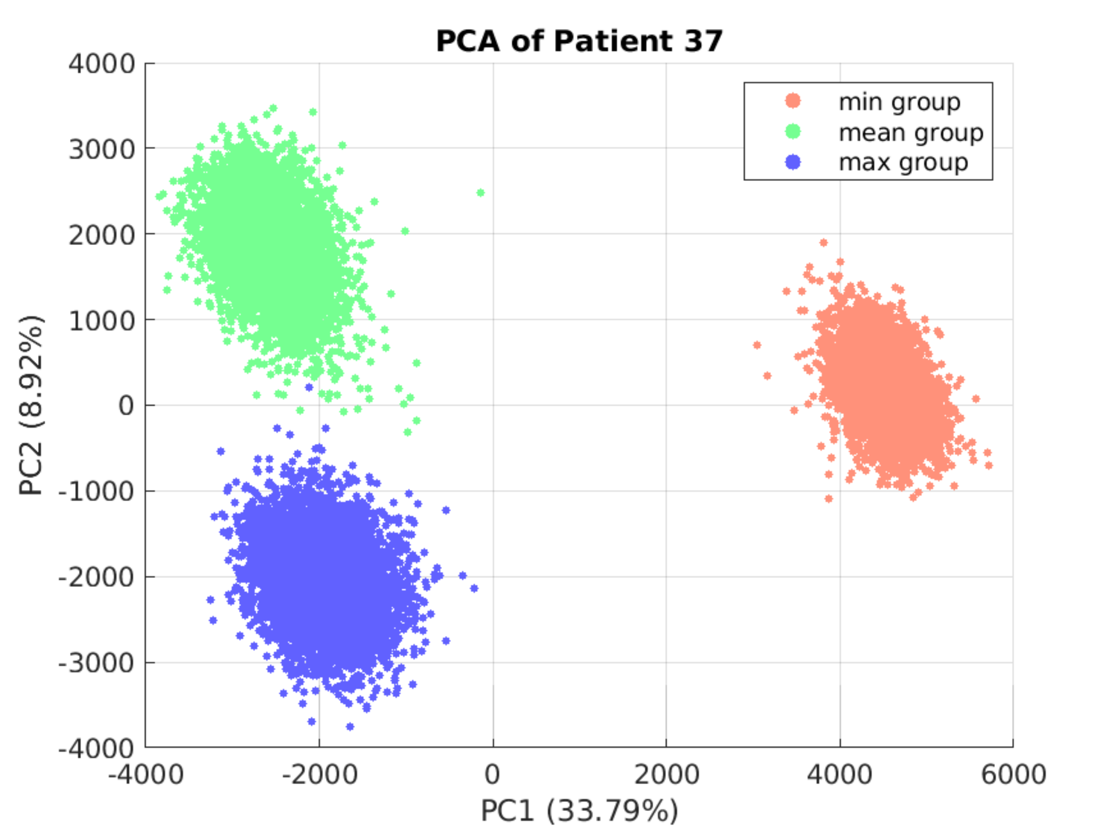

PCA of Patient 37
min group
mean group
max group
PC1 (33.79%)
PC2 (8.92%)
4000
3000
2000
1000
0
-1000
-2000
-3000
-4000
-4000
-2000
0
2000
4000
6000

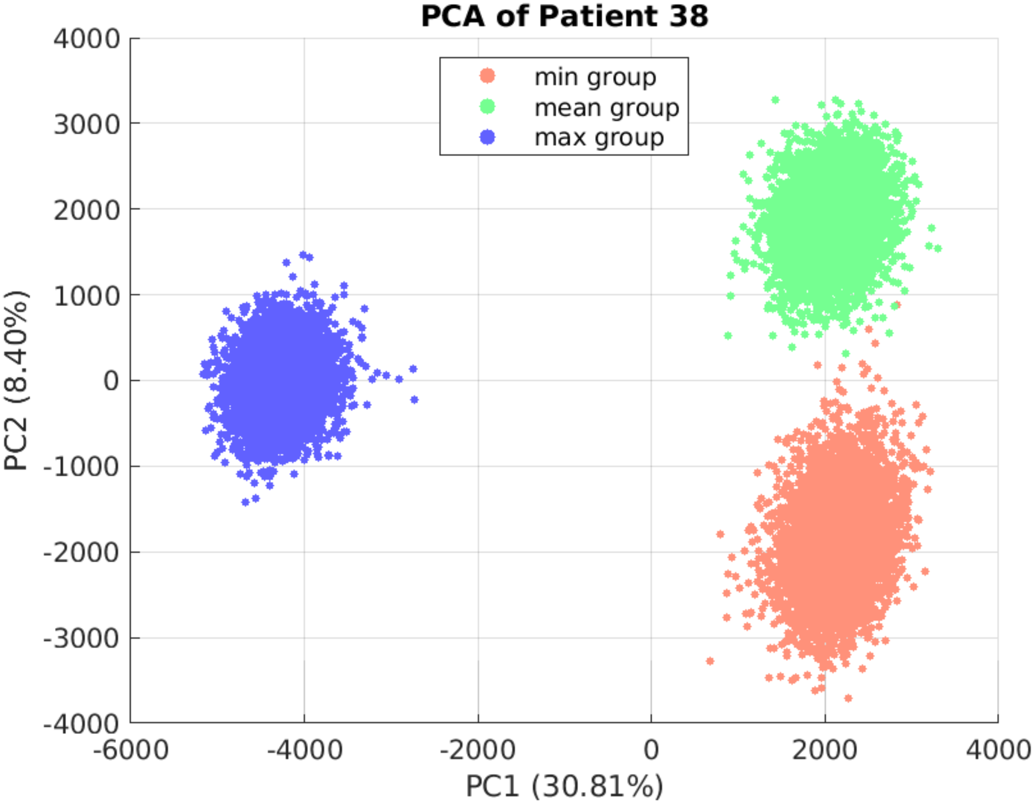
PCA of Patient 38
min group
mean group
max group
PC1 (30.81%)
PC2 (8.40%)
-6000
-4000
-2000
0
2000
4000
-4000
-3000
-2000
-1000
1000
2000
3000

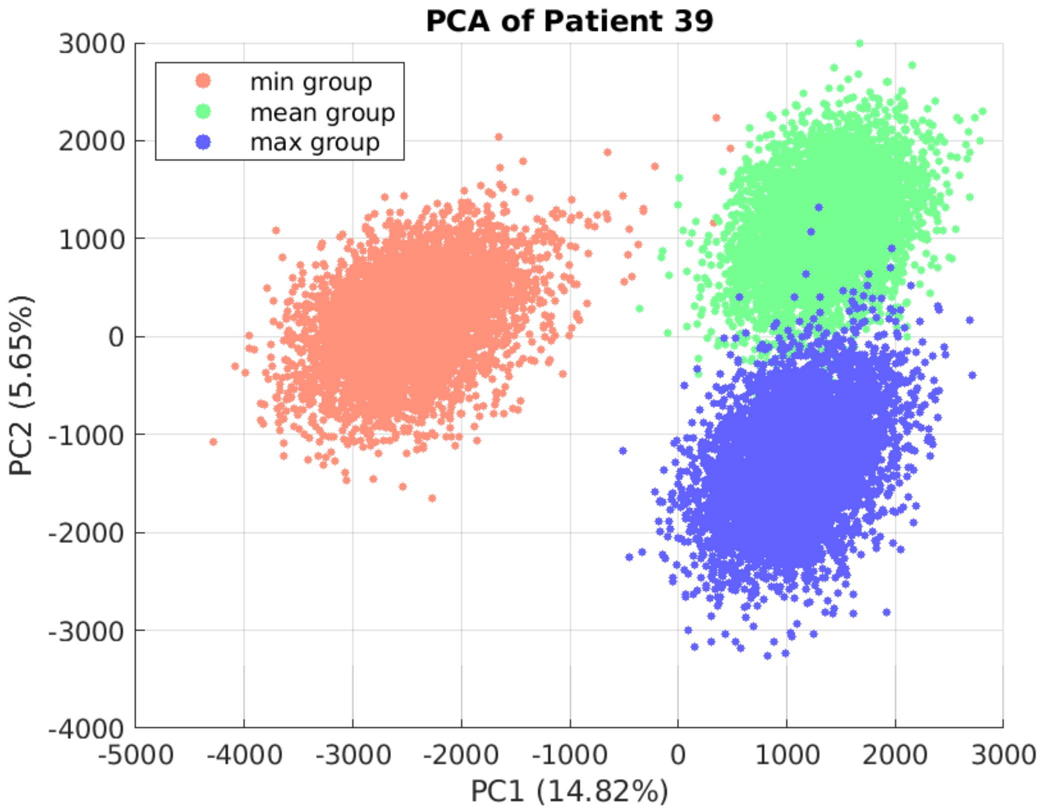
PCA of Patient 39
min group
mean group
max group
PC1 (14.82%)
PC2 (5.65%)
3000
2000
1000
0
-1000
-2000
-3000
-4000
-5000
-4000
-3000
-2000
-1000
0
1000
2000
3000

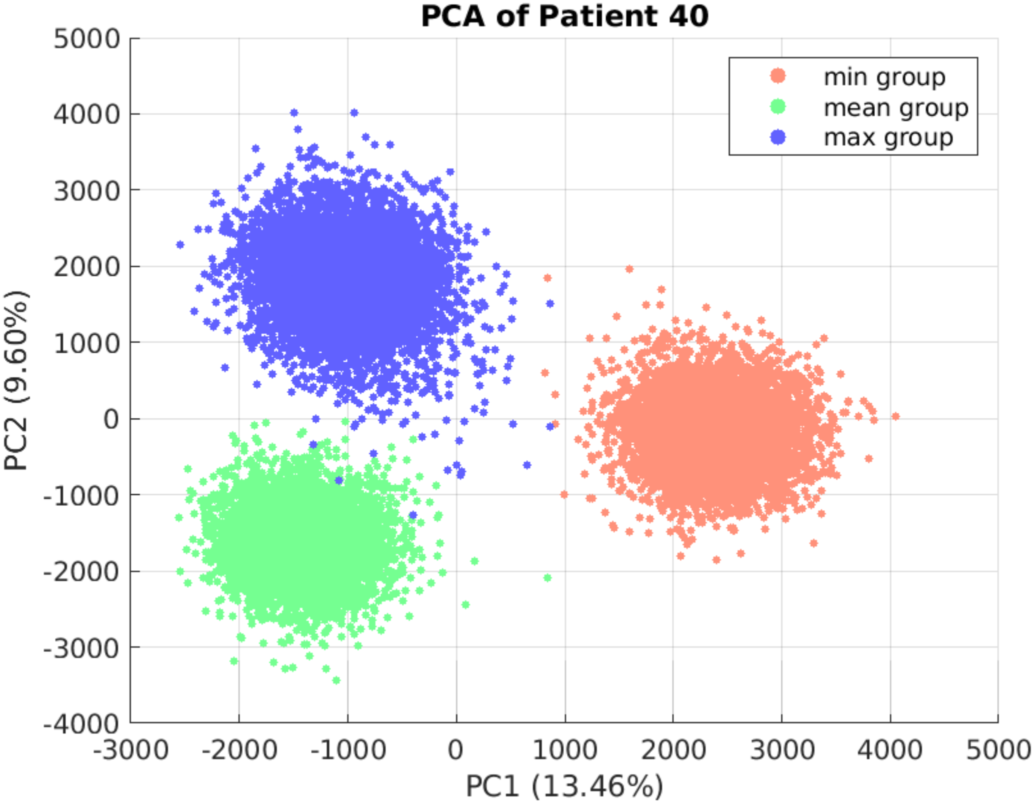

PCA of Patient 40
min group
mean group
max group
PC2 (9.60%)
PC1 (13.46%)
5000
4000
3000
2000
1000
0
-1000
-2000
-3000
-4000
-3000
-2000
-1000
0
1000
2000
3000
4000
5000

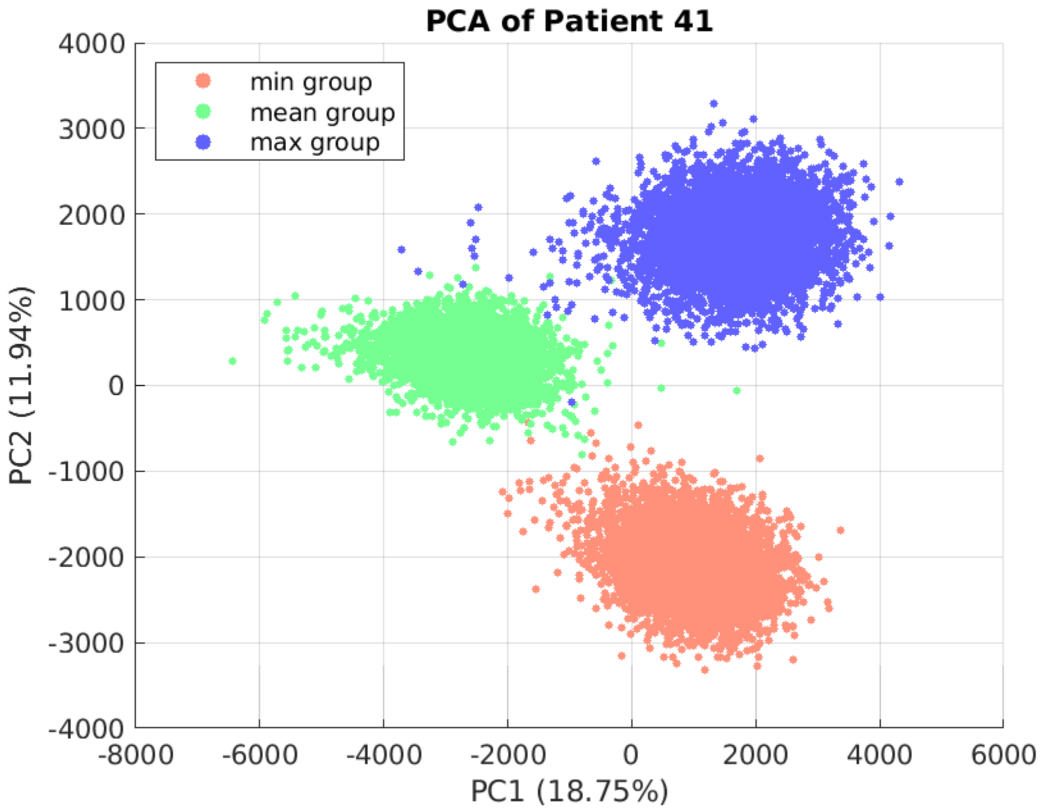
PCA of Patient 41
min group
mean group
max group
PC1 (18.75%)
PC2 (11.94%)
4000
3000
2000
1000
0
-1000
-2000
-3000
-4000
-8000
-6000
-4000
-2000
0
2000
4000
6000

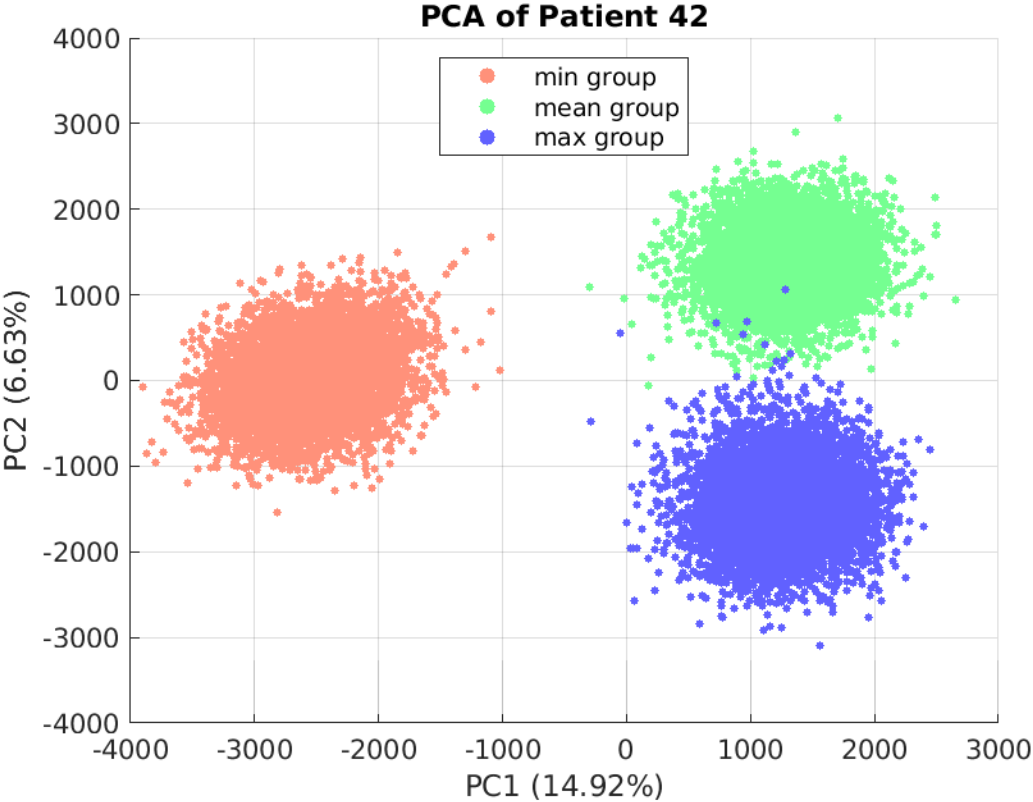
PCA of Patient 42
min group
mean group
max group
PC1 (14.92%)
PC2 (6.63%)
4000
3000
2000
1000
0
-1000
-2000
-3000
-4000
-4000
-3000
-2000
-1000
0
1000
2000
3000

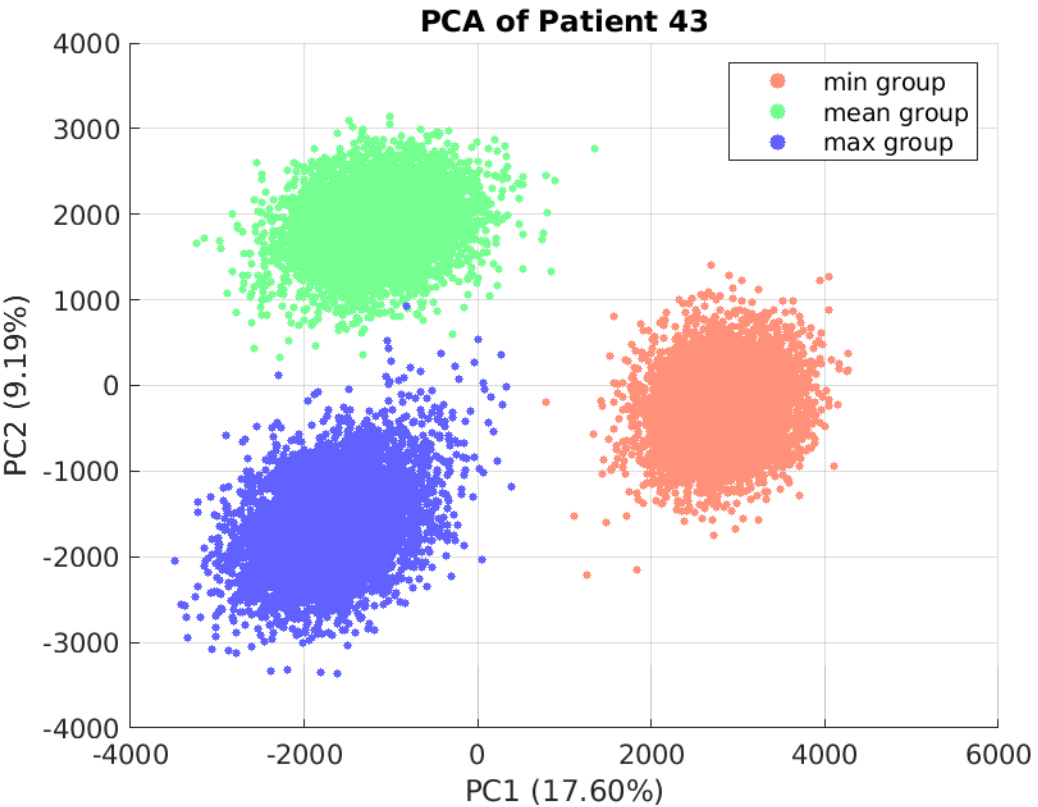
PCA of Patient 43
min group
mean group
max group
PC1 (17.60%)
PC2 (9.19%)
4000
3000
2000
1000
0
-1000
-2000
-3000
-4000
-4000
-2000
0
2000
4000
6000

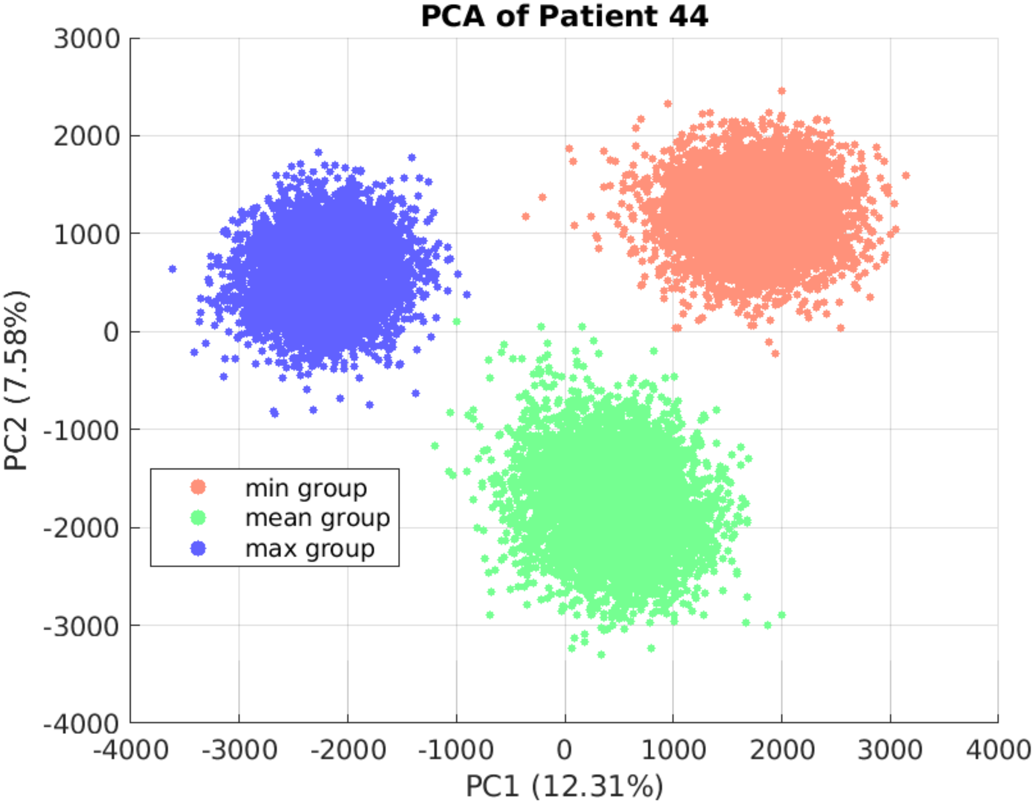
PCA of Patient 44
PC2 (7.58%)
PC1 (12.31%)
min group
mean group
max group
3000
2000
1000
0
-1000
-2000
-3000
-4000
-4000
-3000
-2000
-1000
0
1000
2000
3000
4000

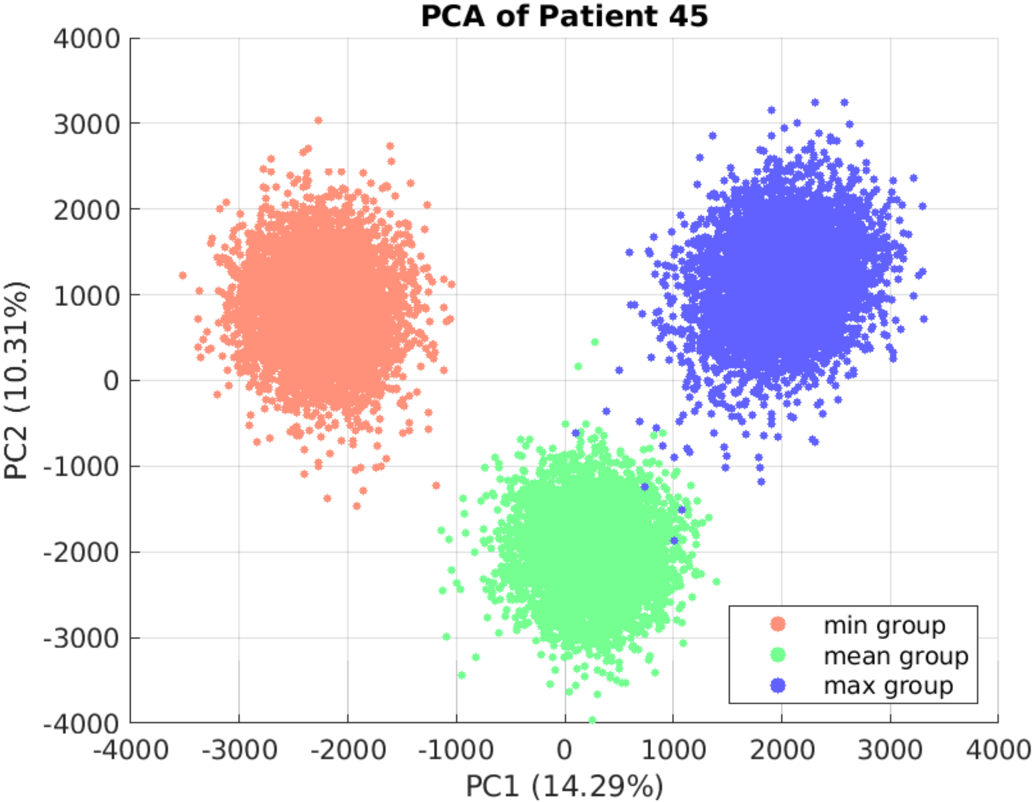

PCA of Patient 45
4000
3000
2000
1000
0
-1000
-2000
-3000
-4000
-4000
-3000
-2000
-1000
0
1000
2000
3000
4000
PC1 (14.29%)
PC2 (10.31%)
min group
mean group
max group

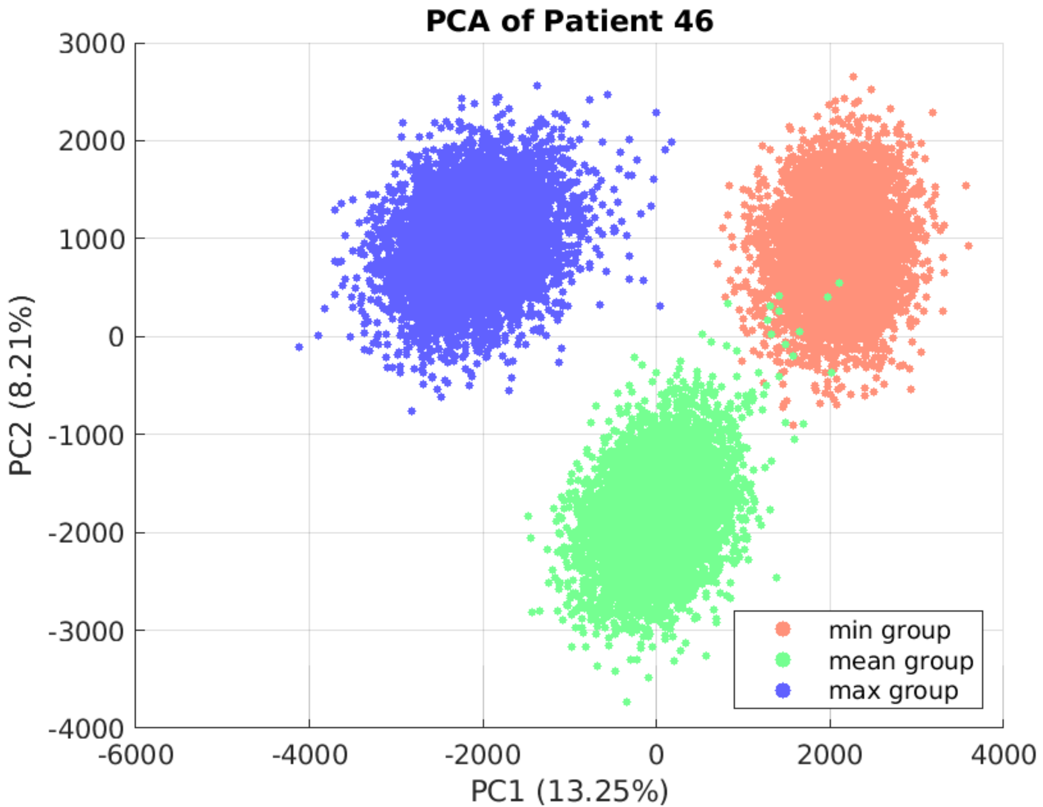
PCA of Patient 46
PC1 (13.25%)
PC2 (8.21%)
-6000
-4000
-2000
0
2000
4000
3000
2000
1000
0
-1000
-2000
-3000
-4000
min group
mean group
max group

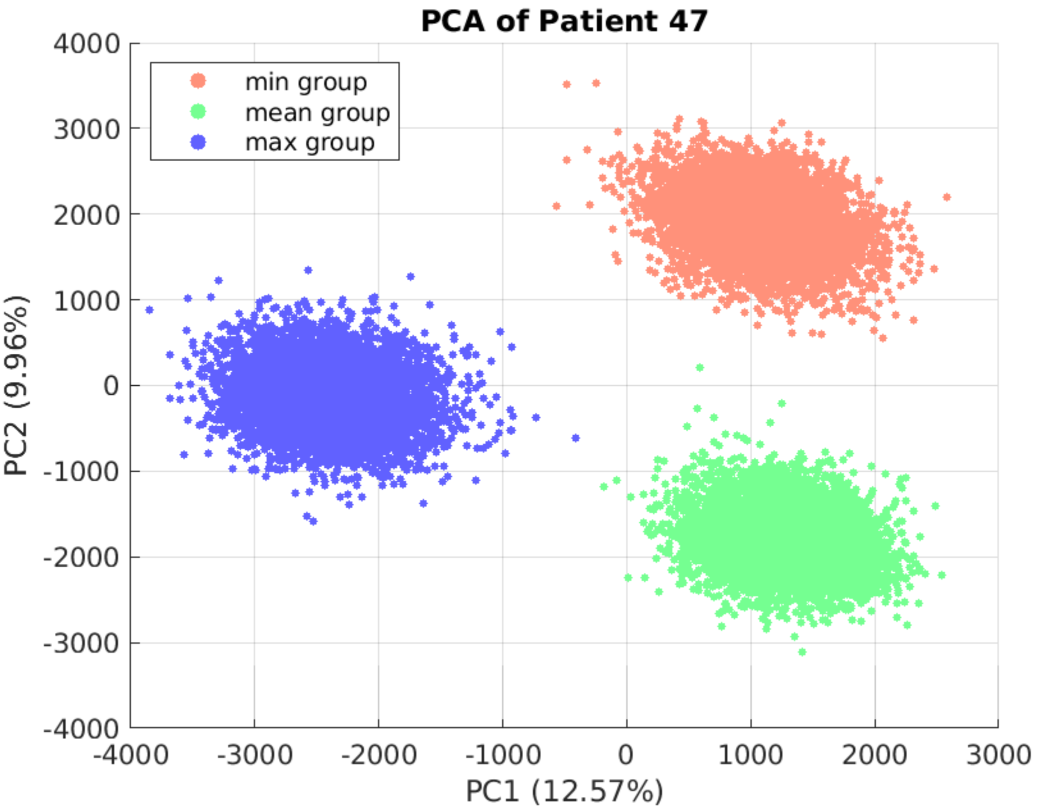

PCA of Patient 47
min group
mean group
max group
PC2 (9.96%)
PC1 (12.57%)
4000
3000
2000
1000
0
-1000
-2000
-3000
-4000
-4000
-3000
-2000
-1000
0
1000
2000
3000

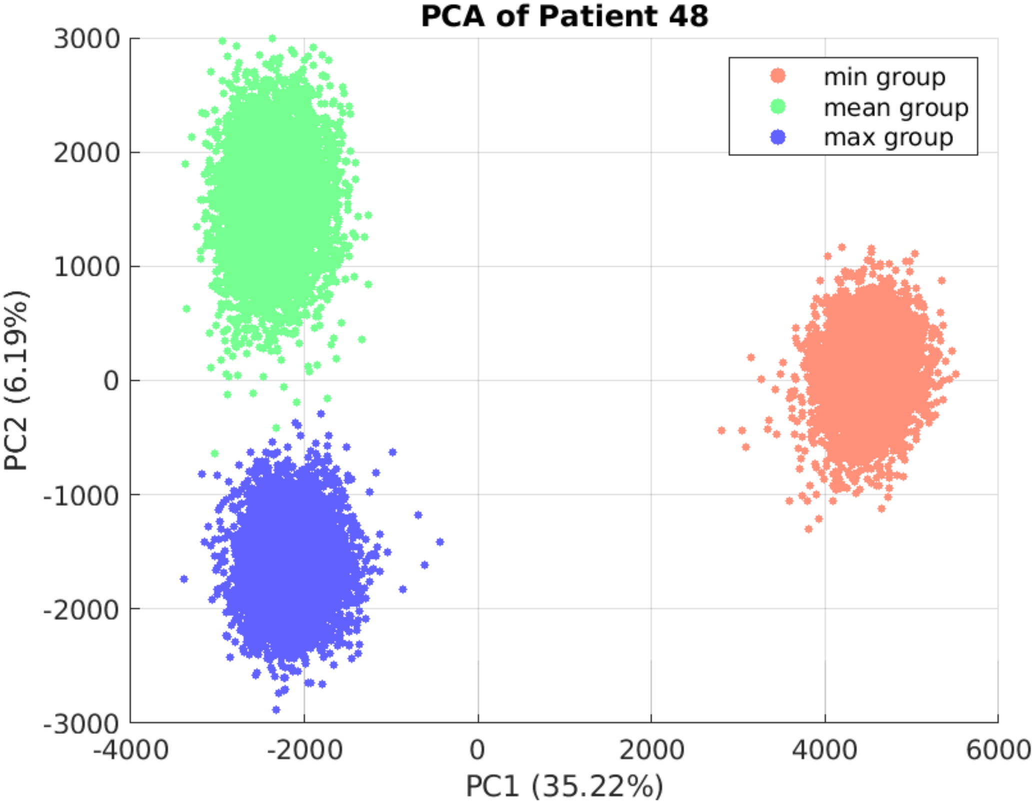
PCA of Patient 48
min group
mean group
max group
PC2 (6.19%)
PC1 (35.22%)
3000
2000
1000
0
-1000
-2000
-3000
-4000
-2000
0
2000
4000
6000

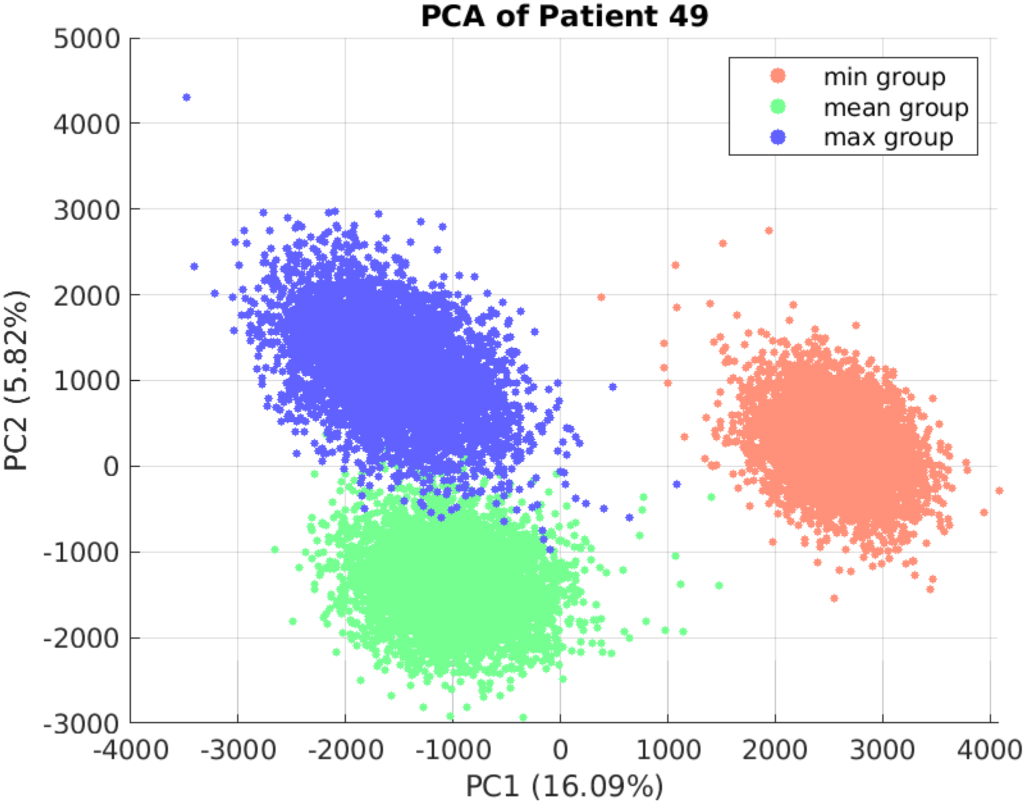
PCA of Patient 49
min group
mean group
max group
PC1 (16.09%)
PC2 (5.82%)
-4000
-3000
-2000
-1000
0
1000
2000
3000
4000
5000

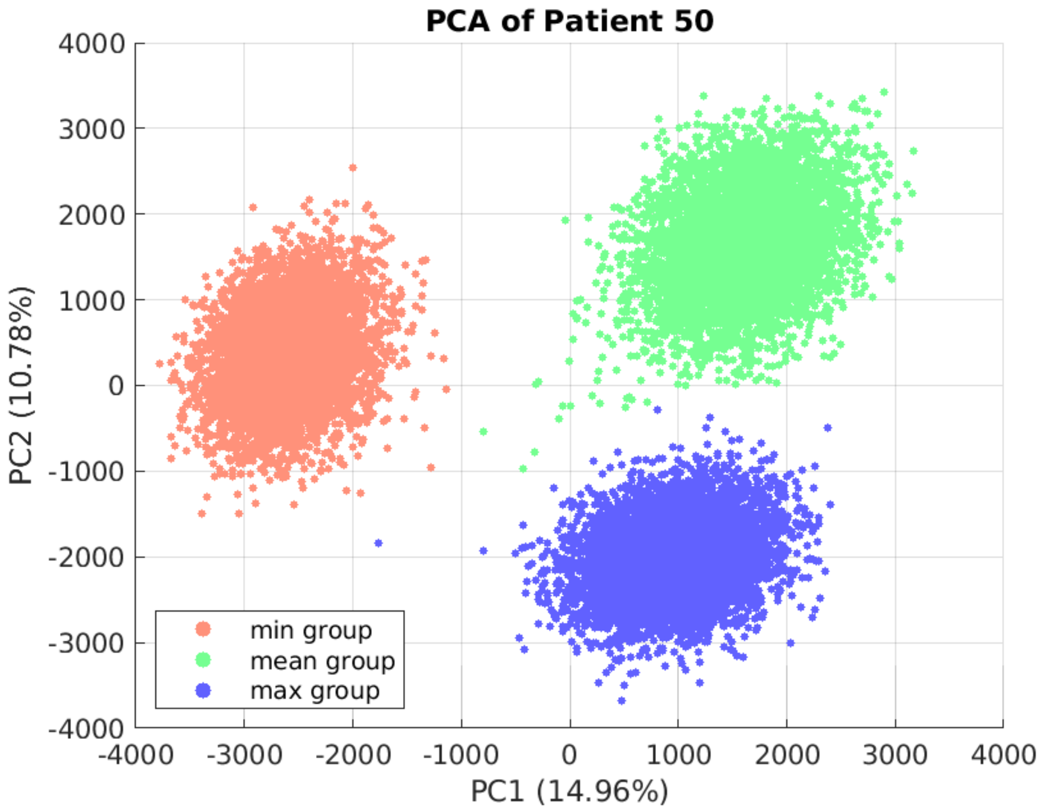

PCA of Patient 50
PC2 (10.78%)
PC1 (14.96%)
4000
3000
2000
1000
0
-1000
-2000
-3000
-4000
-4000
-3000
-2000
-1000
0
1000
2000
3000
4000
min group
mean group
max group

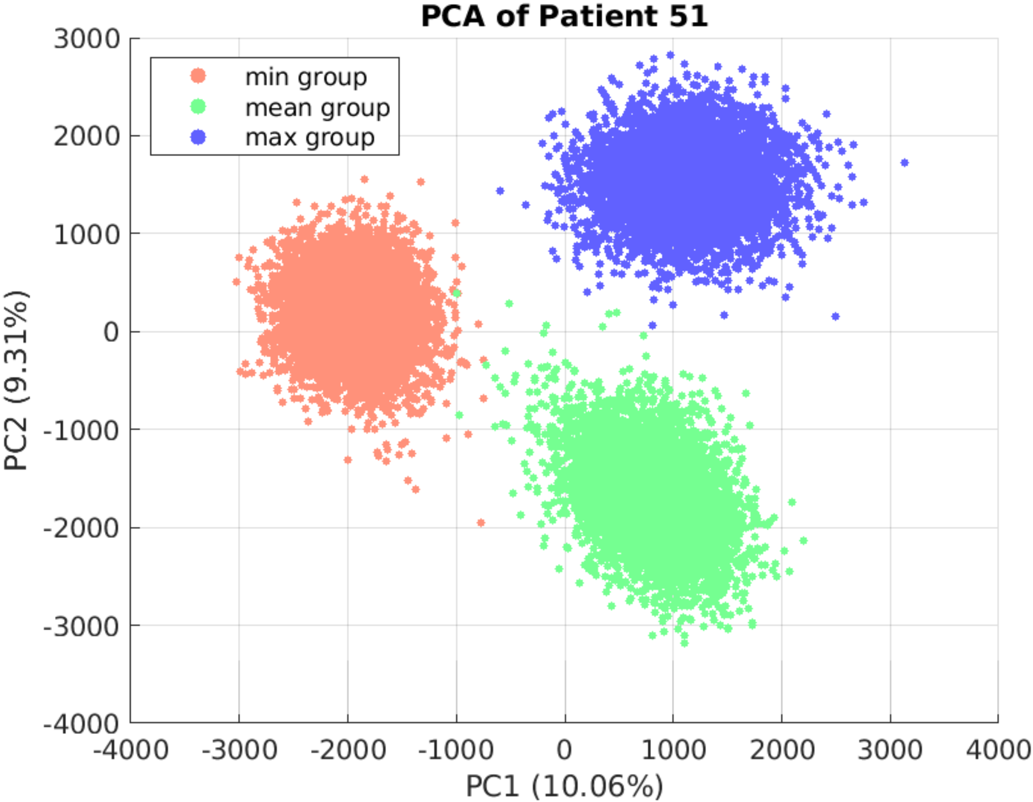

PCA of Patient 51
min group
mean group
max group
PC2 (9.31%)
PC1 (10.06%)
3000
2000
1000
0
-1000
-2000
-3000
-4000
-4000
-3000
-2000
-1000
0
1000
2000
3000
4000

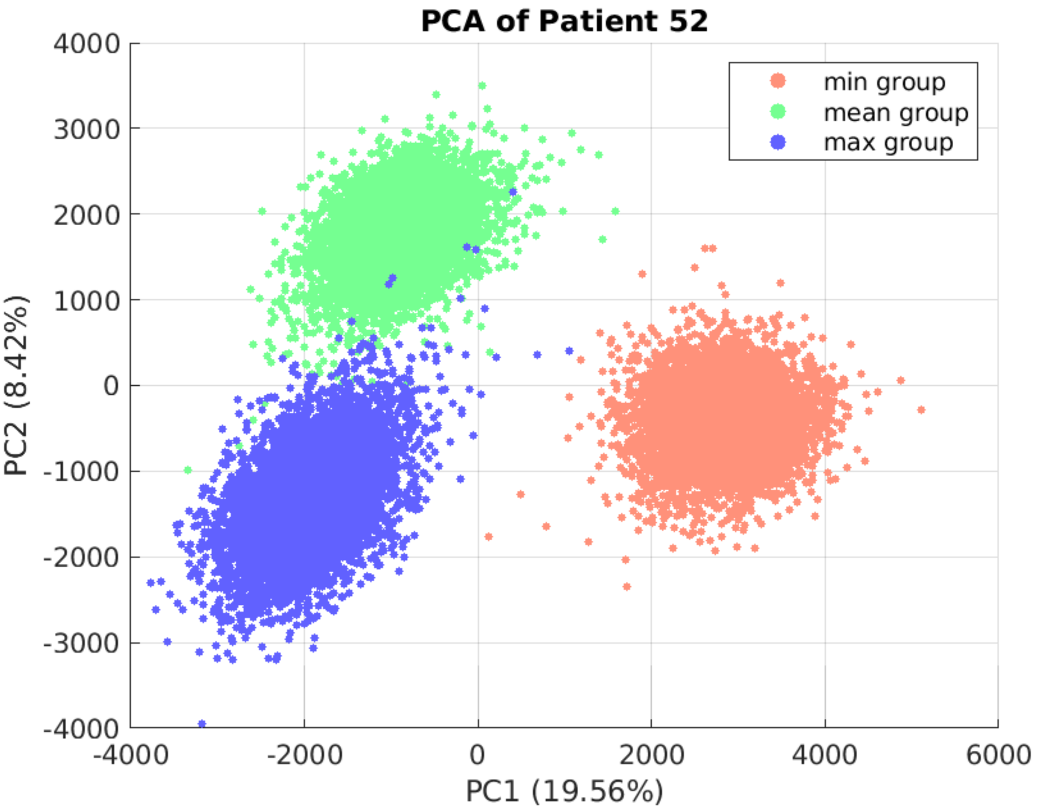
PCA of Patient 52
min group
mean group
max group
PC1 (19.56%)
PC2 (8.42%)
-4000
-2000
0
2000
4000
6000
-3000
-1000
1000
3000

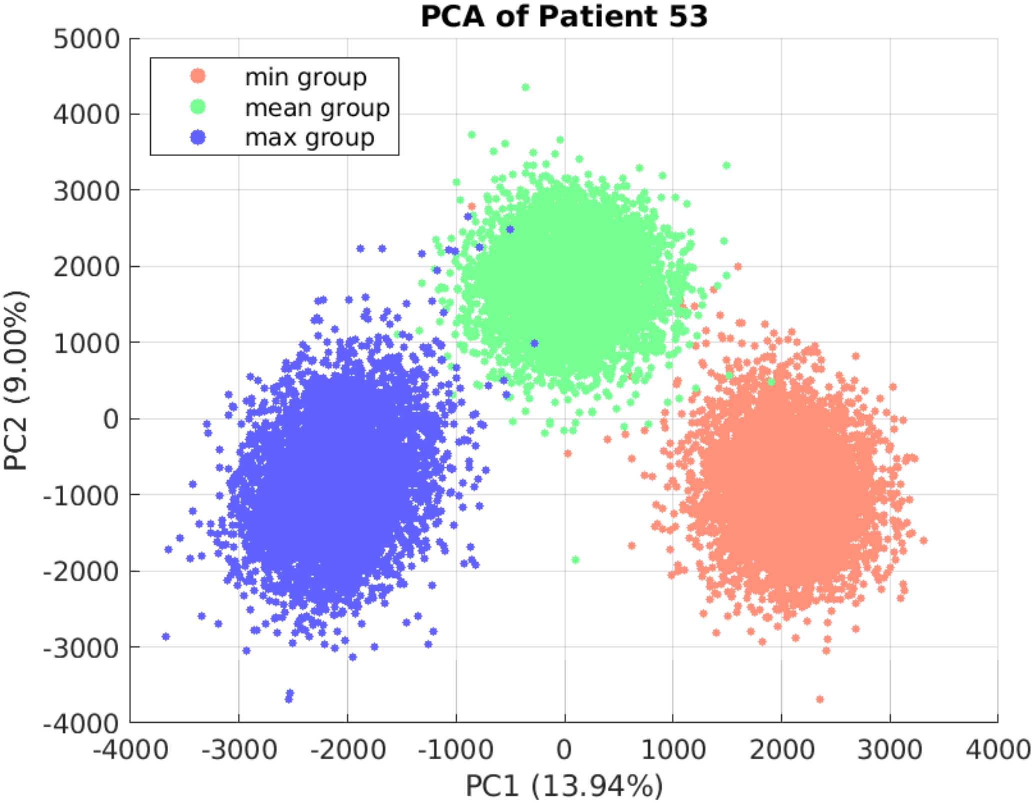
PCA of Patient 53
min group
mean group
max group
PC1 (13.94%)
PC2 (9.00%)
5000
4000
3000
2000
1000
0
-1000
-2000
-3000
-4000
-4000
-3000
-2000
-1000
0
1000
2000
3000
4000

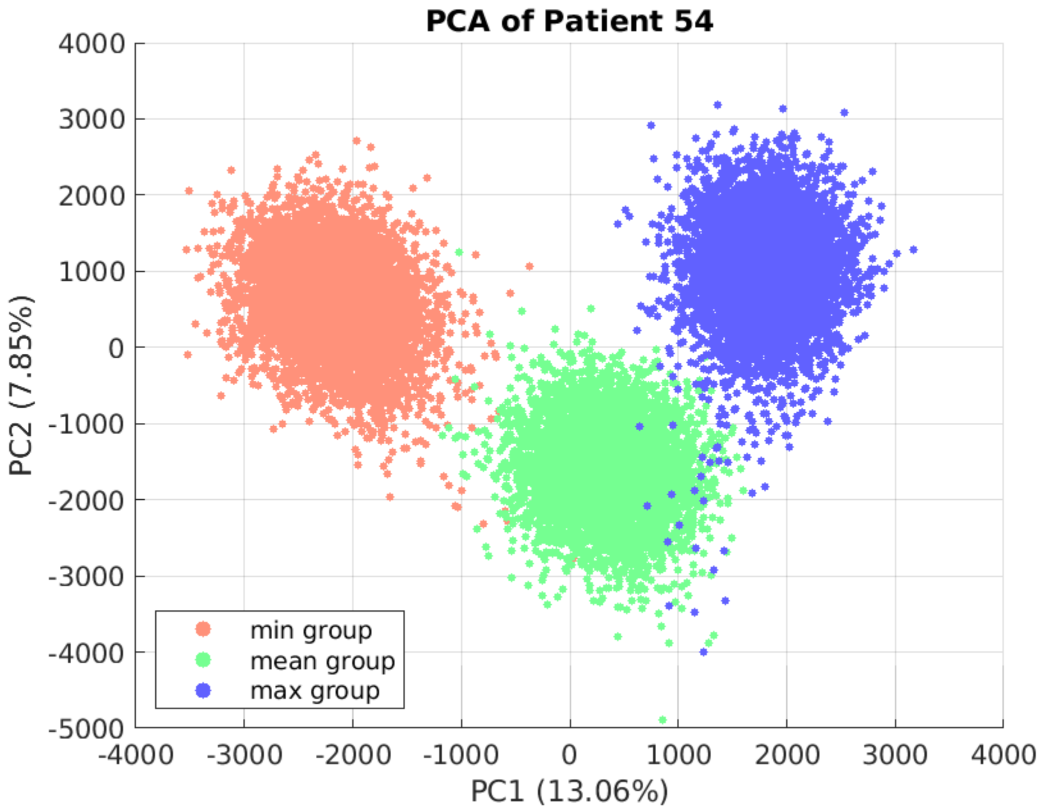
PCA of Patient 54
PC2 (7.85%)
PC1 (13.06%)
4000
3000
2000
1000
0
-1000
-2000
-3000
-4000
-5000
-4000
-3000
-2000
-1000
0
1000
2000
3000
4000
min group
mean group
max group

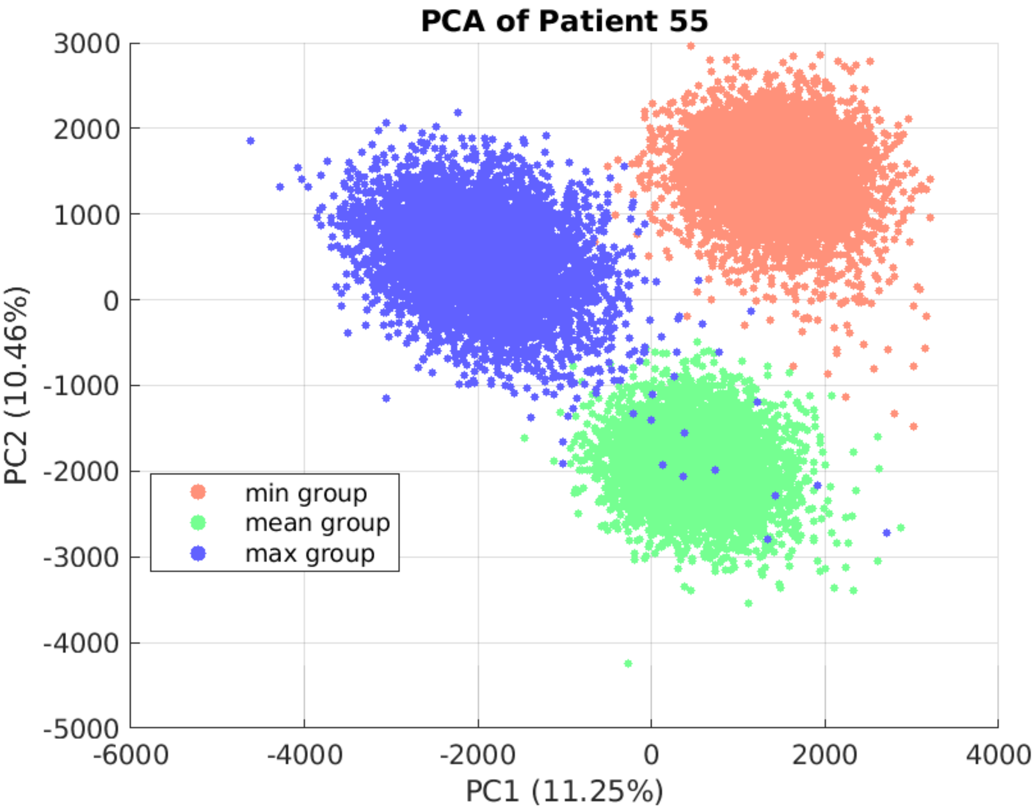
PCA of Patient 55
PC1 (11.25%)
PC2 (10.46%)
min group
mean group
max group
-6000
-4000
-2000
0
2000
4000
3000
2000
1000
0
-1000
-2000
-3000
-4000
-5000

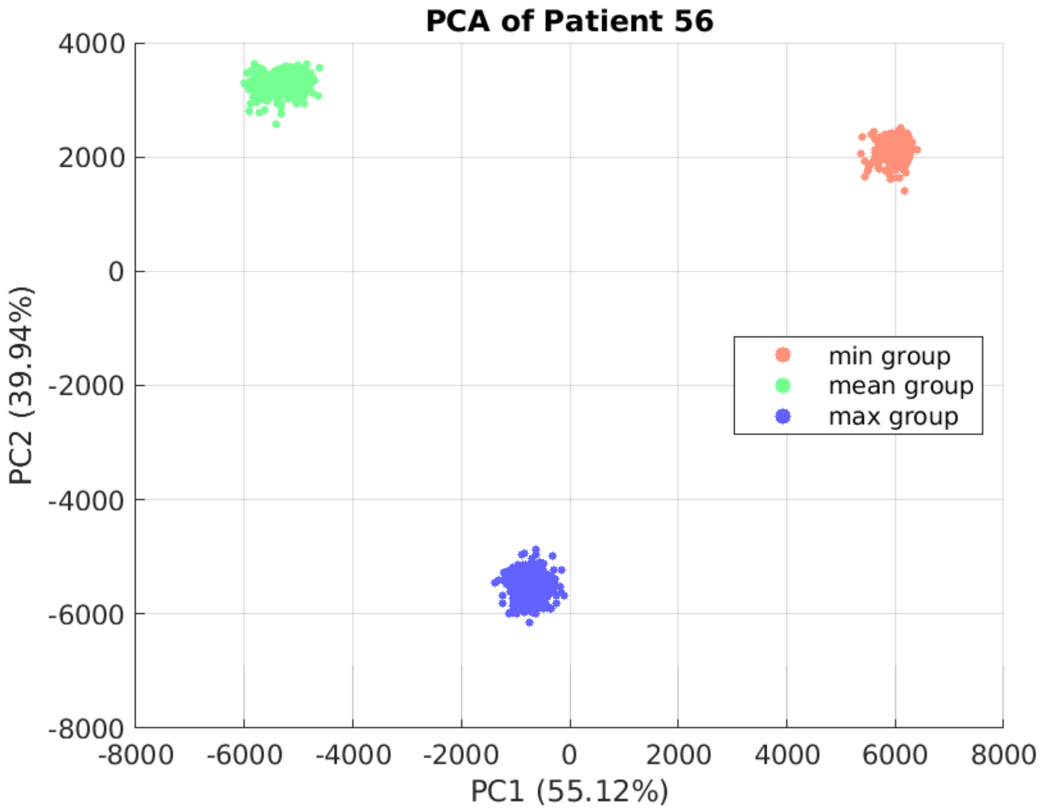
PCA of Patient 56
PC1 (55.12%)
PC2 (39.94%)
-8000
-6000
-4000
-2000
0
2000
4000
6000
8000
min group
mean group
max group

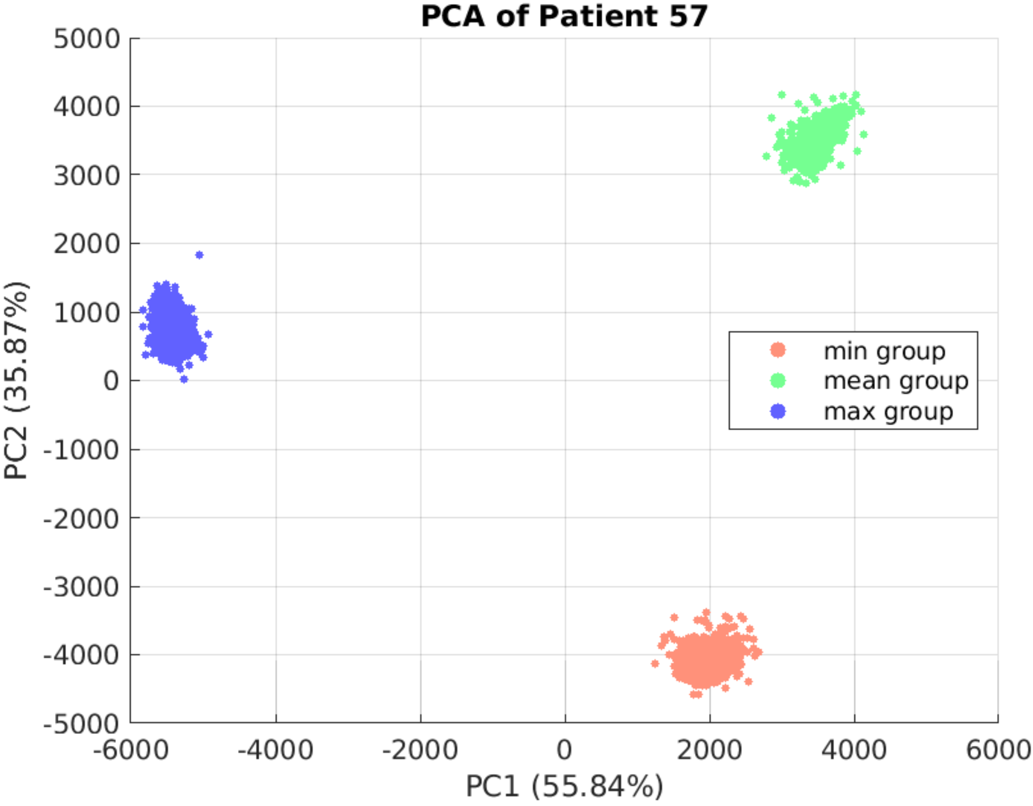

PCA of Patient 57
5000
4000
3000
2000
1000
0
-1000
-2000
-3000
-4000
-5000
PC2 (35.87%)
-6000
-4000
-2000
0
2000
4000
6000
PC1 (55.84%)
min group
mean group
max group

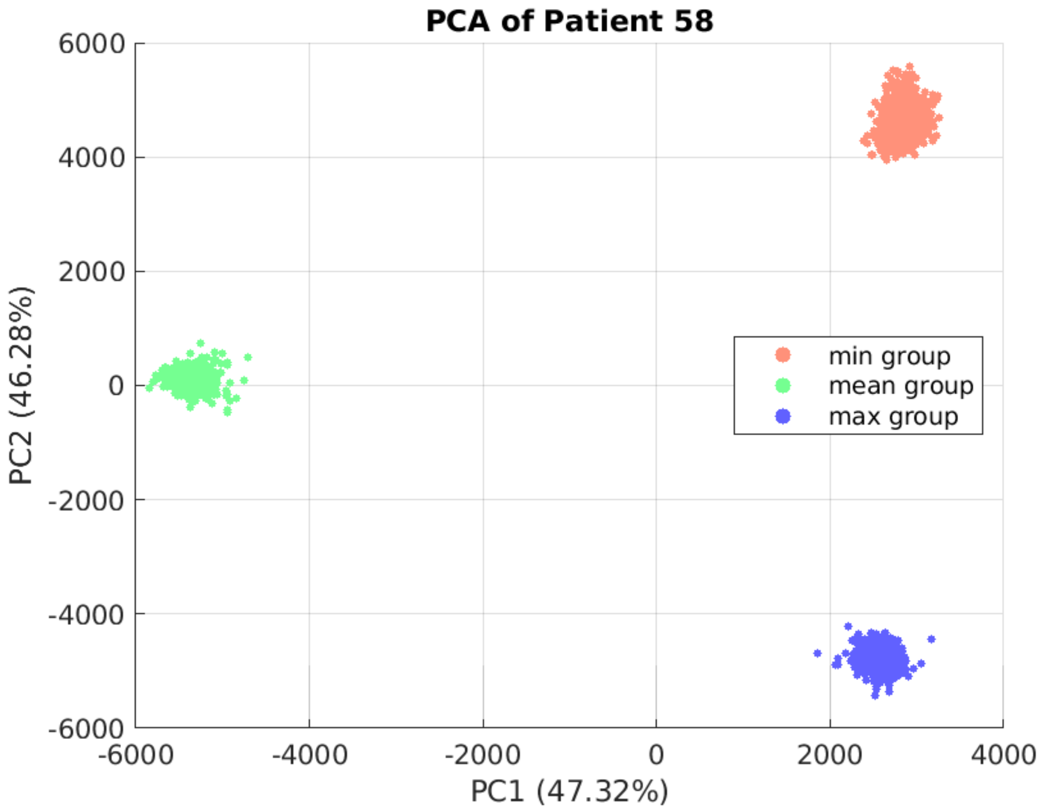
PCA of Patient 58
min group
mean group
max group
PC1 (47.32%)
PC2 (46.28%)
6000
4000
2000
0
-2000
-4000
-6000
-6000
-4000
-2000
0
2000
4000

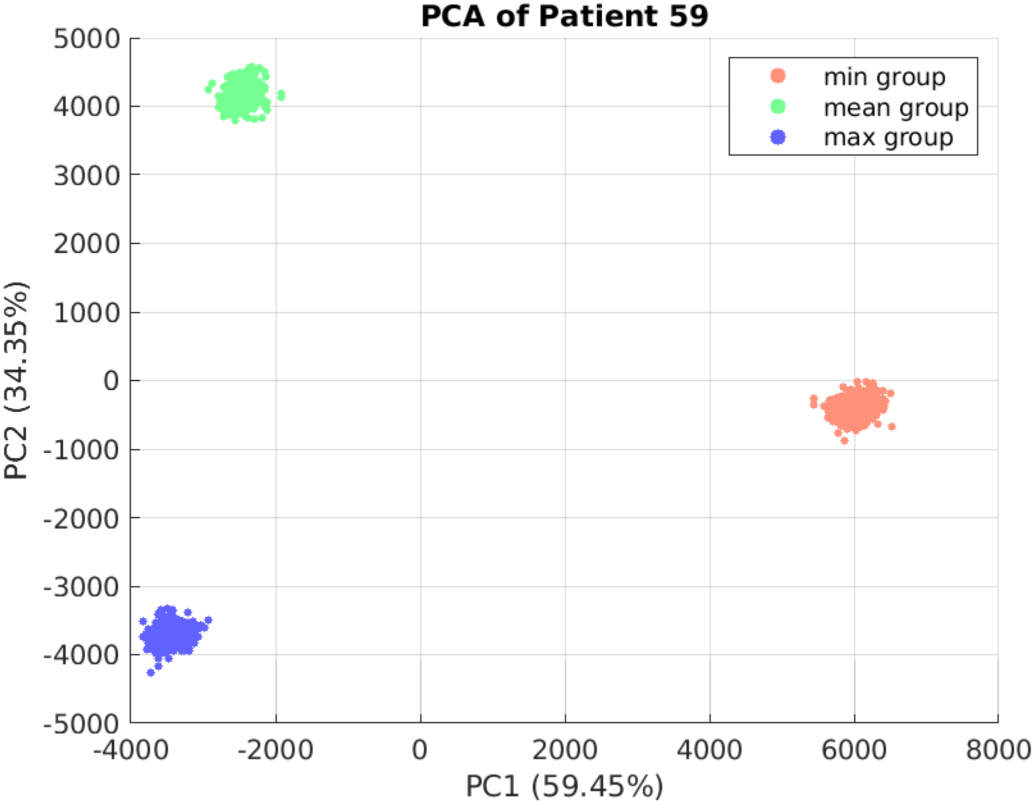
PCA of Patient 59
min group
mean group
max group
PC1 (59.45%)
PC2 (34.35%)
5000
4000
3000
2000
1000
0
-1000
-2000
-3000
-4000
-5000
-4000
-2000
0
2000
4000
6000
8000

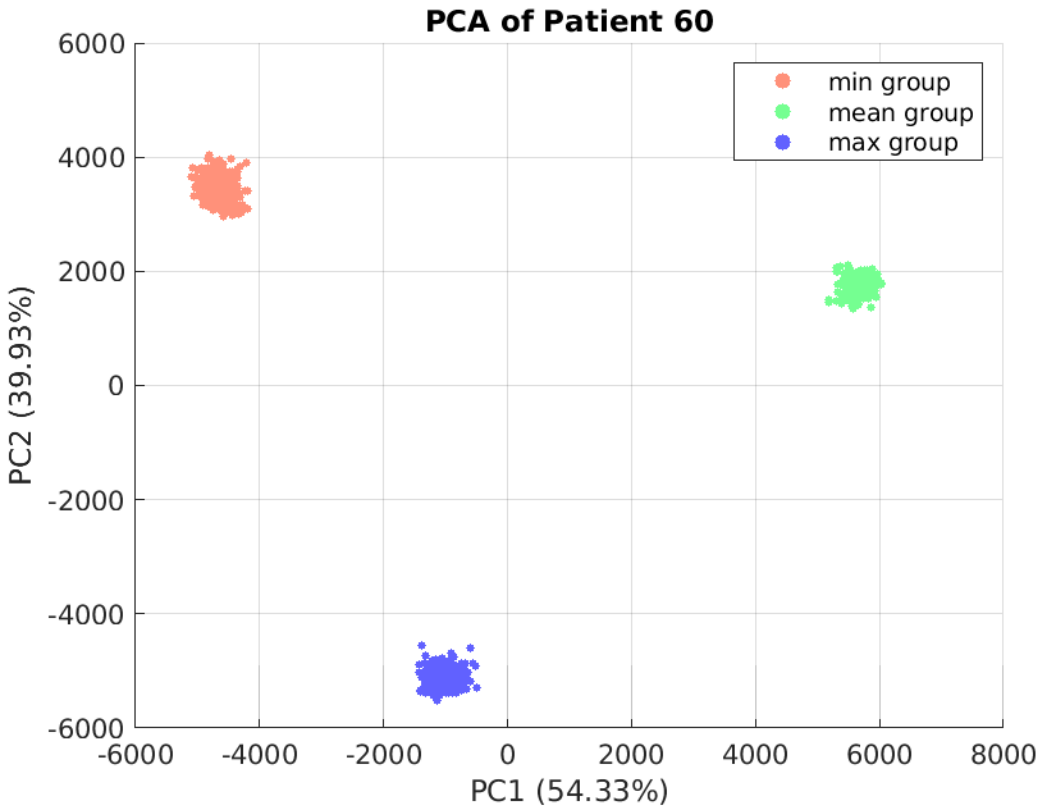
PCA of Patient 60
min group
mean group
max group
PC1 (54.33%)
PC2 (39.93%)
6000
4000
2000
0
-2000
-4000
-6000
-6000
-4000
-2000
0
2000
4000
6000
8000

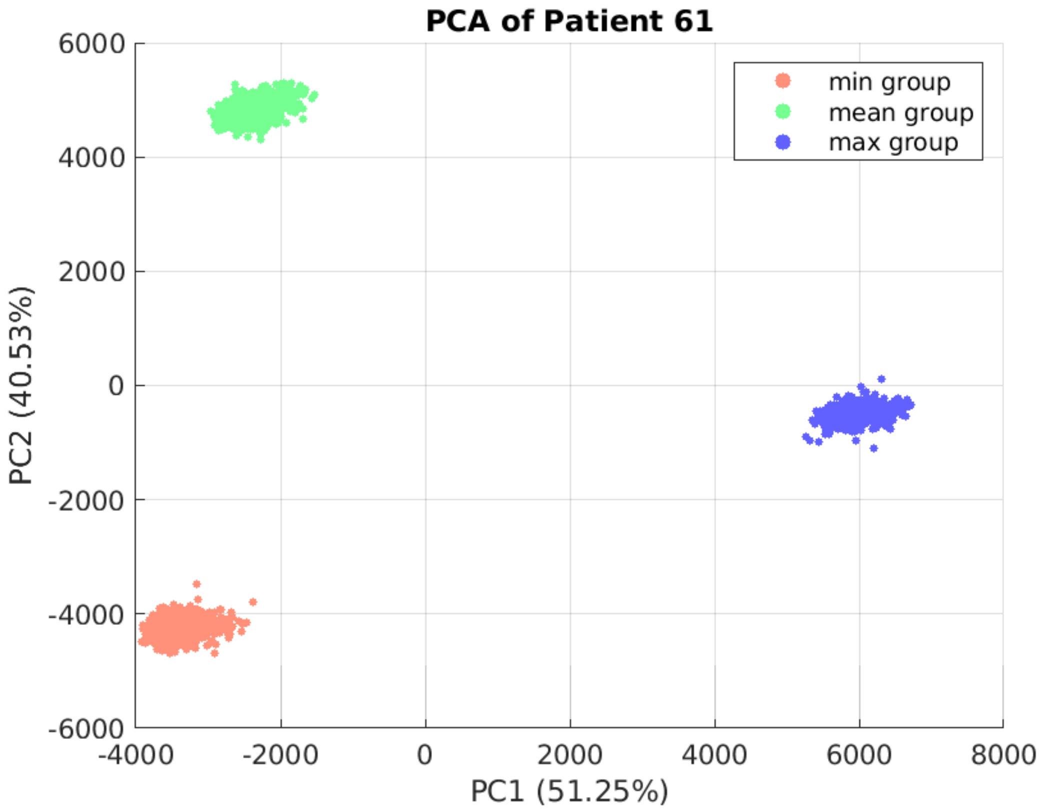
PCA of Patient 61
min group
mean group
max group
6000
4000
2000
0
-2000
-4000
-6000
-4000
-2000
0
2000
4000
6000
8000
PC1 (51.25%)
PC2 (40.53%)

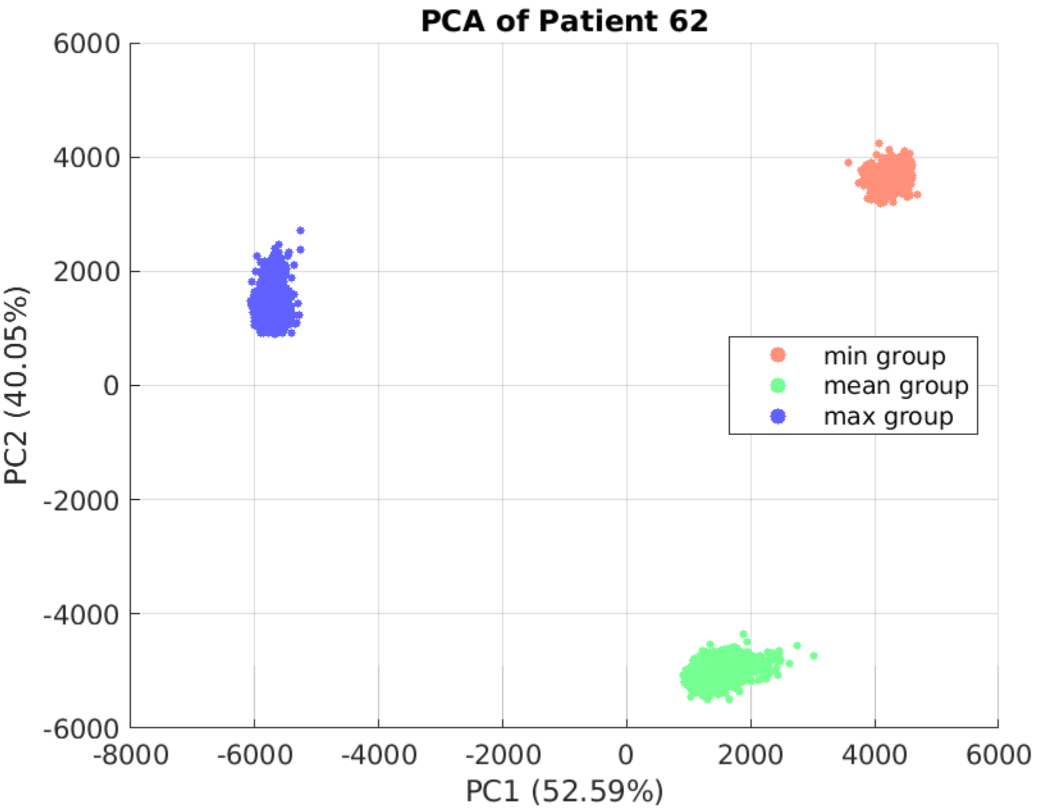
PCA of Patient 62
6000
4000
2000
0
-2000
-4000
-6000
-8000
-6000
-4000
-2000
0
2000
4000
6000
PC2 (40.05%)
PC1 (52.59%)
min group
mean group
max group

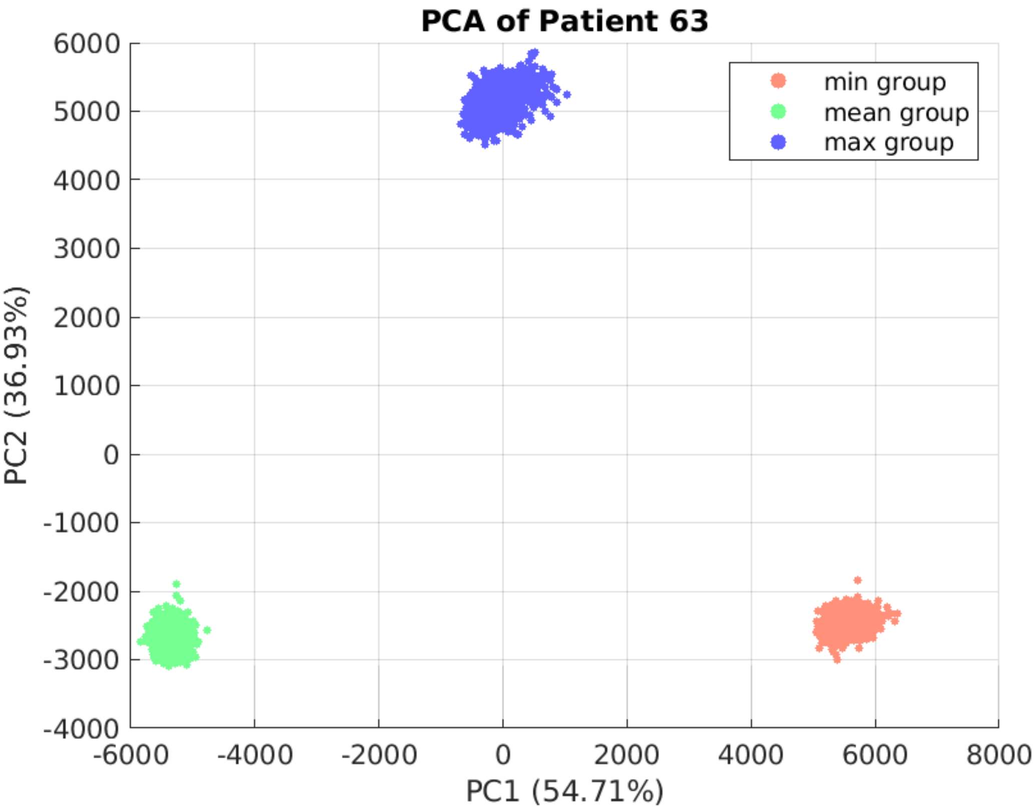
PCA of Patient 63
min group
mean group
max group
6000
5000
4000
3000
2000
1000
0
-1000
-2000
-3000
-4000
-6000
-4000
-2000
0
2000
4000
6000
8000
PC1 (54.71%)
PC2 (36.93%)

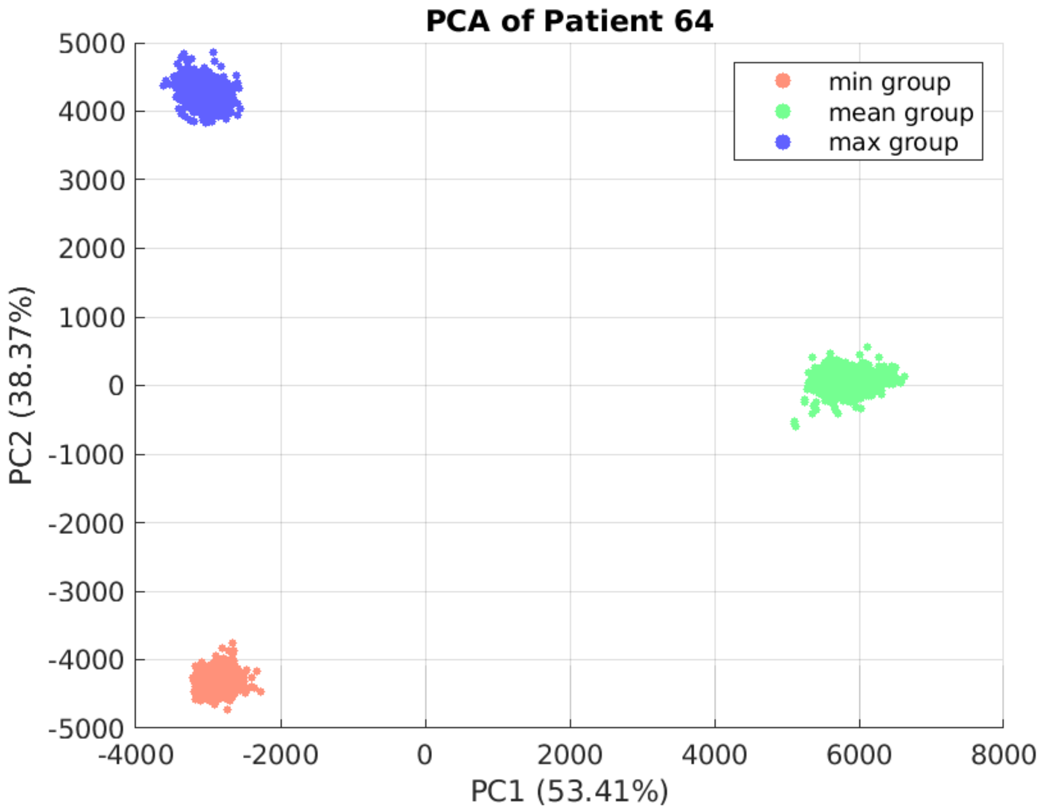
PCA of Patient 64
min group
mean group
max group
PC1 (53.41%)
PC2 (38.37%)
-4000
-2000
0
2000
4000
6000
8000
5000
4000
3000
2000
1000
0
-1000
-2000
-3000
-4000
-5000

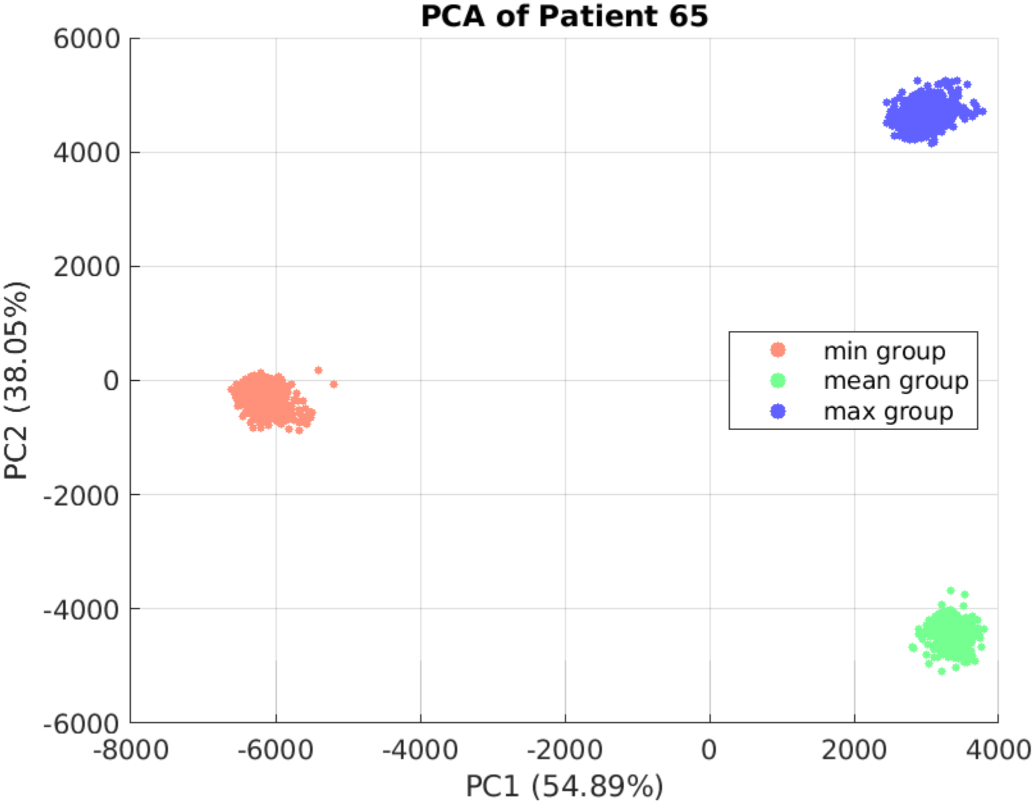
PCA of Patient 65
PC1 (54.89%)
PC2 (38.05%)
min group
mean group
max group
-8000
-6000
-4000
-2000
0
2000
4000
6000

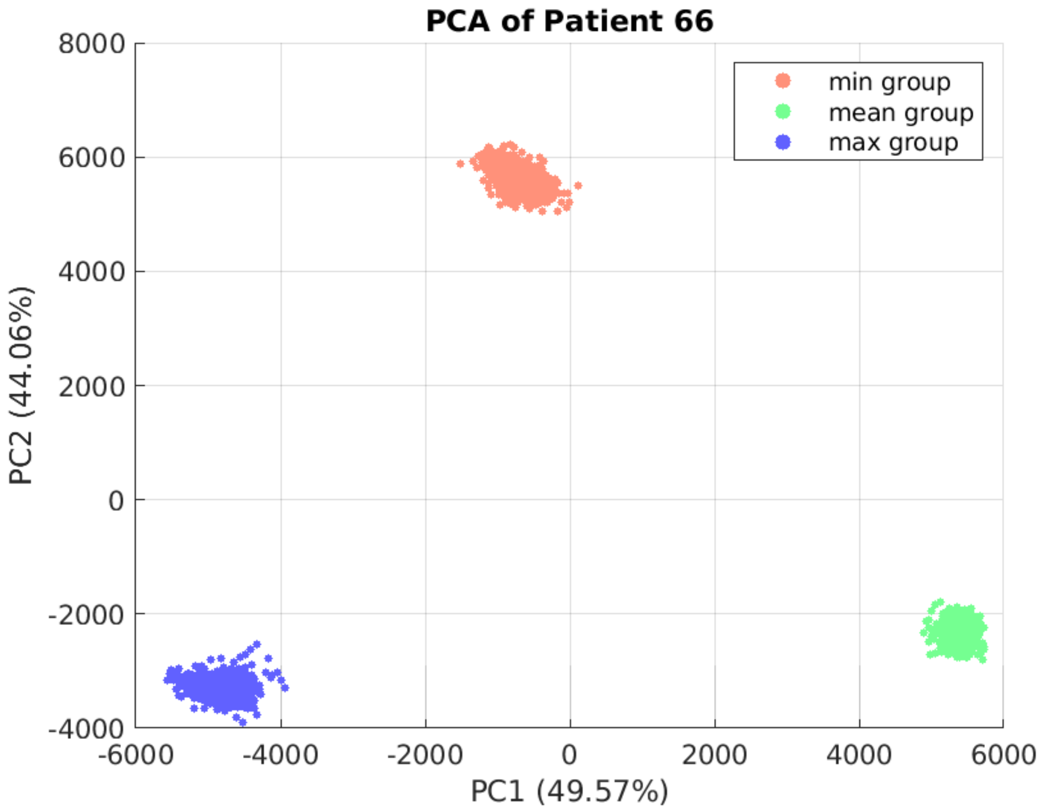
PCA of Patient 66
min group
mean group
max group
PC2 (44.06%)
PC1 (49.57%)
8000
6000
4000
2000
0
-2000
-4000
-6000
-4000
-2000
0
2000
4000
6000

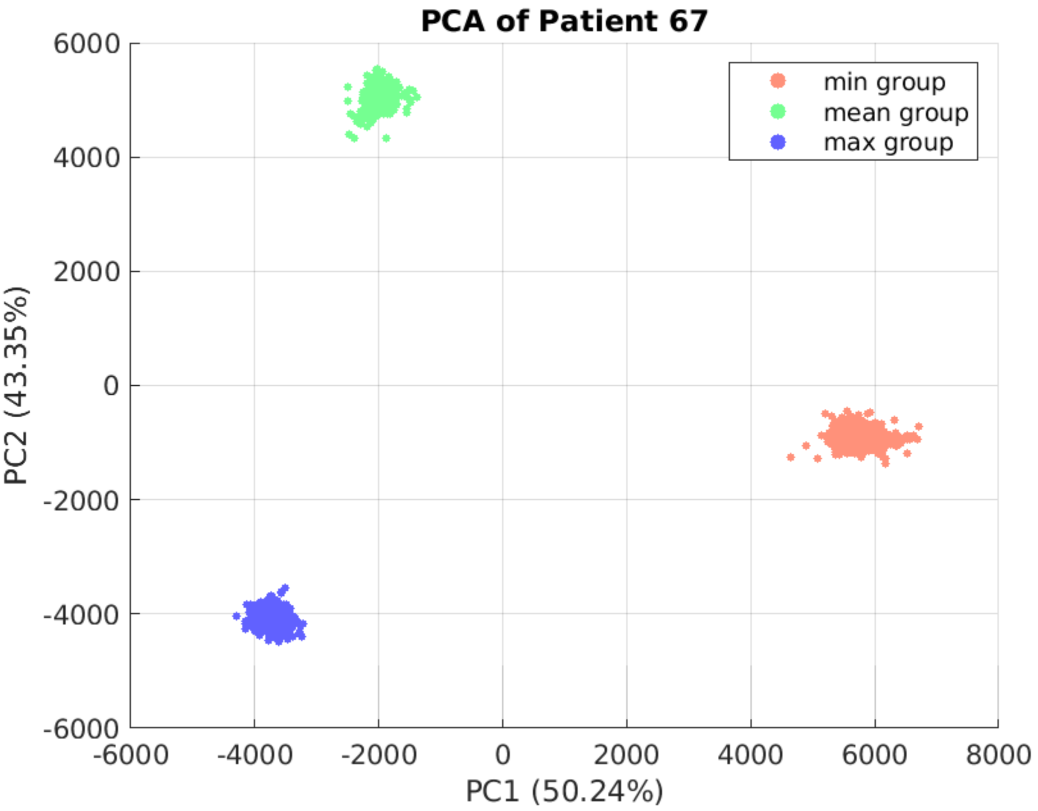
PCA of Patient 67
min group
mean group
max group
PC1 (50.24%)
PC2 (43.35%)
6000
4000
2000
0
-2000
-4000
-6000
-6000
-4000
-2000
0
2000
4000
6000
8000

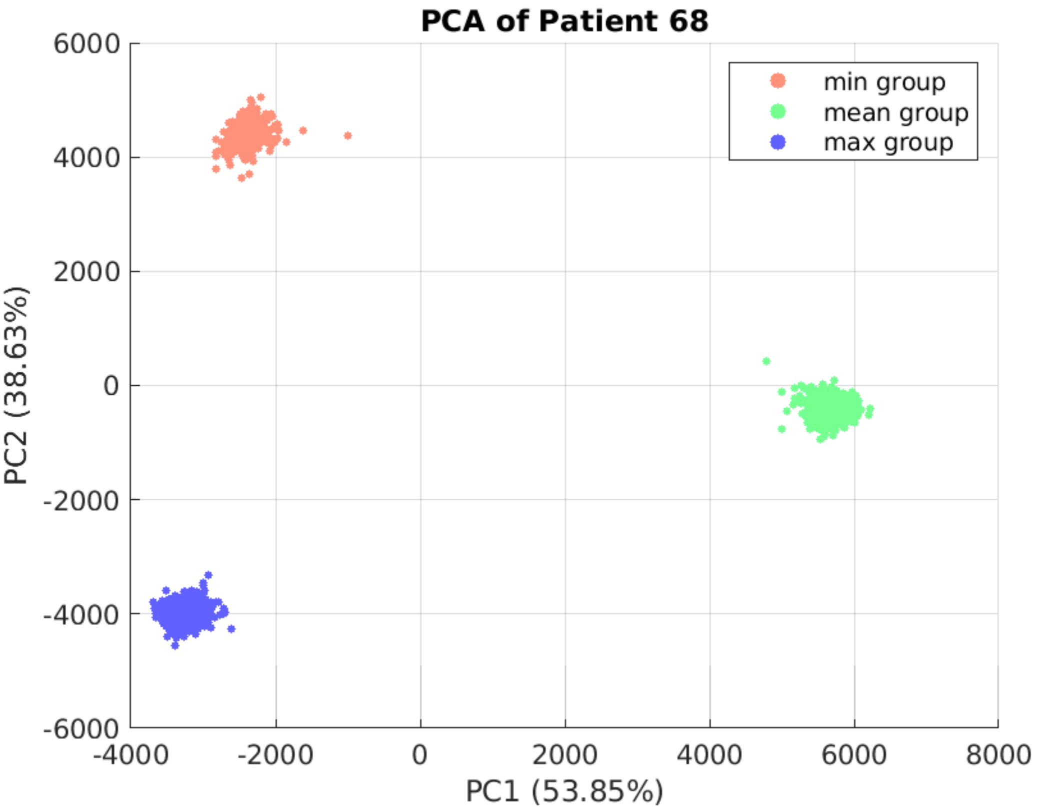
PCA of Patient 68
min group
mean group
max group
6000
4000
2000
0
-2000
-4000
-6000
-4000
-2000
0
2000
4000
6000
8000
PC1 (53.85%)
PC2 (38.63%)

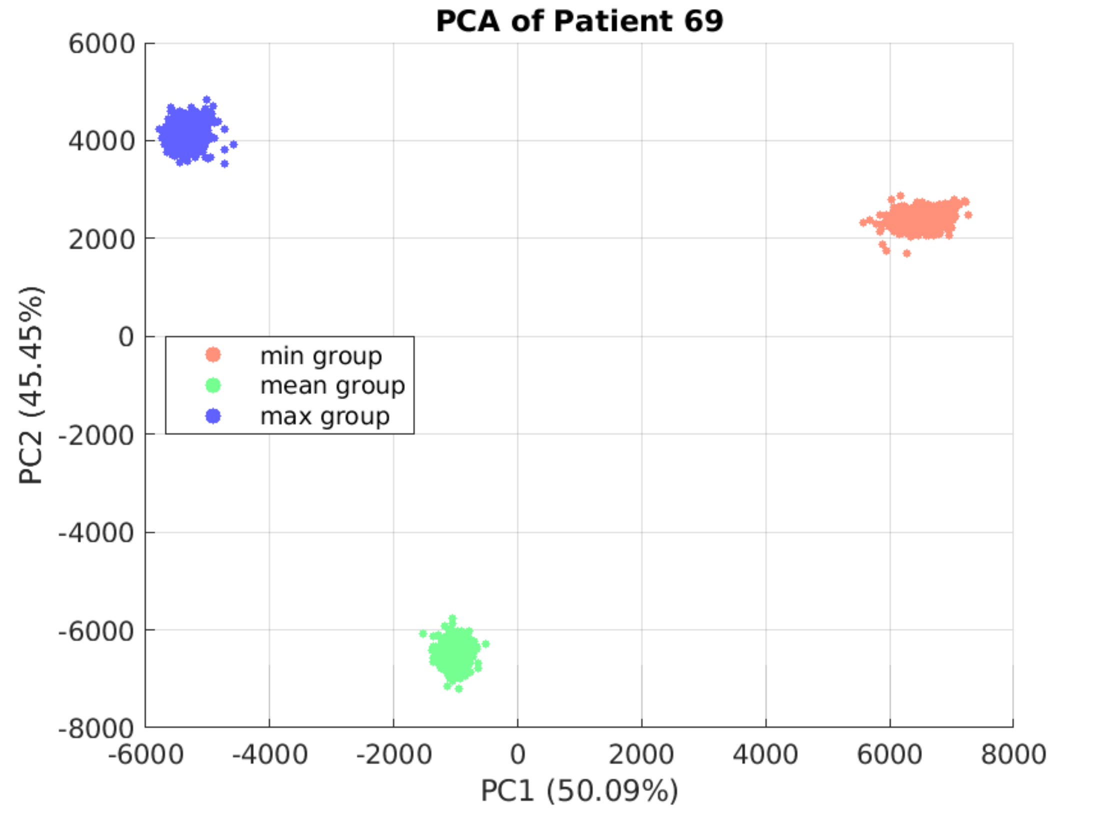
PCA of Patient 69
PC2 (45.45%)
PC1 (50.09%)
6000
4000
2000
0
-2000
-4000
-6000
-8000
-6000
-4000
-2000
0
2000
4000
6000
8000
min group
mean group
max group

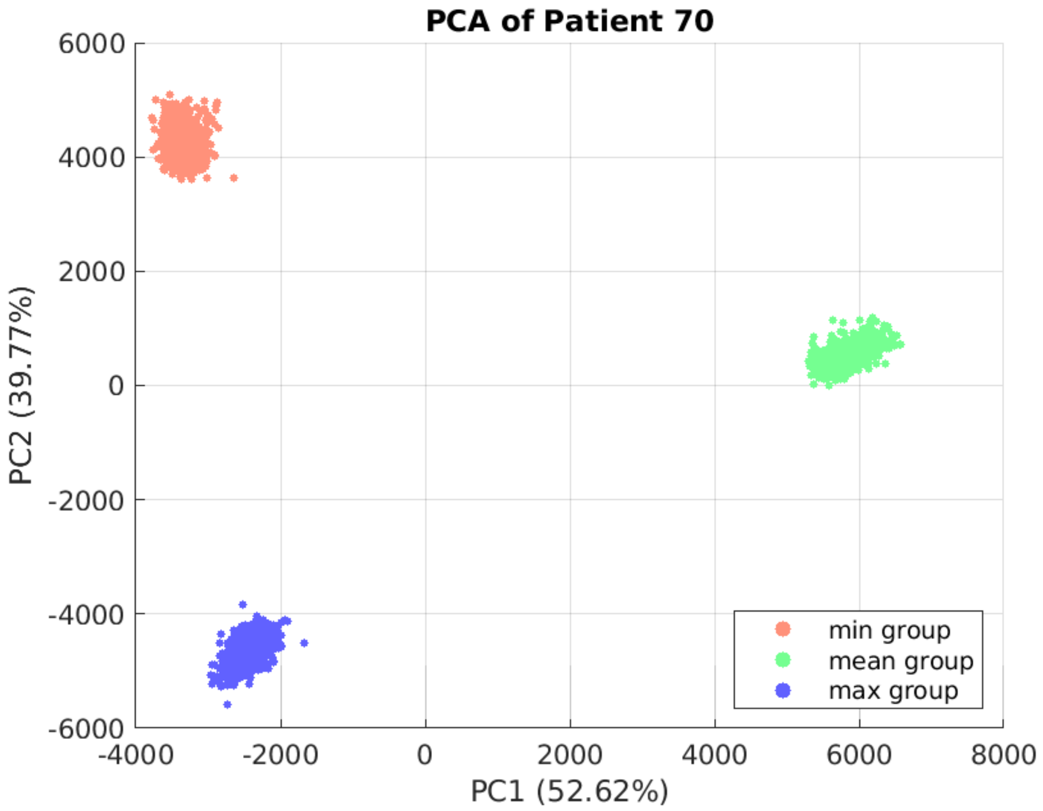
PCA of Patient 70
6000
4000
2000
0
-2000
-4000
-6000
-4000
-2000
0
2000
4000
6000
8000
PC2 (39.77%)
PC1 (52.62%)
min group
mean group
max group

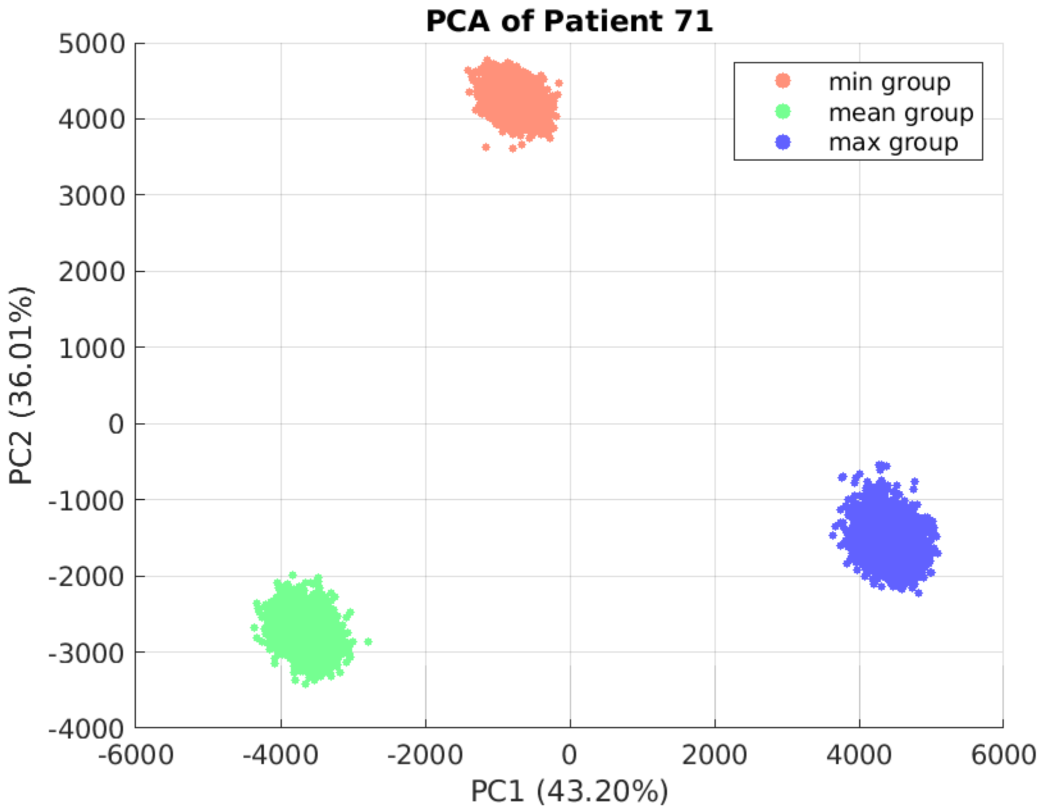
PCA of Patient 71
min group
mean group
max group
5000
4000
3000
2000
1000
0
-1000
-2000
-3000
-4000
-6000
-4000
-2000
0
2000
4000
6000
PC1 (43.20%)
PC2 (36.01%)

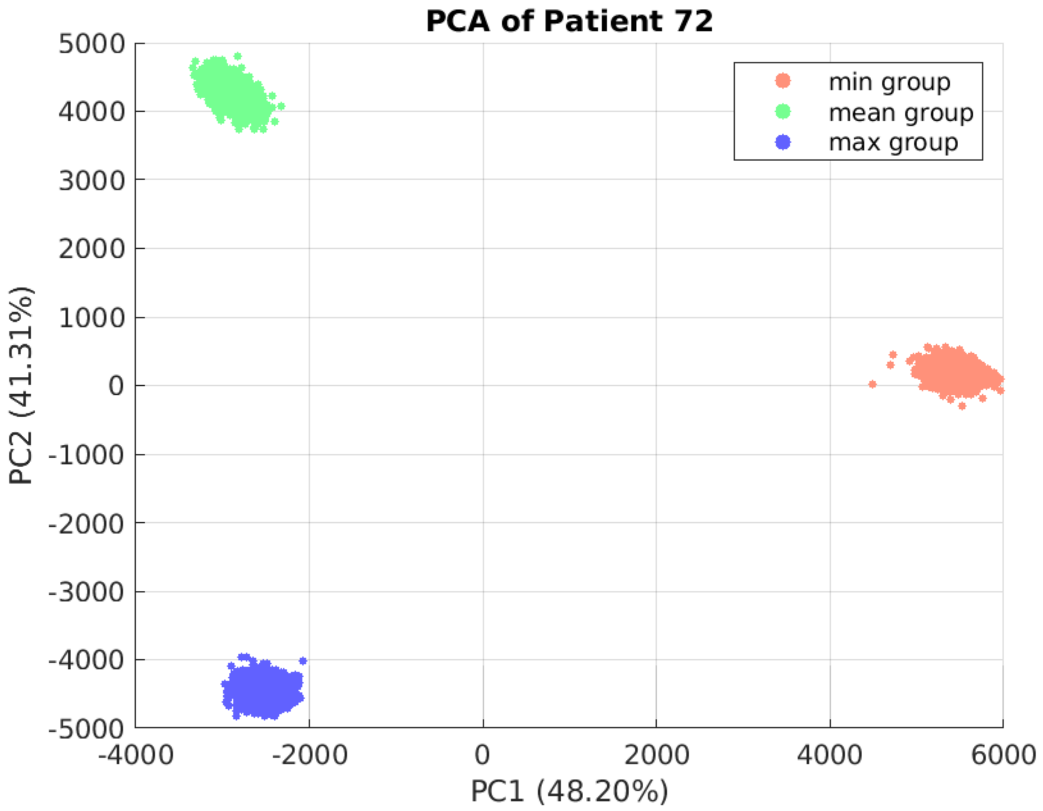
PCA of Patient 72
min group
mean group
max group
PC1 (48.20%)
PC2 (41.31%)
5000
4000
3000
2000
1000
0
-1000
-2000
-3000
-4000
-5000
-4000
-2000
0
2000
4000
6000

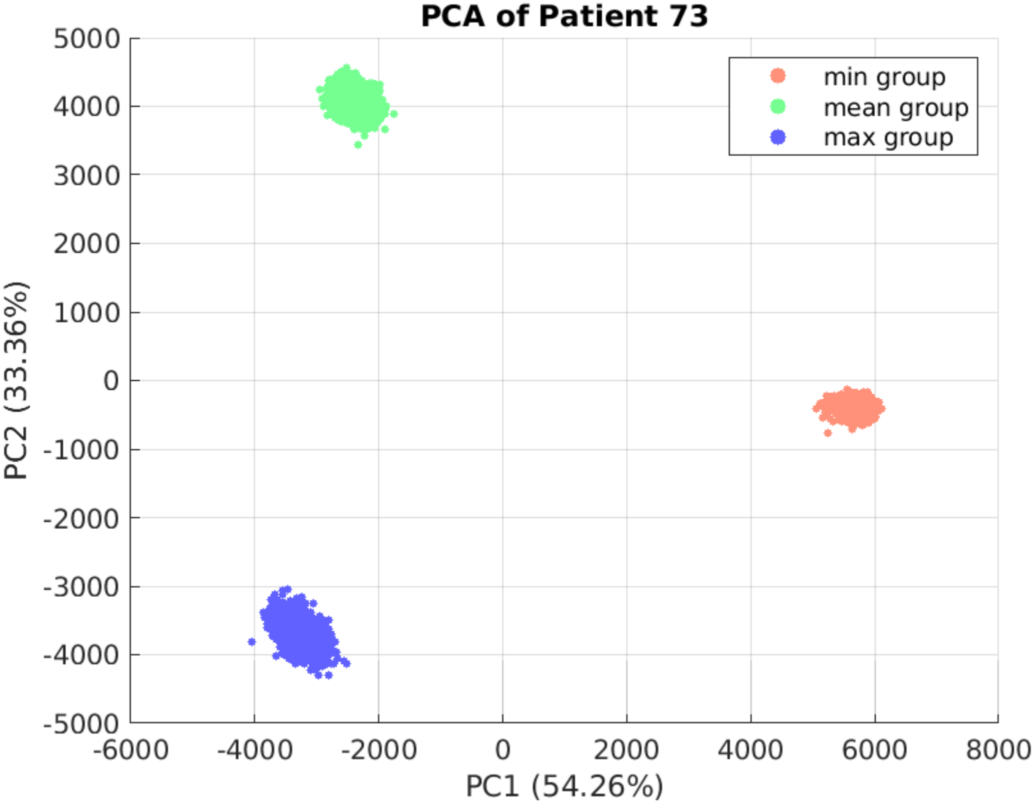
PCA of Patient 73
min group
mean group
max group
PC2 (33.36%)
PC1 (54.26%)
5000
4000
3000
2000
1000
0
-1000
-2000
-3000
-4000
-5000
-6000
-4000
-2000
0
2000
4000
6000
8000

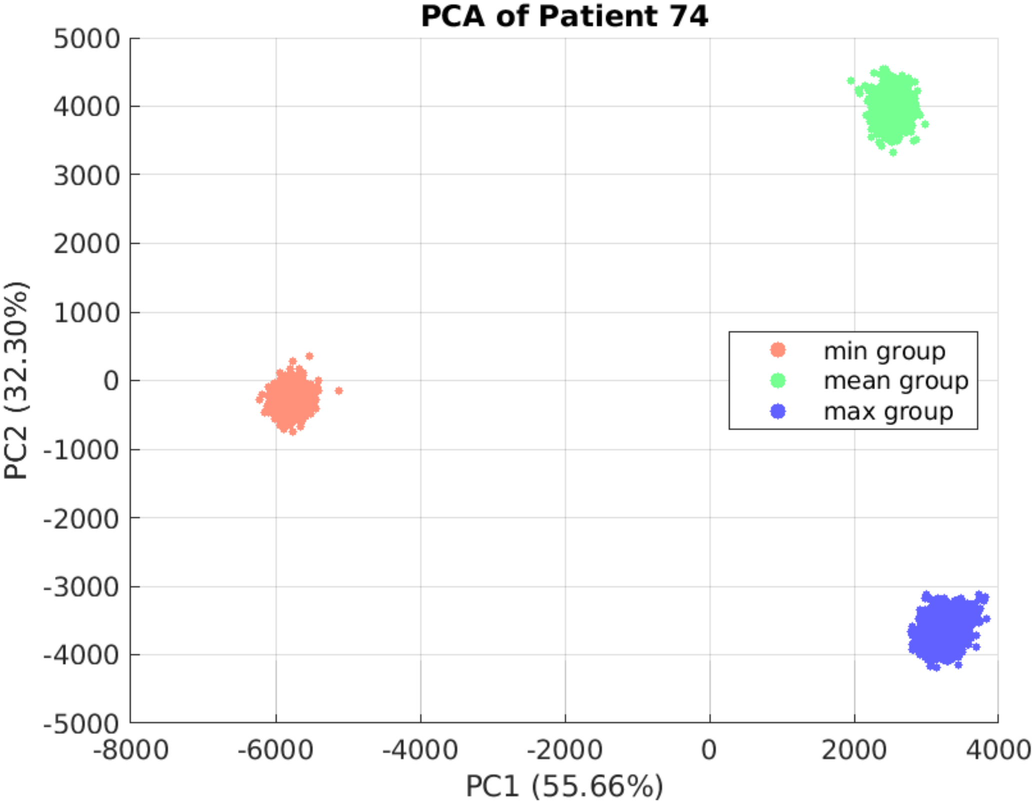

PCA of Patient 74
5000
4000
3000
2000
1000
0
-1000
-2000
-3000
-4000
-5000
-8000
-6000
-4000
-2000
0
2000
4000
PC1 (55.66%)
PC2 (32.30%)
min group
mean group
max group

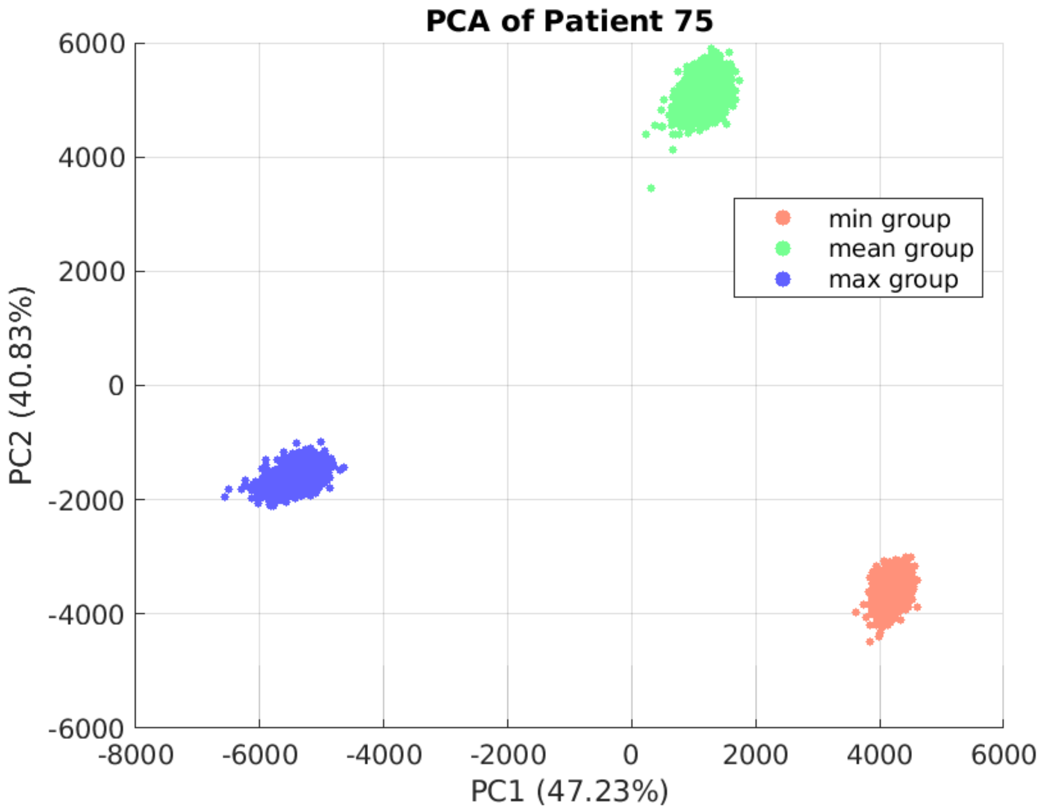
PCA of Patient 75
PC2 (40.83%)
PC1 (47.23%)
min group
mean group
max group
6000
4000
2000
0
-2000
-4000
-6000
-8000
-6000
-4000
-2000
0
2000
4000
6000

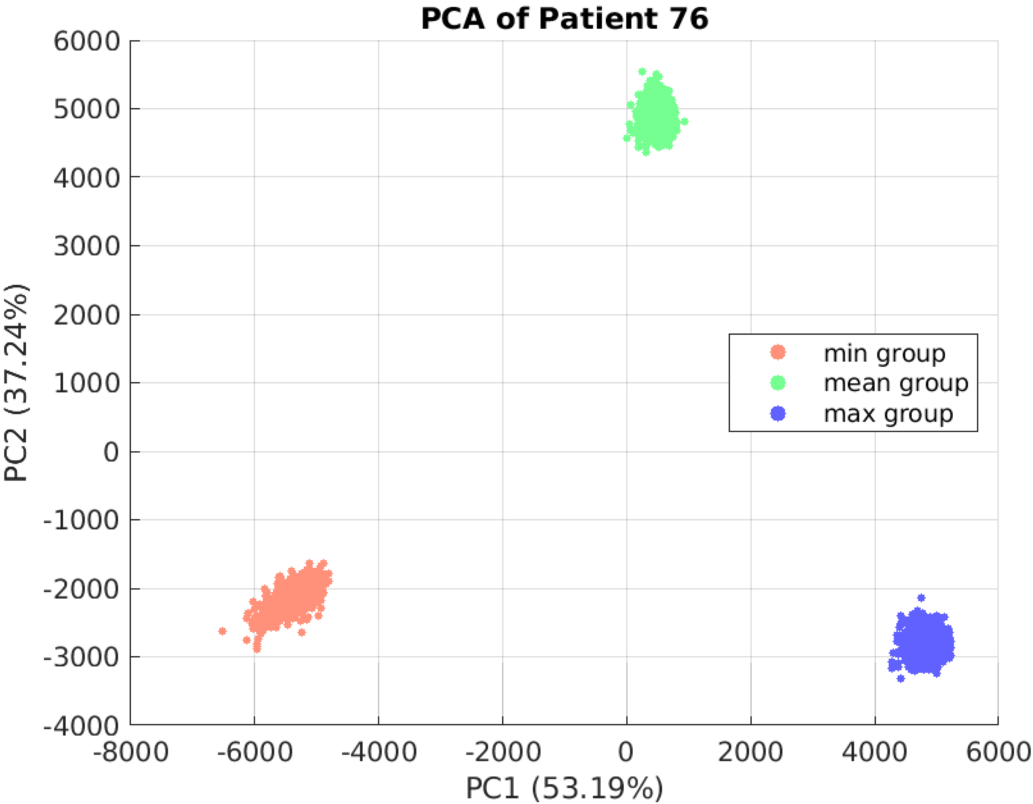

PCA of Patient 76
6000
5000
4000
3000
2000
1000
0
-1000
-2000
-3000
-4000
PC2 (37.24%)
-8000
-6000
-4000
-2000
0
2000
4000
6000
PC1 (53.19%)
min group
mean group
max group

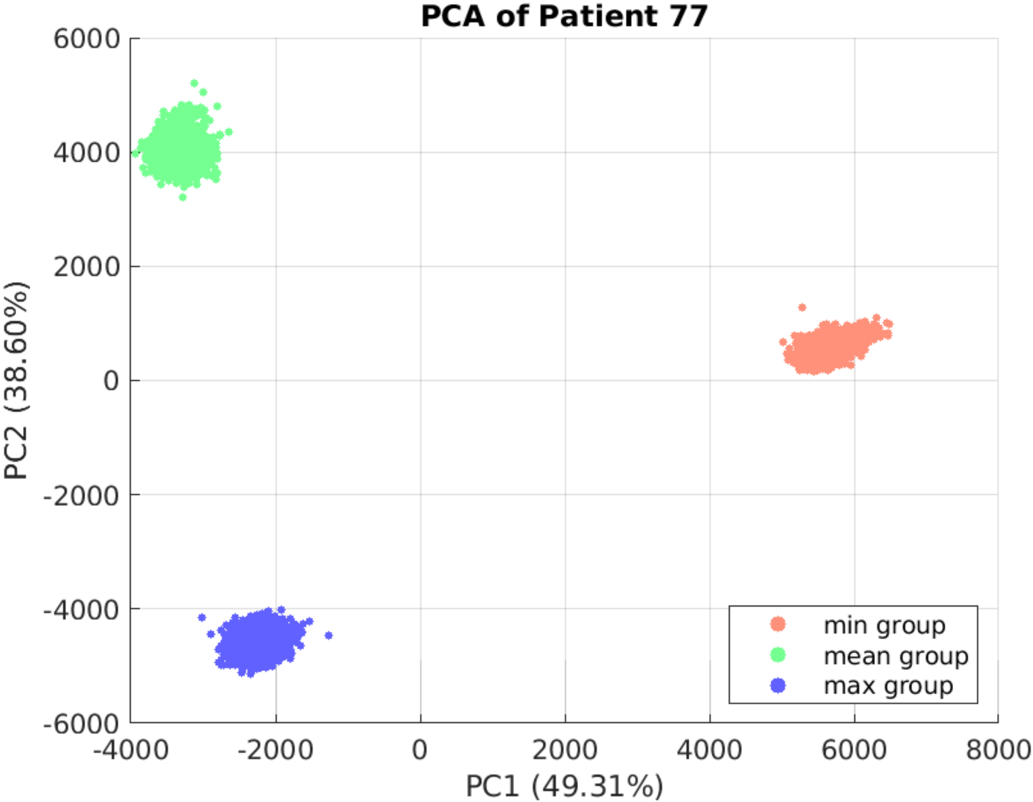
PCA of Patient 77
6000
4000
2000
0
-2000
-4000
-6000
-4000
-2000
0
2000
4000
6000
8000
PC1 (49.31%)
PC2 (38.60%)
min group
mean group
max group

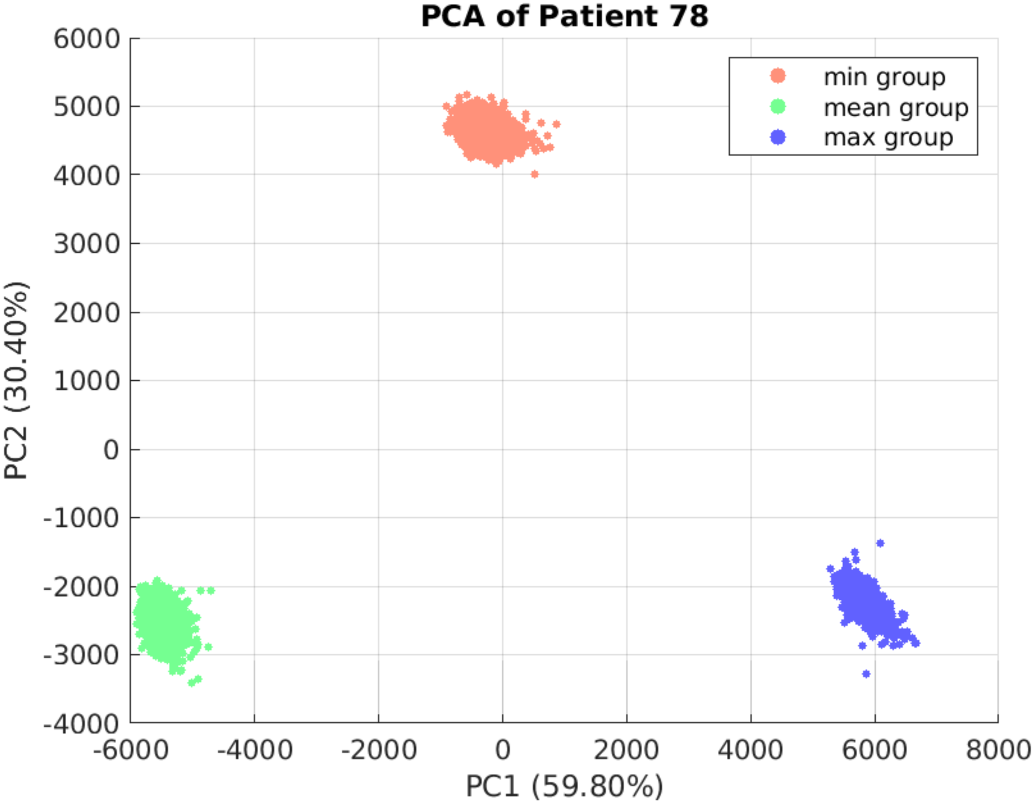

PCA of Patient 78
min group
mean group
max group
PC1 (59.80%)
PC2 (30.40%)
6000
5000
4000
3000
2000
1000
0
-1000
-2000
-3000
-4000
-6000
-4000
-2000
0
2000
4000
6000
8000

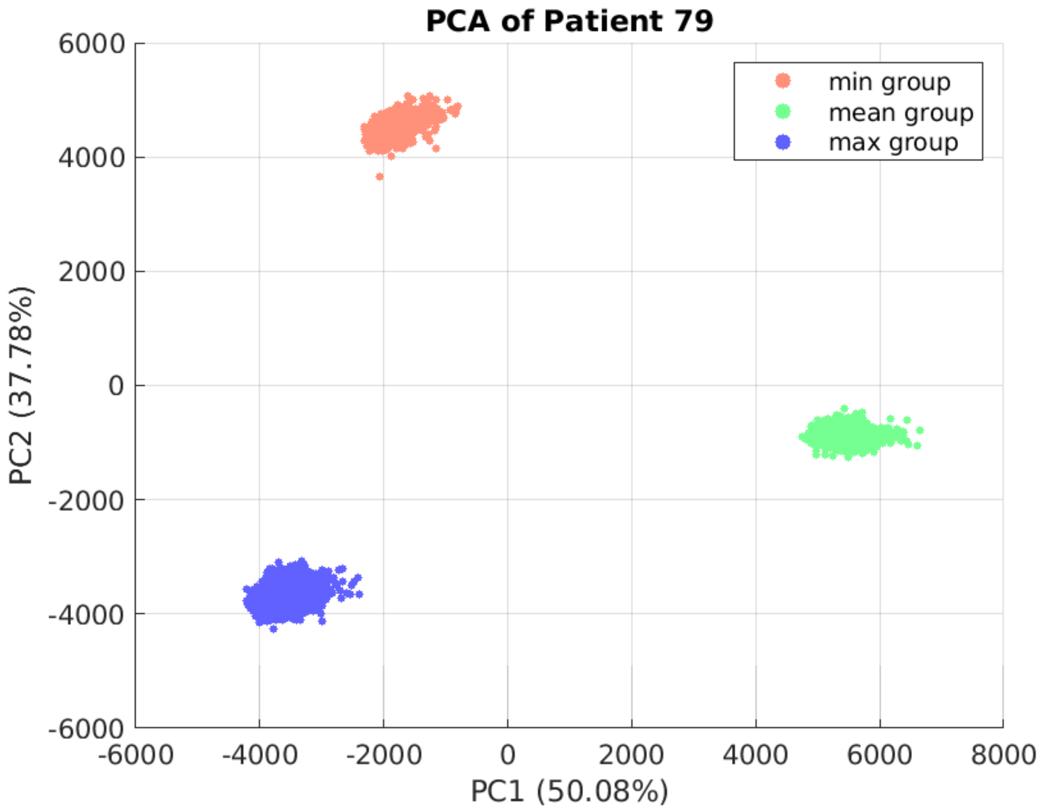
PCA of Patient 79
min group
mean group
max group
6000
4000
2000
0
-2000
-4000
-6000
-6000
-4000
-2000
0
2000
4000
6000
8000
PC1 (50.08%)
PC2 (37.78%)

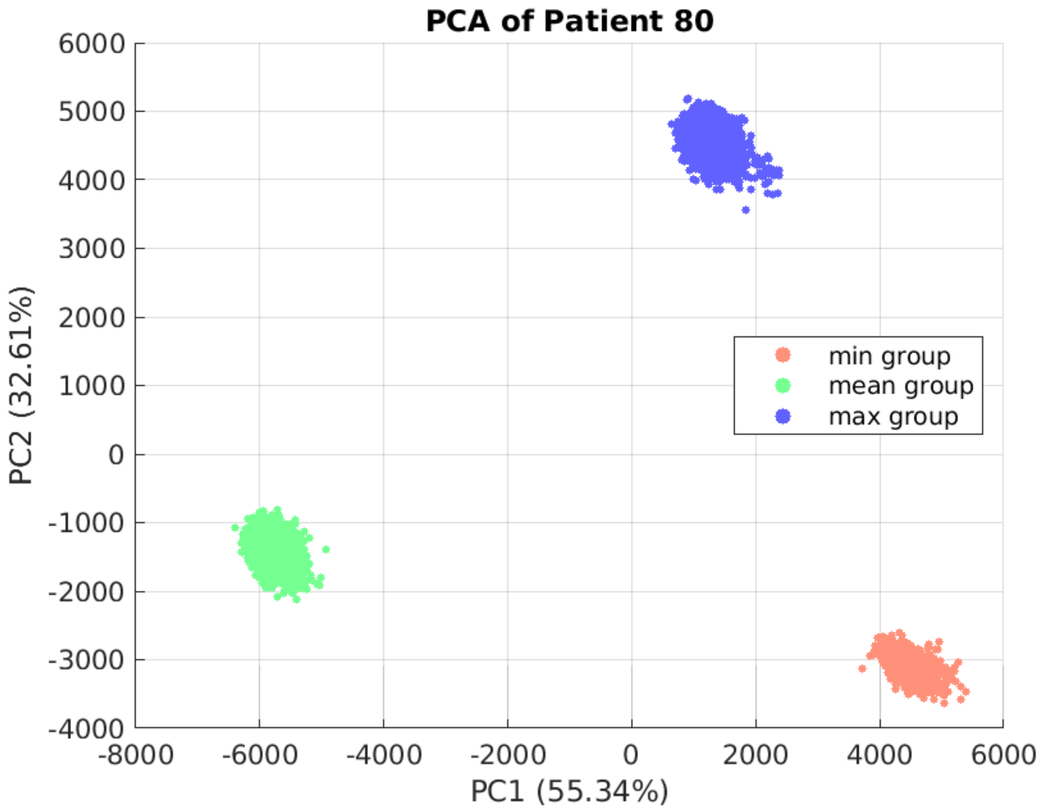
PCA of Patient 80
PC1 (55.34%)
PC2 (32.61%)
min group
mean group
max group
-8000
-6000
-4000
-2000
0
2000
4000
6000
-4000
-3000
-2000
-1000
0
1000
2000
3000
4000
5000
6000

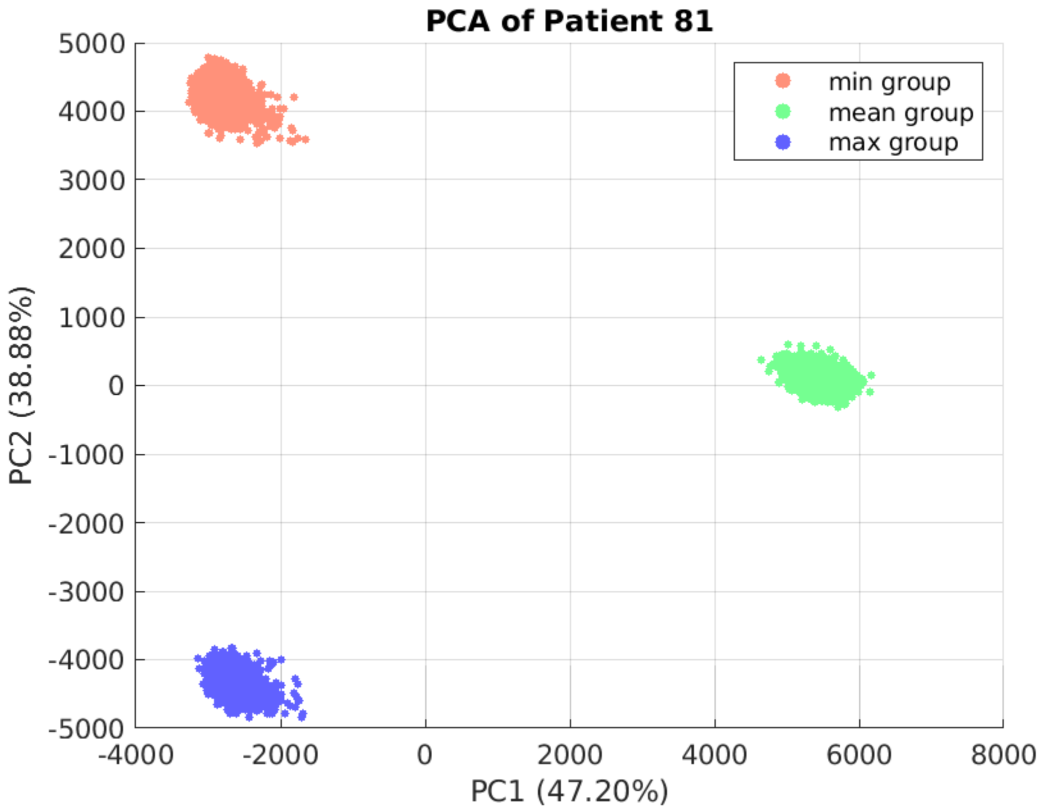
PCA of Patient 81
min group
mean group
max group
PC1 (47.20%)
PC2 (38.88%)
5000
4000
3000
2000
1000
0
-1000
-2000
-3000
-4000
-5000
-4000
-2000
0
2000
4000
6000
8000

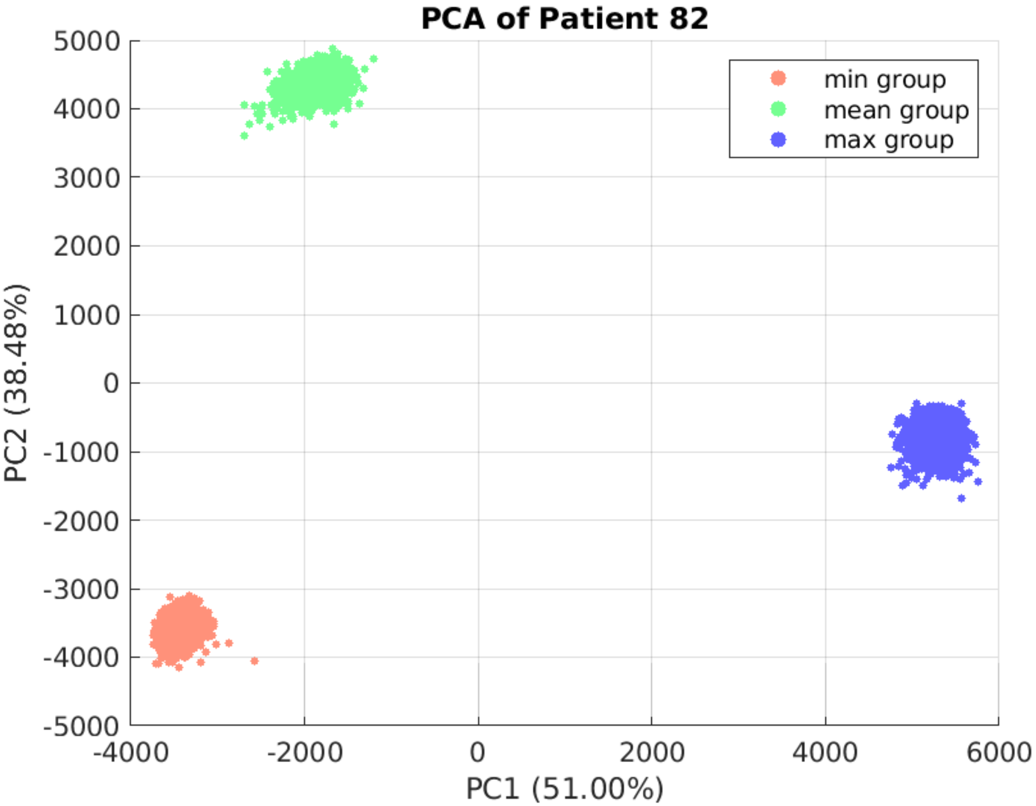
PCA of Patient 82
min group
mean group
max group
5000
4000
3000
2000
1000
0
-1000
-2000
-3000
-4000
-5000
-4000
-2000
0
2000
4000
6000
PC1 (51.00%)
PC2 (38.48%)

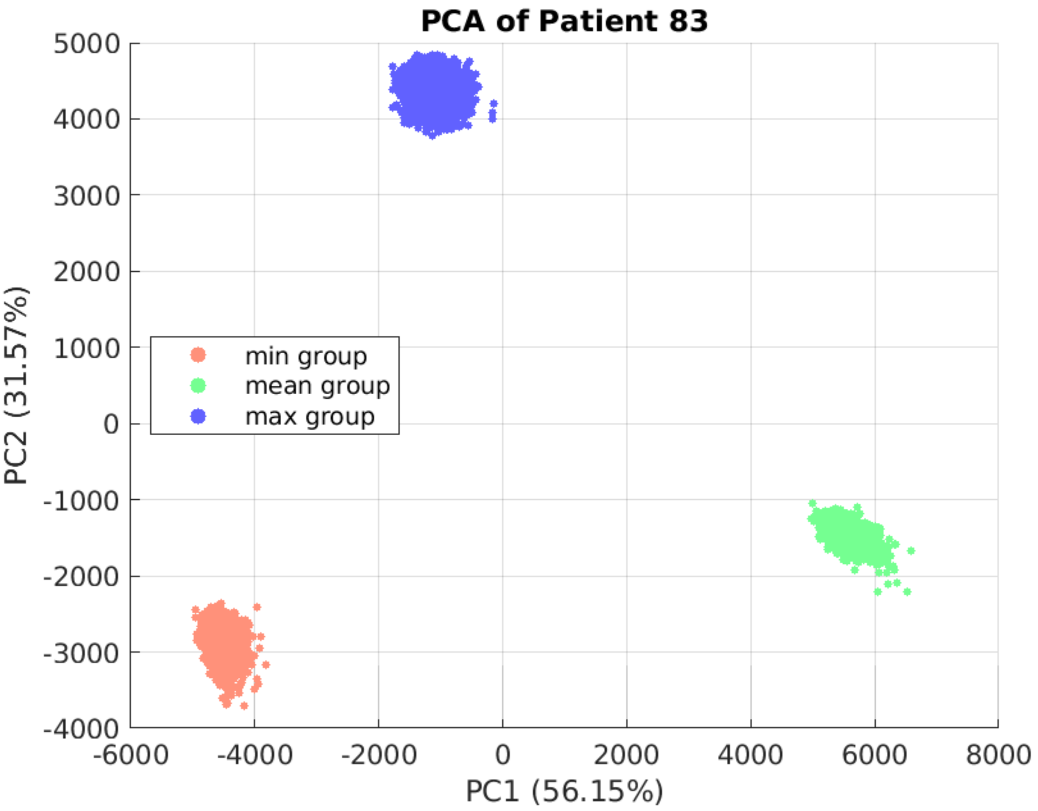
PCA of Patient 83
5000
4000
3000
2000
1000
0
-1000
-2000
-3000
-4000
-6000
-4000
-2000
0
2000
4000
6000
8000
PC1 (56.15%)
PC2 (31.57%)
min group
mean group
max group

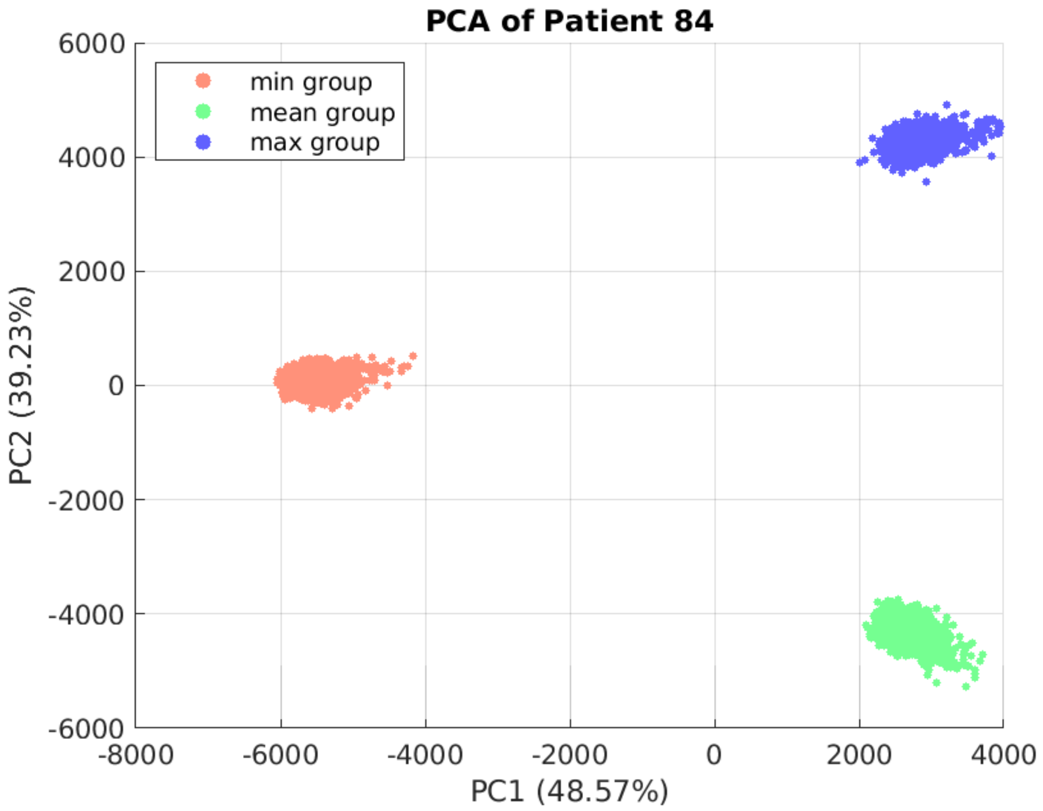
PCA of Patient 84
min group
mean group
max group
PC1 (48.57%)
PC2 (39.23%)
6000
4000
2000
0
-2000
-4000
-6000
-8000
-6000
-4000
-2000
0
2000
4000

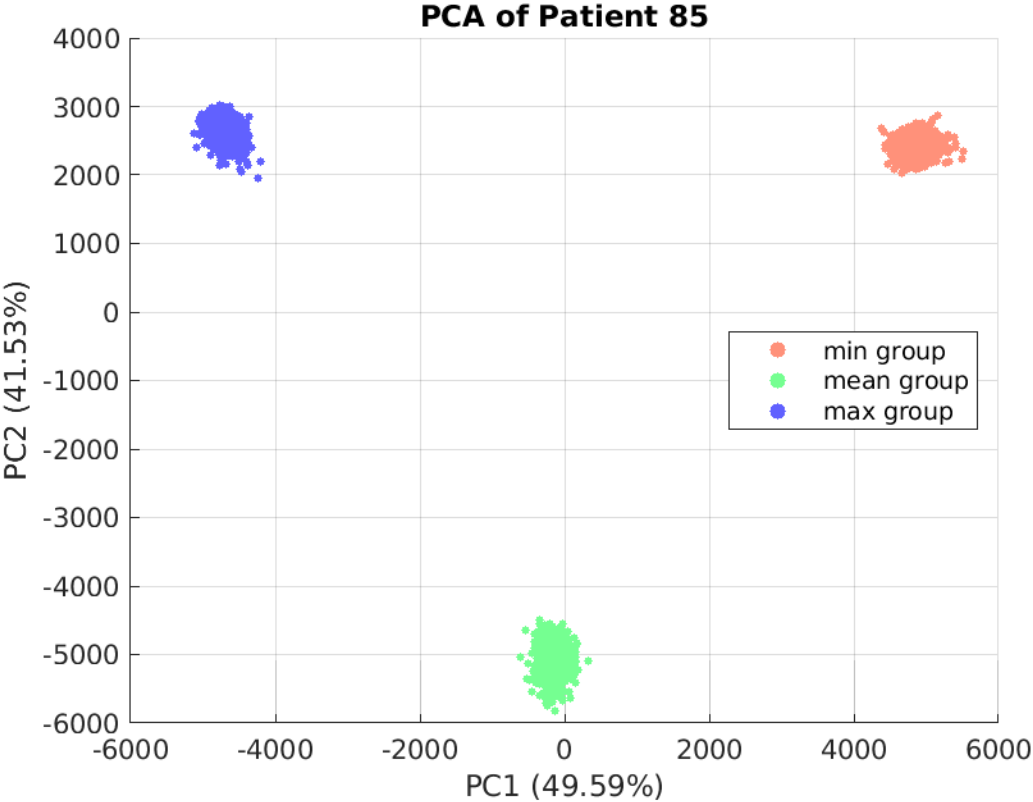
PCA of Patient 85
PC2 (41.53%)
PC1 (49.59%)
4000
3000
2000
1000
0
-1000
-2000
-3000
-4000
-5000
-6000
-6000
-4000
-2000
0
2000
4000
6000
min group
mean group
max group

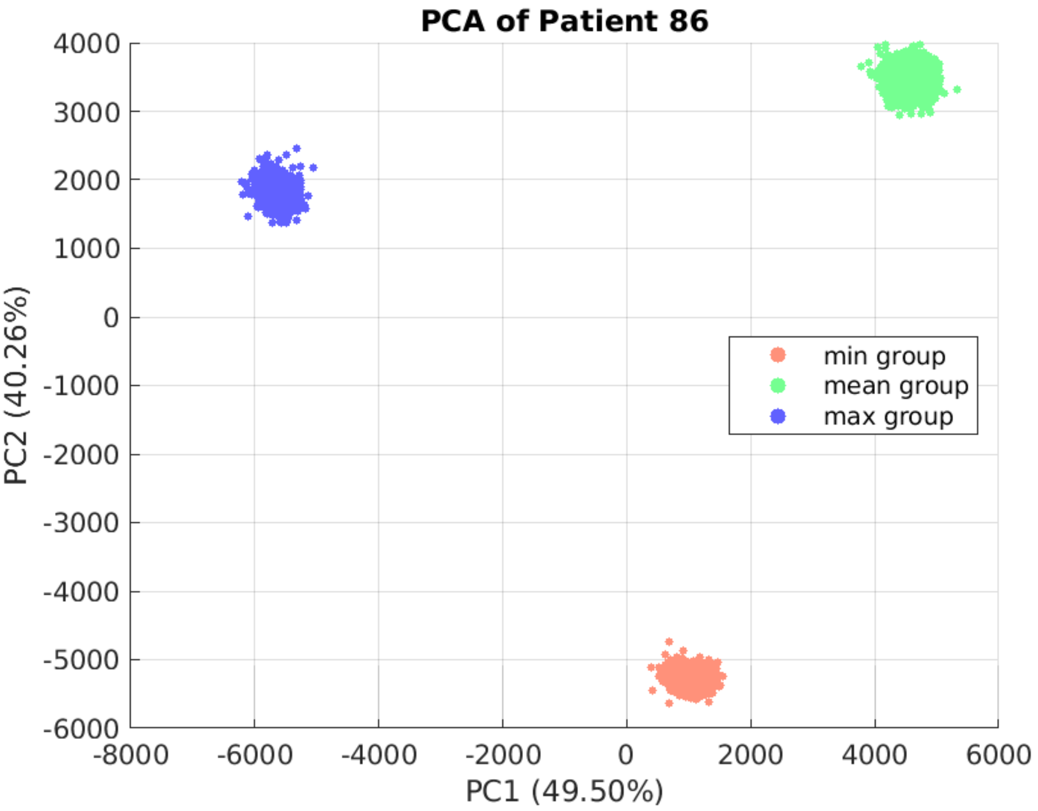
PCA of Patient 86
PC1 (49.50%)
PC2 (40.26%)
min group
mean group
max group
-8000
-6000
-4000
-2000
0
2000
4000
6000
4000
3000
2000
1000
0
-1000
-2000
-3000
-4000
-5000
-6000

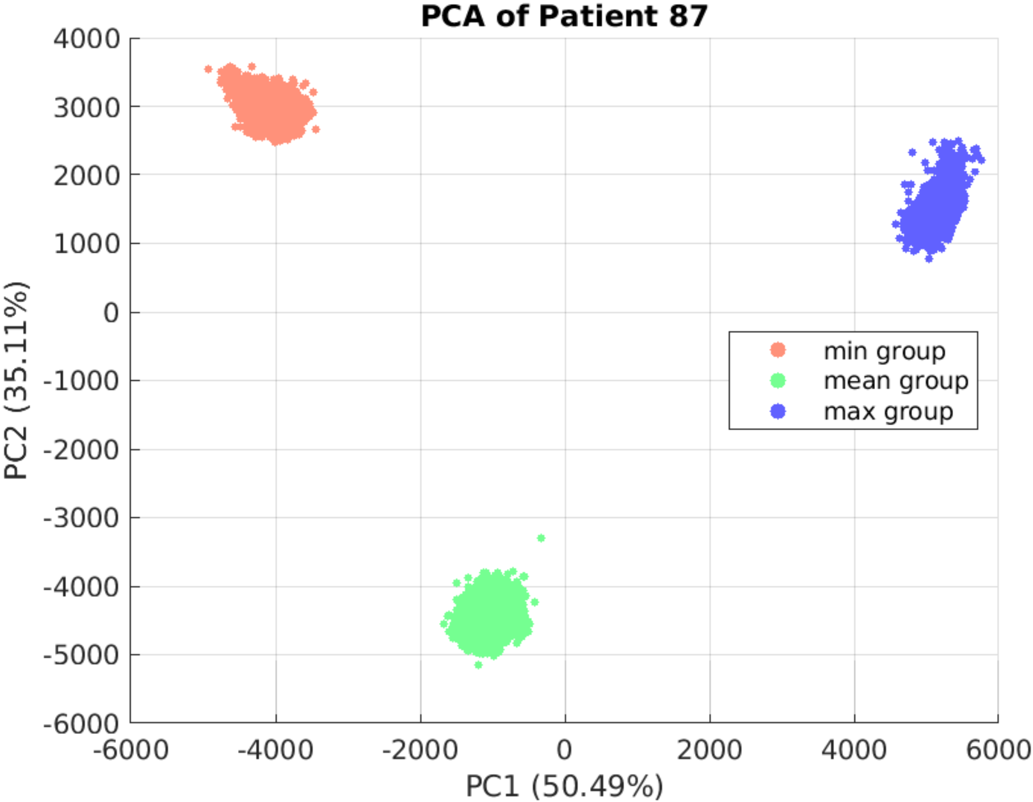
PCA of Patient 87
PC2 (35.11%)
PC1 (50.49%)
4000
3000
2000
1000
0
-1000
-2000
-3000
-4000
-5000
-6000
-6000
-4000
-2000
0
2000
4000
6000
min group
mean group
max group

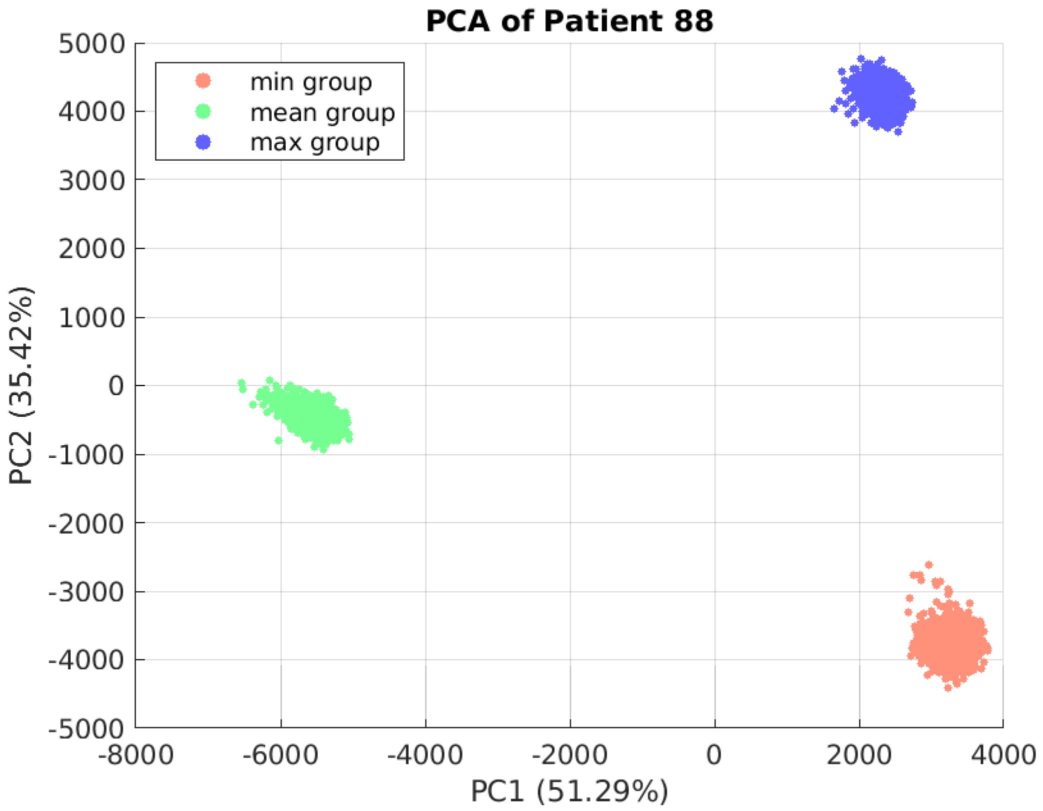
PCA of Patient 88
min group
mean group
max group
5000
4000
3000
2000
1000
0
-1000
-2000
-3000
-4000
-5000
PC2 (35.42%)
-8000
-6000
-4000
-2000
0
2000
4000
PC1 (51.29%)

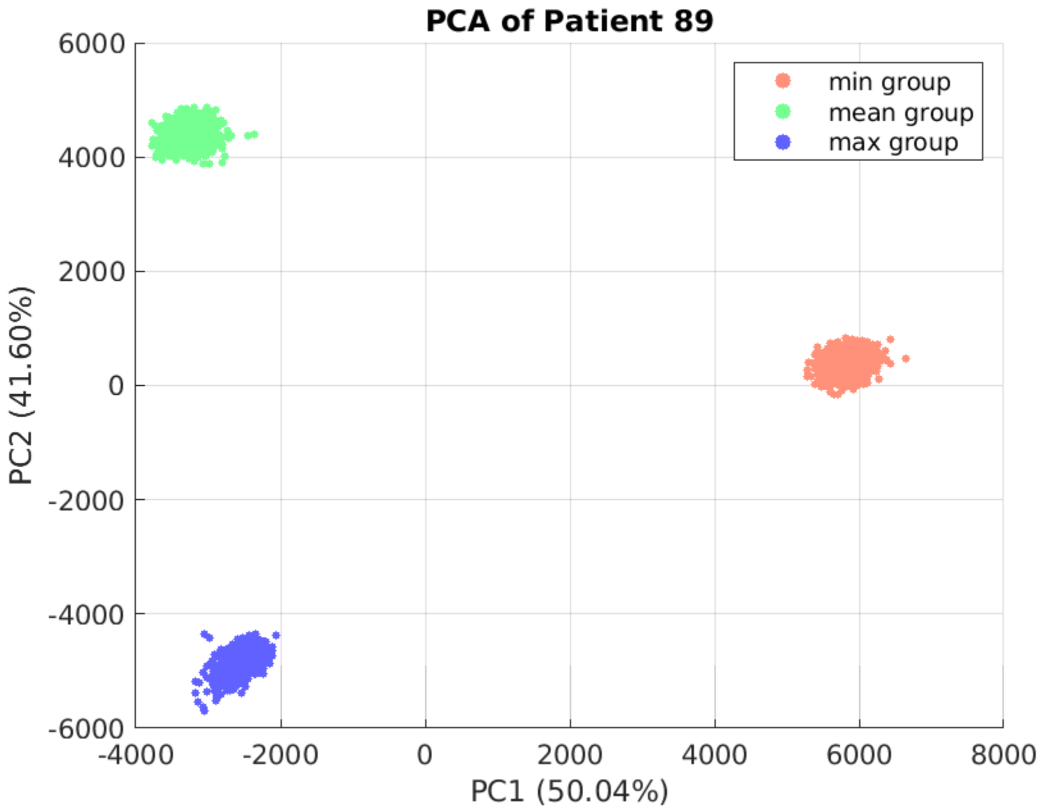
PCA of Patient 89
min group
mean group
max group
6000
4000
2000
0
-2000
-4000
-6000
-4000
-2000
0
2000
4000
6000
8000
PC1 (50.04%)
PC2 (41.60%)

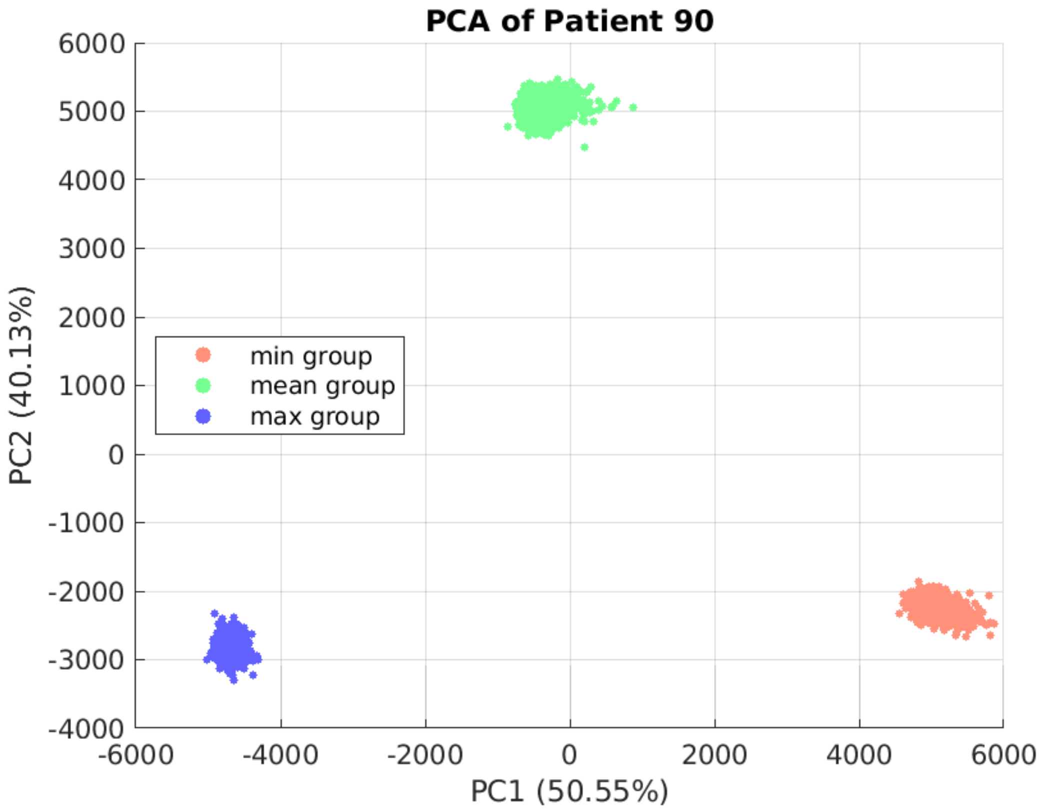
PCA of Patient 90
PC2 (40.13%)
PC1 (50.55%)
min group
mean group
max group
6000
5000
4000
3000
2000
1000
0
-1000
-2000
-3000
-4000
-6000
-4000
-2000
0
2000
4000
6000

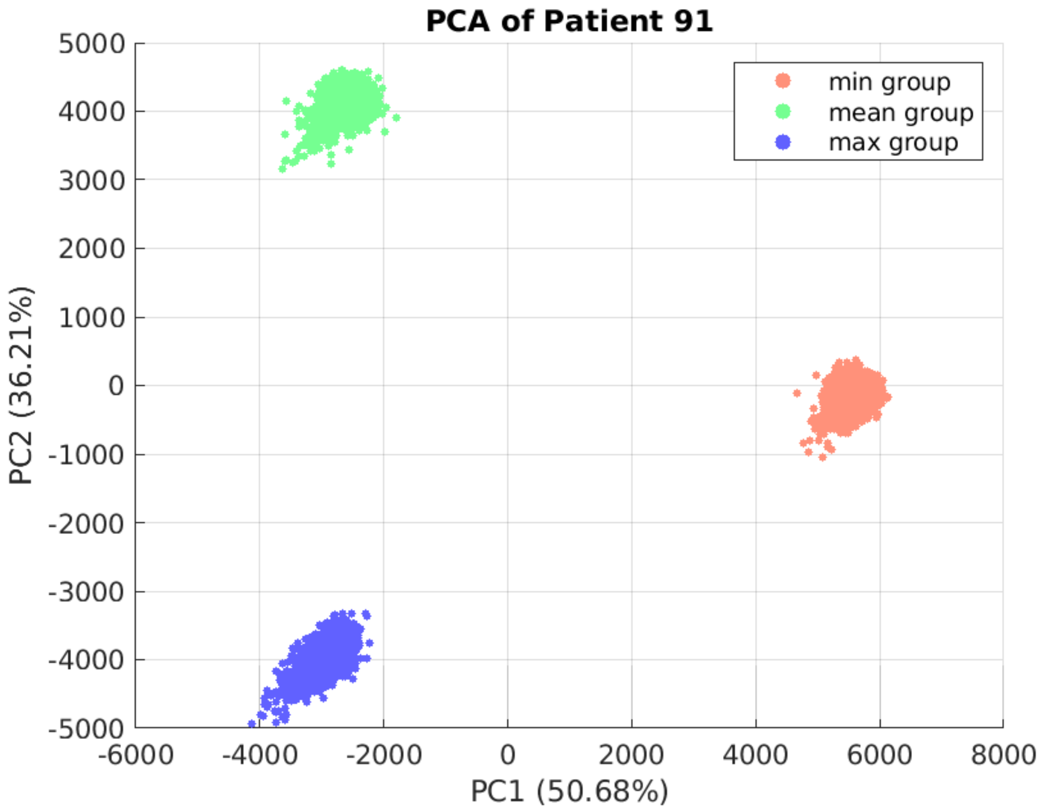
PCA of Patient 91
min group
mean group
max group
5000
4000
3000
2000
1000
0
-1000
-2000
-3000
-4000
-5000
-6000
-4000
-2000
0
2000
4000
6000
8000
PC2 (36.21%)
PC1 (50.68%)

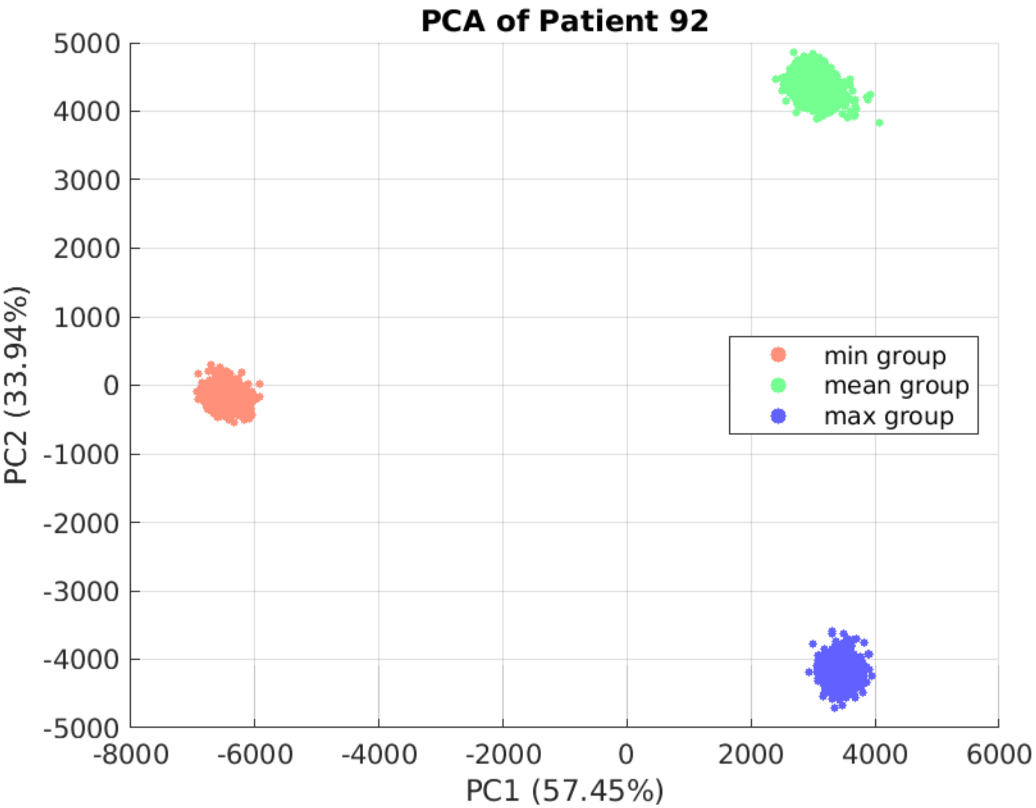
PCA of Patient 92
PC2 (33.94%)
PC1 (57.45%)
min group
mean group
max group
5000
4000
3000
2000
1000
0
-1000
-2000
-3000
-4000
-5000
-8000
-6000
-4000
-2000
0
2000
4000
6000

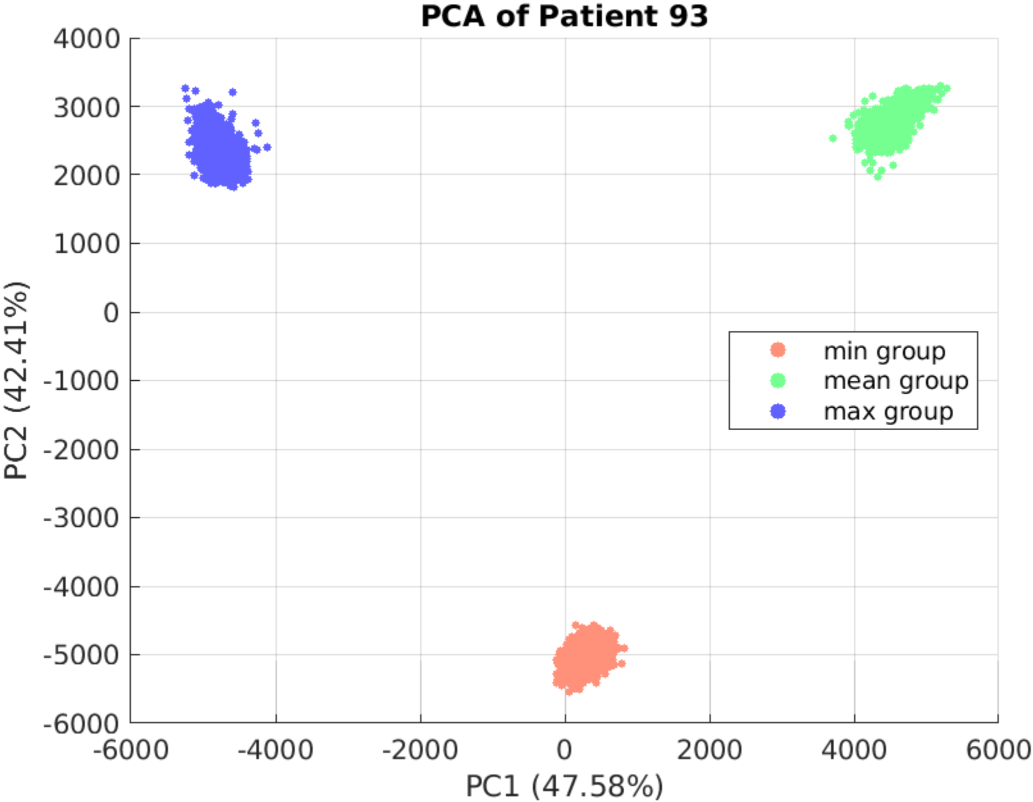

PCA of Patient 93
PC2 (42.41%)
PC1 (47.58%)
4000
3000
2000
1000
0
-1000
-2000
-3000
-4000
-5000
-6000
-6000
-4000
-2000
0
2000
4000
6000
min group
mean group
max group

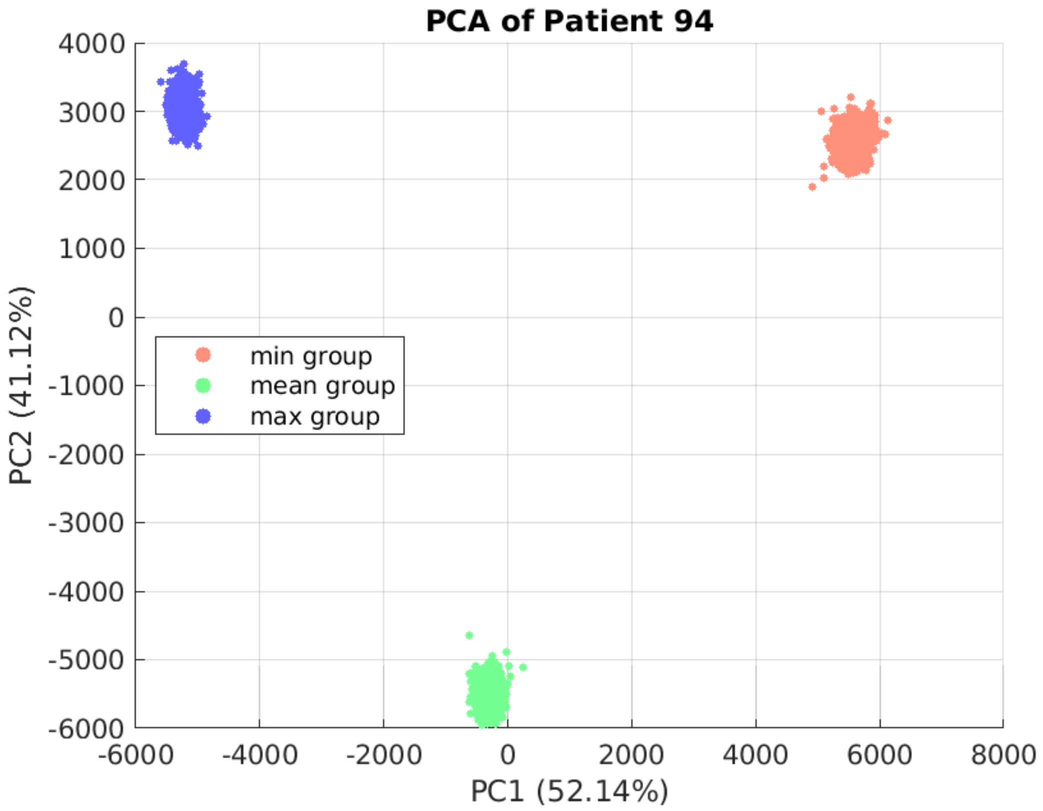
PCA of Patient 94
PC2 (41.12%)
PC1 (52.14%)
4000
3000
2000
1000
0
-1000
-2000
-3000
-4000
-5000
-6000
-6000
-4000
-2000
0
2000
4000
6000
8000
min group
mean group
max group

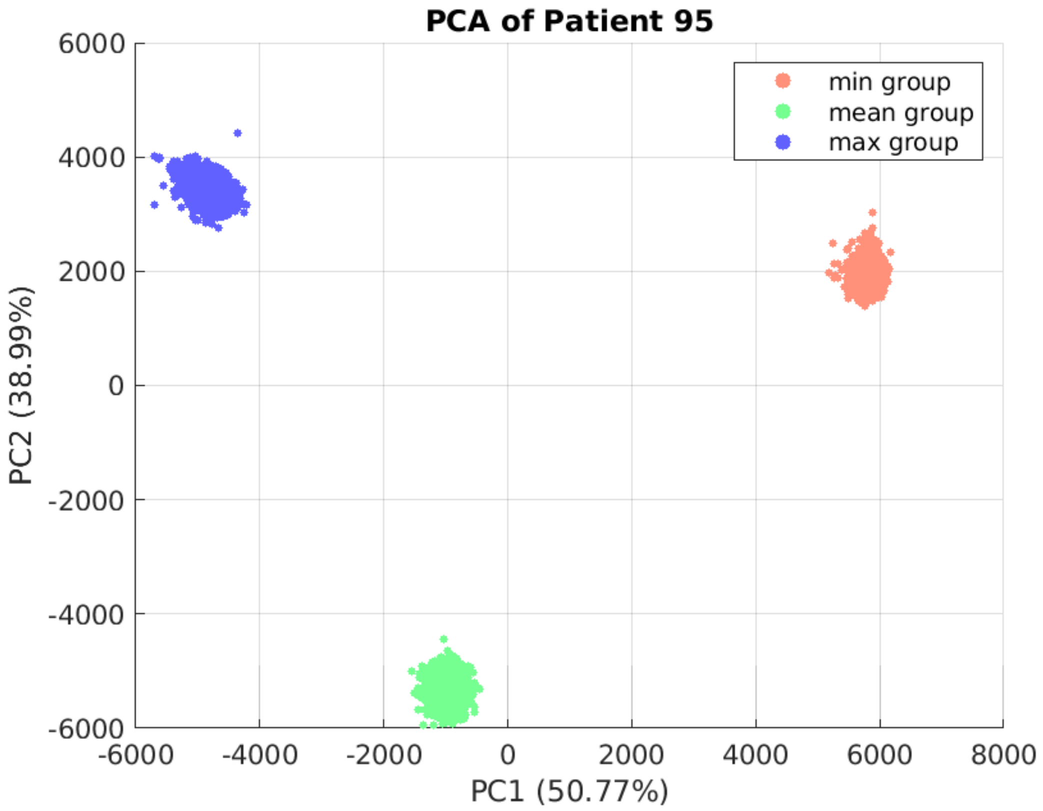
PCA of Patient 95
min group
mean group
max group
PC1 (50.77%)
PC2 (38.99%)
6000
4000
2000
0
-2000
-4000
-6000
-6000
-4000
-2000
0
2000
4000
6000
8000
