# Supplementary material for: Integrating Genome-Scale Metabolic Models with Patient Plasma Metabolome to Study Endothelial Metabolism In Situ
Source: Int J Mol Sci. 2024 May 15;25(10):5406. doi: 10.3390/ijms25105406 (PMC11121795; doi:10.3390/ijms25105406)
Supplement: Supplementary file 1 [file ijms-25-05406-s001.zip › Silva-Lance et al-Supplementary Material 4.zip!/Silva-Lance et al - Supplementary Material 4.pdf]

Supplementary Material 5: Clustering analisys plots

- Clustering analysis of the whole dataset

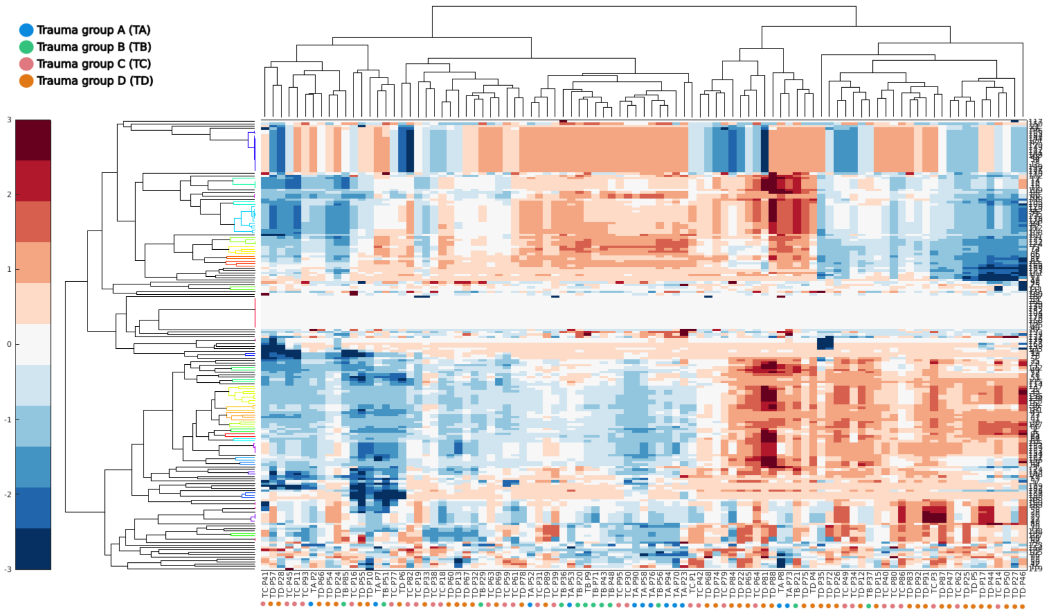

Figure S1: Heat map and dendrogram of the 190 metabolic tasks and 95 trauma patients. Data standardized by column

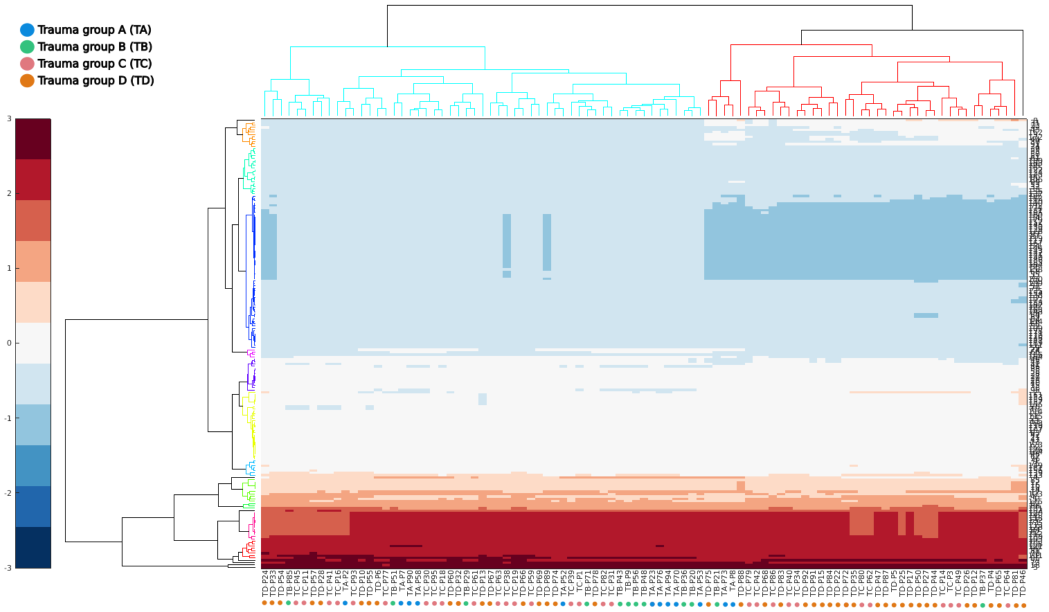

Figure S2: Heat map and dendrogram of the 190 metabolic tasks and 95 trauma patients. Data standardized by row

- Clustering analysis of Trauma group A

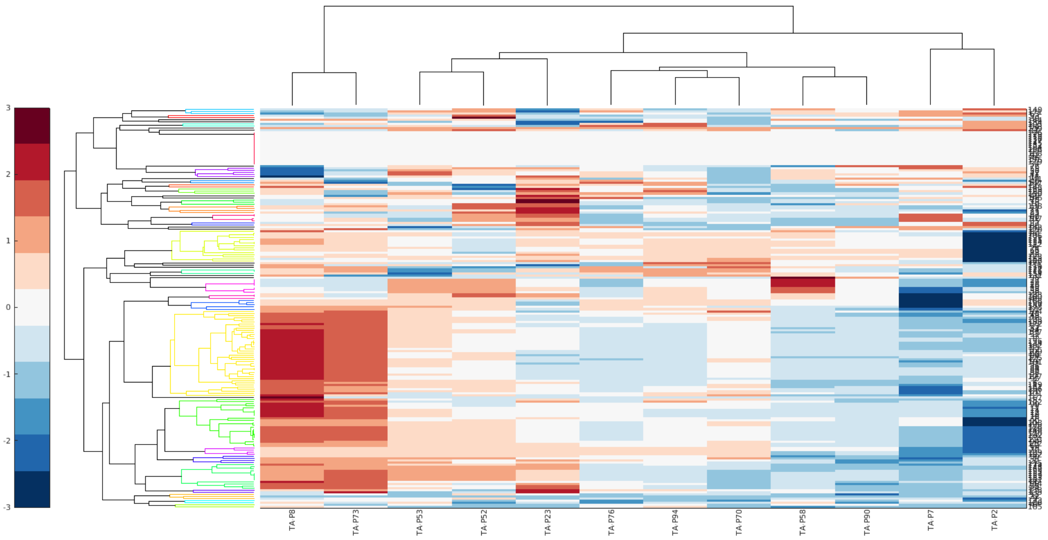

Figure S3: Heat map and dendrogram of the 190 metabolic tasks and trauma group A. Data standardized by column

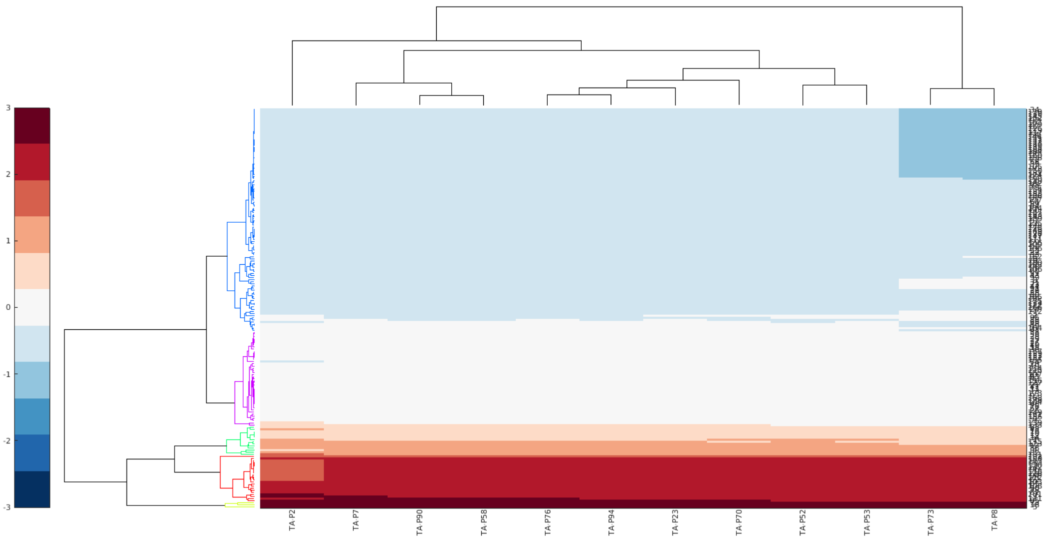

Figure S4: Heat map and dendrogram of the 190 metabolic tasks and trauma group A. Data standardized by row

- Clustering analysis of Trauma group B

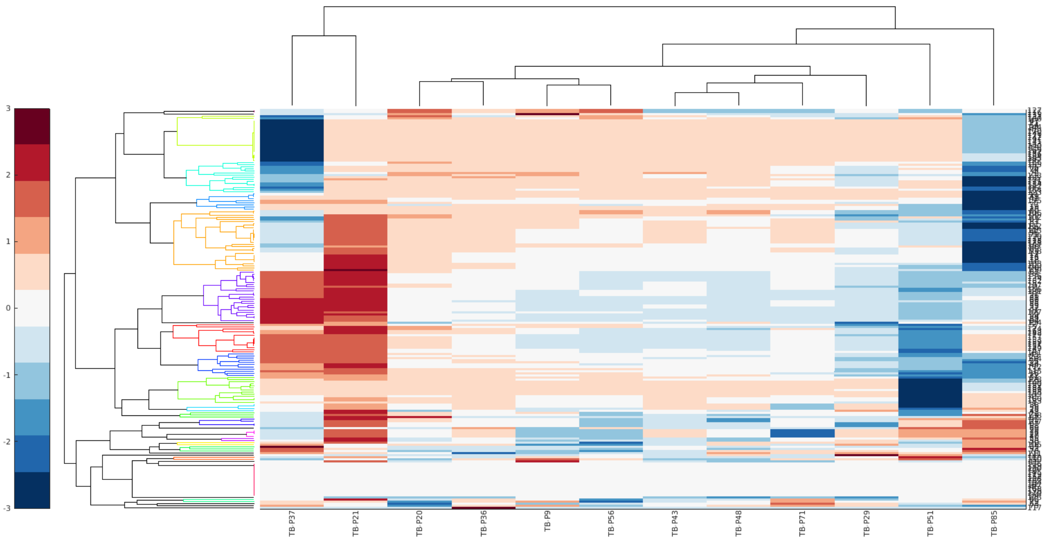

Figure S5: Heat map and dendrogram of the 190 metabolic tasks and trauma group B. Data standardized by column

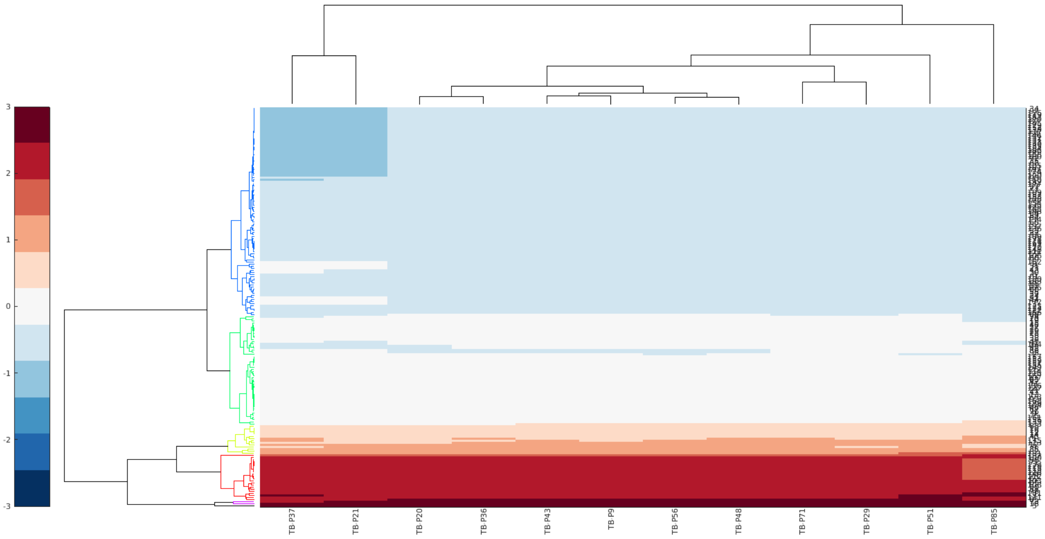

Figure S6: Heat map and dendrogram of the 190 metabolic tasks and trauma group B. Data standardized by row

- Clustering analysis of Trauma group C

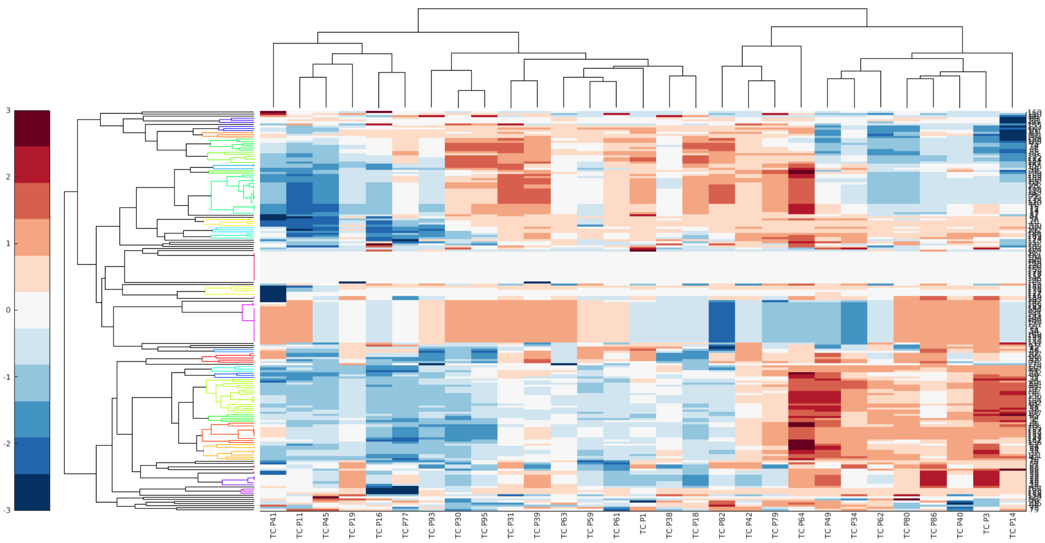

Figure S7: Heat map and dendrogram of the 190 metabolic tasks and trauma group C. Data standardized by column

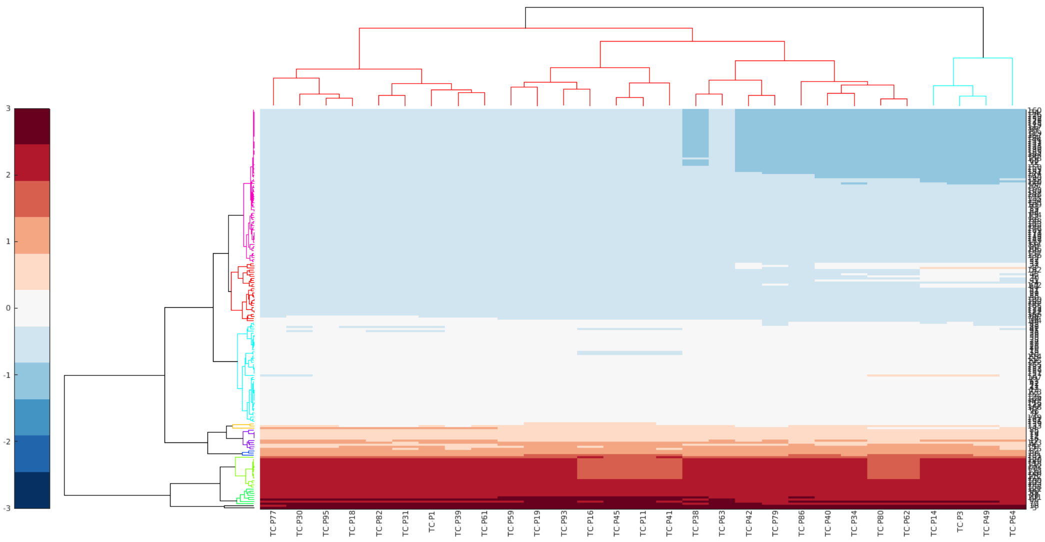

Figure S8: Heat map and dendrogram of the 190 metabolic tasks and trauma group C. Data standardized by row

- Clustering analysis of Trauma group D

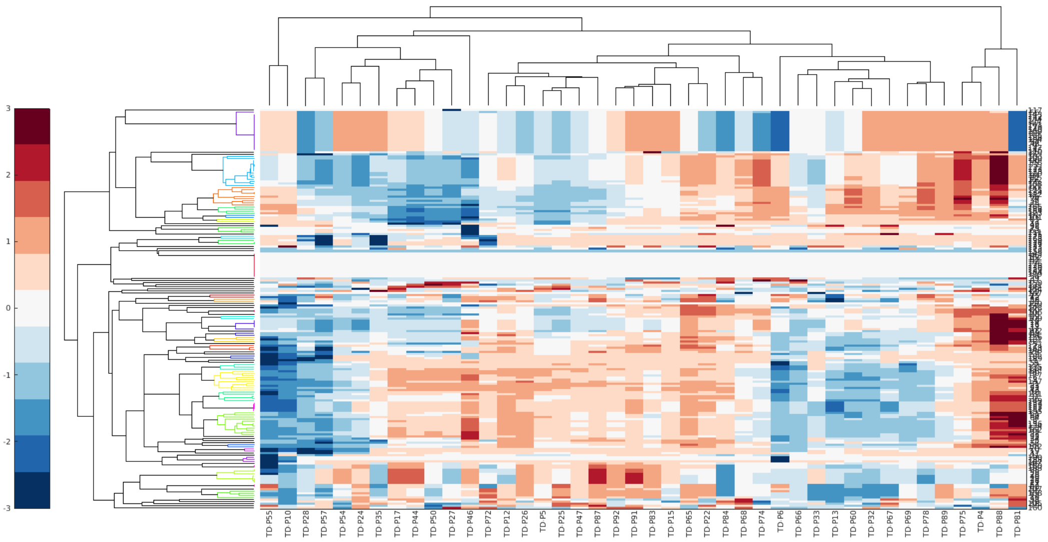

Figure S9: Heat map and dendrogram of the 190 metabolic tasks and trauma group D. Data standardized by column

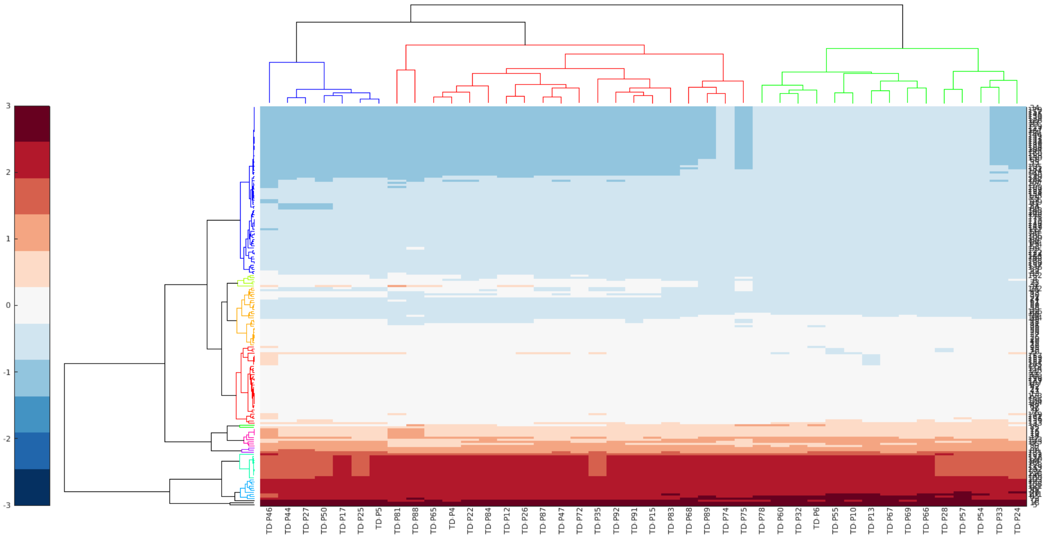

Figure S10: Heat map and dendrogram of the 190 metabolic tasks and trauma group D. Data standardized by row

- Clustering analysis of all patients and relevant tasks

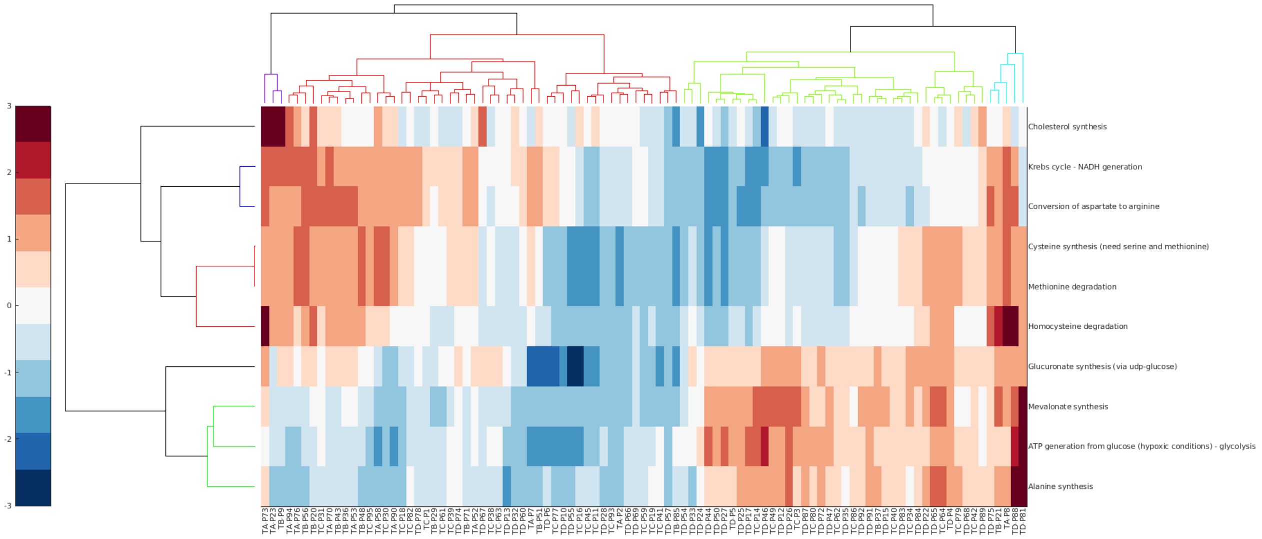

Figure S11: Heat map and dendrogram of 10 relevant metabolic tasks and 95 trauma patients. Data standardized by column
