# Supplementary material for: Integrating Genome-Scale Metabolic Models with Patient Plasma Metabolome to Study Endothelial Metabolism In Situ
Source: Int J Mol Sci. 2024 May 15;25(10):5406. doi: 10.3390/ijms25105406 (PMC11121795; doi:10.3390/ijms25105406)
Supplement: Supplementary file 1 [file ijms-25-05406-s001.zip › Silva-Lance et al-Supplementary Material 5.zip!/Sens_Ans_All_Patients.pdf]

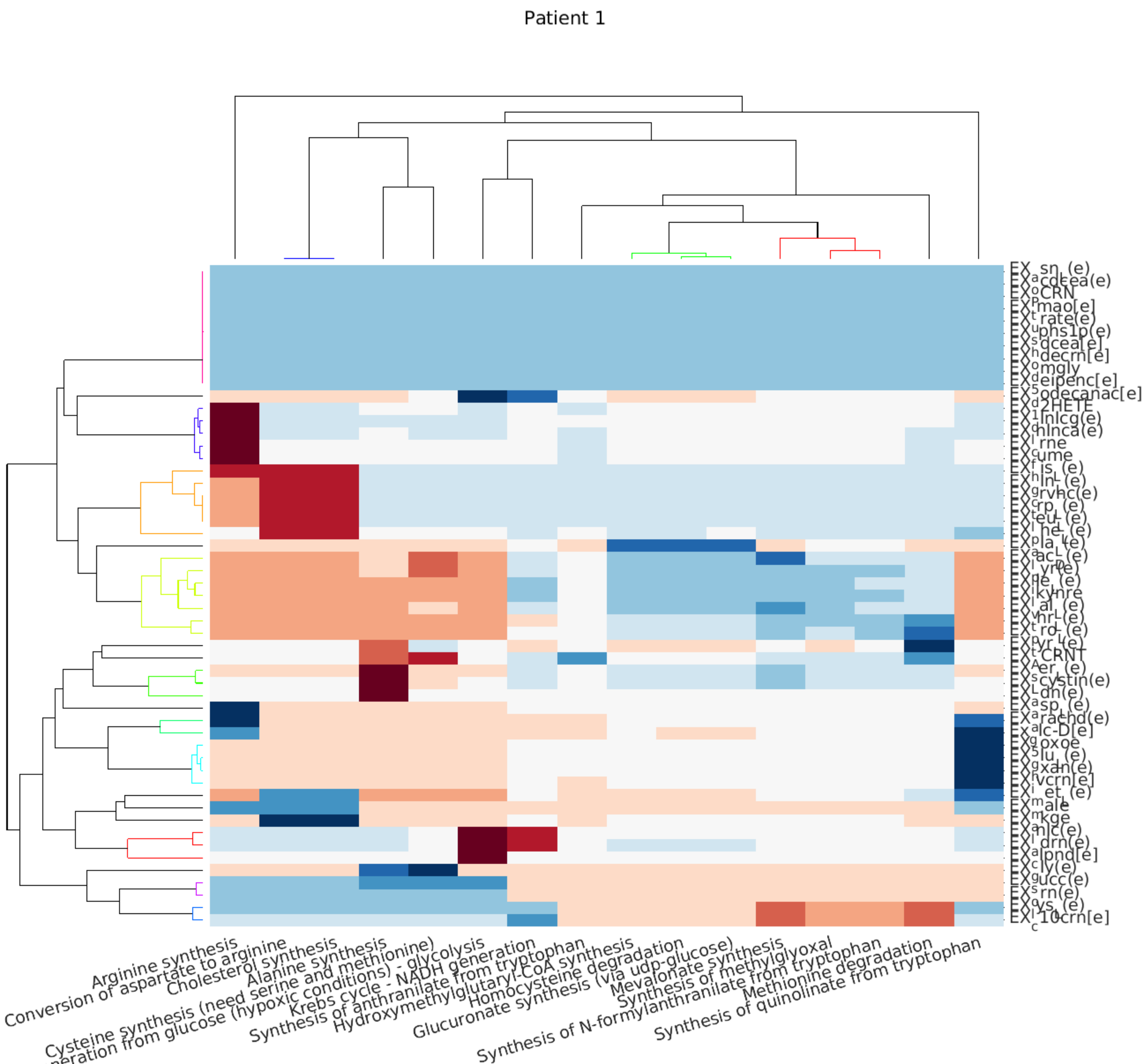

Patient 1
Arginine synthesis
Conversion of aspartate to arginine
Cholesterol synthesis
Alanine synthesis
Cysteine synthesis (need serine and methionine)
Krebs cycle - NADH generation
Synthesis of anthranilate from tryptophan
Hydroxymethylglutaryl-CoA synthesis
Homocysteine degradation
Glucuronate synthesis (via udp-glucose)
Mevalonate synthesis
Synthesis of methylglyoxal
Synthesis of N-formylanthranilate from tryptophan
Methionine degradation
Synthesis of quinolinate from tryptophan

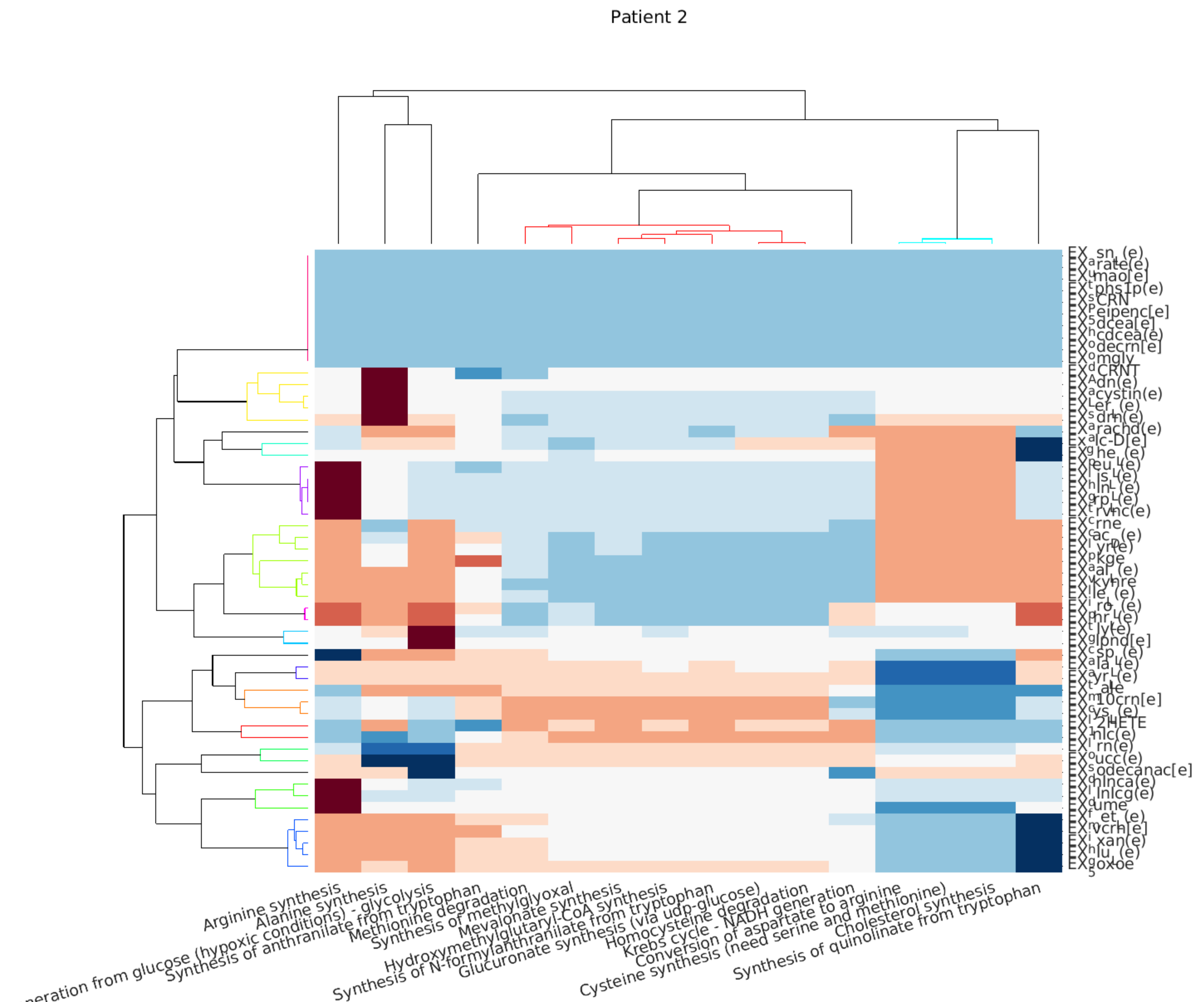
Patient 2
Arginine synthesis
Alanine synthesis
neration from glucose (hypoxic conditions) - glycolysis
Synthesis of anthranilate from tryptophan
Methionine degradation
Synthesis of methylglyoxal
Hydroxymethylglutaryl-CoA synthesis
Mevalonate synthesis
Synthesis of N-formylanthranilate from tryptophan
Glucuronate synthesis (via udp-glucose)
Homocysteine degradation
Krebs cycle - NADH generation
Cysteine synthesis (need serine and methionine)
Conversion of aspartate to arginine
Cholesterol synthesis
Synthesis of quinolinate from tryptophan

Patient 3

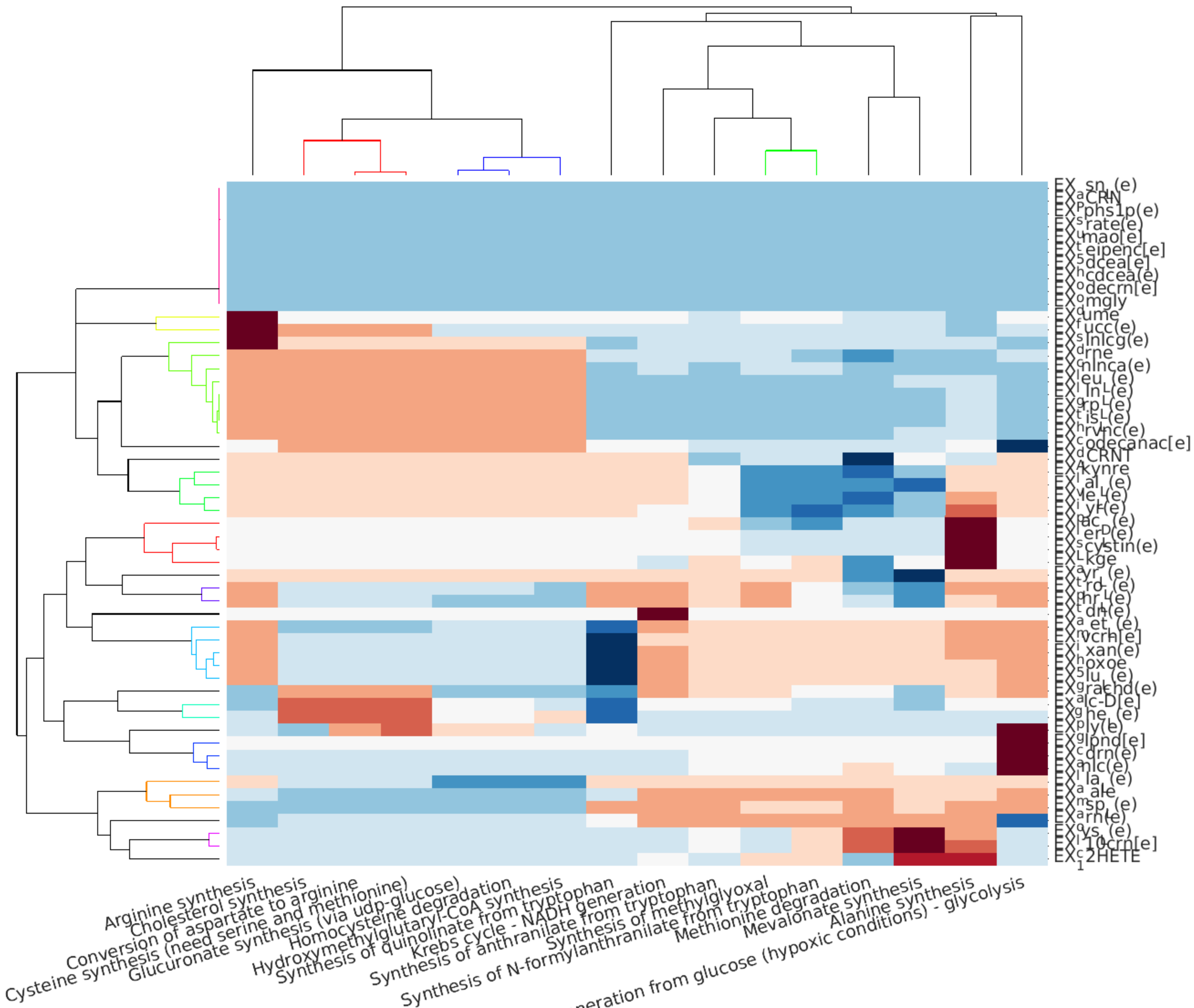

Arginine synthesis
Cholesterol synthesis
Conversion of aspartate to arginine
Cysteine synthesis (need serine and methionine)
Glucuronate synthesis (via udp-glucose)
Homocysteine degradation
Hydroxymethylglutaryl-CoA synthesis
Synthesis of quinolinate from tryptophan
Krebs cycle - NADH generation
Synthesis of anthranilate from tryptophan
Synthesis of methylglyoxal
Synthesis of N-formylanthranilate from tryptophan
Methionine degradation
Mevalonate synthesis
Alanine synthesis
neration from glucose (hypoxic conditions) - glycolysis

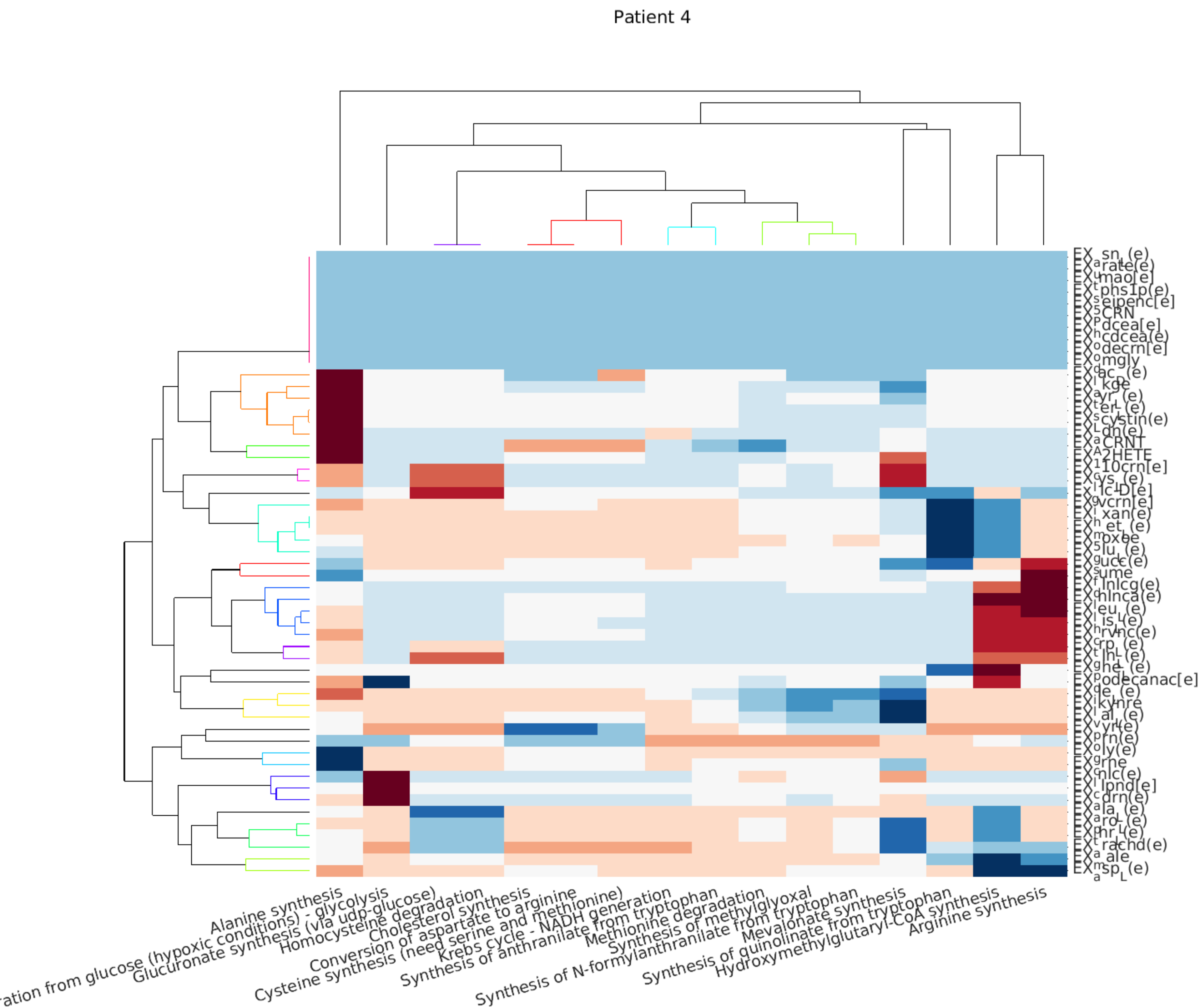
Patient 4
Alanine synthesis
ation from glucose (hypoxic conditions) - glycolysis
Glucuronate synthesis (via udp-glucose)
Homocysteine degradation
Cholesterol synthesis
Conversion of aspartate to arginine
Cysteine synthesis (need serine and methionine)
Krebs cycle - NADH generation
Synthesis of anthranilate from tryptophan
Methionine degradation
Synthesis of methylglyoxal
Synthesis of N-formylanthranilate from tryptophan
Mevalonate synthesis
Synthesis of quinolinate from tryptophan
Hydroxymethylglutaryl-CoA synthesis
Arginine synthesis

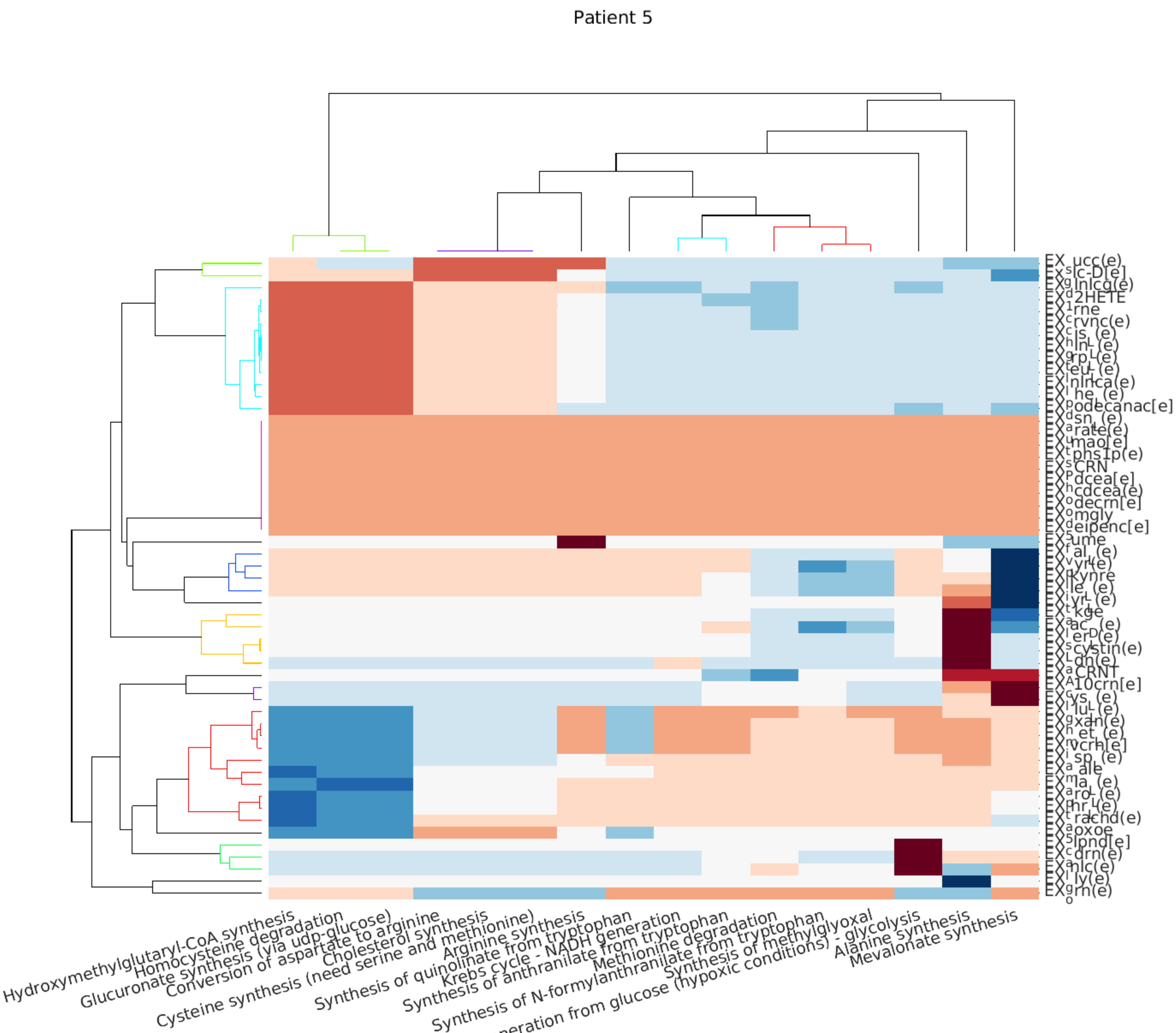
Patient 5
Hydroxymethylglutaryl-CoA synthesis
Homocysteine degradation
Glucuronate synthesis (via udp-glucose)
Conversion of aspartate to arginine
Cholesterol synthesis
Cysteine synthesis (need serine and methionine)
Arginine synthesis
Synthesis of quinolinate from tryptophan
Krebs cycle - NADH generation
Synthesis of anthranilate from tryptophan
Methionine degradation
Synthesis of N-formylanthranilate from tryptophan
Synthesis of methylglyoxal
from glucose (hypoxic conditions) - glycolysis
Alanine synthesis
Mevalonate synthesis

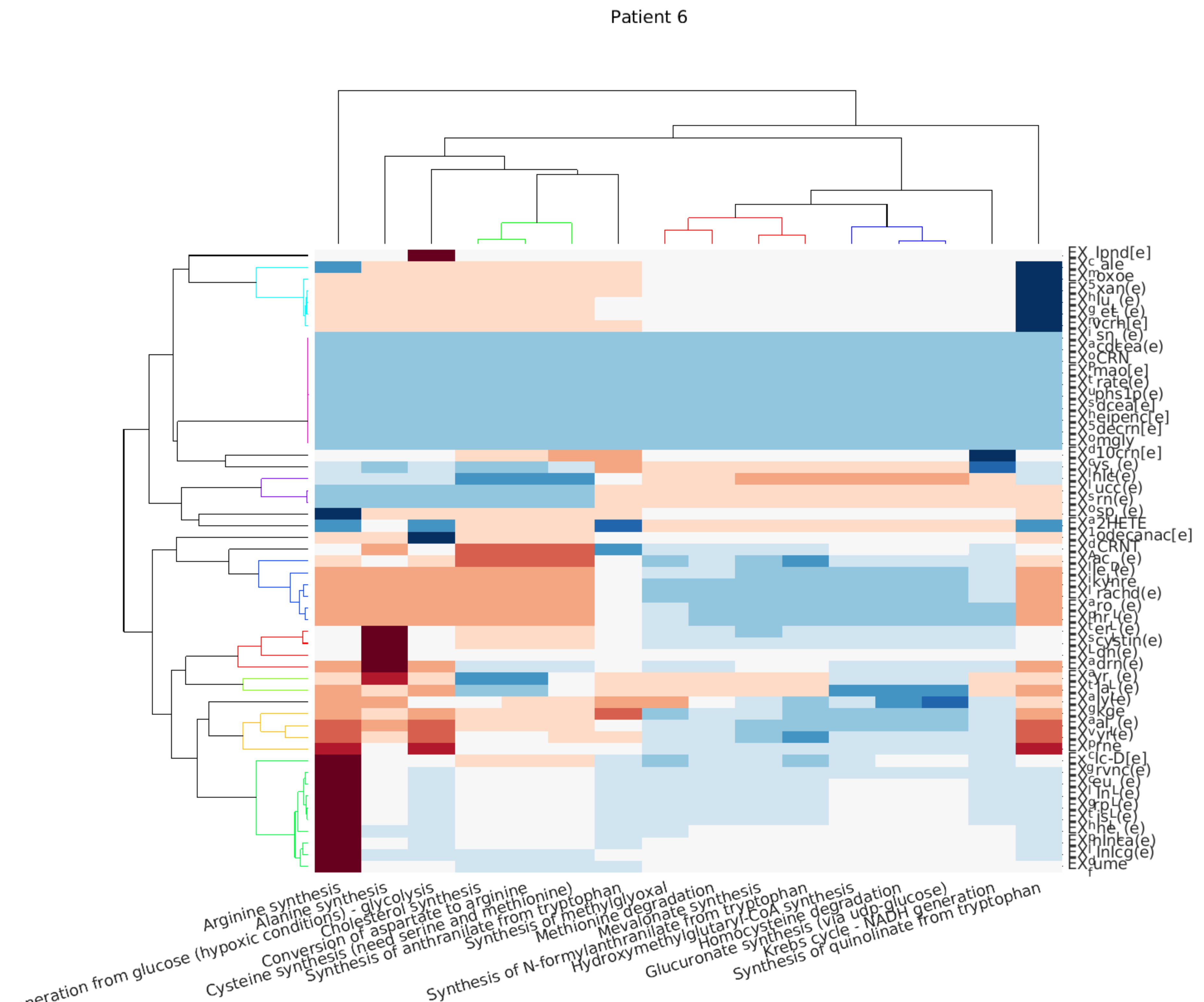

Patient 6
Arginine synthesis
Alanine synthesis
Cholesterol synthesis
Conversion of aspartate to arginine
Cysteine synthesis (need serine and methionine)
Synthesis of anthranilate from tryptophan
Synthesis of methylglyoxal
Methionine degradation
Mevalonate synthesis
Synthesis of N-formylanthranilate from tryptophan
Hydroxymethylglutaryl-CoA synthesis
Homocysteine degradation
Glucuronate synthesis (via udp-glucose)
Krebs cycle - NADH generation
Synthesis of quinolinate from tryptophan

Patient 7

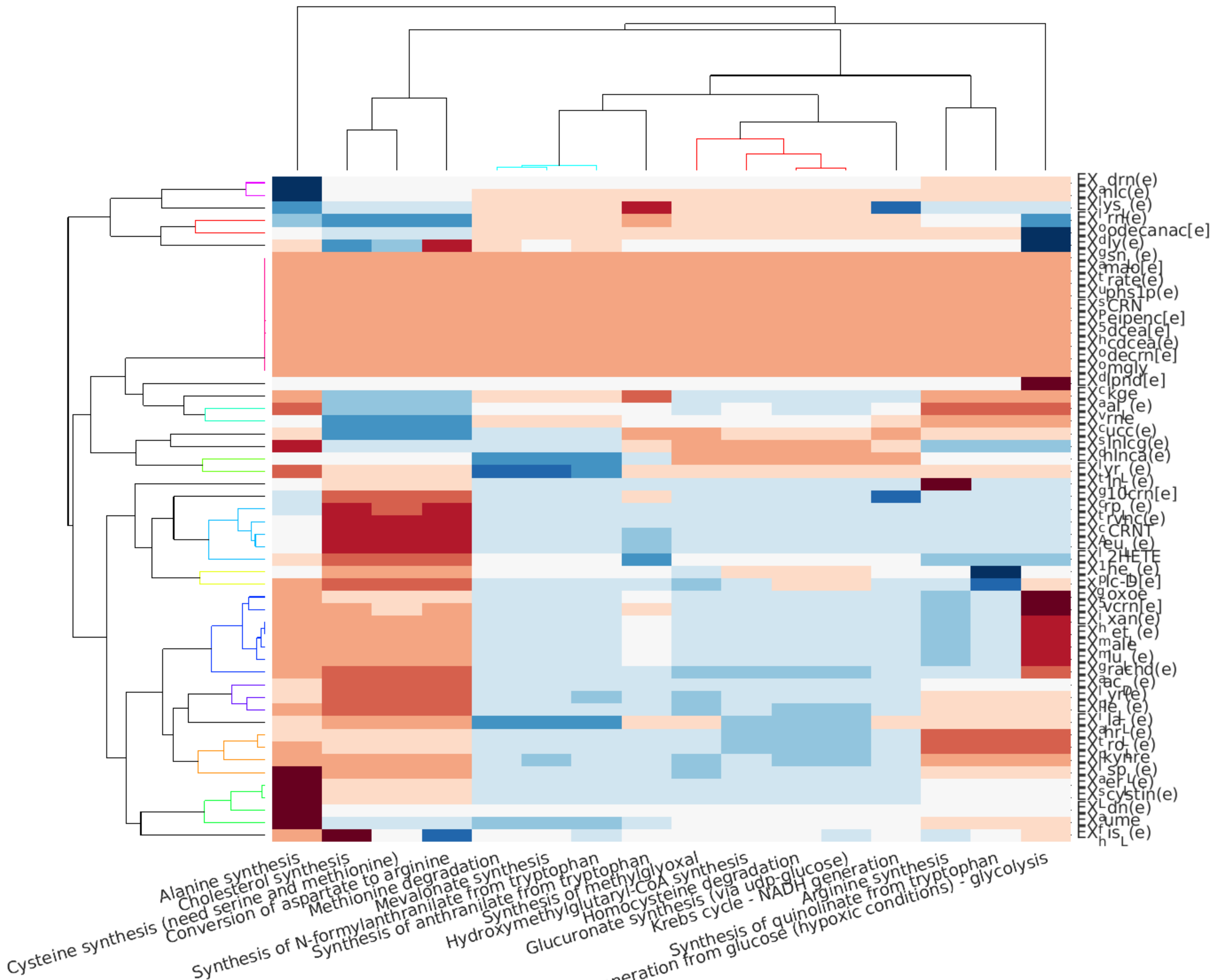
Alanine synthesis
Cholesterol synthesis
Cysteine synthesis (need serine and methionine)
Conversion of aspartate to arginine
Methionine degradation
Mevalonate synthesis
Synthesis of N-formylanthranilate from tryptophan
Synthesis of anthranilate from tryptophan
Synthesis of methylglyoxal
Hydroxymethylglutaryl-CoA synthesis
Homocysteine degradation
Glucuronate synthesis (via udp-glucose)
Krebs cycle - NADH generation
Arginine synthesis
Synthesis of quinolinate from tryptophan

Patient 8

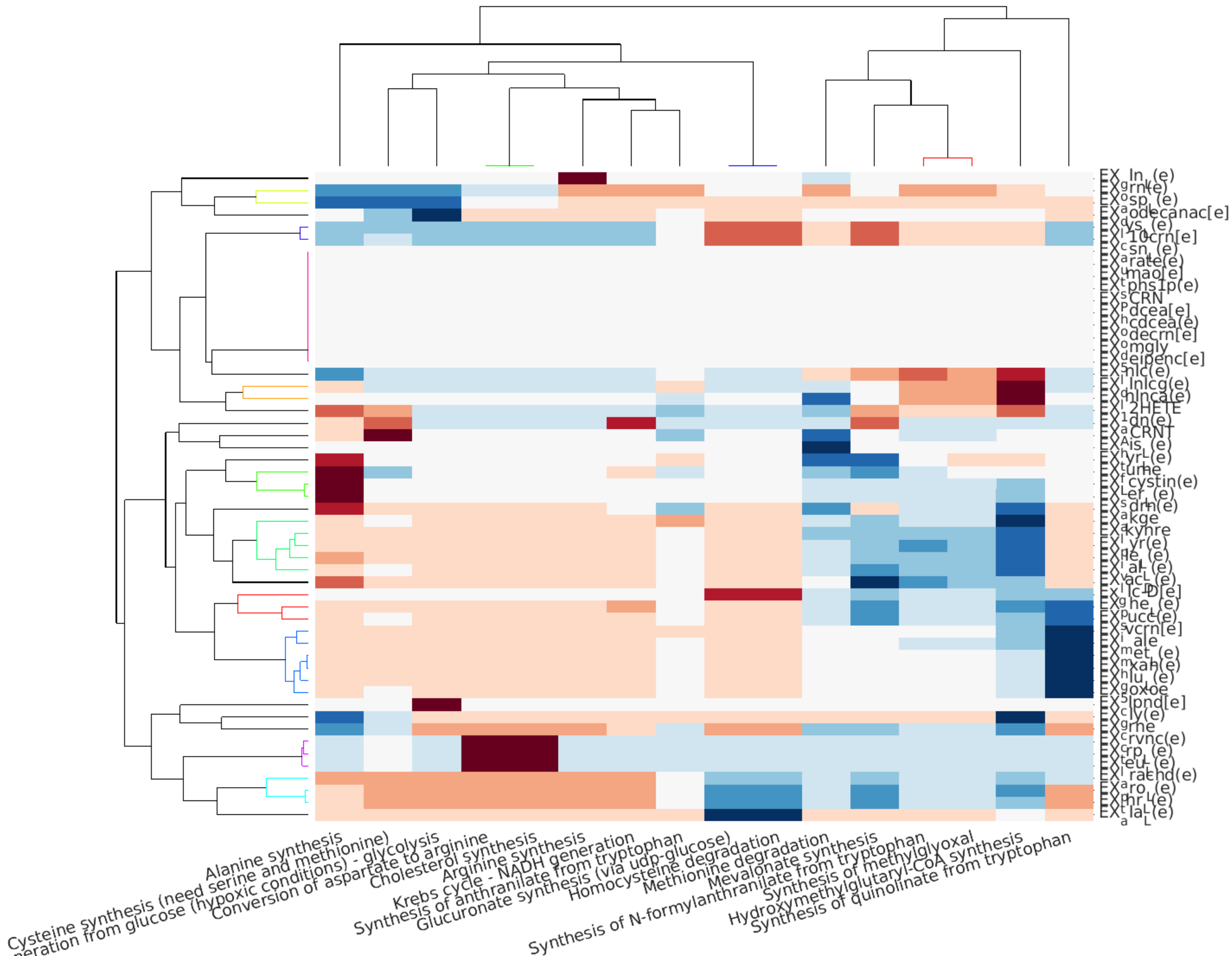
Alanine synthesis
Cysteine synthesis (need serine and methionine)
Conversion of aspartate to arginine
Cholesterol synthesis
Arginine synthesis
Krebs cycle - NADH generation
Synthesis of anthranilate from tryptophan
Glucuronate synthesis (via udp-glucose)
Homocysteine degradation
Methionine degradation
Mevalonate synthesis
Synthesis of N-formylanthranilate from tryptophan
Synthesis of methylglyoxal
Hydroxymethylglutaryl-CoA synthesis
Synthesis of quinolinate from tryptophan

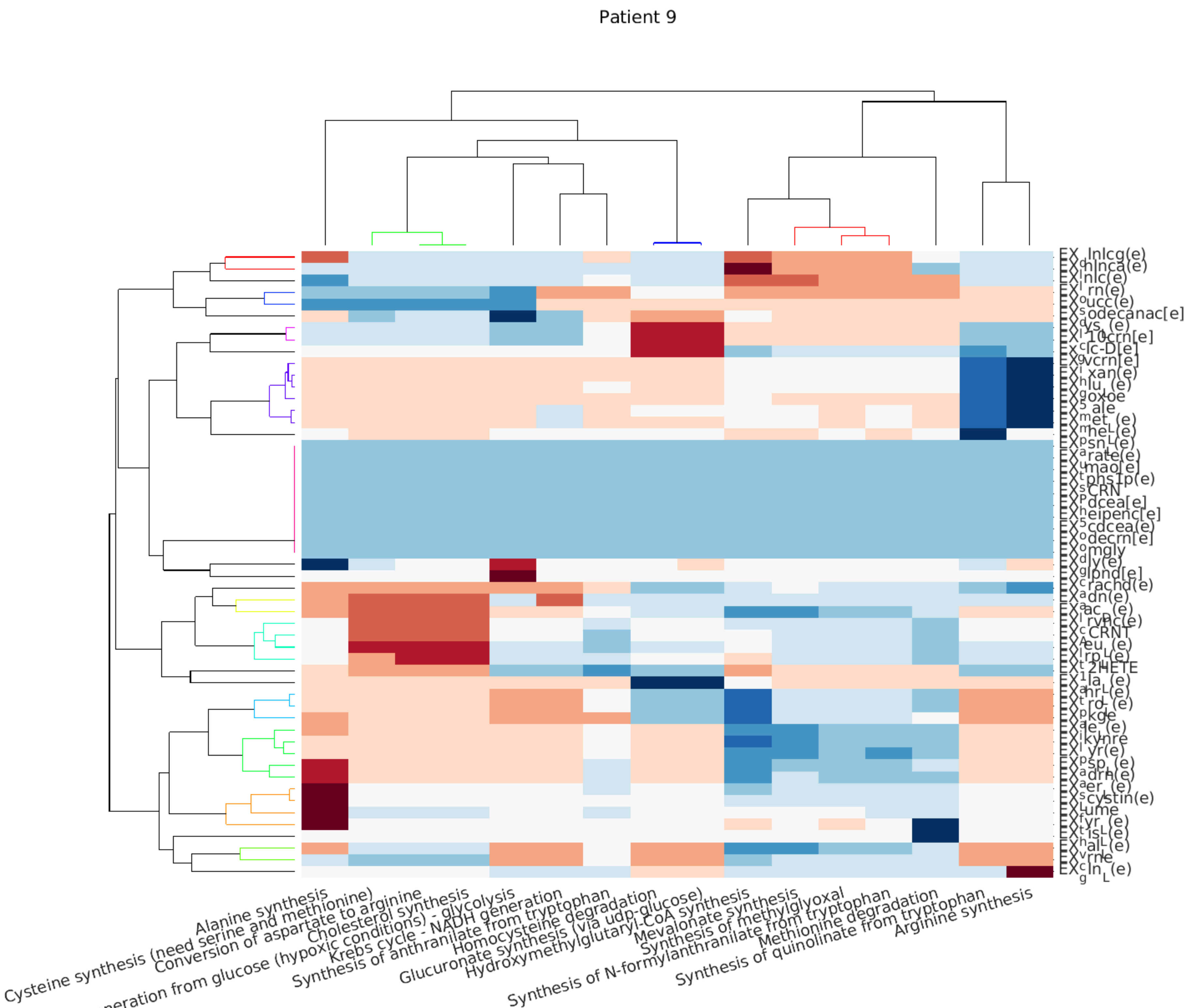
Patient 9
Alanine synthesis
Cysteine synthesis (need serine and methionine)
Conversion of aspartate to arginine
Cholesterol synthesis
neration from glucose (hypoxic conditions) - glycolysis
Krebs cycle - NADH generation
Synthesis of anthranilate from tryptophan
Homocysteine degradation
Glucuronate synthesis (via udp-glucose)
Hydroxymethylglutaryl-CoA synthesis
Mevalonate synthesis
Synthesis of N-formylanthranilate from tryptophan
Synthesis of methylglyoxal
Methionine degradation
Synthesis of quinolinate from tryptophan
Arginine synthesis

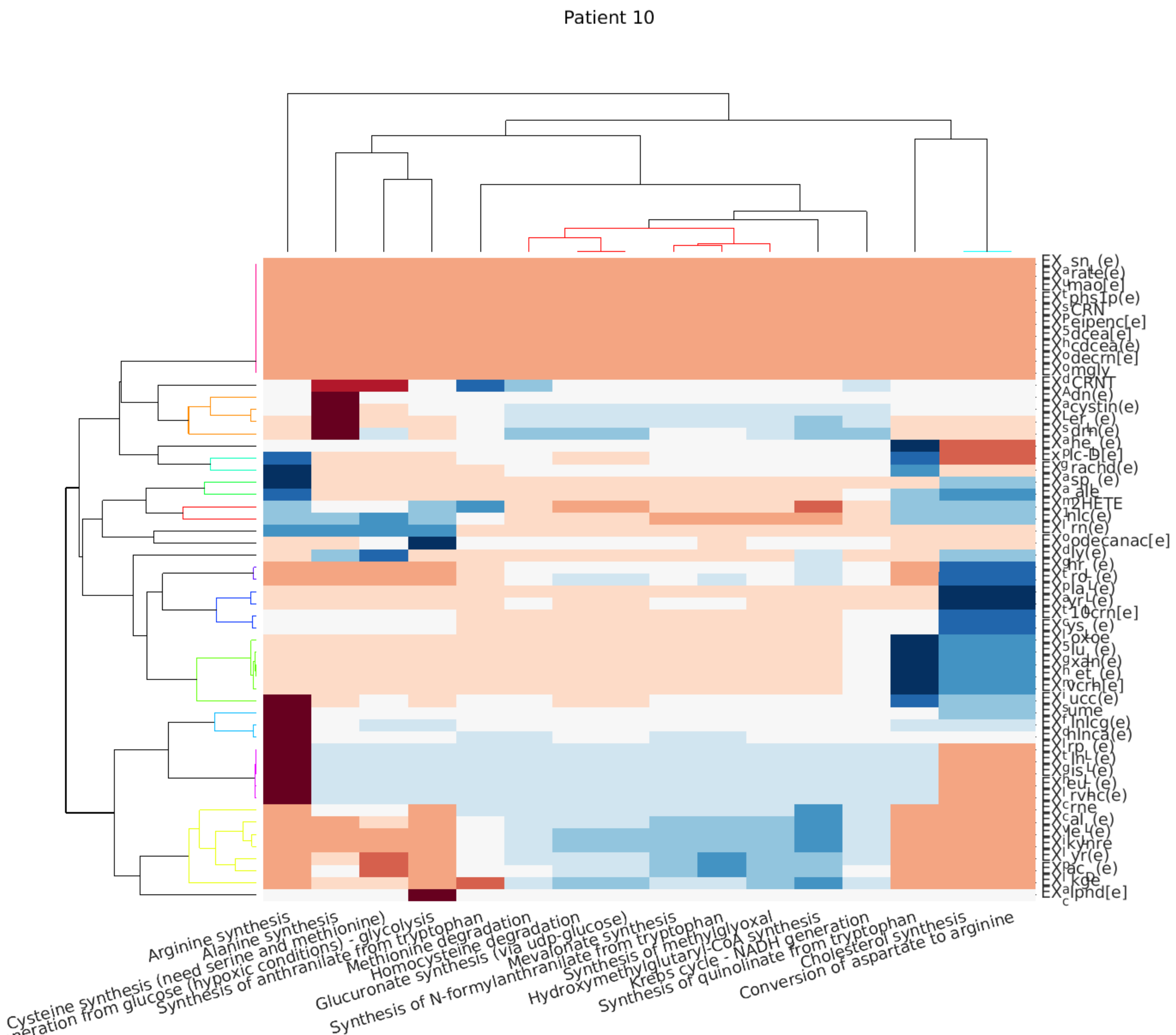
Patient 10
Arginine synthesis
Alanine synthesis
Cysteine synthesis (need serine and methionine)
Synthesis of anthranilate from tryptophan
Methionine degradation
Homocysteine degradation
Glucuronate synthesis (via udp-glucose)
Mevalonate synthesis
Synthesis of N-formylanthranilate from tryptophan
Synthesis of methylglyoxal
Hydroxymethylglutaryl-CoA synthesis
Krebs cycle - NADH generation
Synthesis of quinolinate from tryptophan
Cholesterol synthesis
Conversion of aspartate to arginine

Patient 11

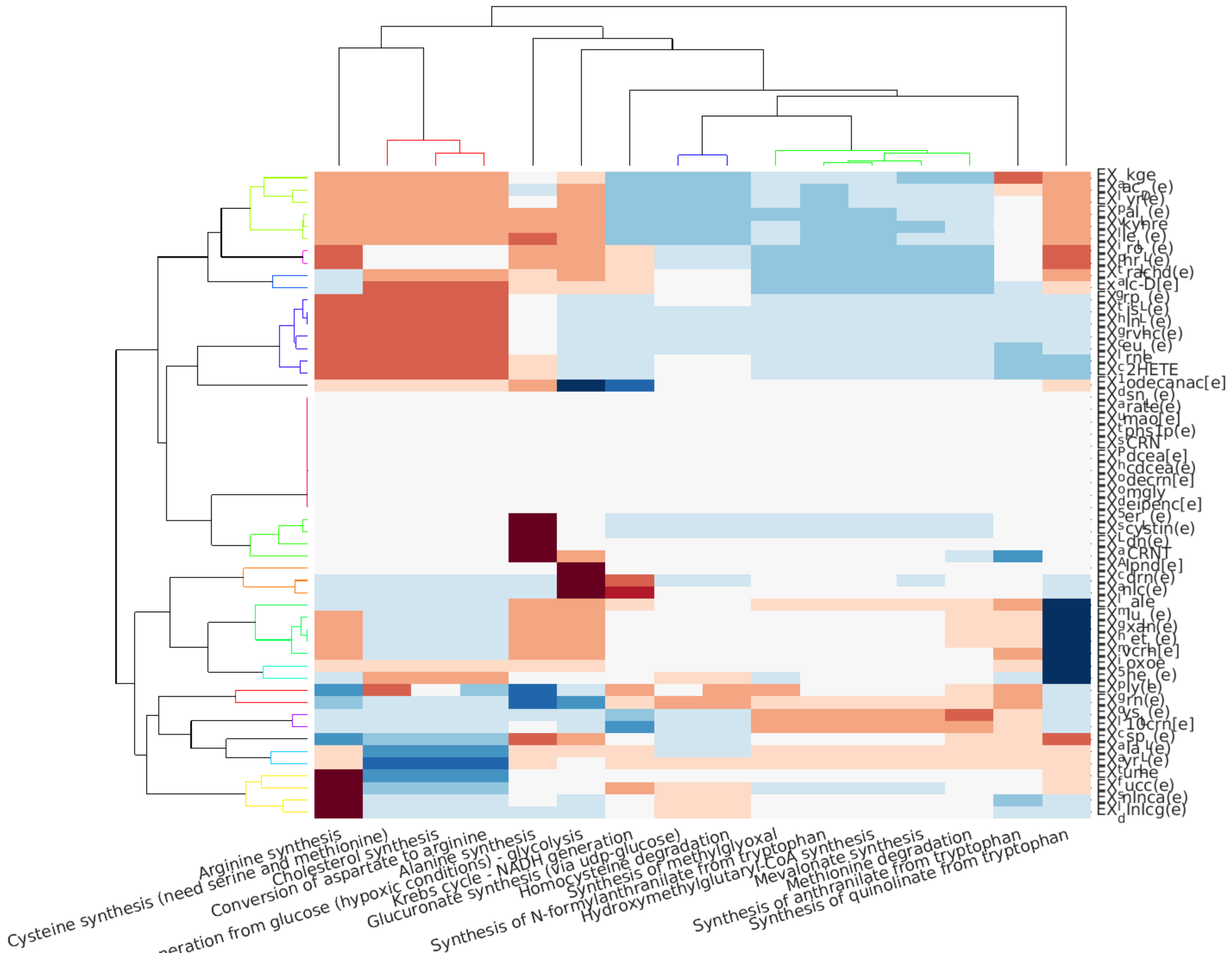
Arginine synthesis
Cysteine synthesis (need serine and methionine)
Cholesterol synthesis
Conversion of aspartate to arginine
Alanine synthesis
eration from glucose (hypoxic conditions) - glycolysis
Krebs cycle - NADH generation
Glucuronate synthesis (via udp-glucose)
Homocysteine degradation
Synthesis of N-formylanthranilate from tryptophan
Synthesis of methylglyoxal
Hydroxymethylglutaryl-CoA synthesis
Mevalonate synthesis
Methionine degradation
Synthesis of anthranilate from tryptophan
Synthesis of quinolinate from tryptophan

Patient 12

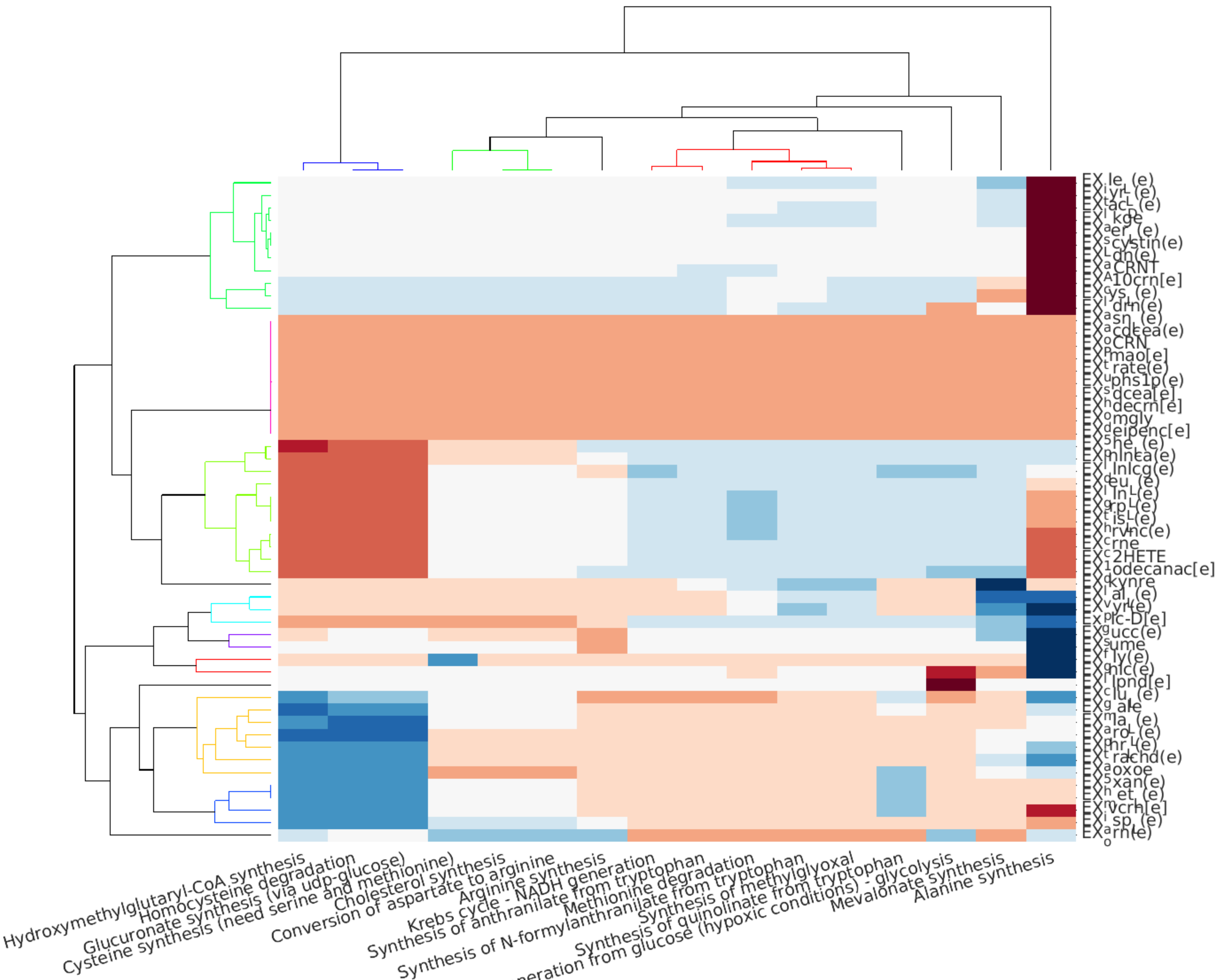
Hydroxymethylglutaryl-CoA synthesis
Homocysteine degradation
Glucuronate synthesis (via udp-glucose)
Cysteine synthesis (need serine and methionine)
Cholesterol synthesis
Conversion of aspartate to arginine
Arginine synthesis
Krebs cycle - NADH generation
Synthesis of anthranilate from tryptophan
Methionine degradation
Synthesis of N-formylanthranilate from tryptophan
Synthesis of methylglyoxal
Synthesis of quinolinate from tryptophan
Mevalonate synthesis
Alanine synthesis

Patient 13

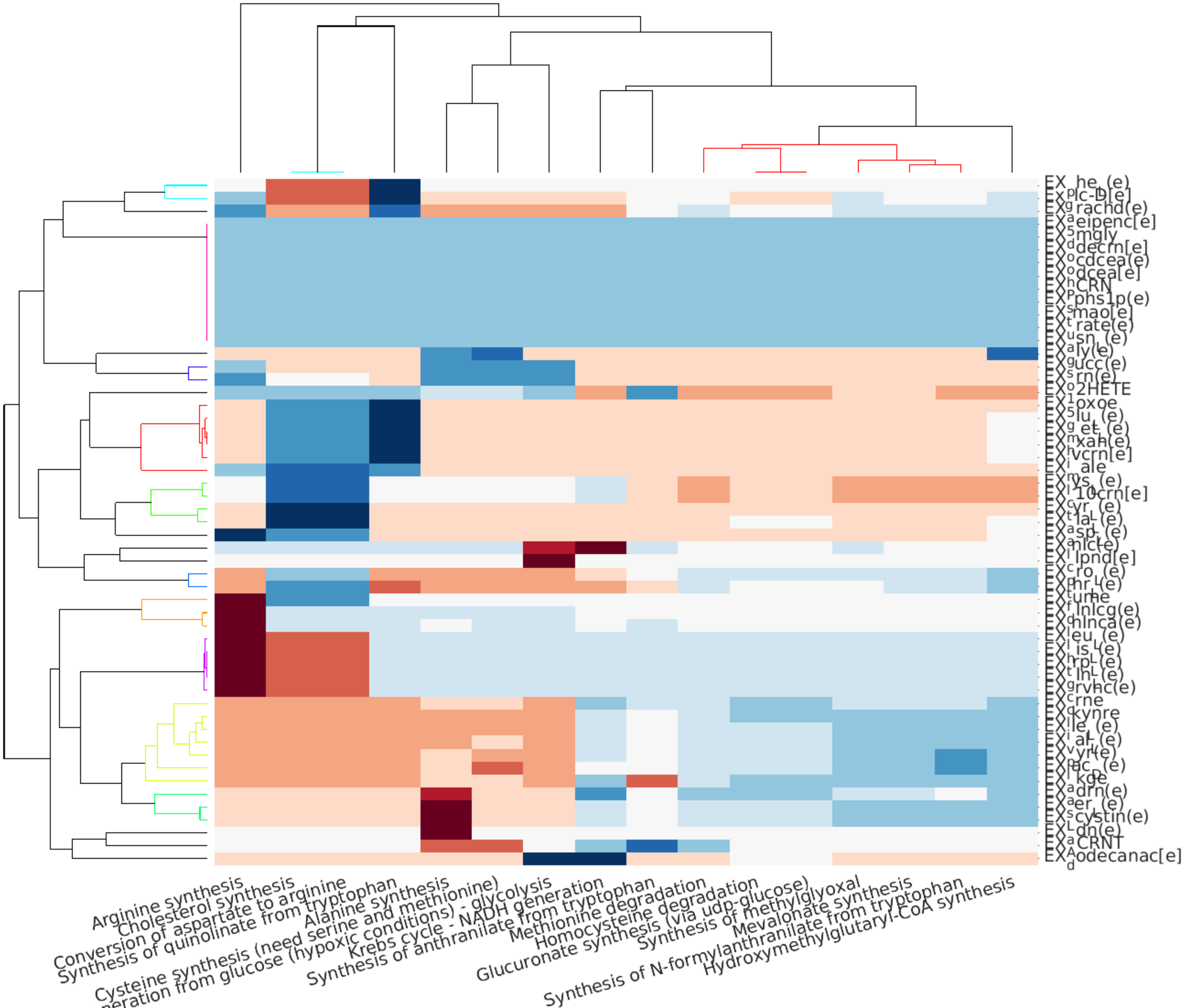
Arginine synthesis
Cholesterol synthesis
Conversion of aspartate to arginine
Synthesis of quinolinate from tryptophan
Alanine synthesis
Cysteine synthesis (need serine and methionine)
Krebs cycle - NADH generation
Synthesis of anthranilate from tryptophan
Methionine degradation
Glucuronate synthesis (via udp-glucose)
Homocysteine degradation
Synthesis of methylglyoxal
Synthesis of N-formylanthranilate from tryptophan
Mevalonate synthesis
Hydroxymethylglutaryl-CoA synthesis
EXo2HETE
EXAodecanac[e]

Patient 14

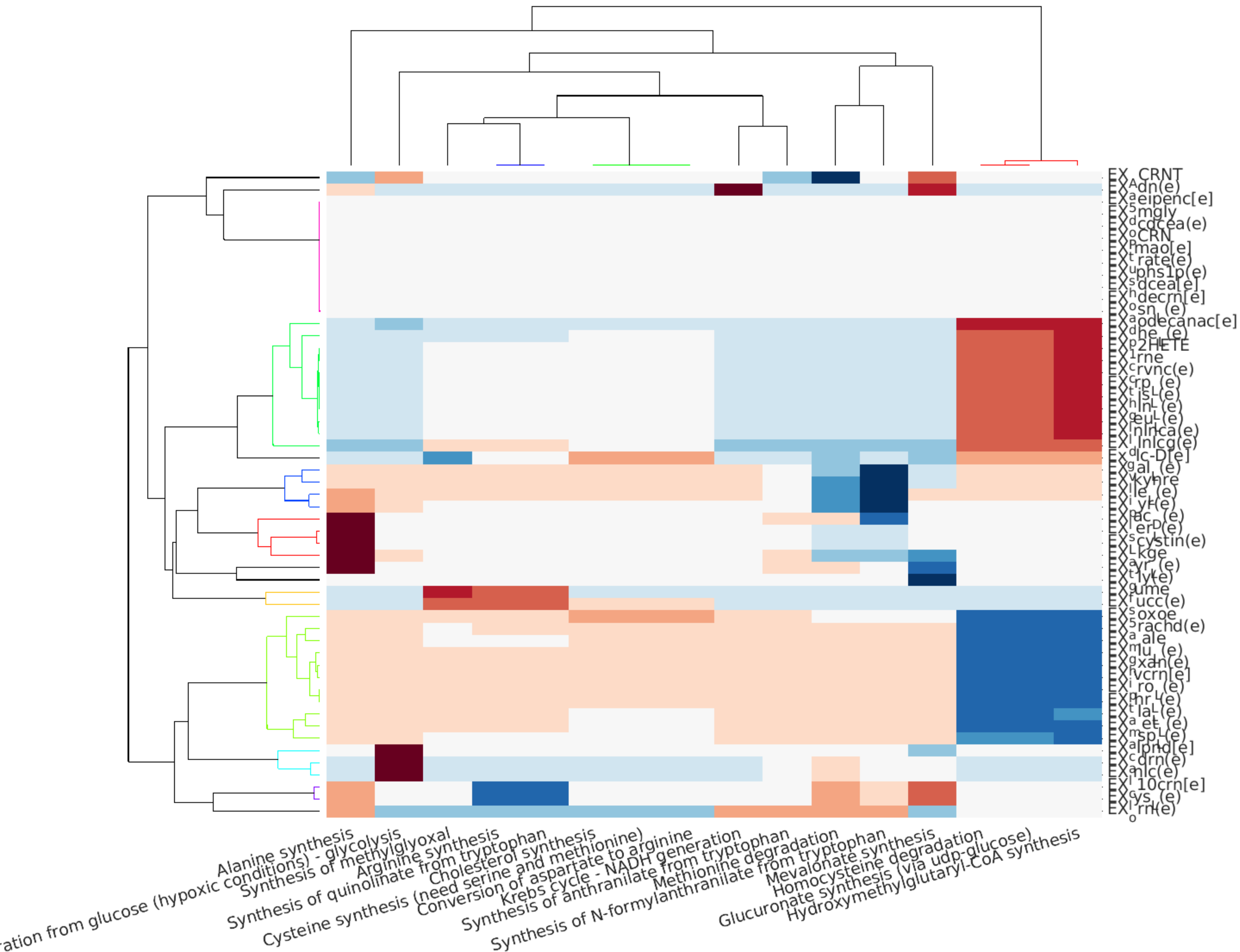
EX_CRNT
Alanine synthesis
ration from glucose (hypoxic conditions) - glycolysis
Synthesis of methylglyoxal
Arginine synthesis
Synthesis of quinolinate from tryptophan
Cholesterol synthesis
Cysteine synthesis (need serine and methionine)
Conversion of aspartate to arginine
Krebs cycle - NADH generation
Synthesis of anthranilate from tryptophan
Methionine degradation
Synthesis of N-formylanthranilate from tryptophan
Mevalonate synthesis
Homocysteine degradation
Glucuronate synthesis (via udp-glucose)
Hydroxymethylglutaryl-CoA synthesis

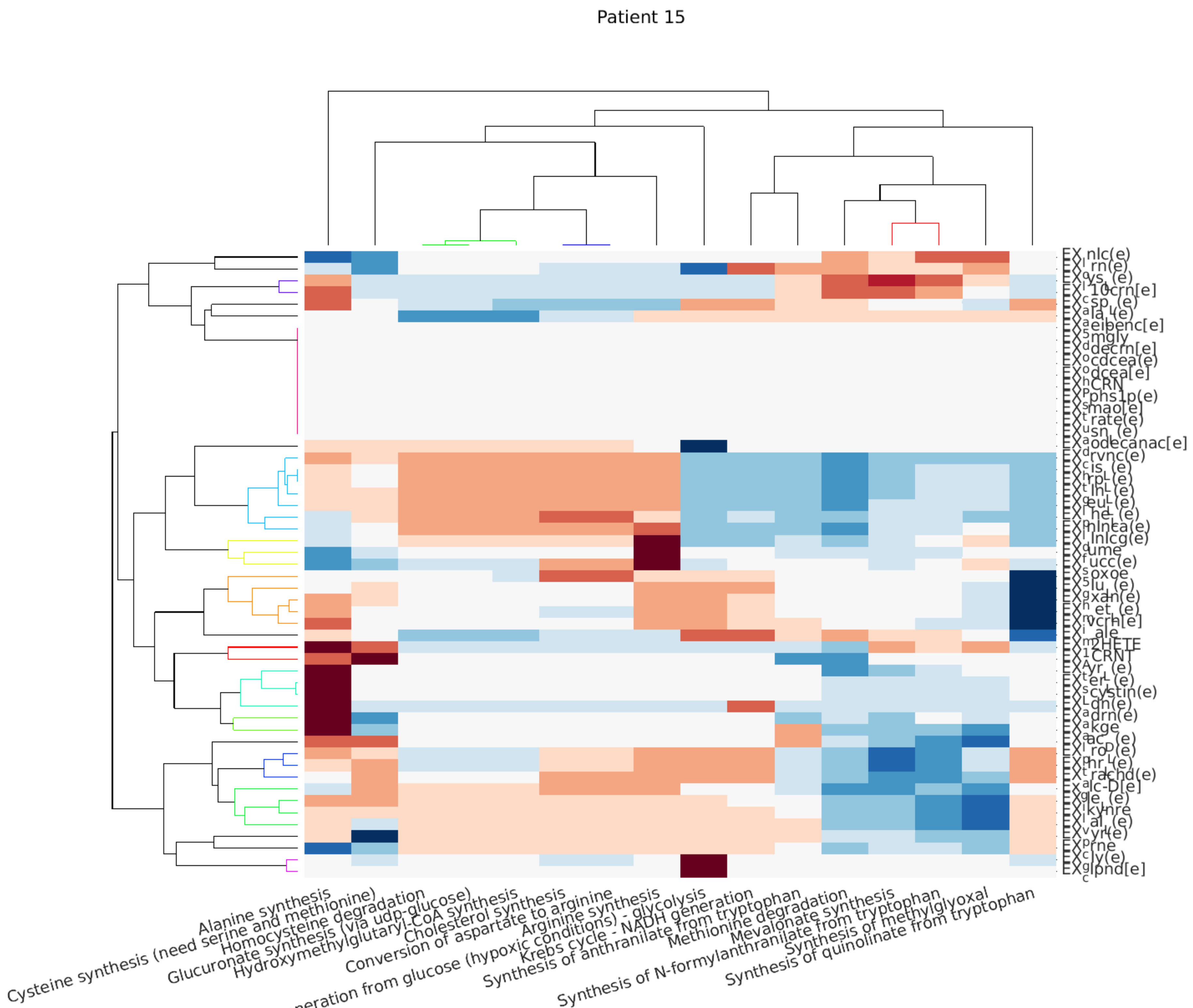

Patient 15
Alanine synthesis
Cysteine synthesis (need serine and methionine)
Homocysteine degradation
Glucuronate synthesis (via udp-glucose)
Hydroxymethylglutaryl-CoA synthesis
Cholesterol synthesis
Conversion of aspartate to arginine
Arginine synthesis
Krebs cycle - NADH generation
Synthesis of anthranilate from tryptophan
Methionine degradation
Mevalonate synthesis
Synthesis of N-formylanthranilate from tryptophan
Synthesis of methylglyoxal
Synthesis of quinolinate from tryptophan

Patient 16

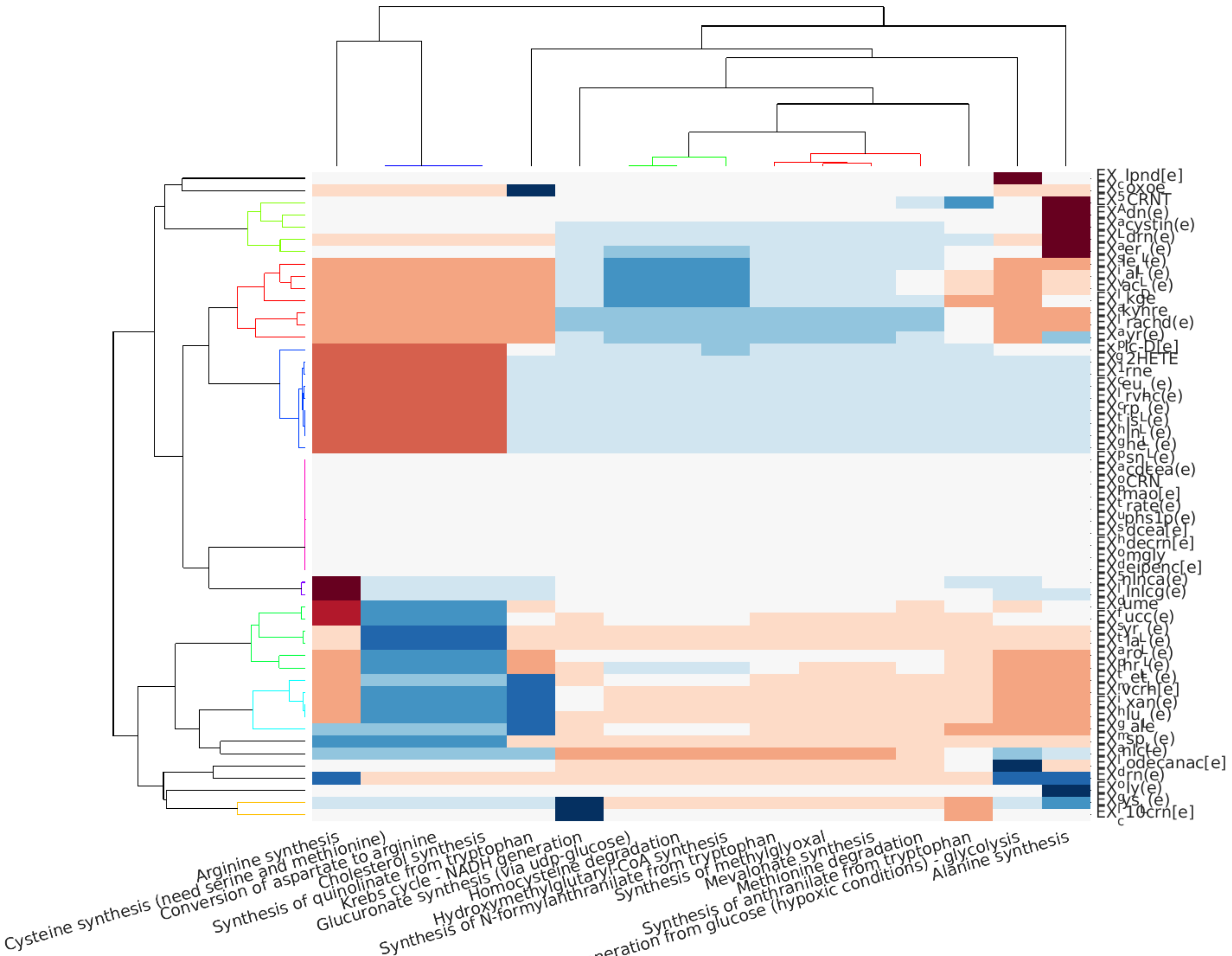
Arginine synthesis
Cysteine synthesis (need serine and methionine)
Conversion of aspartate to arginine
Cholesterol synthesis
Synthesis of quinolinate from tryptophan
Krebs cycle - NADH generation
Glucuronate synthesis (via udp-glucose)
Homocysteine degradation
Hydroxymethylglutaryl-CoA synthesis
Synthesis of N-formylanthranilate from tryptophan
Synthesis of methylglyoxal
Mevalonate synthesis
Methionine degradation
Synthesis of anthranilate from tryptophan
glycolysis
Alanine synthesis

Patient 17

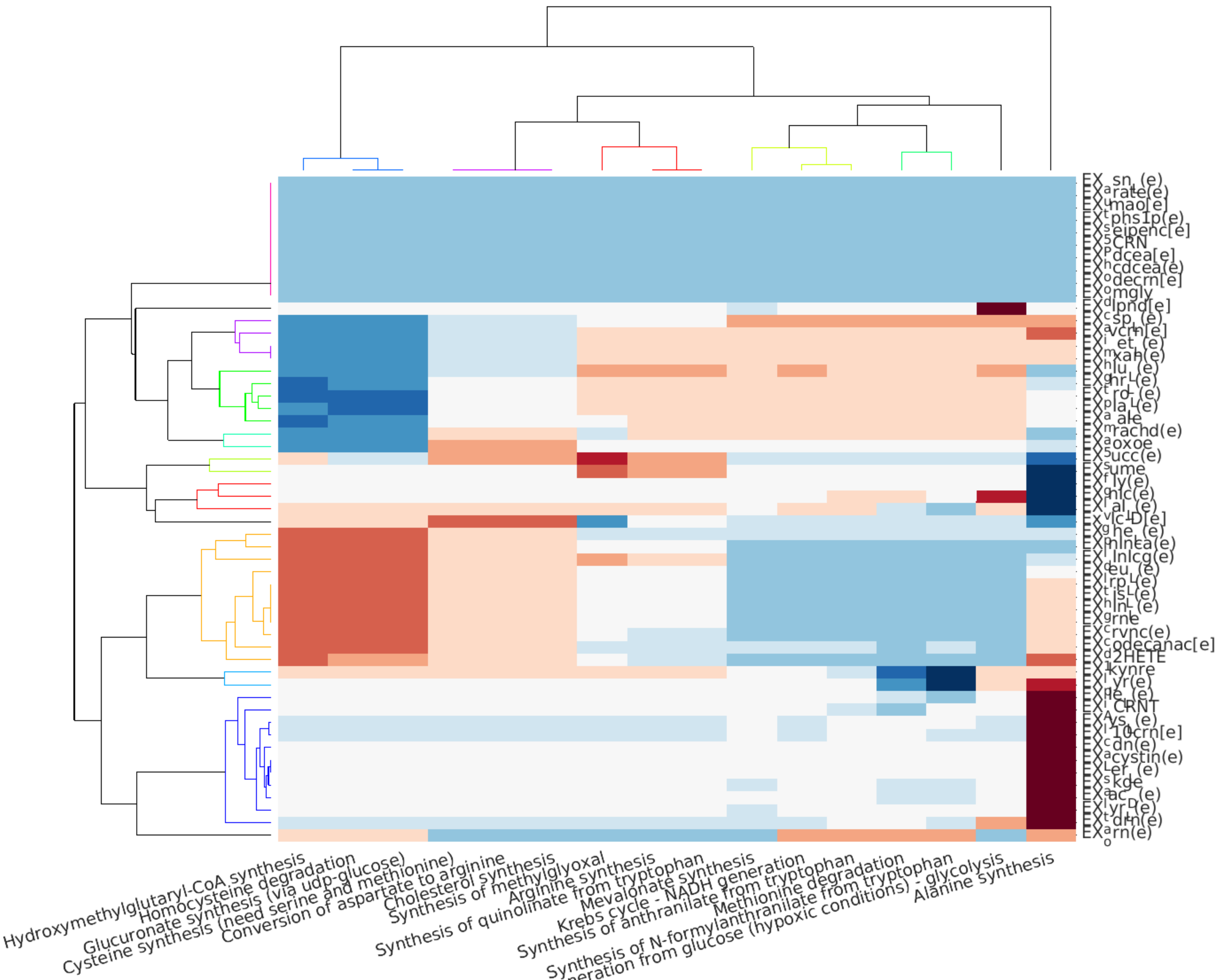

Hydroxymethylglutaryl-CoA synthesis
Homocysteine degradation
Glucuronate synthesis (via udp-glucose)
Cysteine synthesis (need serine and methionine)
Conversion of aspartate to arginine
Cholesterol synthesis
Synthesis of methylglyoxal
Arginine synthesis
Synthesis of quinolinate from tryptophan
Mevalonate synthesis
Krebs cycle - NADH generation
Synthesis of anthranilate from tryptophan
Methionine degradation
Synthesis of N-formylanthranilate from tryptophan
Alanine synthesis

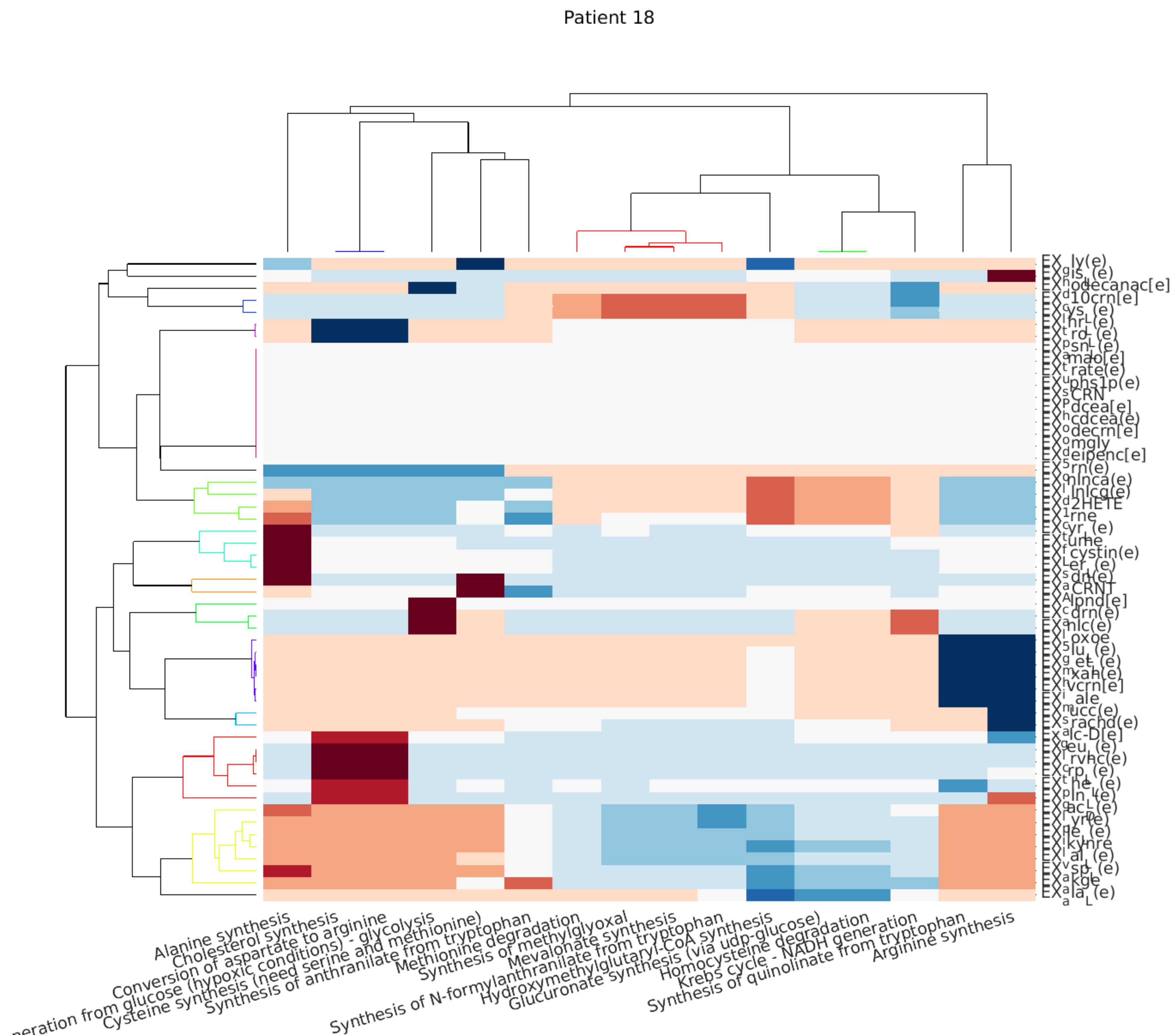
Patient 18
Alanine synthesis
Cholesterol synthesis
Conversion of aspartate to arginine
Cysteine synthesis (need serine and methionine)
Synthesis of anthranilate from tryptophan
Methionine degradation
Synthesis of methylglyoxal
Synthesis of N-formylanthranilate from tryptophan
Mevalonate synthesis
Hydroxymethylglutaryl-CoA synthesis
Glucuronate synthesis (via udp-glucose)
Homocysteine degradation
Krebs cycle - NADH generation
Synthesis of quinolinate from tryptophan
Arginine synthesis

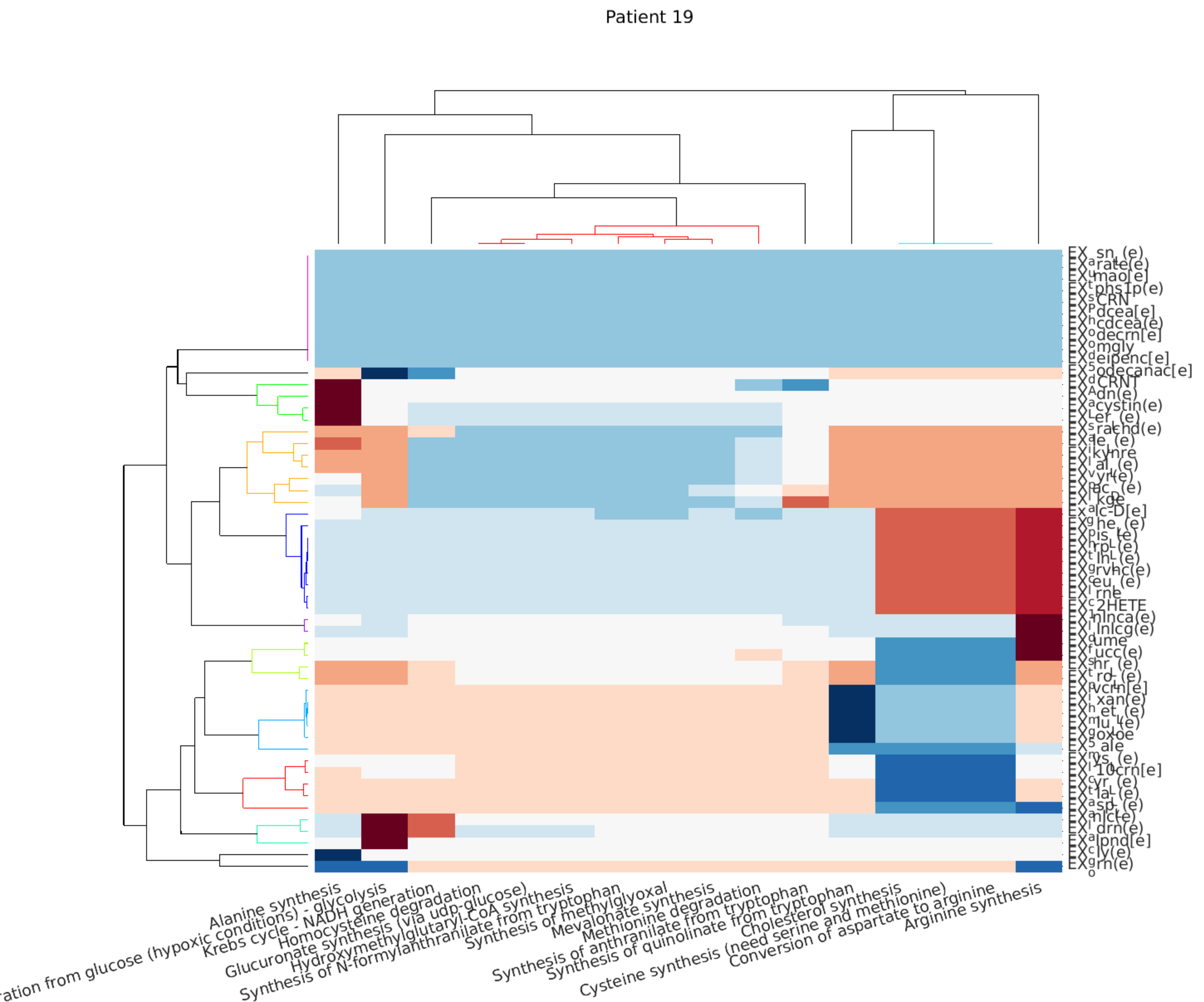

Patient 19
Alanine synthesis
Krebs cycle - NADH generation
Homocysteine degradation
Glucuronate synthesis (via udp-glucose)
Hydroxymethylglutaryl-CoA synthesis
Synthesis of N-formylanthranilate from tryptophan
Synthesis of methylglyoxal
Mevalonate synthesis
Methionine degradation
Synthesis of anthranilate from tryptophan
Synthesis of quinolinate from tryptophan
Cholesterol synthesis
Cysteine synthesis (need serine and methionine)
Conversion of aspartate to arginine
Arginine synthesis

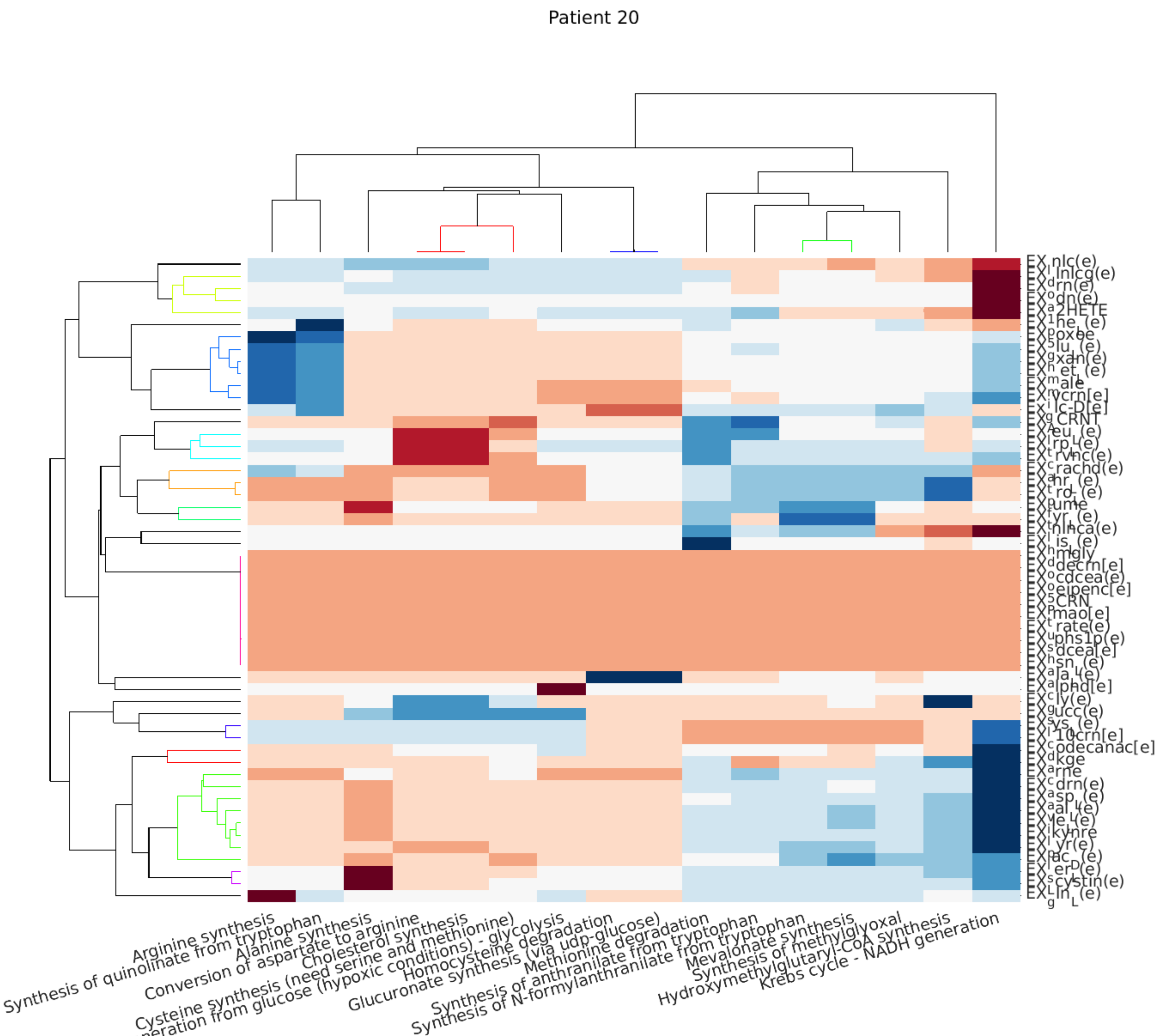
Patient 20
Arginine synthesis
Synthesis of quinolinate from tryptophan
Alanine synthesis
Conversion of aspartate to arginine
Cholesterol synthesis
Cysteine synthesis (need serine and methionine)
neration from glucose (hypoxic conditions) - glycolysis
Homocysteine degradation
Glucuronate synthesis (via udp-glucose)
Methionine degradation
Synthesis of anthranilate from tryptophan
Synthesis of N-formylanthranilate from tryptophan
Mevalonate synthesis
Synthesis of methylglyoxal
Hydroxymethylglutaryl-CoA synthesis
Krebs cycle - NADH generation

Patient 21

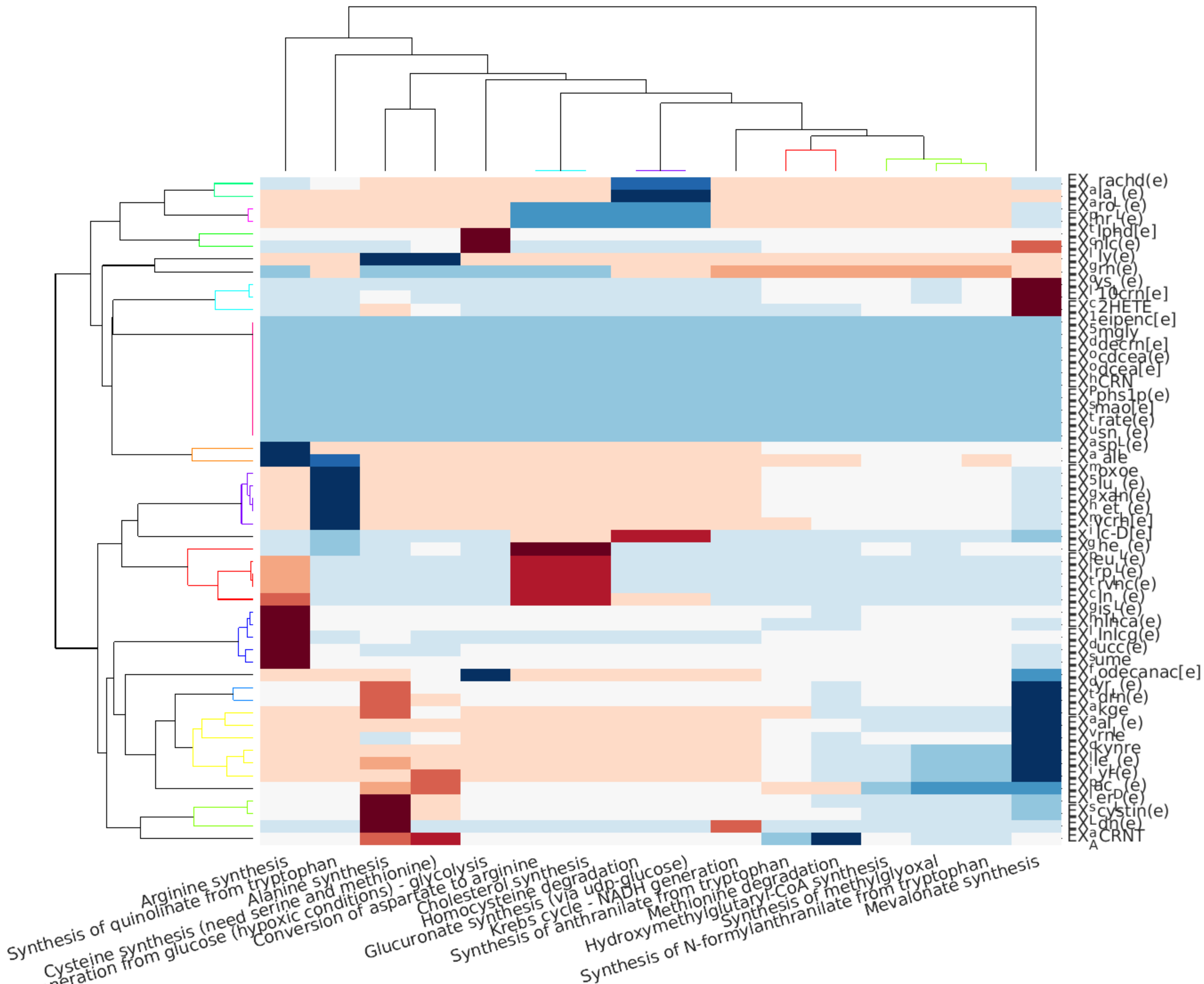

Arginine synthesis
Synthesis of quinolinate from tryptophan
Alanine synthesis
Cysteine synthesis (need serine and methionine)
Conversion of aspartate to arginine
Cholesterol synthesis
Homocysteine degradation
Glucuronate synthesis (via udp-glucose)
Krebs cycle - NADH generation
Synthesis of anthranilate from tryptophan
Methionine degradation
Hydroxymethylglutaryl-CoA synthesis
Synthesis of methylglyoxal
Synthesis of N-formylanthranilate from tryptophan
Mevalonate synthesis

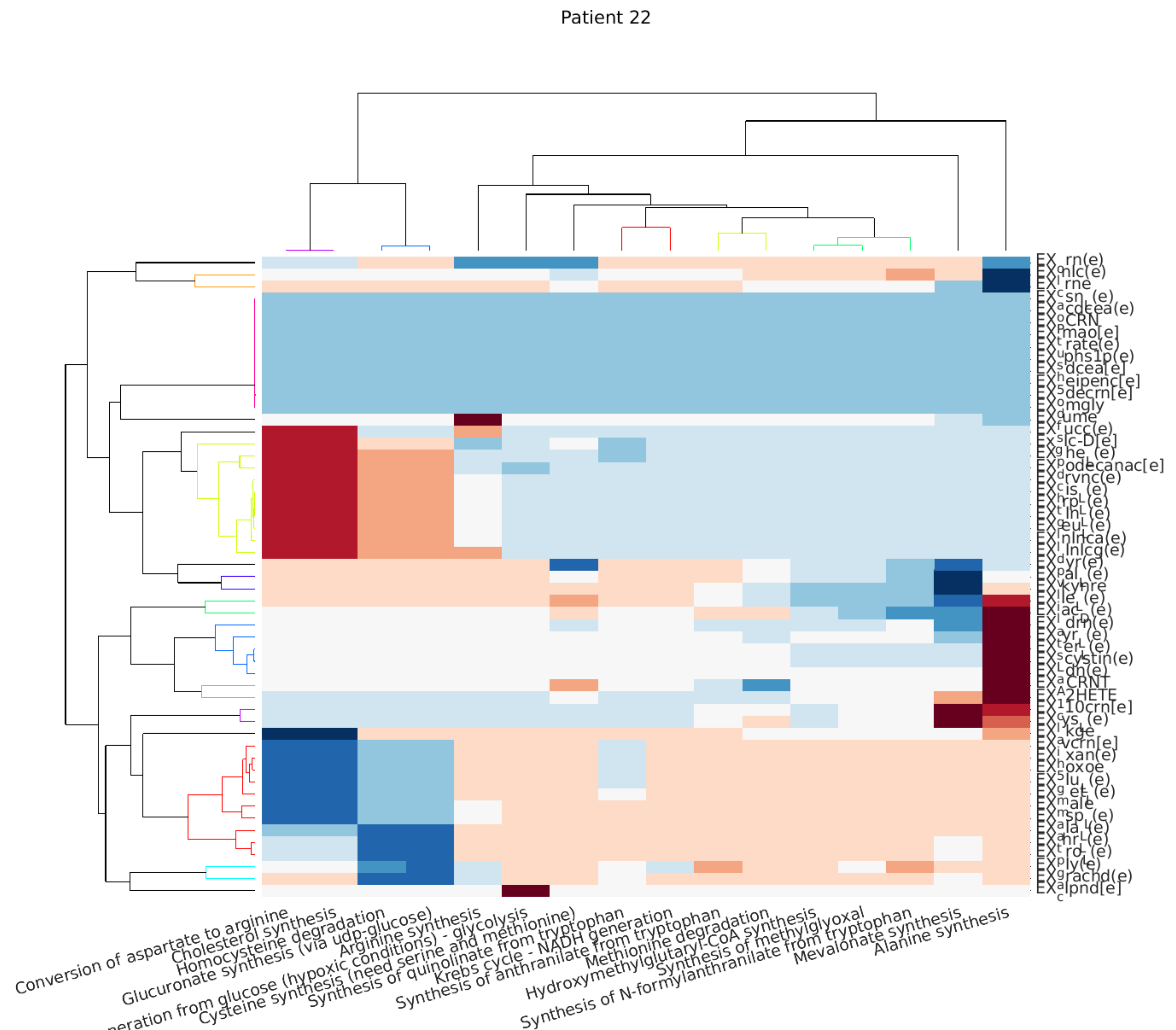
Patient 22
Conversion of aspartate to arginine
Cholesterol synthesis
Homocysteine degradation
Glucuronate synthesis (via udp-glucose)
Arginine synthesis
from glucose (hypoxic conditions) - glycolysis
Cysteine synthesis (need serine and methionine)
Synthesis of quinolinate from tryptophan
Krebs cycle - NADH generation
Synthesis of anthranilate from tryptophan
Methionine degradation
Hydroxymethylglutaryl-CoA synthesis
Synthesis of methylglyoxal
Synthesis of N-formylanthranilate from tryptophan
Mevalonate synthesis
Alanine synthesis

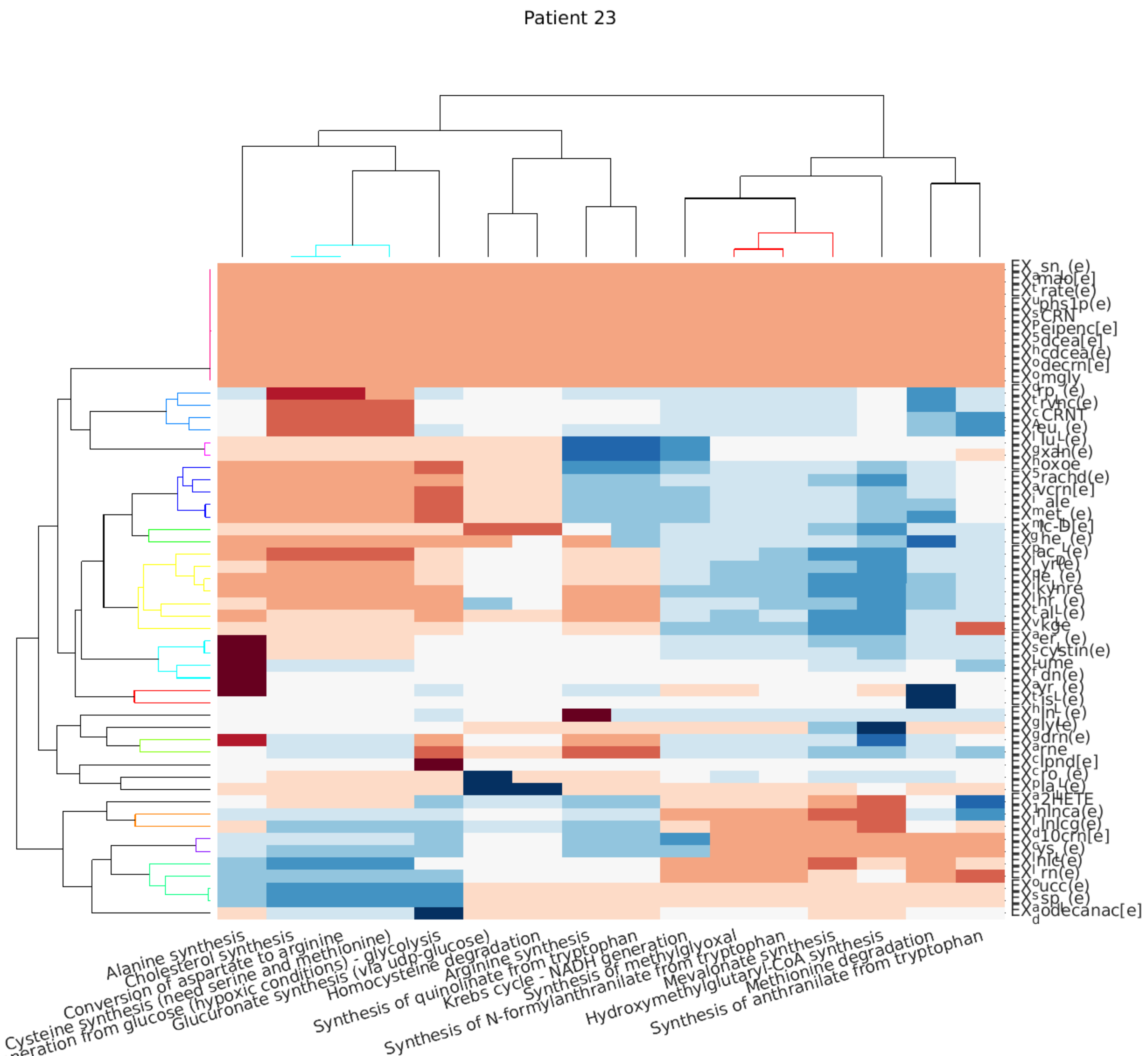
Patient 23
Alanine synthesis
Cholesterol synthesis
Conversion of aspartate to arginine
Cysteine synthesis (need serine and methionine)
from glucose (hypoxic conditions) - glycolysis
Glucuronate synthesis (via udp-glucose)
Homocysteine degradation
Synthesis of quinolinate from tryptophan
Arginine synthesis
Krebs cycle - NADH generation
Synthesis of N-formylanthranilate from tryptophan
Synthesis of methylglyoxal
Hydroxymethylglutaryl-CoA synthesis
Mevalonate synthesis
Methionine degradation
Synthesis of anthranilate from tryptophan

Patient 24

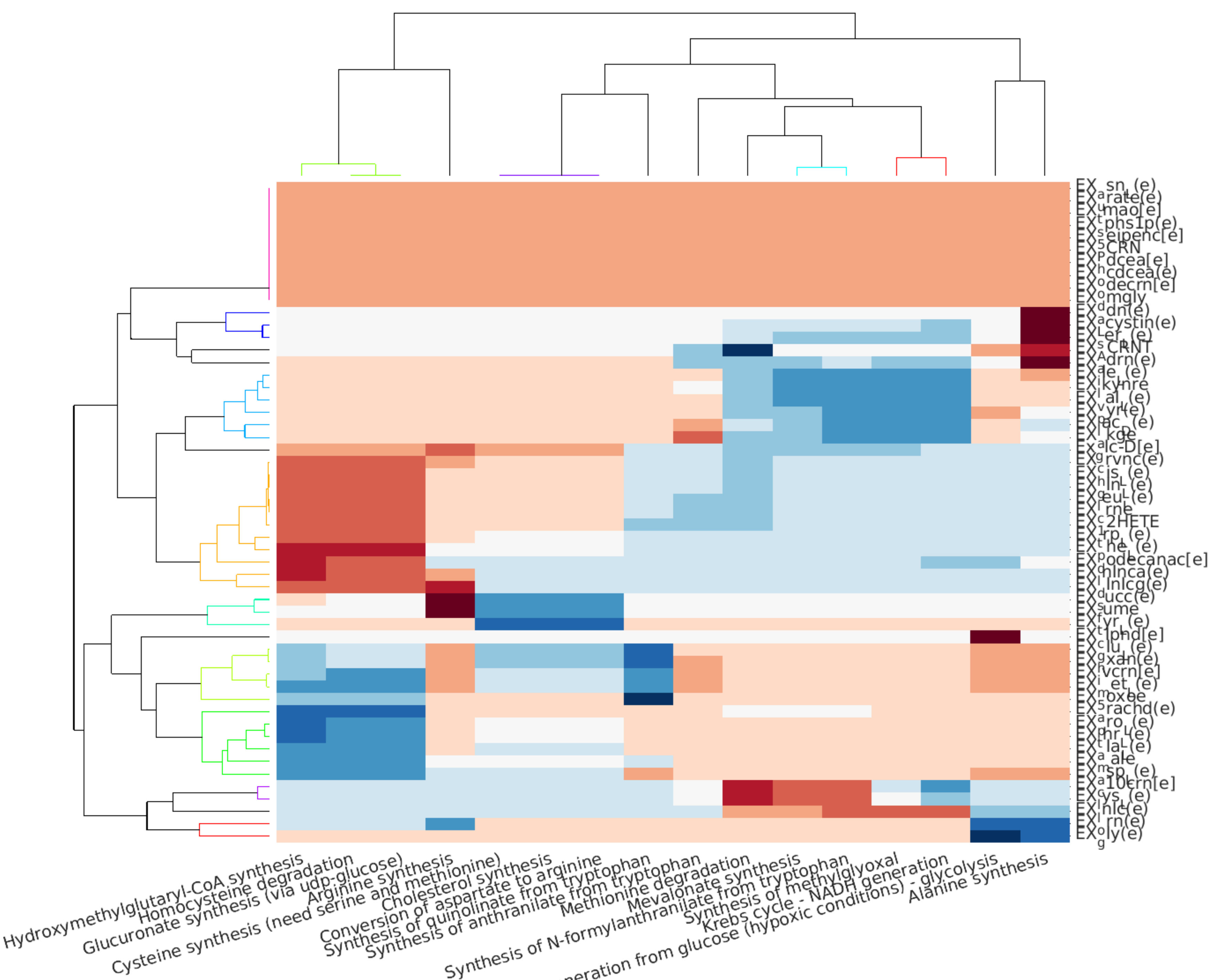
Hydroxymethylglutaryl-CoA synthesis
Homocysteine degradation
Glucuronate synthesis (via udp-glucose)
Arginine synthesis
Cysteine synthesis (need serine and methionine)
Cholesterol synthesis
Conversion of aspartate to arginine
Synthesis of quinolinate from tryptophan
Synthesis of anthranilate from tryptophan
Methionine degradation
Synthesis of N-formylanthranilate from tryptophan
Mevalonate synthesis
Synthesis of methylglyoxal
Krebs cycle - NADH generation
Alanine synthesis

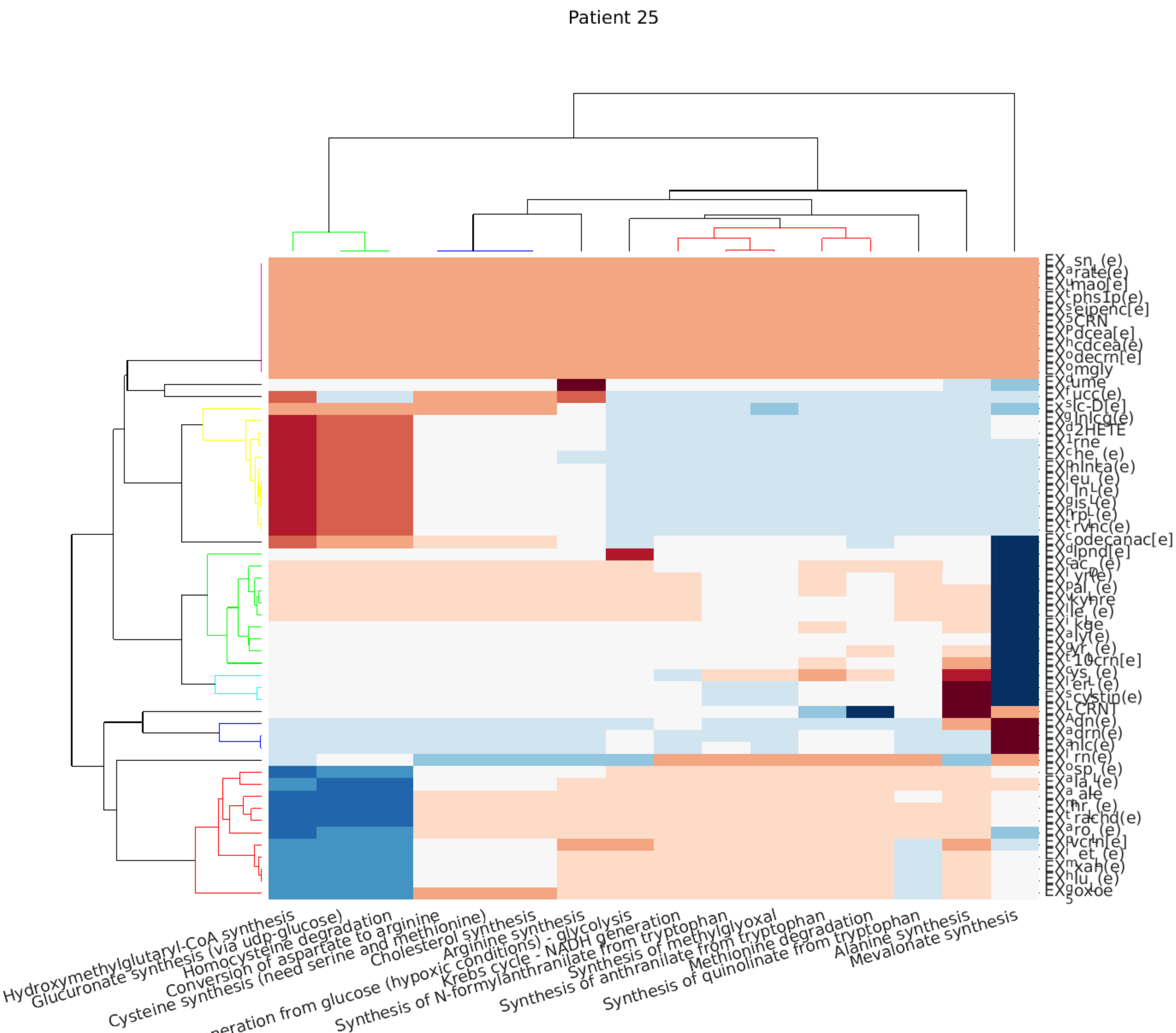
Patient 25
Hydroxymethylglutaryl-CoA synthesis
Glucuronate synthesis (via udp-glucose)
Homocysteine degradation
Conversion of aspartate to arginine
Cysteine synthesis (need serine and methionine)
Cholesterol synthesis
Arginine synthesis
Krebs cycle - NADH generation
Synthesis of N-formylanthranilate from tryptophan
Synthesis of methylglyoxal
Synthesis of anthranilate from tryptophan
Methionine degradation
Synthesis of quinolinate from tryptophan
Alanine synthesis
Mevalonate synthesis

Patient 26

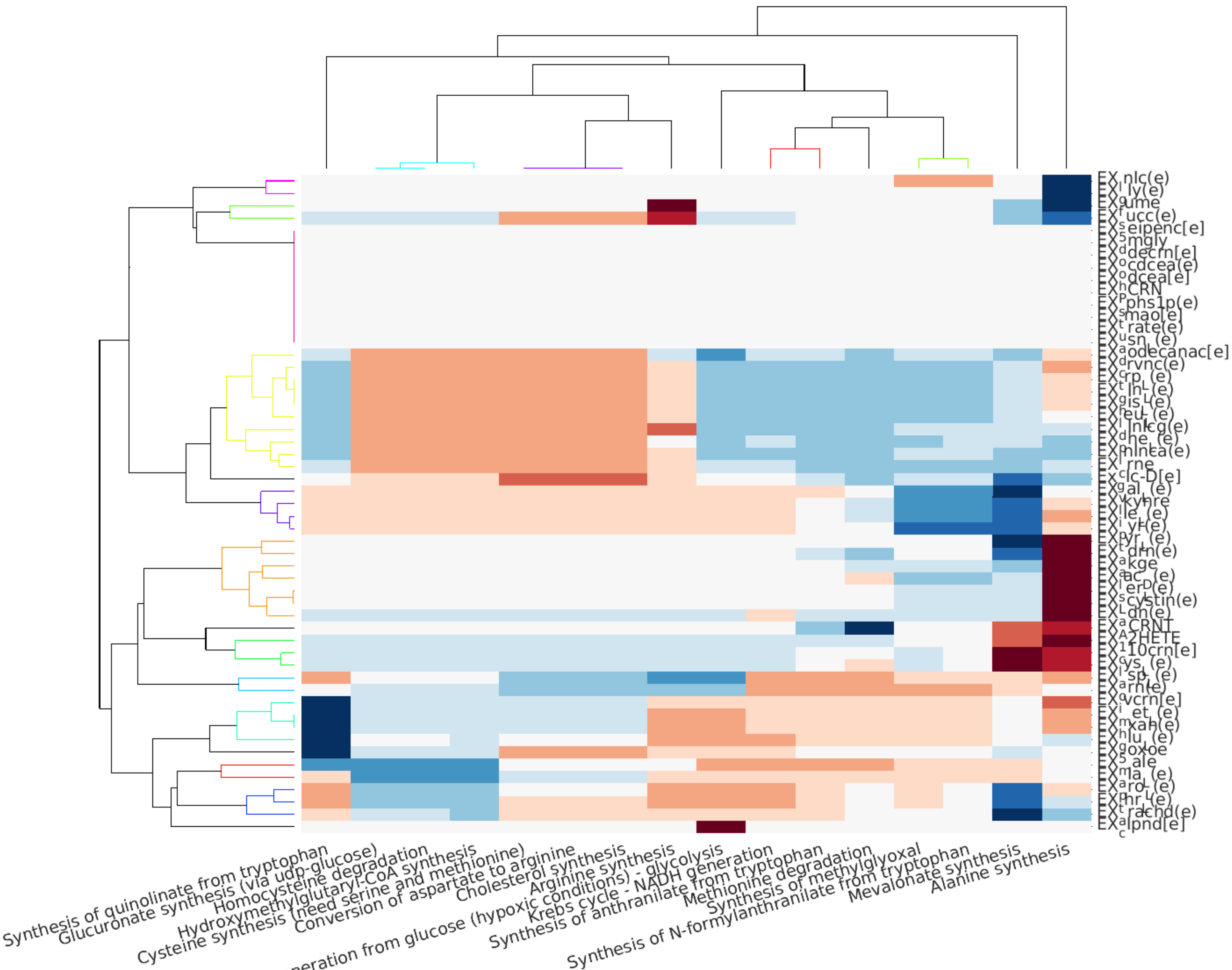
Synthesis of quinolinate from tryptophan
Glucuronate synthesis (via udp-glucose)
Homocysteine degradation
Hydroxymethylglutaryl-CoA synthesis
Cysteine synthesis (need serine and methionine)
Conversion of aspartate to arginine
Cholesterol synthesis
Arginine synthesis
Krebs cycle - NADH generation
Synthesis of anthranilate from tryptophan
Methionine degradation
Synthesis of methylglyoxal
Synthesis of N-formylanthranilate from tryptophan
Mevalonate synthesis
Alanine synthesis

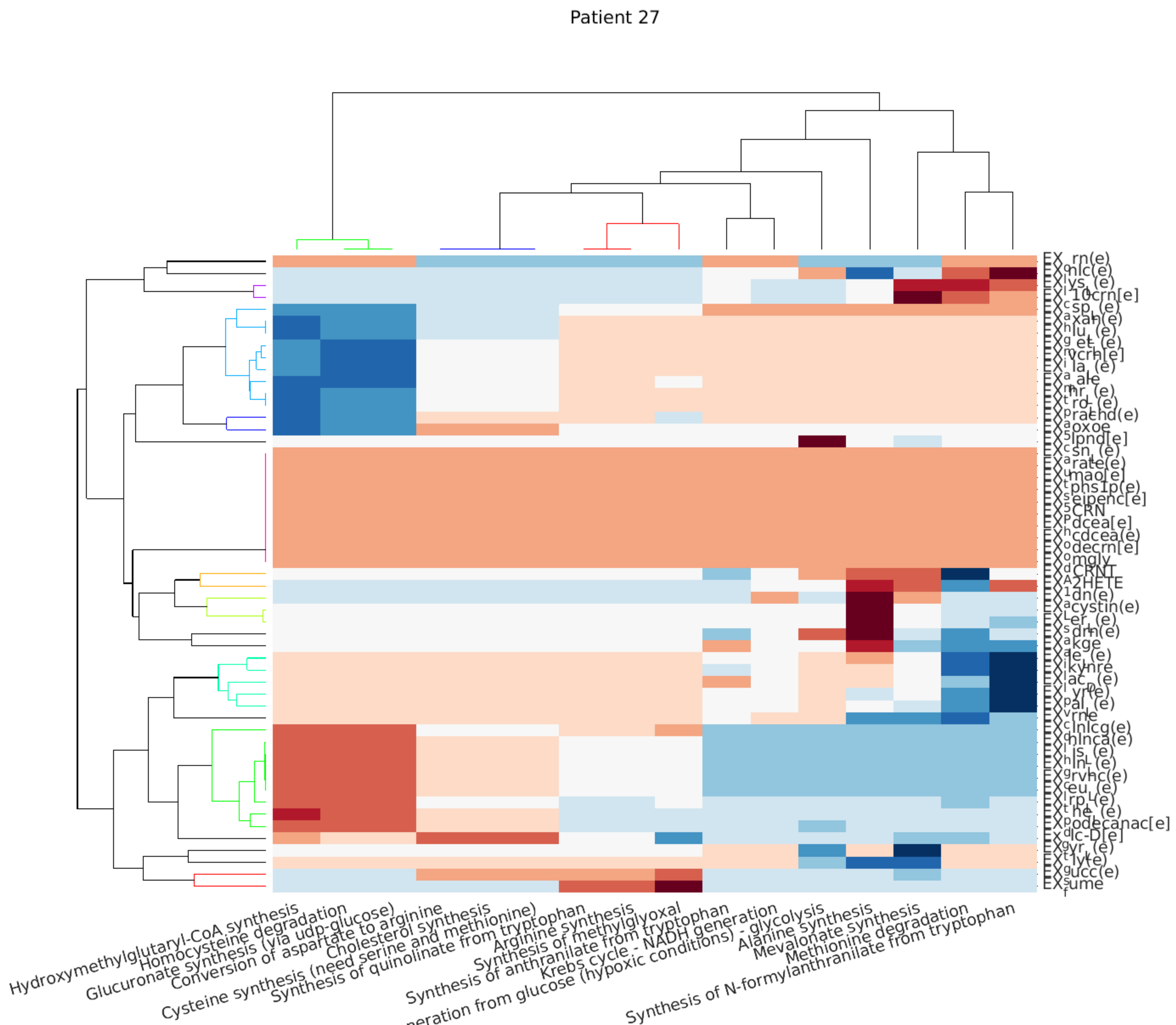
Patient 27
Hydroxymethylglutaryl-CoA synthesis
Homocysteine degradation
Glucuronate synthesis (via udp-glucose)
Conversion of aspartate to arginine
Cholesterol synthesis
Cysteine synthesis (need serine and methionine)
Synthesis of quinolinate from tryptophan
Arginine synthesis
Synthesis of methylglyoxal
Synthesis of anthranilate from tryptophan
Krebs cycle - NADH generation
Alanine synthesis
Mevalonate synthesis
Methionine degradation
Synthesis of N-formylanthranilate from tryptophan

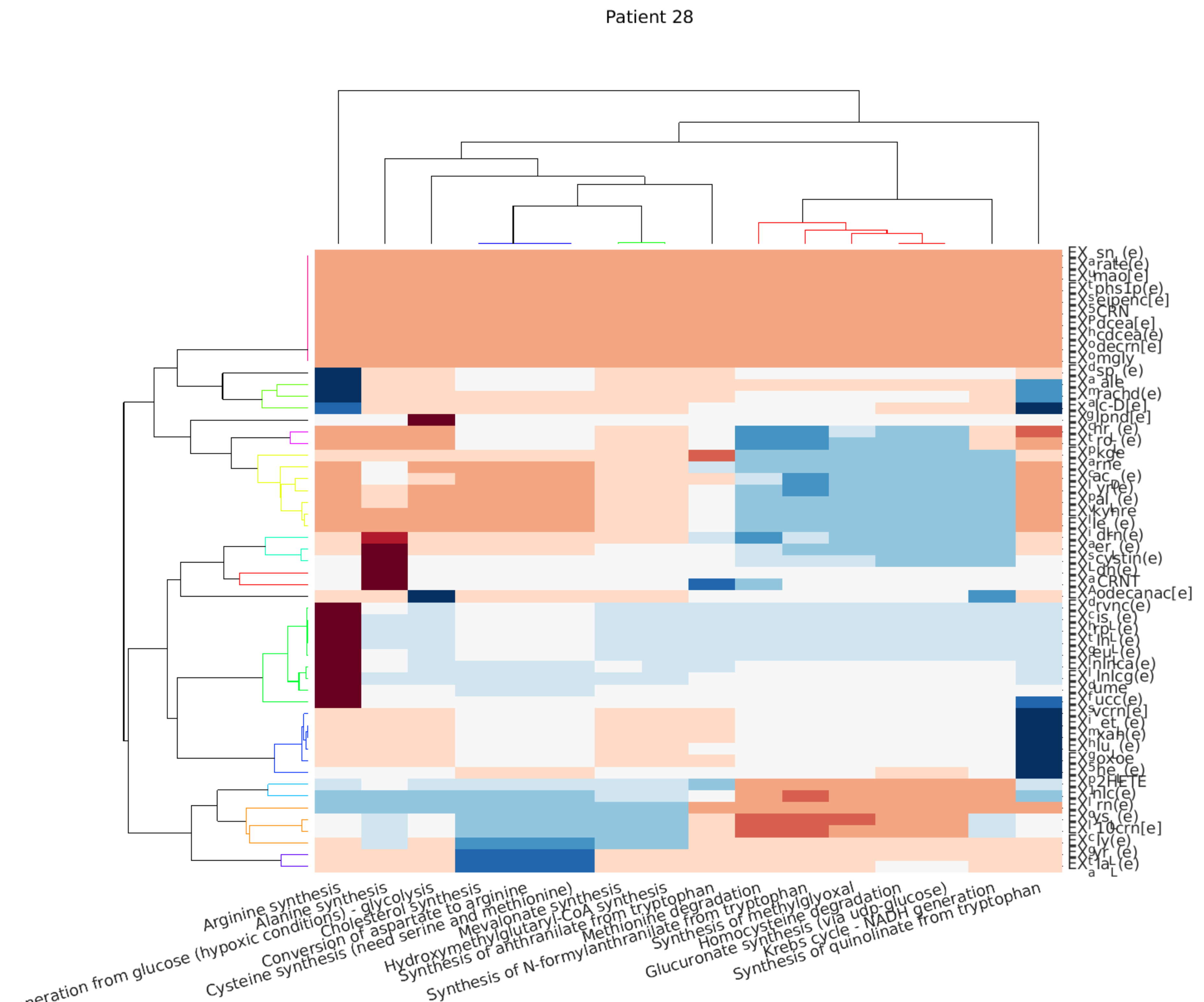
Patient 28
Arginine synthesis
Alanine synthesis
eneration from glucose (hypoxic conditions) - glycolysis
Cholesterol synthesis
Conversion of aspartate to arginine
Cysteine synthesis (need serine and methionine)
Mevalonate synthesis
Hydroxymethylglutaryl-CoA synthesis
Synthesis of anthranilate from tryptophan
Methionine degradation
Synthesis of N-formylanthranilate from tryptophan
Synthesis of methylglyoxal
Glucuronate synthesis (via udp-glucose)
Homocysteine degradation
Krebs cycle - NADH generation
Synthesis of quinolinate from tryptophan

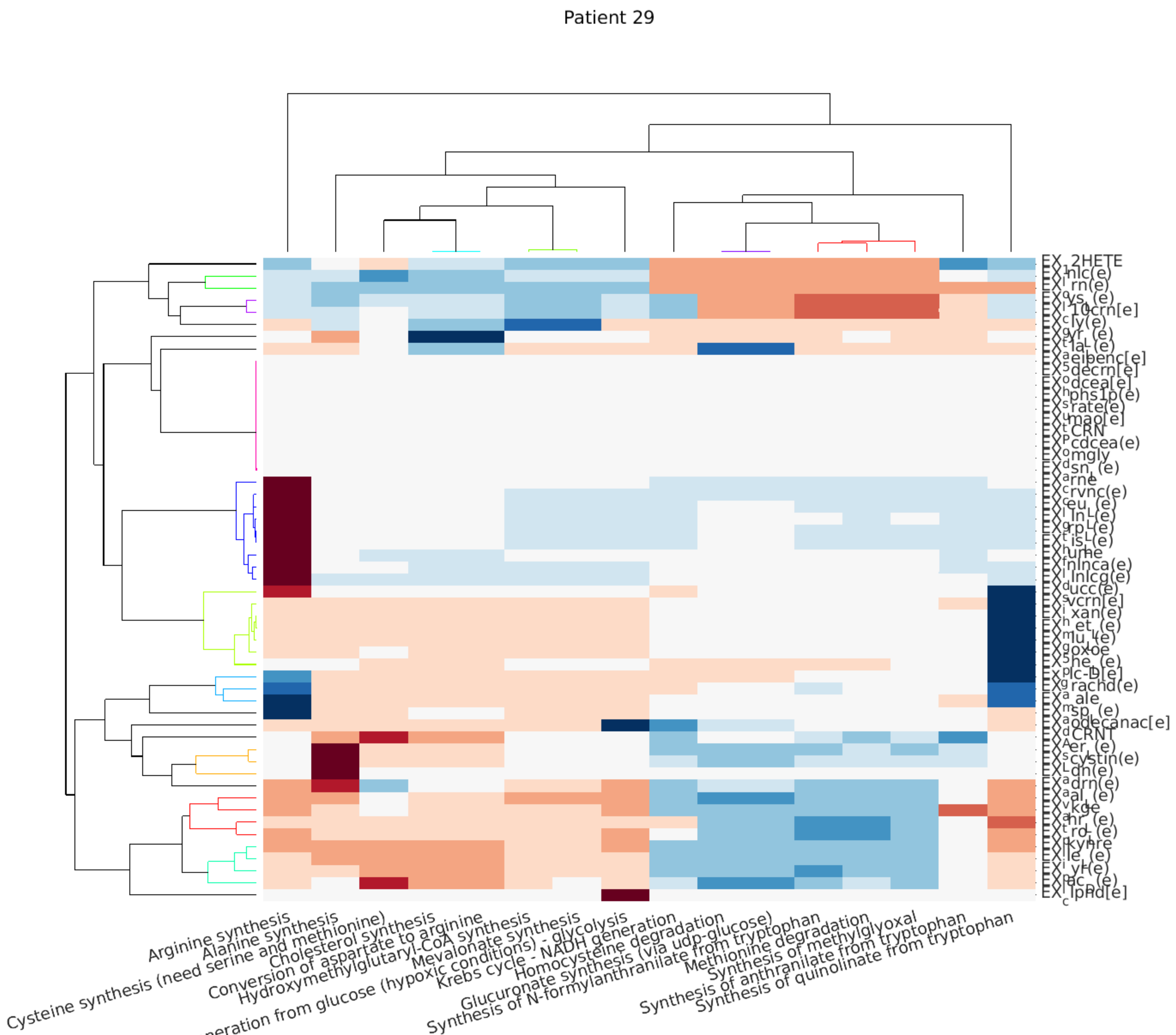
Patient 29
Arginine synthesis
Alanine synthesis
Cysteine synthesis (need serine and methionine)
Cholesterol synthesis
Conversion of aspartate to arginine
Hydroxymethylglutaryl-CoA synthesis
Mevalonate synthesis
Krebs cycle - NADH generation
Homocysteine degradation
Glucuronate synthesis (via udp-glucose)
Synthesis of N-formylanthranilate from tryptophan
Methionine degradation
Synthesis of methylglyoxal
Synthesis of anthranilate from tryptophan
Synthesis of quinolinate from tryptophan

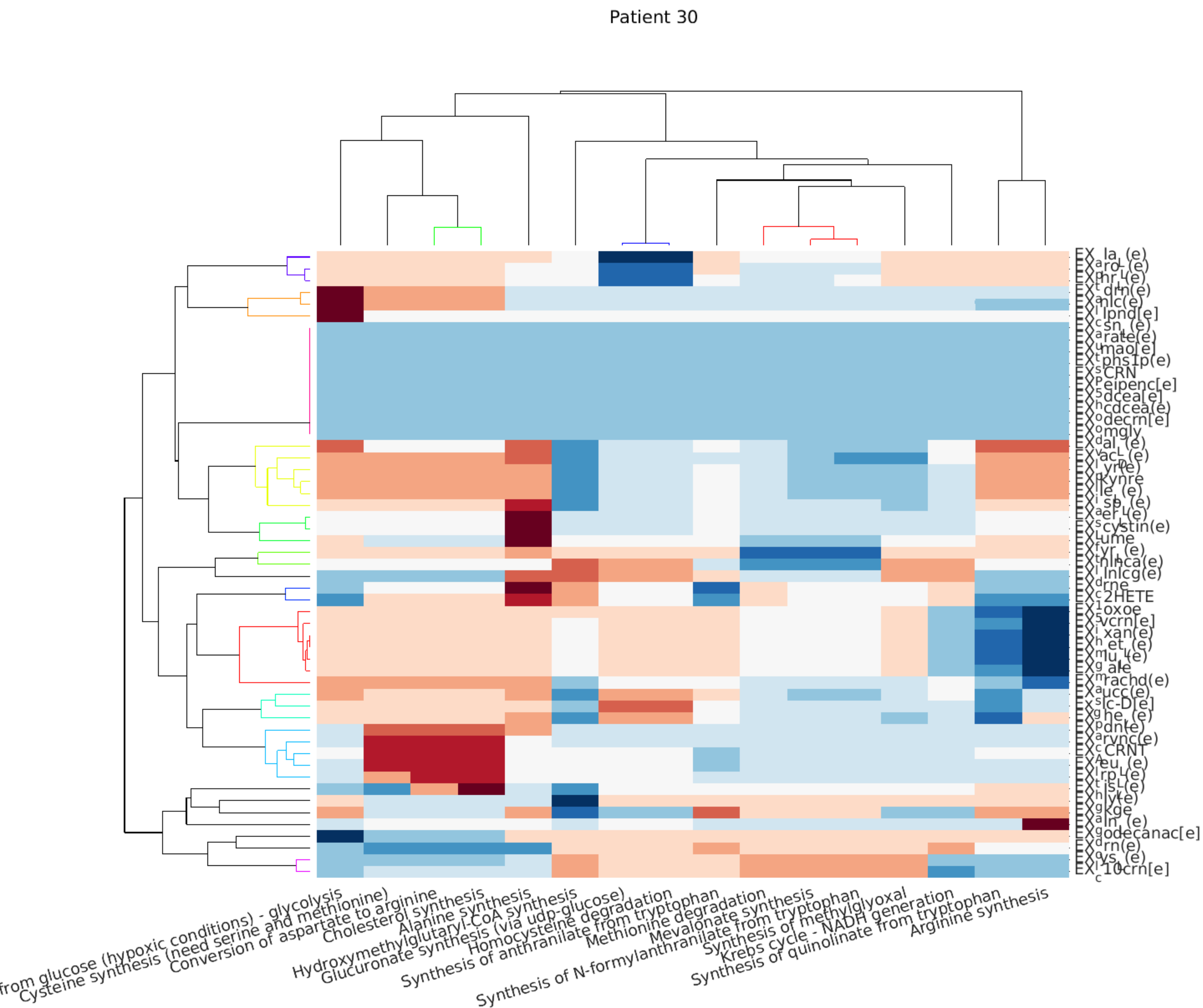
Patient 30
from glucose (hypoxic conditions) - glycolysis
Cysteine synthesis (need serine and methionine)
Conversion of aspartate to arginine
Cholesterol synthesis
Alanine synthesis
Hydroxymethylglutaryl-CoA synthesis
Glucuronate synthesis (via udp-glucose)
Homocysteine degradation
Synthesis of anthranilate from tryptophan
Methionine degradation
Synthesis of N-formylanthranilate from tryptophan
Mevalonate synthesis
Synthesis of methylglyoxal
Krebs cycle - NADH generation
Synthesis of quinolinate from tryptophan
Arginine synthesis

Patient 31

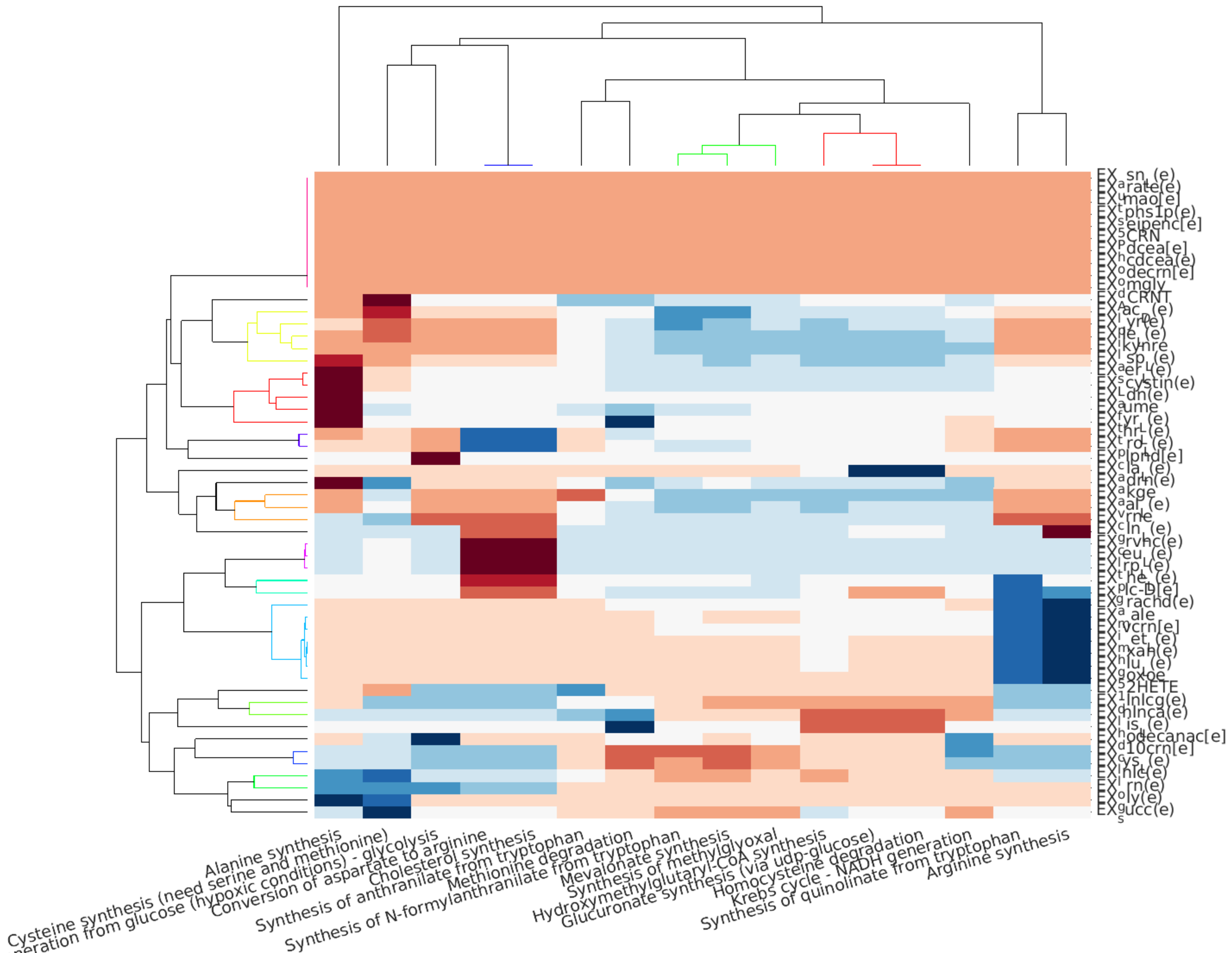
Alanine synthesis
Cysteine synthesis (need serine and methionine)
from glucose (hypoxic conditions) - glycolysis
Conversion of aspartate to arginine
Cholesterol synthesis
Synthesis of anthranilate from tryptophan
Methionine degradation
Synthesis of N-formylanthranilate from tryptophan
Mevalonate synthesis
Synthesis of methylglyoxal
Hydroxymethylglutaryl-CoA synthesis
Glucuronate synthesis (via udp-glucose)
Homocysteine degradation
Krebs cycle - NADH generation
Synthesis of quinolinate from tryptophan
Arginine synthesis

Patient 32

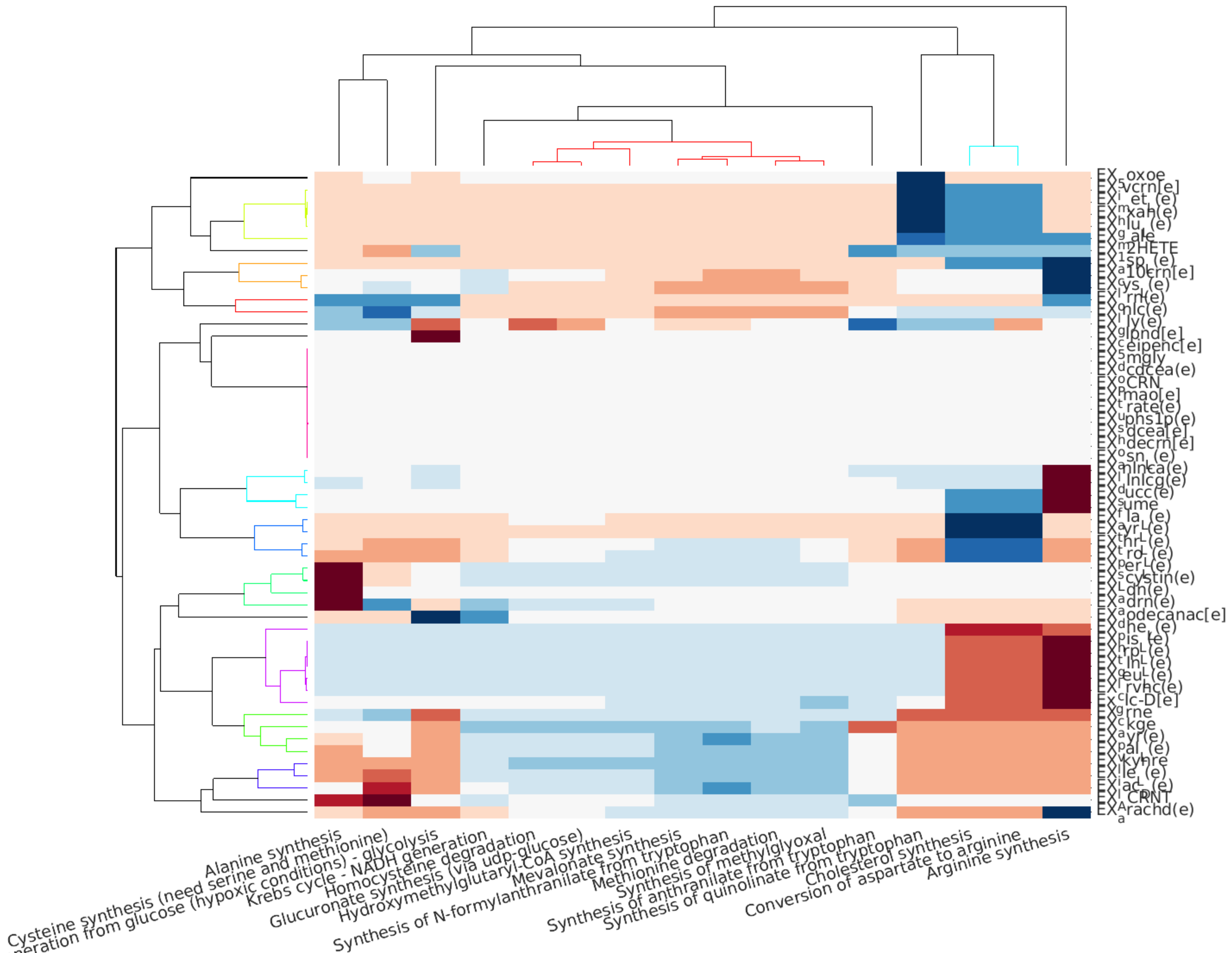
Alanine synthesis
Cysteine synthesis (need serine and methionine)
eration from glucose (hypoxic conditions) - glycolysis
Krebs cycle - NADH generation
Homocysteine degradation
Glucuronate synthesis (via udp-glucose)
Hydroxymethylglutaryl-CoA synthesis
Synthesis of N-formylanthranilate from tryptophan
Mevalonate synthesis
Methionine degradation
Synthesis of methylglyoxal
Synthesis of anthranilate from tryptophan
Synthesis of quinolinate from tryptophan
Cholesterol synthesis
Conversion of aspartate to arginine
Arginine synthesis

Patient 33

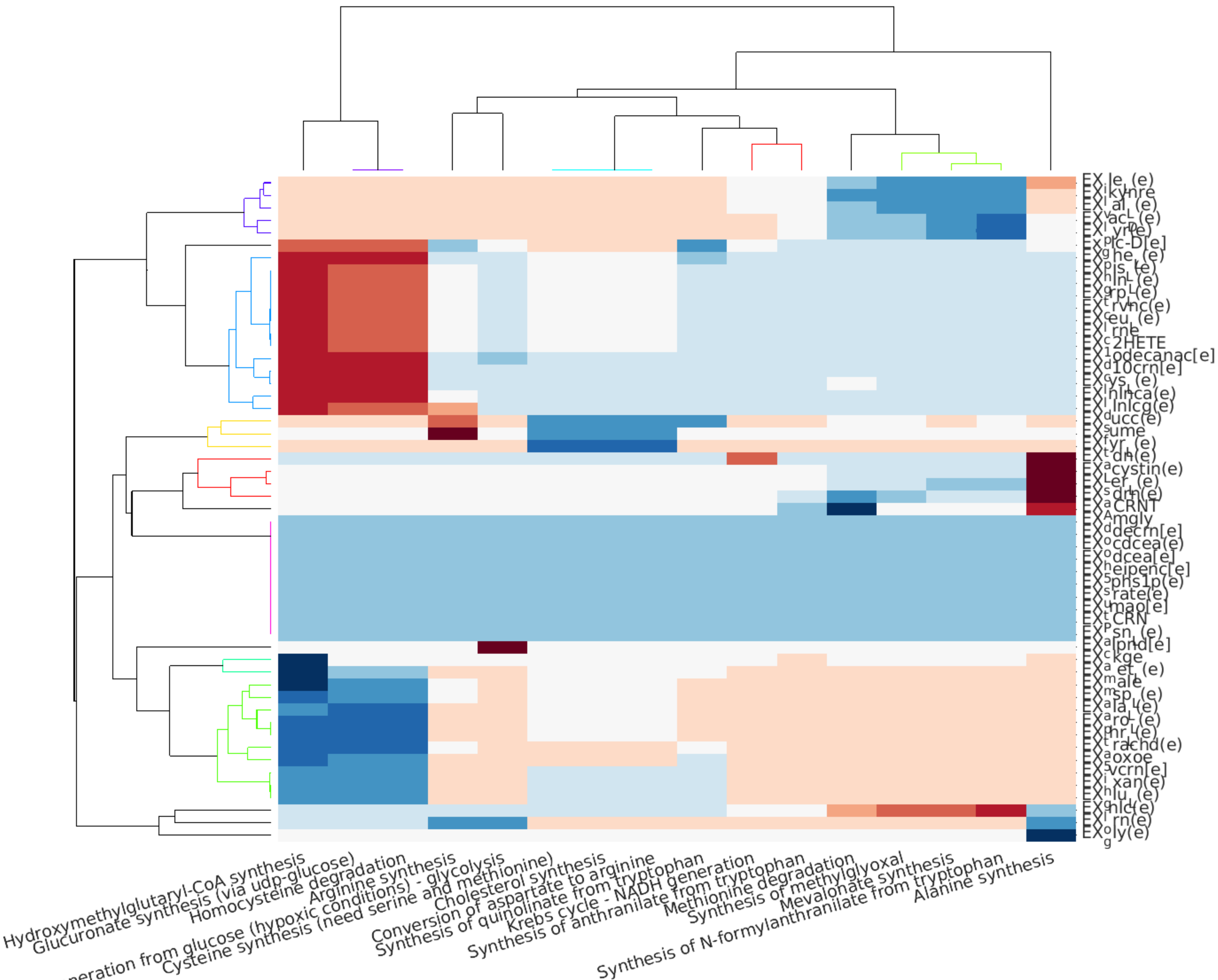

Hydroxymethylglutaryl-CoA synthesis
Glucuronate synthesis (via udp-glucose)
Homocysteine degradation
Arginine synthesis
Cysteine synthesis (need serine and methionine)
Cholesterol synthesis
Conversion of aspartate to arginine
Synthesis of quinolinate from tryptophan
Krebs cycle - NADH generation
Synthesis of anthranilate from tryptophan
Methionine degradation
Synthesis of methylglyoxal
Mevalonate synthesis
Synthesis of N-formylanthranilate from tryptophan
Alanine synthesis

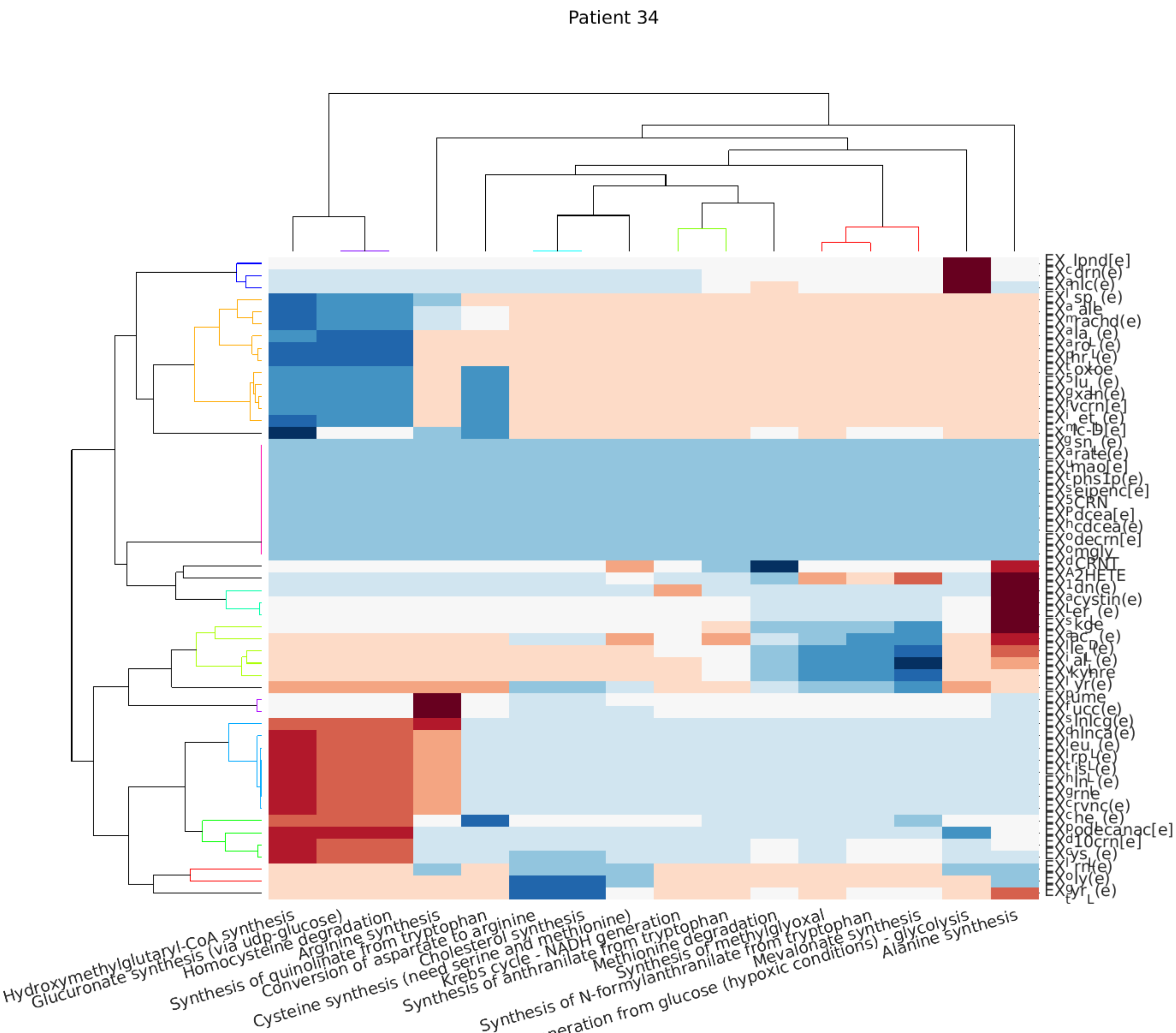
Patient 34
Hydroxymethylglutaryl-CoA synthesis
Glucuronate synthesis (via udp-glucose)
Homocysteine degradation
Arginine synthesis
Synthesis of quinolinate from tryptophan
Conversion of aspartate to arginine
Cholesterol synthesis
Cysteine synthesis (need serine and methionine)
Krebs cycle - NADH generation
Synthesis of anthranilate from tryptophan
Methionine degradation
Synthesis of methylglyoxal
Synthesis of N-formylanthranilate from tryptophan
Mevalonate synthesis
Alanine synthesis

Patient 35

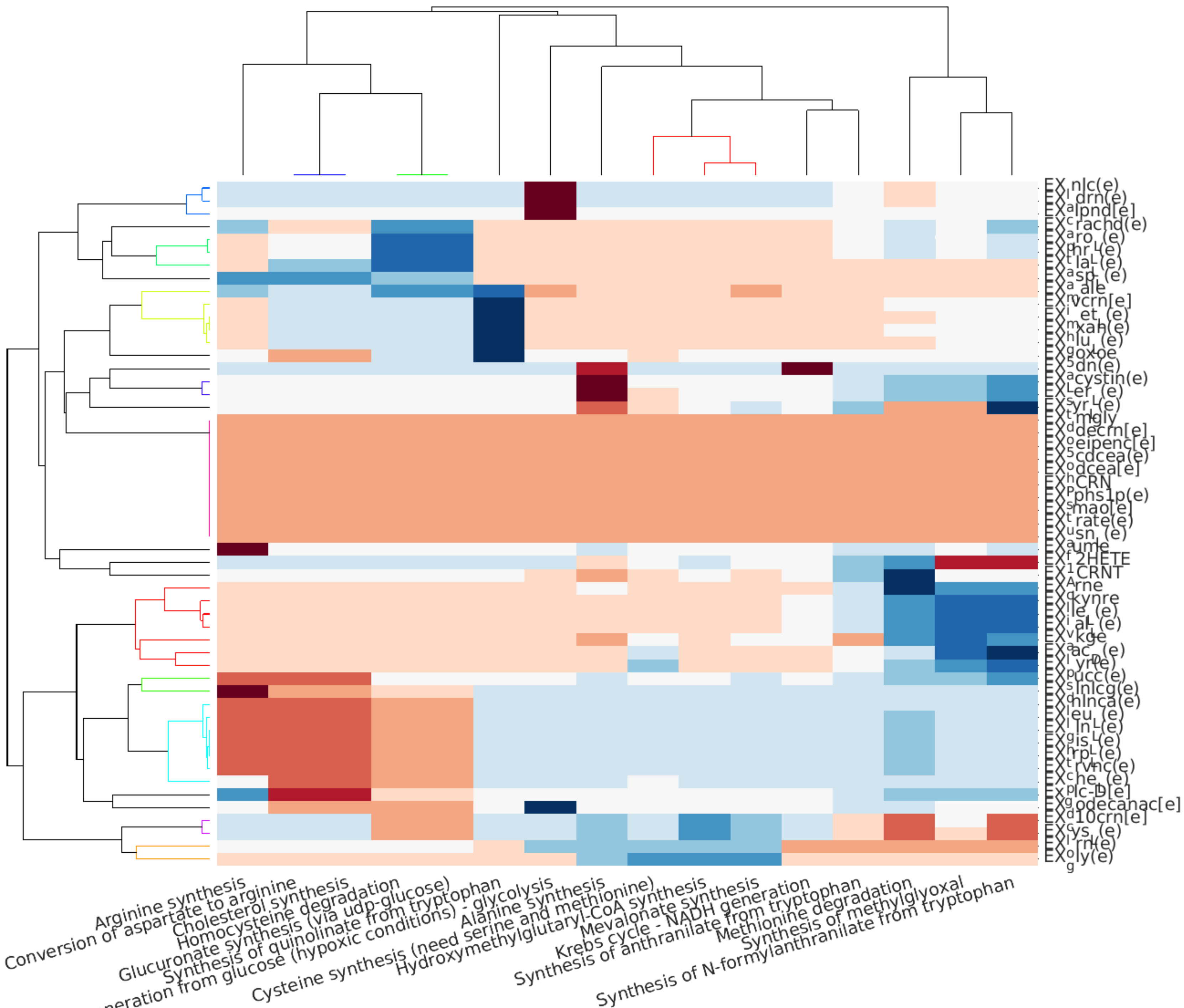
Arginine synthesis
Conversion of aspartate to arginine
Cholesterol synthesis
Homocysteine degradation
Glucuronate synthesis (via udp-glucose)
Synthesis of quinolinate from tryptophan
Cysteine synthesis (need serine and methionine)
Alanine synthesis
Hydroxymethylglutaryl-CoA synthesis
Mevalonate synthesis
Krebs cycle - NADH generation
Synthesis of anthranilate from tryptophan
Methionine degradation
Synthesis of methylglyoxal
Synthesis of N-formylanthranilate from tryptophan

Patient 36

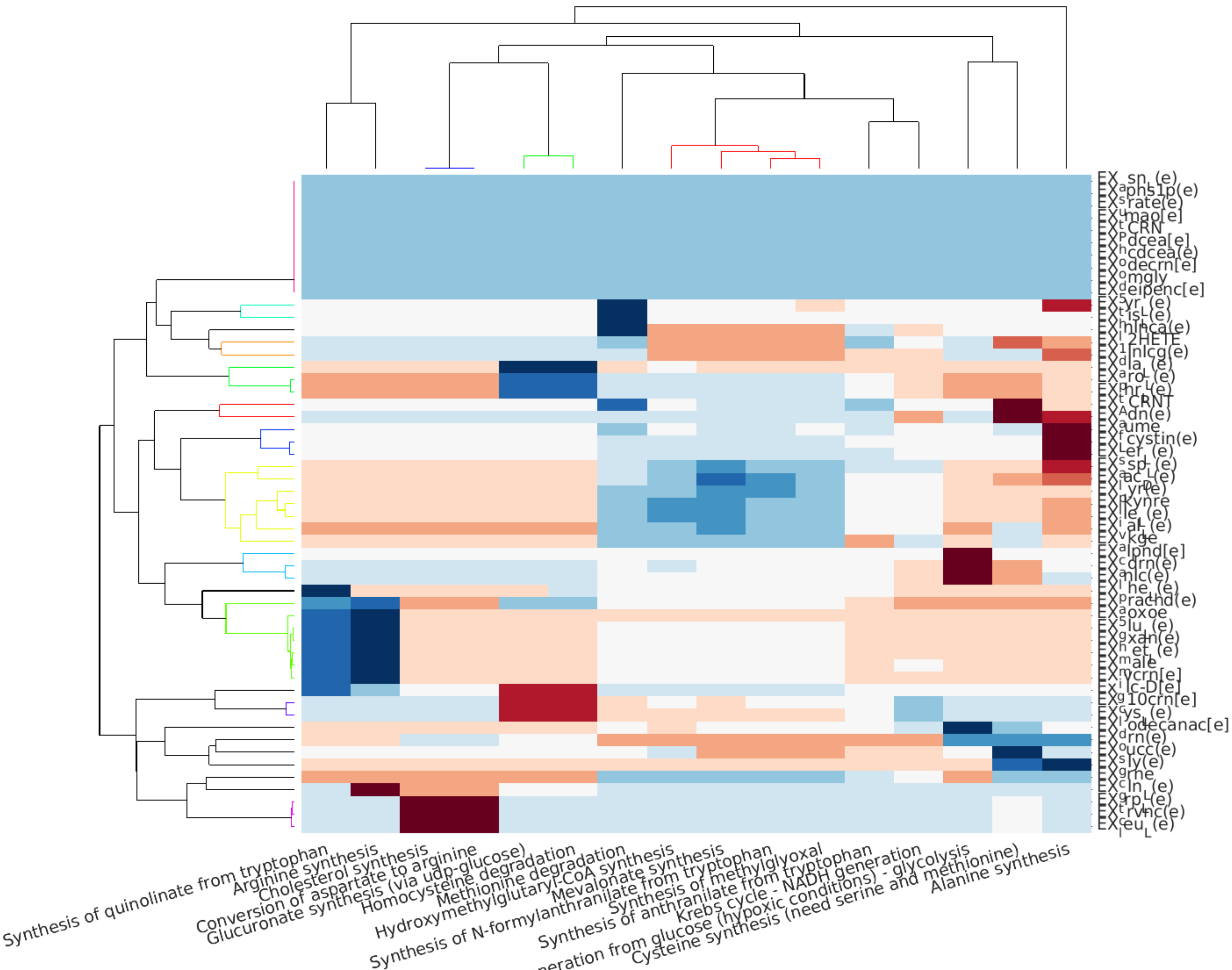
Synthesis of quinolinate from tryptophan
Arginine synthesis
Cholesterol synthesis
Conversion of aspartate to arginine
Glucuronate synthesis (via udp-glucose)
Homocysteine degradation
Methionine degradation
Hydroxymethylglutaryl-CoA synthesis
Mevalonate synthesis
Synthesis of N-formylanthranilate from tryptophan
Synthesis of methylglyoxal
Synthesis of anthranilate from tryptophan
Krebs cycle - NADH generation
neration from glucose (hypoxic conditions) - glycolysis
Cysteine synthesis (need serine and methionine)
Alanine synthesis

Patient 37

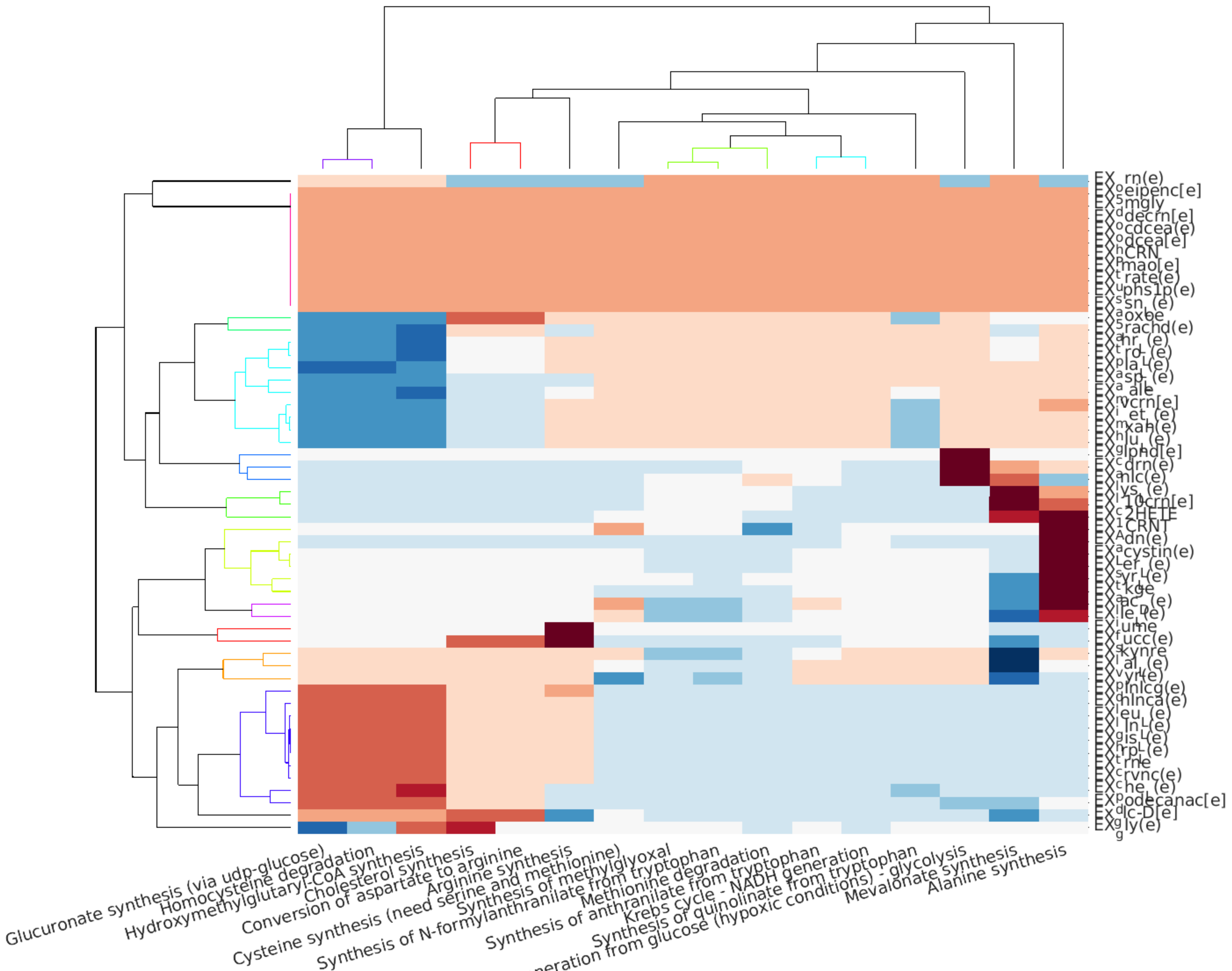
Glucuronate synthesis (via udp-glucose)
Homocysteine degradation
Hydroxymethylglutaryl-CoA synthesis
Cholesterol synthesis
Conversion of aspartate to arginine
Arginine synthesis
Cysteine synthesis (need serine and methionine)
Synthesis of methylglyoxal
Synthesis of N-formylanthranilate from tryptophan
Methionine degradation
Synthesis of anthranilate from tryptophan
Krebs cycle - NADH generation
Synthesis of quinolinate from tryptophan
Mevalonate synthesis
Alanine synthesis

Patient 38

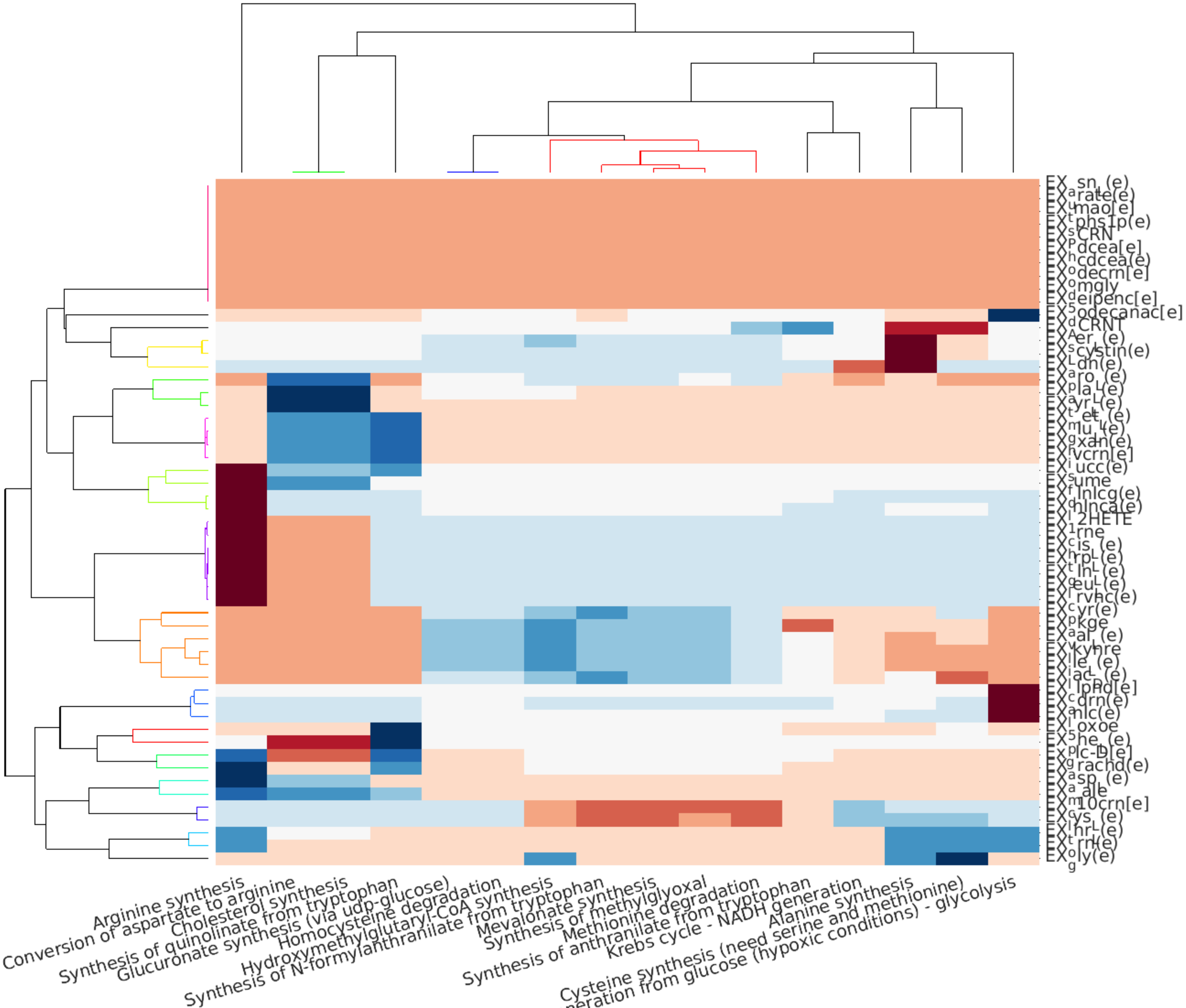
Arginine synthesis
Conversion of aspartate to arginine
Cholesterol synthesis
Synthesis of quinolinate from tryptophan
Glucuronate synthesis (via udp-glucose)
Homocysteine degradation
Hydroxymethylglutaryl-CoA synthesis
Synthesis of N-formylanthranilate from tryptophan
Mevalonate synthesis
Synthesis of methylglyoxal
Methionine degradation
Synthesis of anthranilate from tryptophan
Krebs cycle - NADH generation
Alanine synthesis
Cysteine synthesis (need serine and methionine)
glucose (hypoxic conditions) - glycolysis

Patient 39

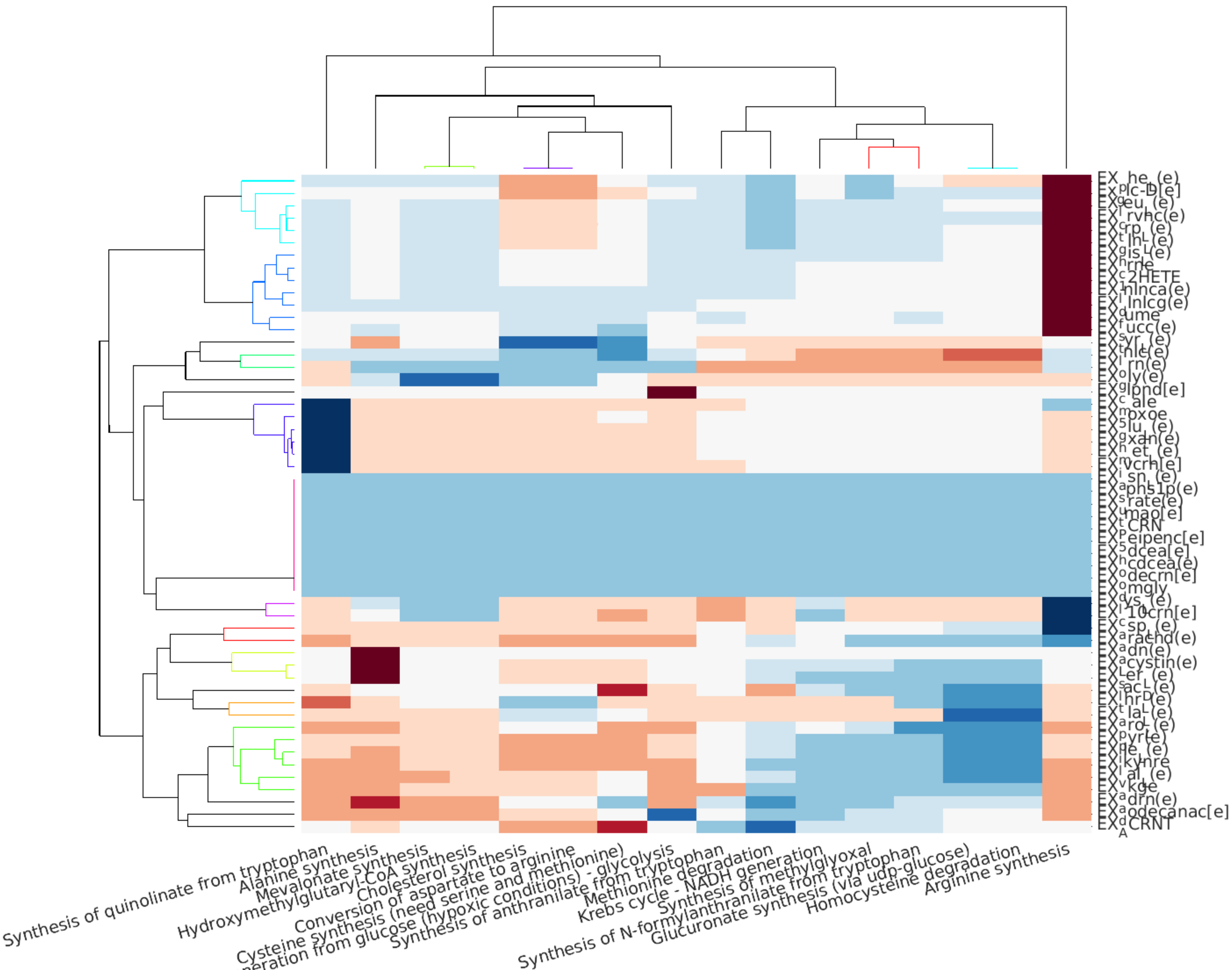
Synthesis of quinolinate from tryptophan
Alanine synthesis
Mevalonate synthesis
Hydroxymethylglutaryl-CoA synthesis
Cholesterol synthesis
Conversion of aspartate to arginine
Cysteine synthesis (need serine and methionine)
Synthesis of anthranilate from tryptophan
Methionine degradation
Krebs cycle - NADH generation
Synthesis of methylglyoxal
Synthesis of N-formylanthranilate from tryptophan
Glucuronate synthesis (via udp-glucose)
Homocysteine degradation
Arginine synthesis

Patient 40

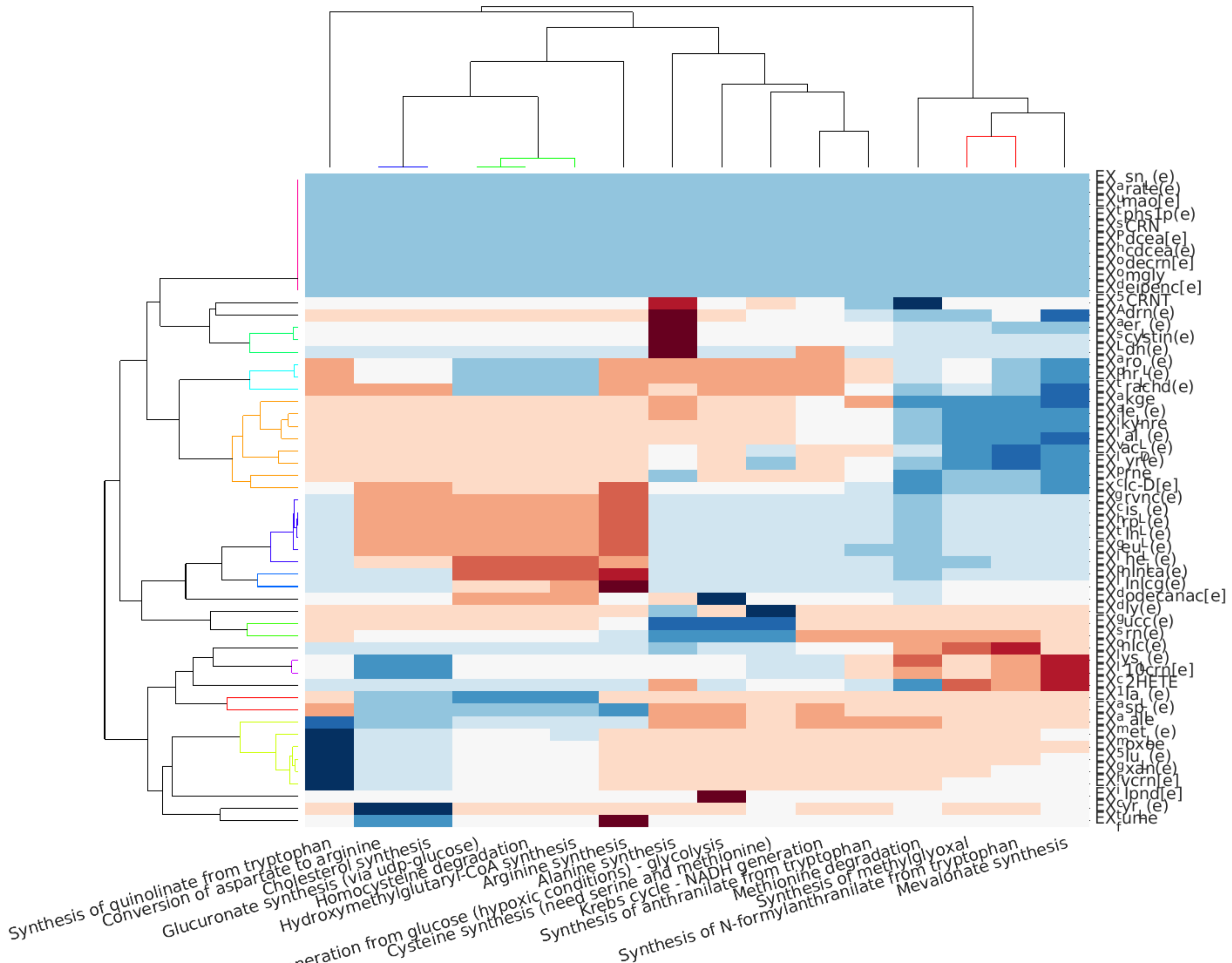

Synthesis of quinolinate from tryptophan
Conversion of aspartate to arginine
Cholesterol synthesis
Glucuronate synthesis (via udp-glucose)
Homocysteine degradation
Hydroxymethylglutaryl-CoA synthesis
Arginine synthesis
Alanine synthesis
Cysteine synthesis (need serine and methionine)
Krebs cycle - NADH generation
Synthesis of anthranilate from tryptophan
Methionine degradation
Synthesis of methylglyoxal
Synthesis of N-formylanthranilate from tryptophan
Mevalonate synthesis

Patient 41

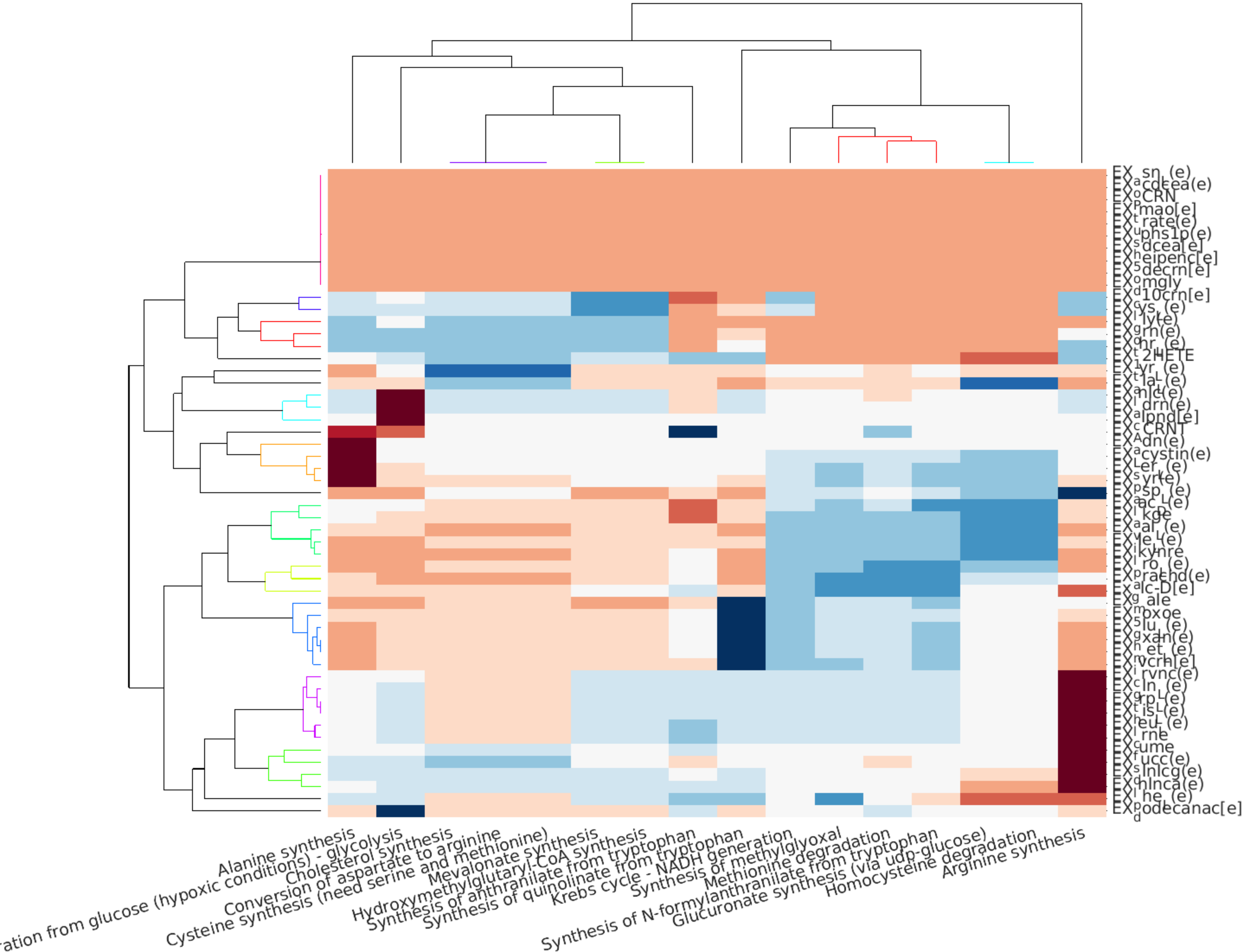
Alanine synthesis
ration from glucose (hypoxic conditions) - glycolysis
Cholesterol synthesis
Conversion of aspartate to arginine
Cysteine synthesis (need serine and methionine)
Mevalonate synthesis
Hydroxymethylglutaryl-CoA synthesis
Synthesis of anthranilate from tryptophan
Synthesis of quinolinate from tryptophan
Krebs cycle - NADH generation
Synthesis of methylglyoxal
Methionine degradation
Synthesis of N-formylanthranilate from tryptophan
Glucuronate synthesis (via udp-glucose)
Homocysteine degradation
Arginine synthesis

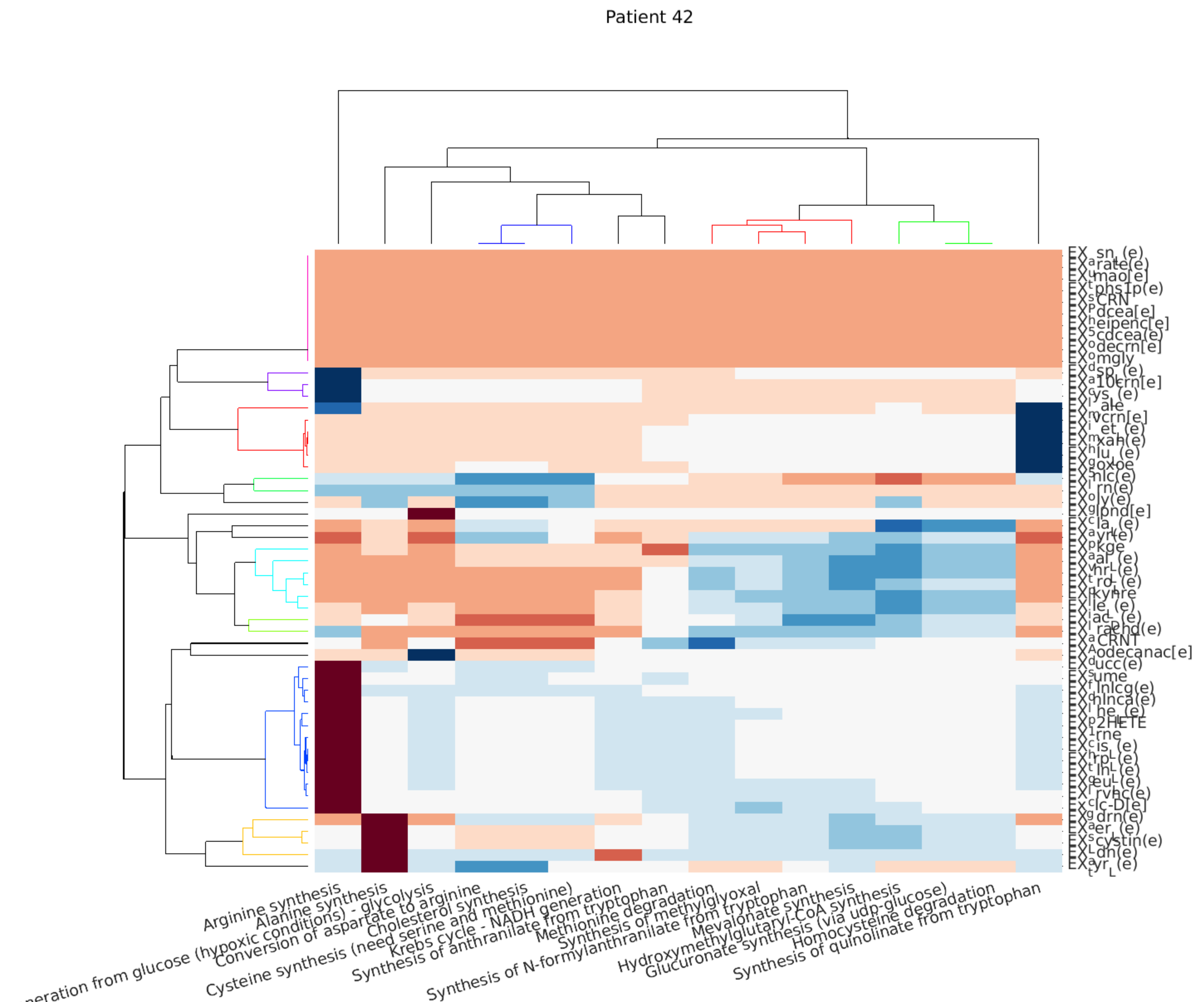
Patient 42
Arginine synthesis
Alanine synthesis
eration from glucose (hypoxic conditions) - glycolysis
Conversion of aspartate to arginine
Cholesterol synthesis
Cysteine synthesis (need serine and methionine)
Krebs cycle - NADH generation
Synthesis of anthranilate from tryptophan
Methionine degradation
Synthesis of N-formylanthranilate from tryptophan
Synthesis of methylglyoxal
Hydroxymethylglutaryl-CoA synthesis
Mevalonate synthesis
Glucuronate synthesis (via udp-glucose)
Homocysteine degradation
Synthesis of quinolinate from tryptophan

Patient 43

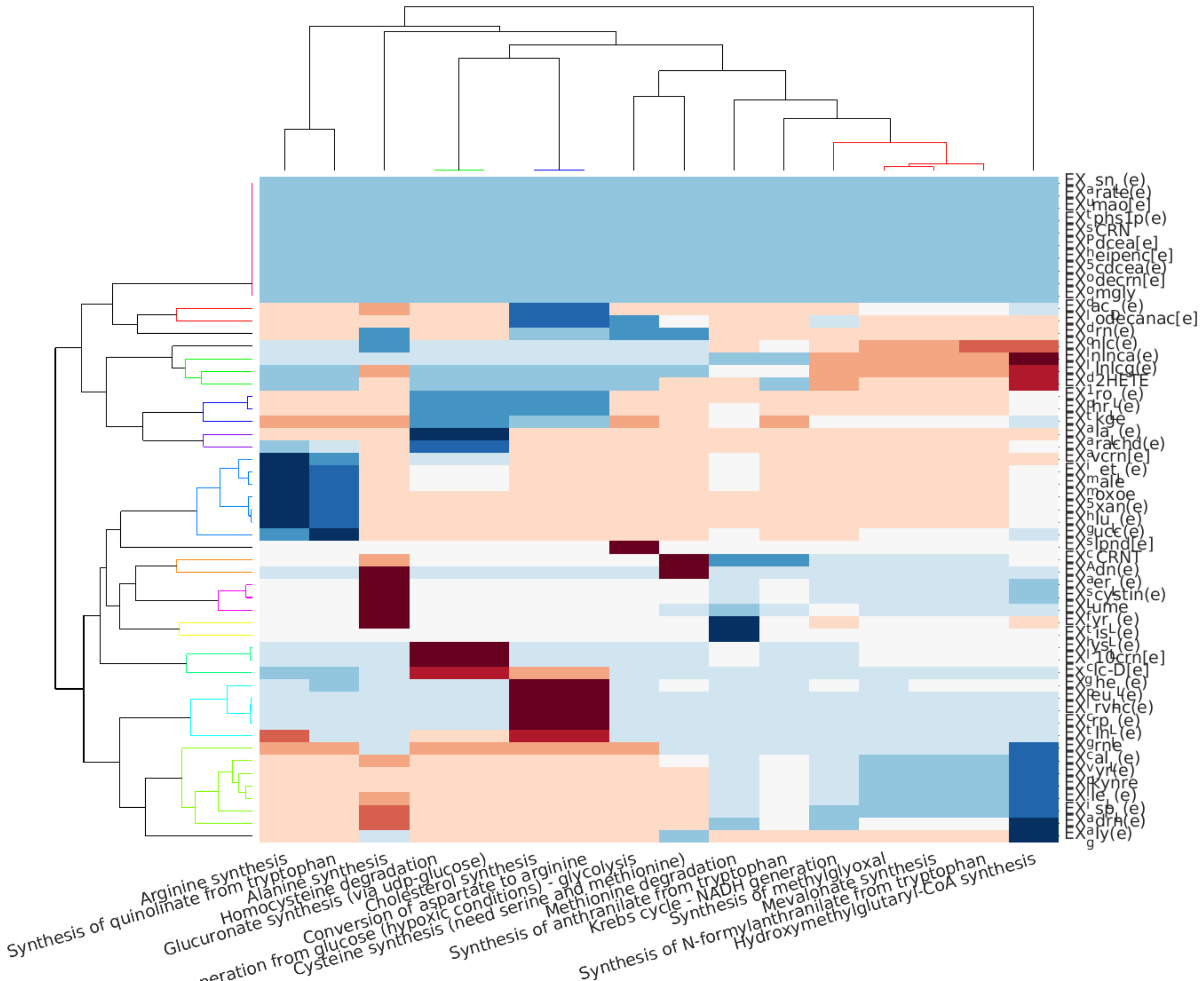

Arginine synthesis
Synthesis of quinolinate from tryptophan
Alanine synthesis
Homocysteine degradation
Glucuronate synthesis (via udp-glucose)
Cholesterol synthesis
Conversion of aspartate to arginine
eration from glucose (hypoxic conditions) - glycolysis
Cysteine synthesis (need serine and methionine)
Methionine degradation
Synthesis of anthranilate from tryptophan
Krebs cycle - NADH generation
Synthesis of methylglyoxal
Mevalonate synthesis
Synthesis of N-formylanthranilate from tryptophan
Hydroxymethylglutaryl-CoA synthesis

Patient 44

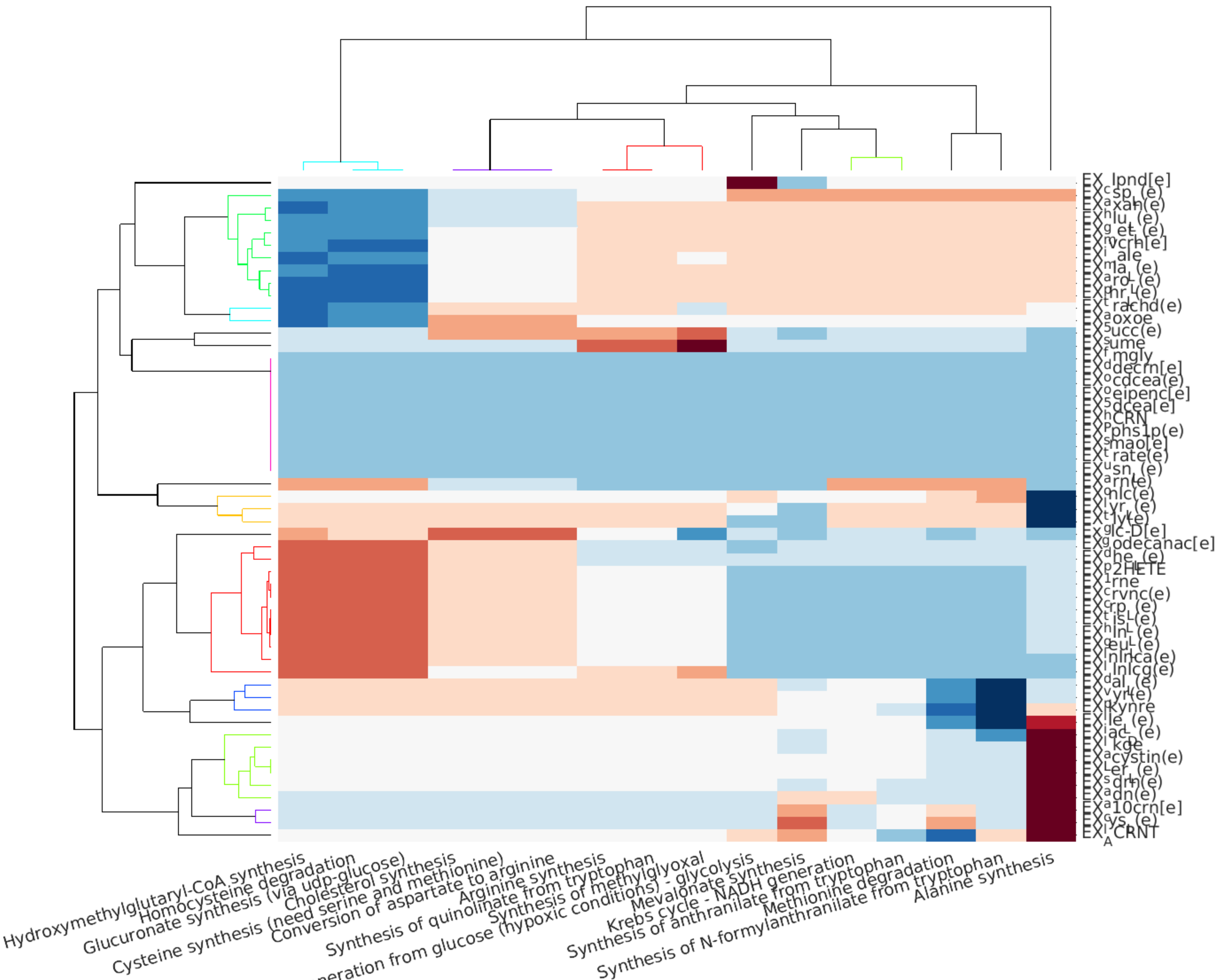
Hydroxymethylglutaryl-CoA synthesis
Homocysteine degradation
Glucuronate synthesis (via udp-glucose)
Cholesterol synthesis
Cysteine synthesis (need serine and methionine)
Conversion of aspartate to arginine
Arginine synthesis
Synthesis of quinolinate from tryptophan
Synthesis of methylglyoxal
from glucose (hypoxic conditions) - glycolysis
Mevalonate synthesis
Krebs cycle - NADH generation
Synthesis of anthranilate from tryptophan
Methionine degradation
Synthesis of N-formylanthranilate from tryptophan
Alanine synthesis

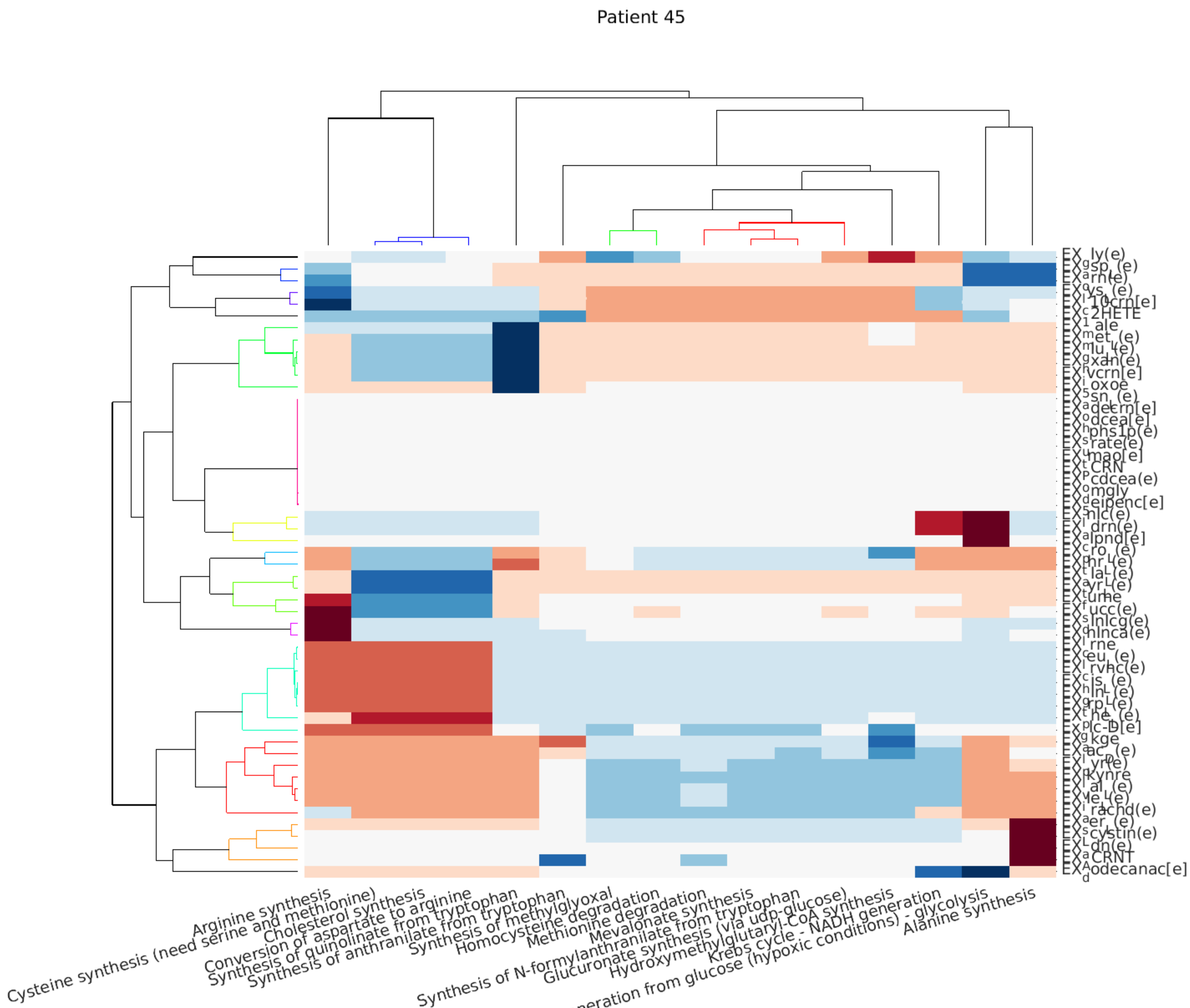
Patient 45
Arginine synthesis
Cysteine synthesis (need serine and methionine)
Cholesterol synthesis
Conversion of aspartate to arginine
Synthesis of quinolinate from tryptophan
Synthesis of anthranilate from tryptophan
Synthesis of methylglyoxal
Homocysteine degradation
Methionine degradation
Mevalonate synthesis
Synthesis of N-formylanthranilate from tryptophan
Glucuronate synthesis (via udp-glucose)
Hydroxymethylglutaryl-CoA synthesis
Krebs cycle - NADH generation
Alanine synthesis

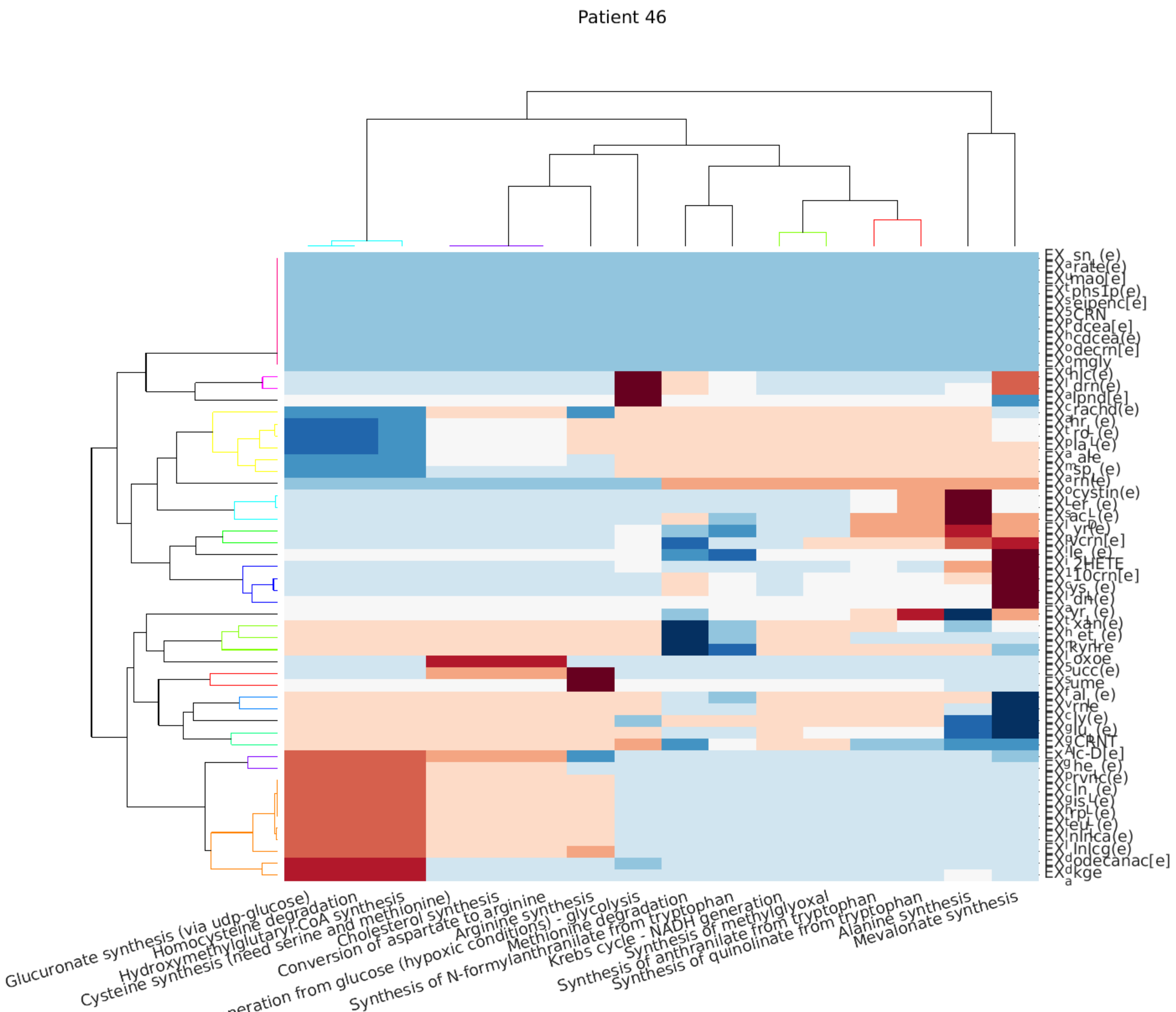

Patient 46
Glucuronate synthesis (via udp-glucose)
Homocysteine degradation
Hydroxymethylglutaryl-CoA synthesis
Cysteine synthesis (need serine and methionine)
Cholesterol synthesis
Conversion of aspartate to arginine
Arginine synthesis
from glucose (hypoxic conditions) - glycolysis
Methionine degradation
Synthesis of N-formylanthranilate from tryptophan
Krebs cycle - NADH generation
Synthesis of methylglyoxal
Synthesis of anthranilate from tryptophan
Synthesis of quinolinate from tryptophan
Alanine synthesis
Mevalonate synthesis

Patient 47

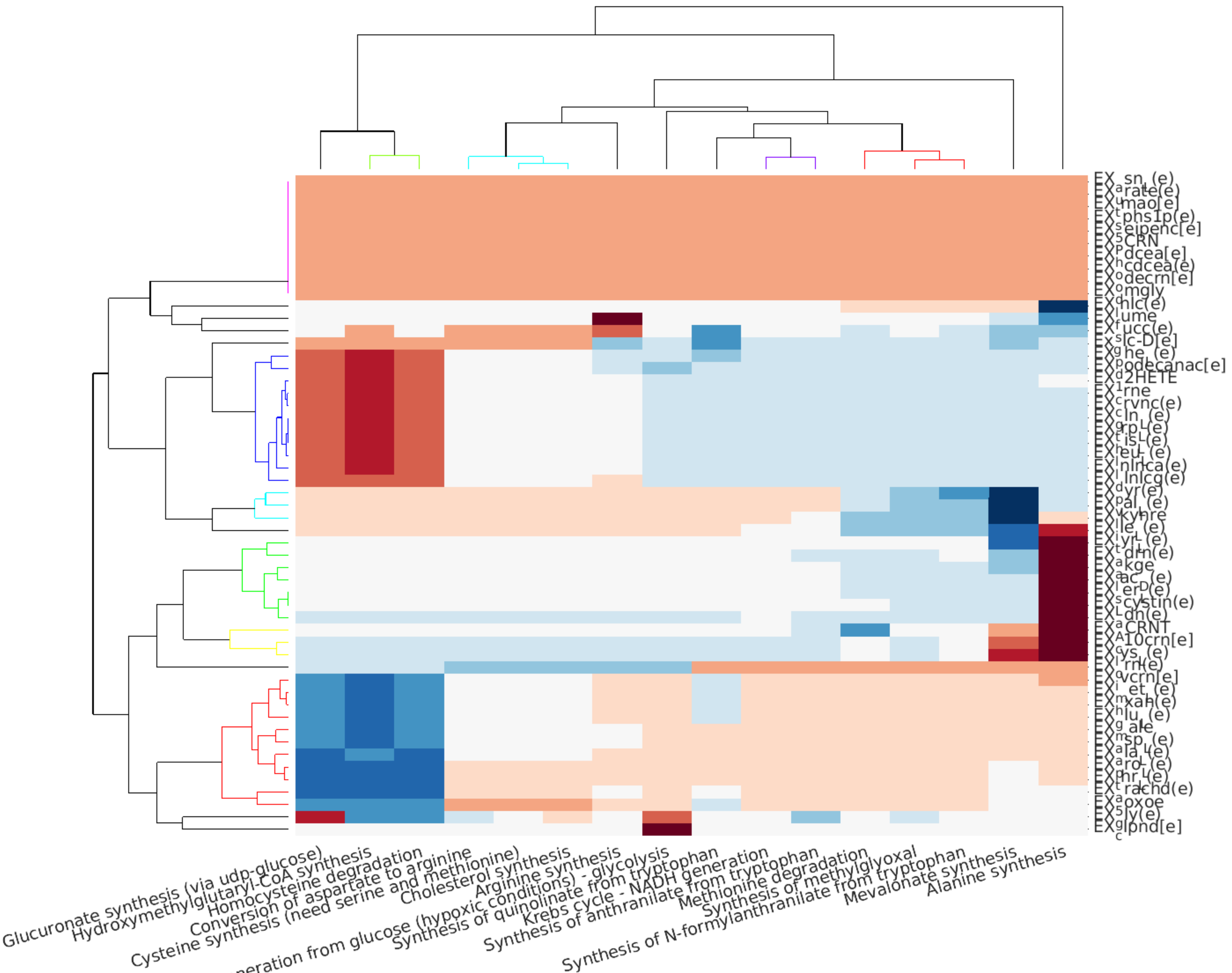
Glucuronate synthesis (via udp-glucose)
Hydroxymethylglutaryl-CoA synthesis
Homocysteine degradation
Conversion of aspartate to arginine
Cysteine synthesis (need serine and methionine)
Cholesterol synthesis
Arginine synthesis
Synthesis of quinolinate from tryptophan
Krebs cycle - NADH generation
Synthesis of anthranilate from tryptophan
Methionine degradation
Synthesis of methylglyoxal
Synthesis of N-formylanthranilate from tryptophan
Mevalonate synthesis
Alanine synthesis

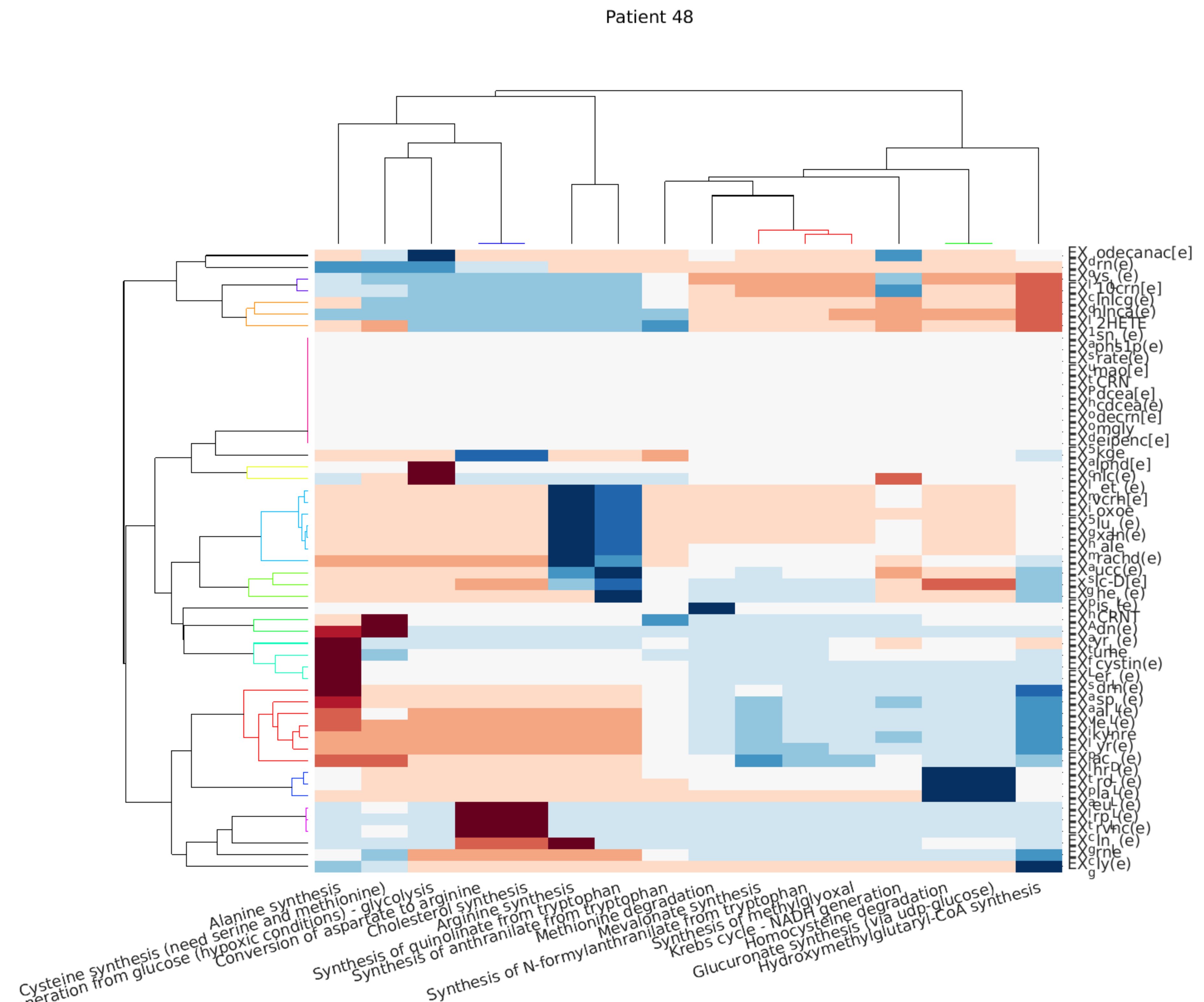
Patient 48
Alanine synthesis
Cysteine synthesis (need serine and methionine)
eration from glucose (hypoxic conditions) - glycolysis
Conversion of aspartate to arginine
Cholesterol synthesis
Arginine synthesis
Synthesis of quinolinate from tryptophan
Synthesis of anthranilate from tryptophan
Methionine degradation
Synthesis of N-formylanthranilate from tryptophan
Mevalonate synthesis
Synthesis of methylglyoxal
Krebs cycle - NADH generation
Homocysteine degradation
Glucuronate synthesis (via udp-glucose)
Hydroxymethylglutaryl-CoA synthesis

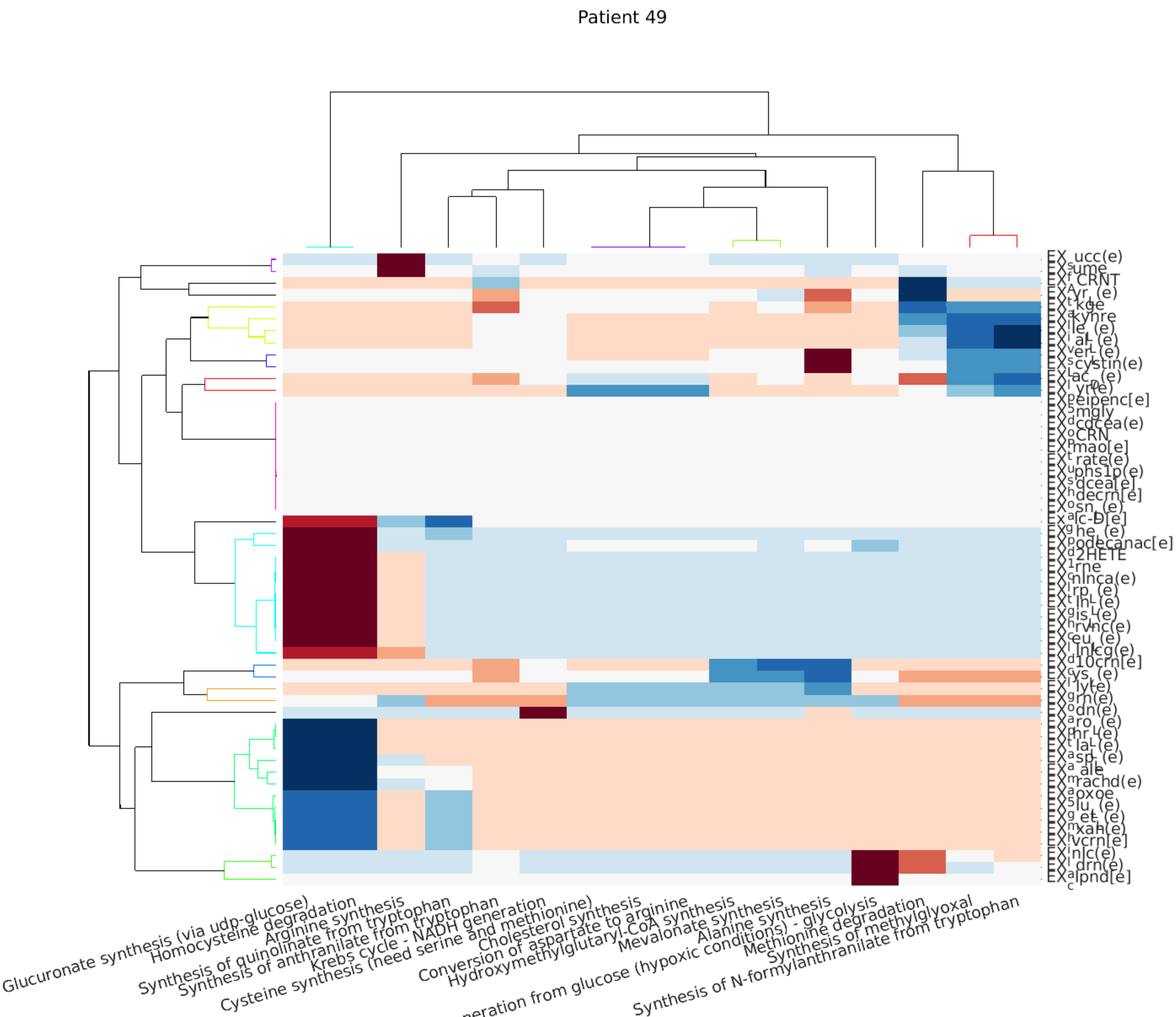
Patient 49
Glucuronate synthesis (via udp-glucose)
Homocysteine degradation
Arginine synthesis
Synthesis of quinolinate from tryptophan
Synthesis of anthranilate from tryptophan
Krebs cycle - NADH generation
Cysteine synthesis (need serine and methionine)
Cholesterol synthesis
Conversion of aspartate to arginine
Hydroxymethylglutaryl-CoA synthesis
Mevalonate synthesis
Alanine synthesis
neration from glucose (hypoxic conditions) - glycolysis
Methionine degradation
Synthesis of methylglyoxal
Synthesis of N-formylanthranilate from tryptophan
EX_ucc(e)
EX_ume
EX_CRNT
EX_yr_(e)
EX_kge
EX_kynre
EX_le_(e)
EX_al_(e)
EX_ver_(e)
EX_cystin(e)
EX_ac_(e)
EX_yr(e)
EX_eipenc[e]
EX_mgly
EX_cdcea(e)
EX_CRN
EX_mao[e]
EX_rate(e)
EX_phs1p(e)
EX_dcea[e]
EX_decrn[e]
EX_sn_(e)
EX_c-D[e]
EX_he_(e)
EX_odecanac[e]
EX_2HETE
EX_rne
EX_nlnca(e)
EX_rp_(e)
EX_ln_(e)
EX_is_(e)
EX_rvnc(e)
EX_eu_(e)
EX_lnlcg(e)
EX_10crn[e]
EX_ys_(e)
EX_lyte
EX_rn(e)
EX_dn(e)
EX_ro_(e)
EX_hr_(e)
EX_la_(e)
EX_sp_(e)
EX_ale
EX_rachd(e)
EX_oxoe
EX_lu_(e)
EX_et_(e)
EX_xan(e)
EX_vcrn[e]
EX_nlc(e)
EX_drn(e)
EX_lpnd[e]

Patient 50

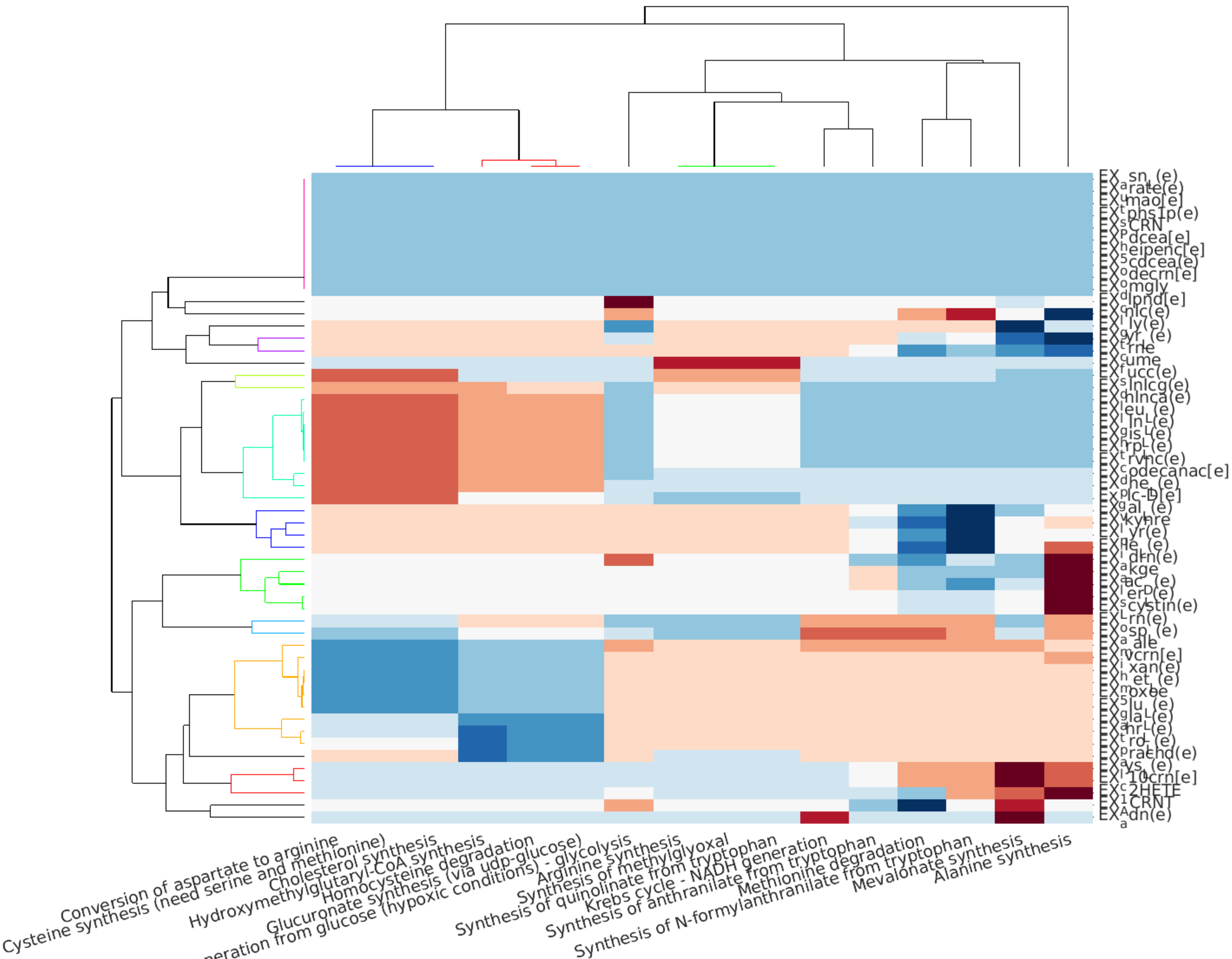

Conversion of aspartate to arginine
Cysteine synthesis (need serine and methionine)
Cholesterol synthesis
Hydroxymethylglutaryl-CoA synthesis
Homocysteine degradation
Glucuronate synthesis (via udp-glucose)
Arginine synthesis
Synthesis of methylglyoxal
Synthesis of quinolinate from tryptophan
Krebs cycle - NADH generation
Synthesis of anthranilate from tryptophan
Methionine degradation
Synthesis of N-formylanthranilate from tryptophan
Mevalonate synthesis
Alanine synthesis

Patient 51

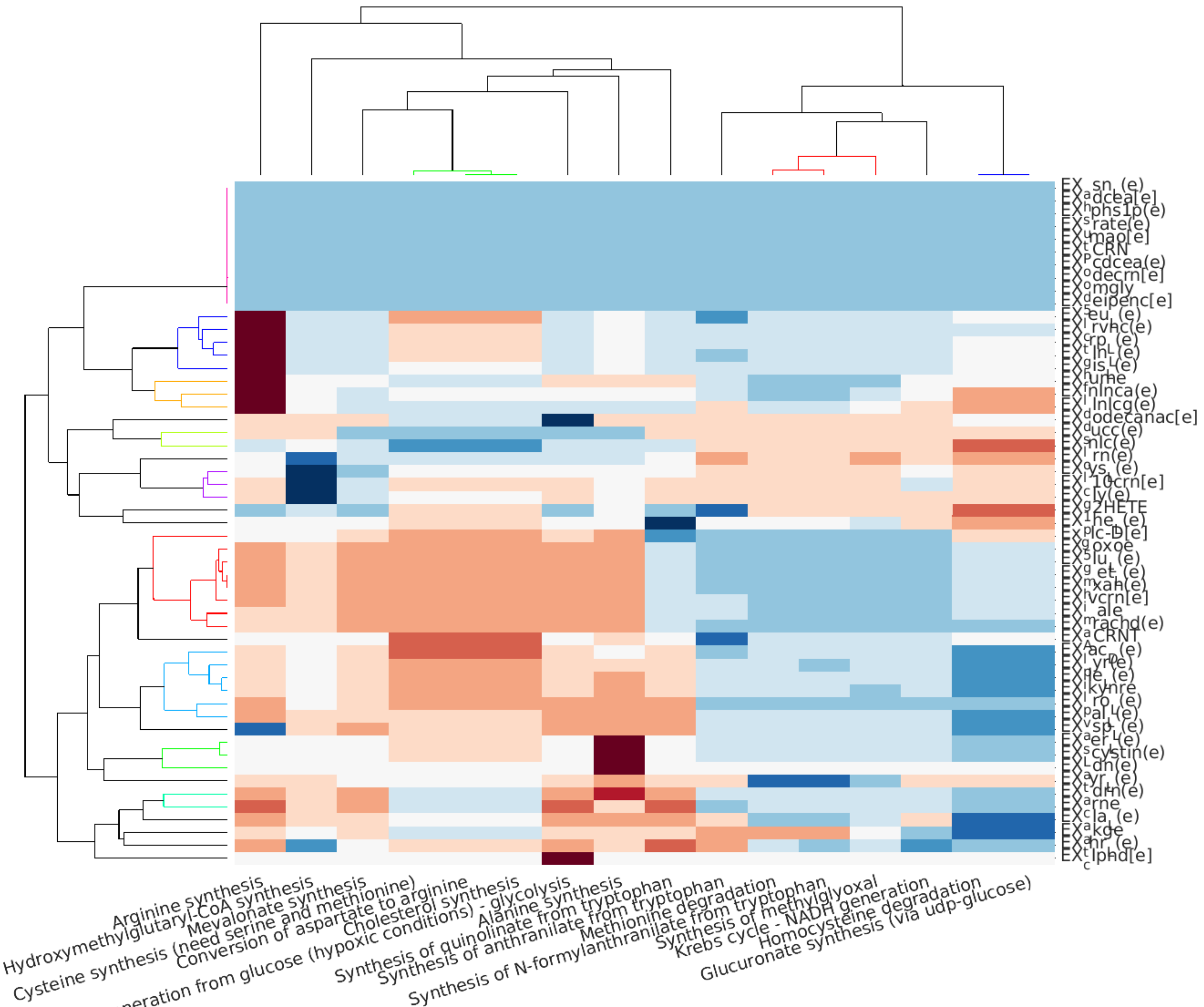
Arginine synthesis
Hydroxymethylglutaryl-CoA synthesis
Mevalonate synthesis
Cysteine synthesis (need serine and methionine)
Conversion of aspartate to arginine
Cholesterol synthesis
Alanine synthesis
Synthesis of quinolinate from tryptophan
Synthesis of anthranilate from tryptophan
Synthesis of N-formylanthranilate from tryptophan
Methionine degradation
Synthesis of methylglyoxal
Krebs cycle - NADH generation
Homocysteine degradation
Glucuronate synthesis (via udp-glucose)

Patient 52

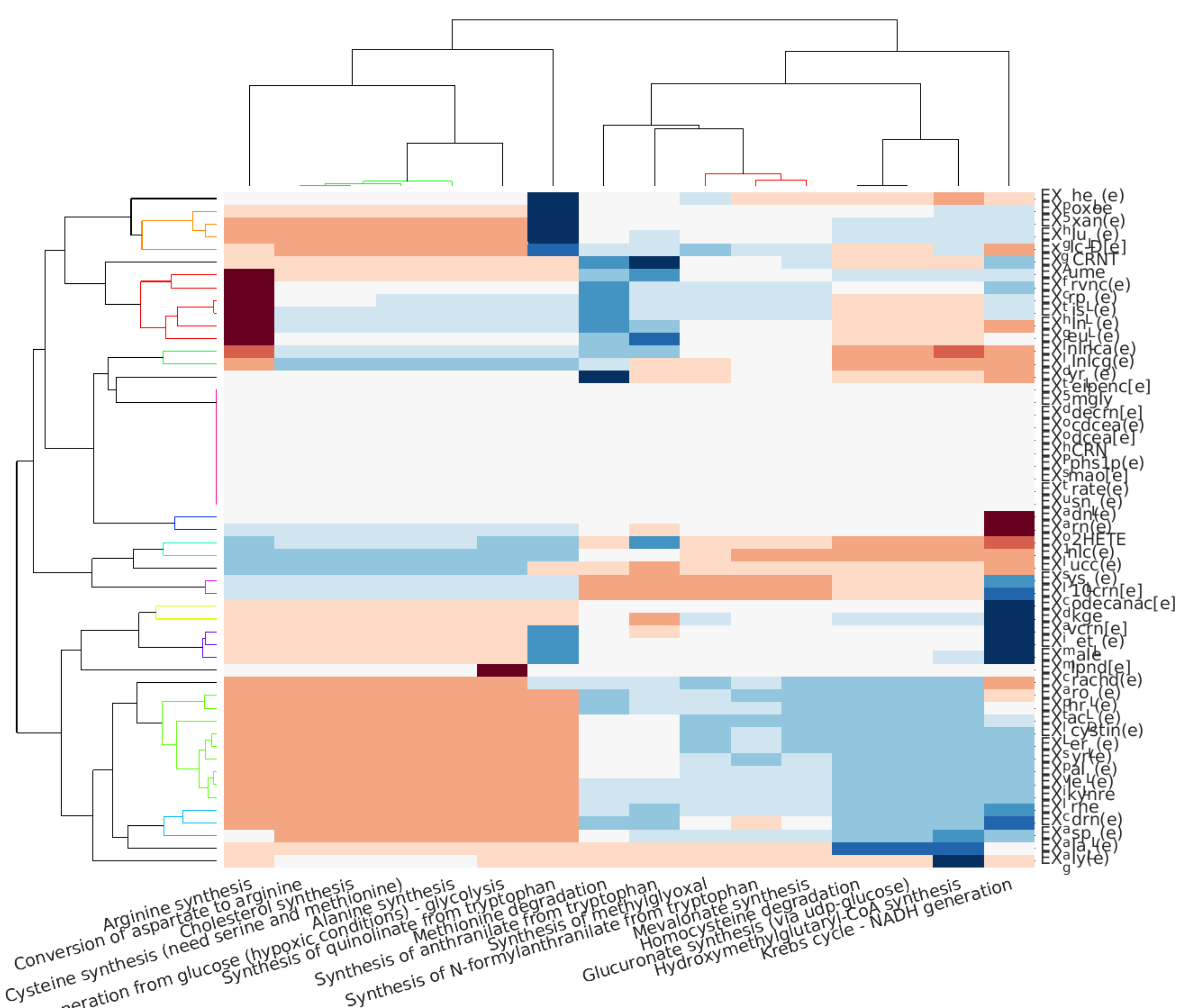
Arginine synthesis
Conversion of aspartate to arginine
Cholesterol synthesis
Cysteine synthesis (need serine and methionine)
Alanine synthesis
Synthesis of quinolinate from tryptophan
Methionine degradation
Synthesis of anthranilate from tryptophan
Synthesis of methylglyoxal
Synthesis of N-formylanthranilate from tryptophan
Mevalonate synthesis
Homocysteine degradation
Glucuronate synthesis (via udp-glucose)
Hydroxymethylglutaryl-CoA synthesis
Krebs cycle - NADH generation

Patient 53

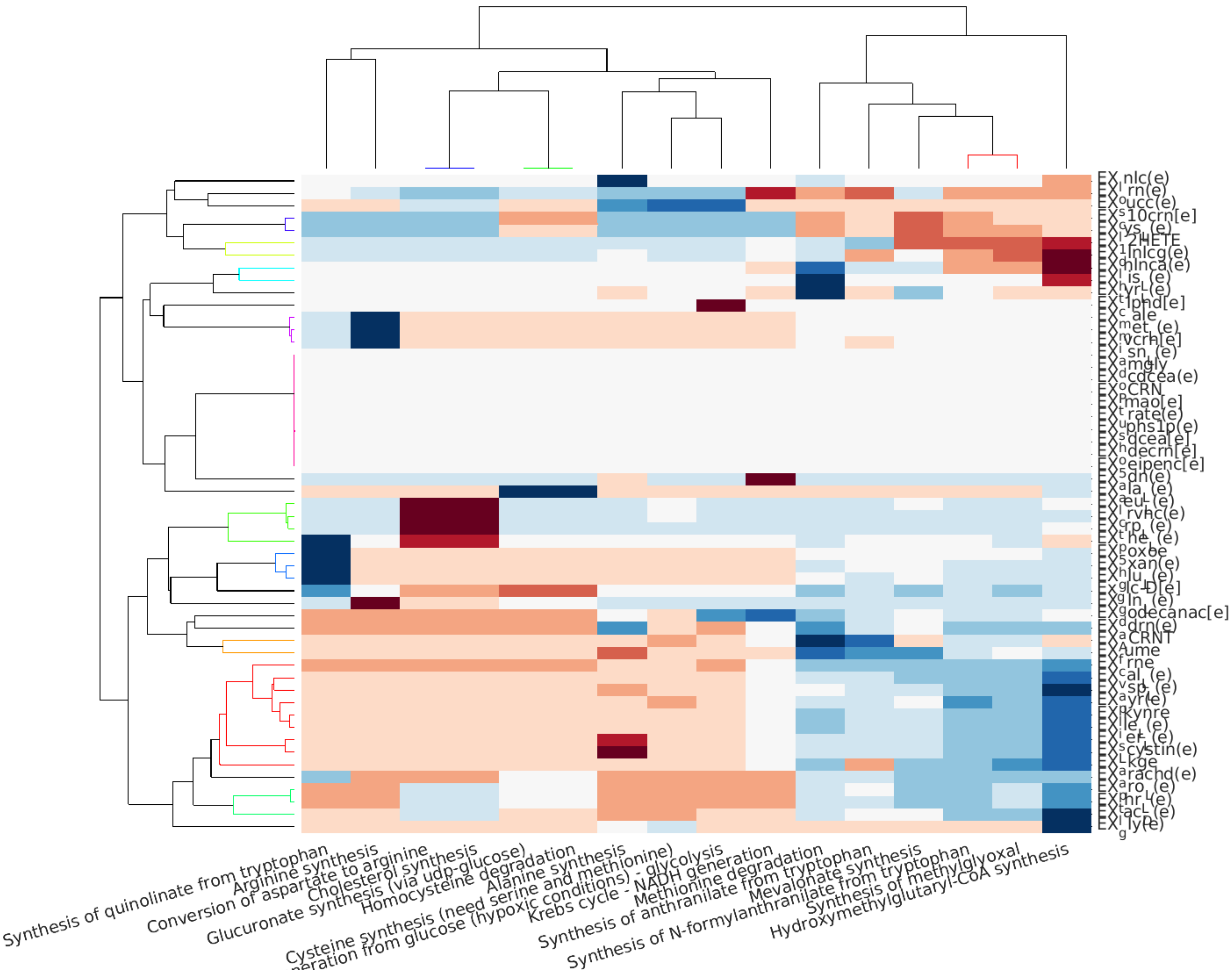

Synthesis of quinolinate from tryptophan
Arginine synthesis
Conversion of aspartate to arginine
Cholesterol synthesis
Glucuronate synthesis (via udp-glucose)
Homocysteine degradation
Alanine synthesis
Cysteine synthesis (need serine and methionine)
Krebs cycle - NADH generation
Methionine degradation
Synthesis of anthranilate from tryptophan
Mevalonate synthesis
Synthesis of N-formylanthranilate from tryptophan
Synthesis of methylglyoxal
Hydroxymethylglutaryl-CoA synthesis

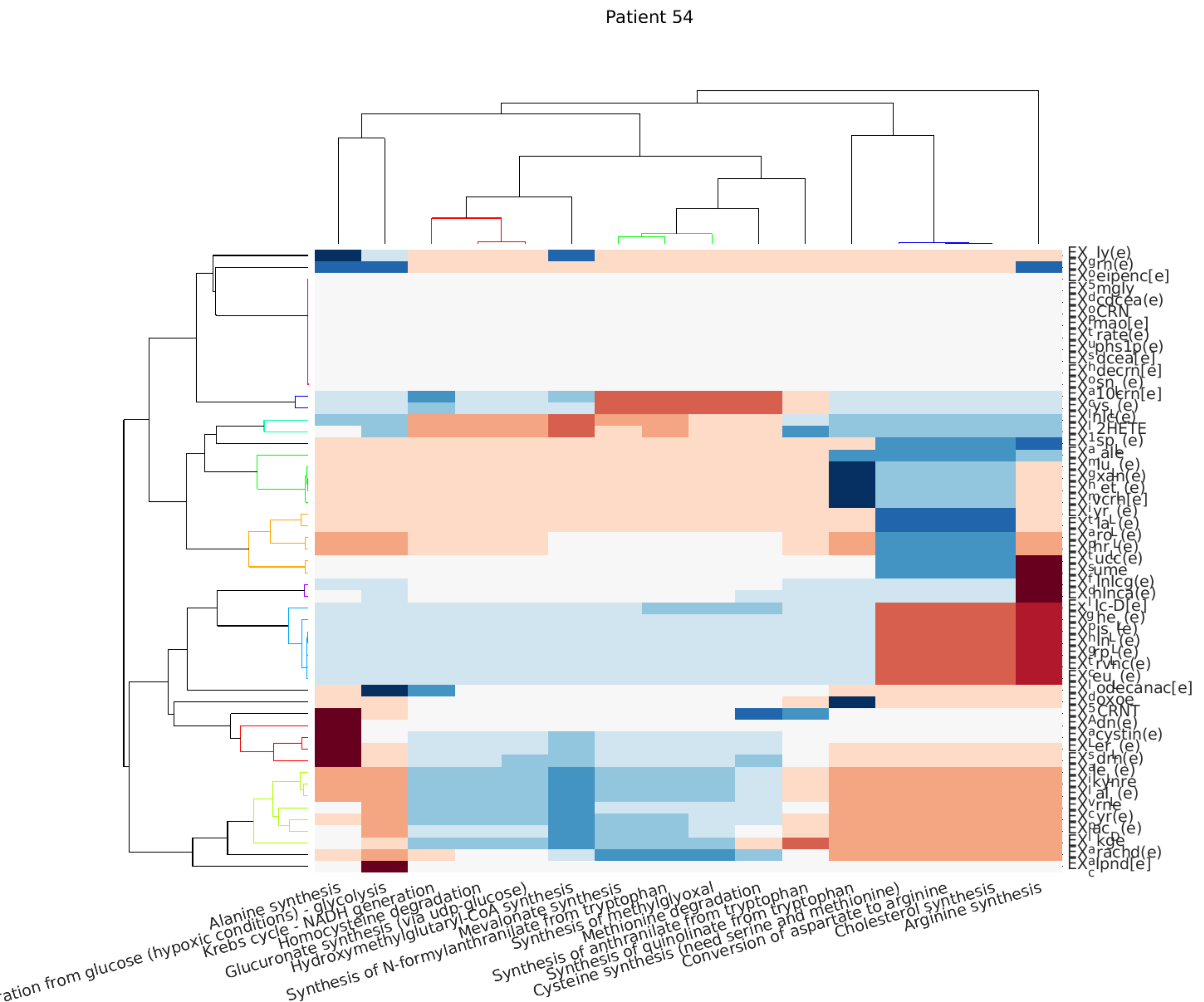
Patient 54
Alanine synthesis
Krebs cycle - NADH generation
Homocysteine degradation
Glucuronate synthesis (via udp-glucose)
Hydroxymethylglutaryl-CoA synthesis
Mevalonate synthesis
Synthesis of N-formylanthranilate from tryptophan
Synthesis of methylglyoxal
Methionine degradation
Synthesis of anthranilate from tryptophan
Synthesis of quinolinate from tryptophan
Cysteine synthesis (need serine and methionine)
Conversion of aspartate to arginine
Cholesterol synthesis
Arginine synthesis

Patient 55

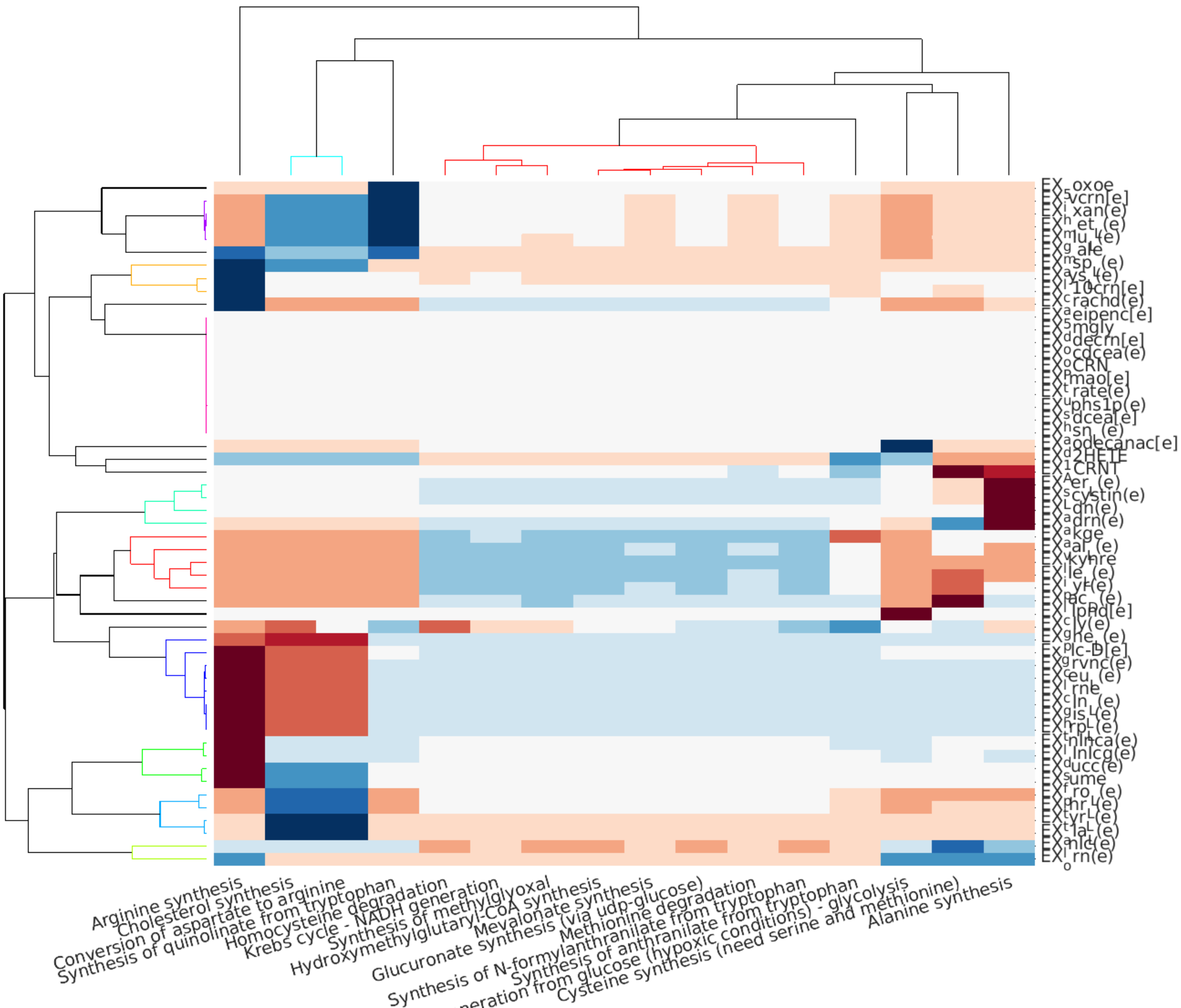
Arginine synthesis
Cholesterol synthesis
Conversion of aspartate to arginine
Synthesis of quinolinate from tryptophan
Homocysteine degradation
Krebs cycle - NADH generation
Synthesis of methylglyoxal
Hydroxymethylglutaryl-CoA synthesis
Mevalonate synthesis
Glucuronate synthesis (via udp-glucose)
Methionine degradation
Synthesis of N-formylanthranilate from tryptophan
Synthesis of anthranilate from tryptophan
glucose (hypoxic conditions) - glycolysis
Cysteine synthesis (need serine and methionine)
Alanine synthesis

Patient 56

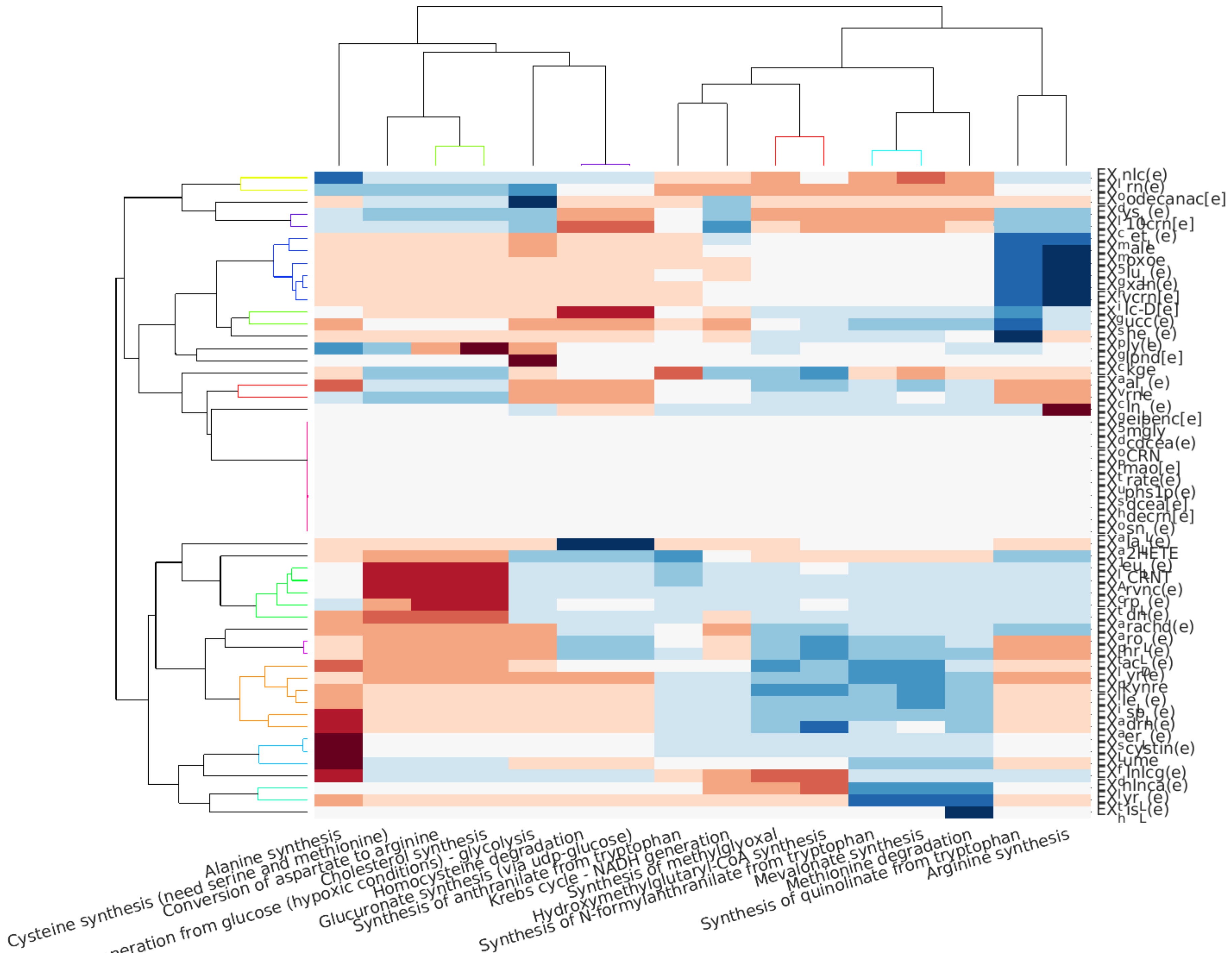
Alanine synthesis
Cysteine synthesis (need serine and methionine)
Conversion of aspartate to arginine
Cholesterol synthesis
Homocysteine degradation
Glucuronate synthesis (via udp-glucose)
Synthesis of anthranilate from tryptophan
Krebs cycle - NADH generation
Synthesis of methylglyoxal
Hydroxymethylglutaryl-CoA synthesis
Synthesis of N-formylanthranilate from tryptophan
Mevalonate synthesis
Methionine degradation
Synthesis of quinolinate from tryptophan
Arginine synthesis

Patient 57

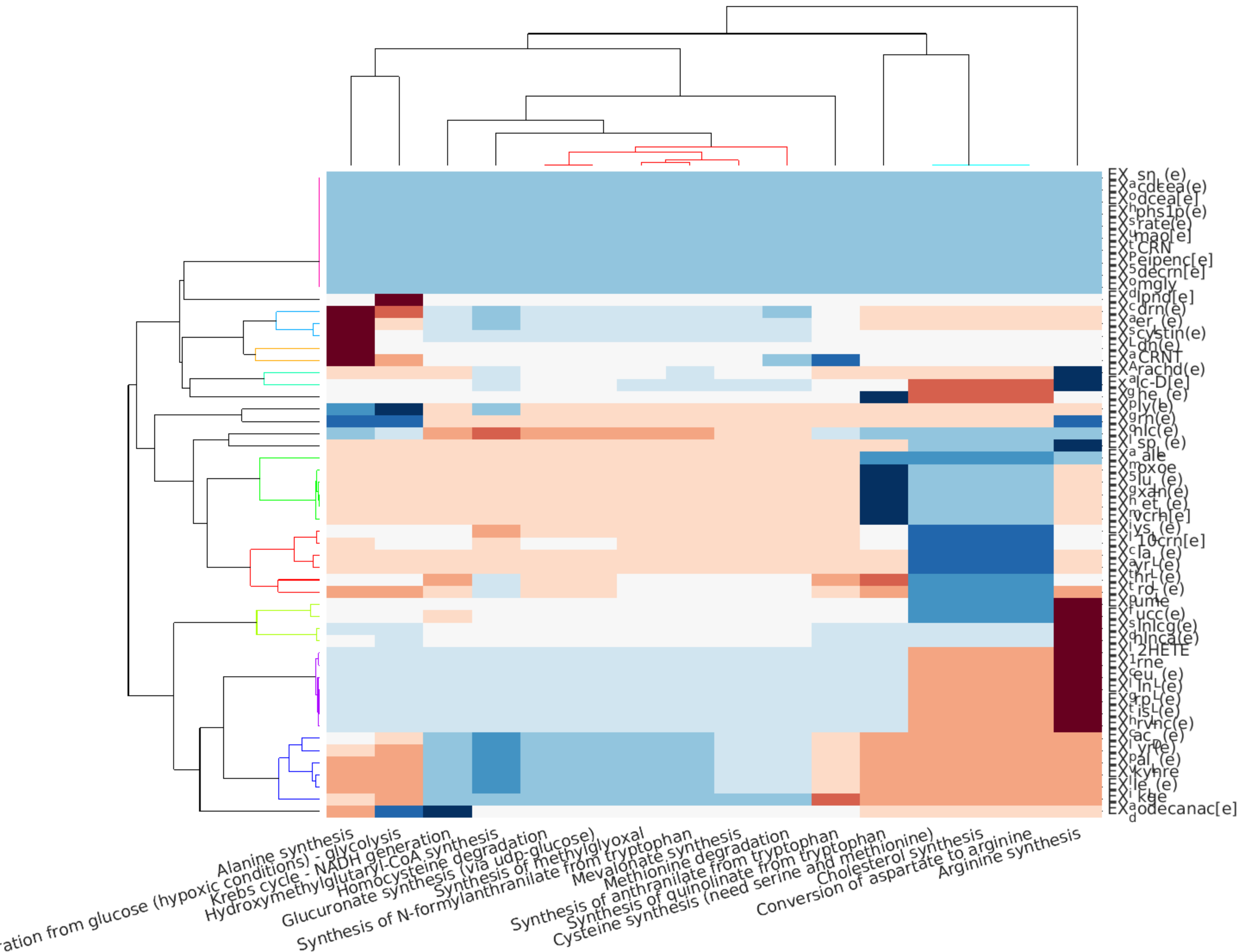
Alanine synthesis
Krebs cycle - NADH generation
Hydroxymethylglutaryl-CoA synthesis
Homocysteine degradation
Glucuronate synthesis (via udp-glucose)
Synthesis of methylglyoxal
Synthesis of N-formylanthranilate from tryptophan
Mevalonate synthesis
Methionine degradation
Synthesis of anthranilate from tryptophan
Synthesis of quinolinate from tryptophan
Cysteine synthesis (need serine and methionine)
Cholesterol synthesis
Conversion of aspartate to arginine
Arginine synthesis

Patient 58

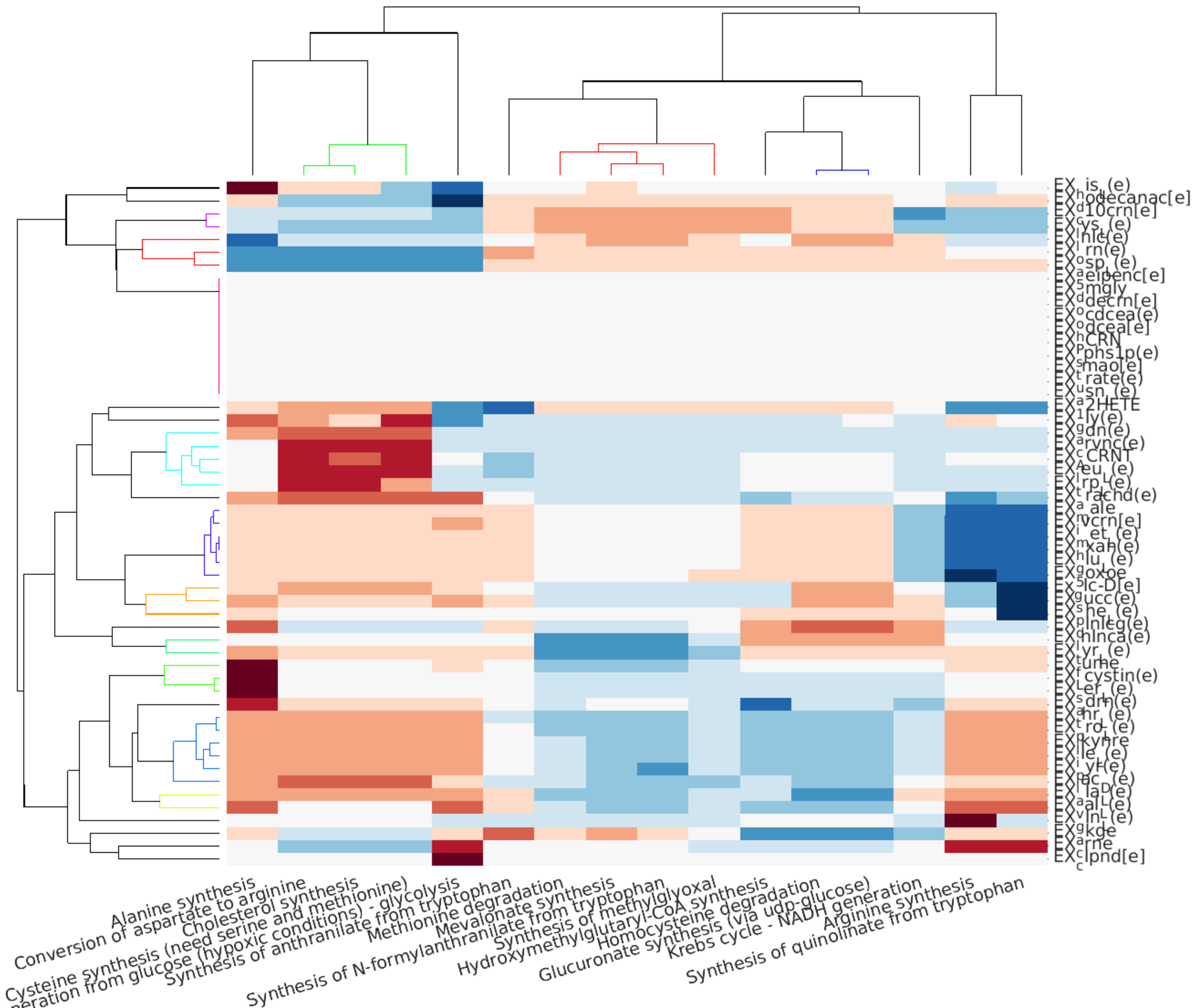

Alanine synthesis
Conversion of aspartate to arginine
Cholesterol synthesis
Cysteine synthesis (need serine and methionine)
Synthesis of anthranilate from tryptophan
Methionine degradation
Synthesis of N-formylanthranilate from tryptophan
Mevalonate synthesis
Synthesis of methylglyoxal
Hydroxymethylglutaryl-CoA synthesis
Homocysteine degradation
Glucuronate synthesis (via udp-glucose)
Krebs cycle - NADH generation
Arginine synthesis
Synthesis of quinolinate from tryptophan

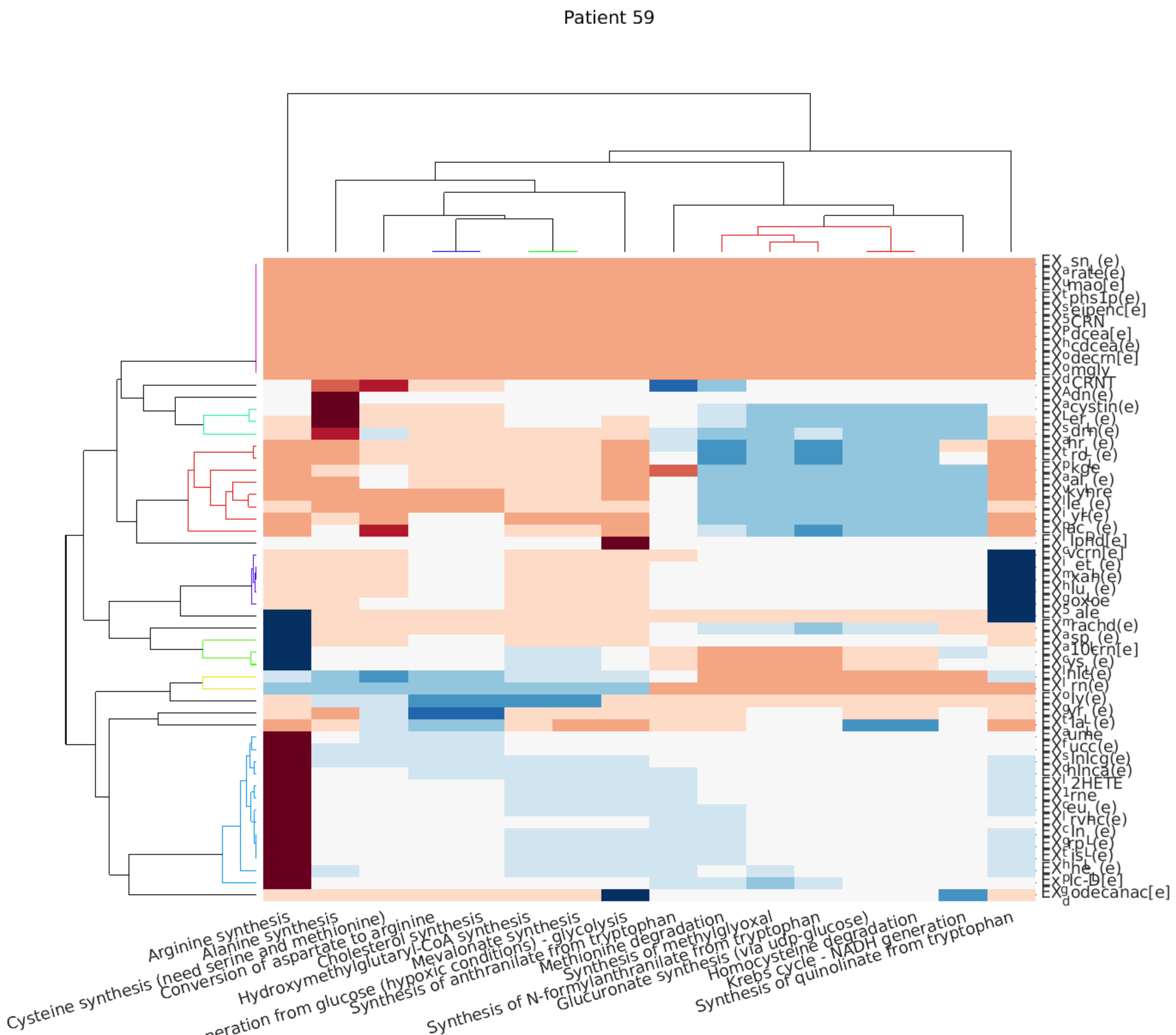
Patient 59
Arginine synthesis
Cysteine synthesis (need serine and methionine)
Alanine synthesis
Conversion of aspartate to arginine
Hydroxymethylglutaryl-CoA synthesis
Cholesterol synthesis
Mevalonate synthesis
Synthesis of anthranilate from tryptophan
Methionine degradation
Synthesis of N-formylanthranilate from tryptophan
Synthesis of methylglyoxal
Glucuronate synthesis (via udp-glucose)
Homocysteine degradation
Krebs cycle - NADH generation
Synthesis of quinolinate from tryptophan

Patient 60

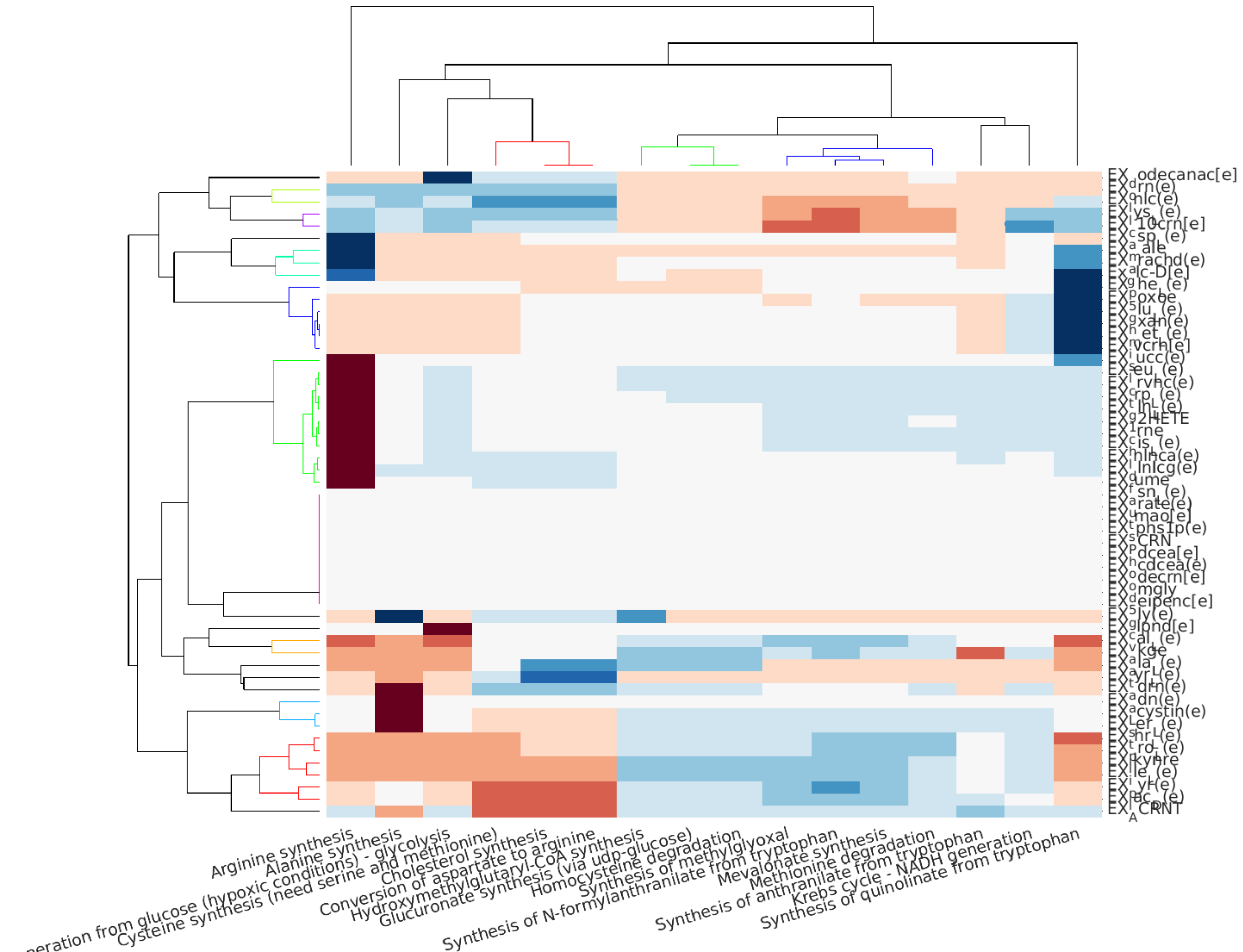

Arginine synthesis
Alanine synthesis
from glucose (hypoxic conditions) - glycolysis
Cysteine synthesis (need serine and methionine)
Cholesterol synthesis
Conversion of aspartate to arginine
Hydroxymethylglutaryl-CoA synthesis
Glucuronate synthesis (via udp-glucose)
Homocysteine degradation
Synthesis of N-formylanthranilate from tryptophan
Synthesis of methylglyoxal
Mevalonate synthesis
Methionine degradation
Synthesis of anthranilate from tryptophan
Krebs cycle - NADH generation
Synthesis of quinolinate from tryptophan

Patient 61

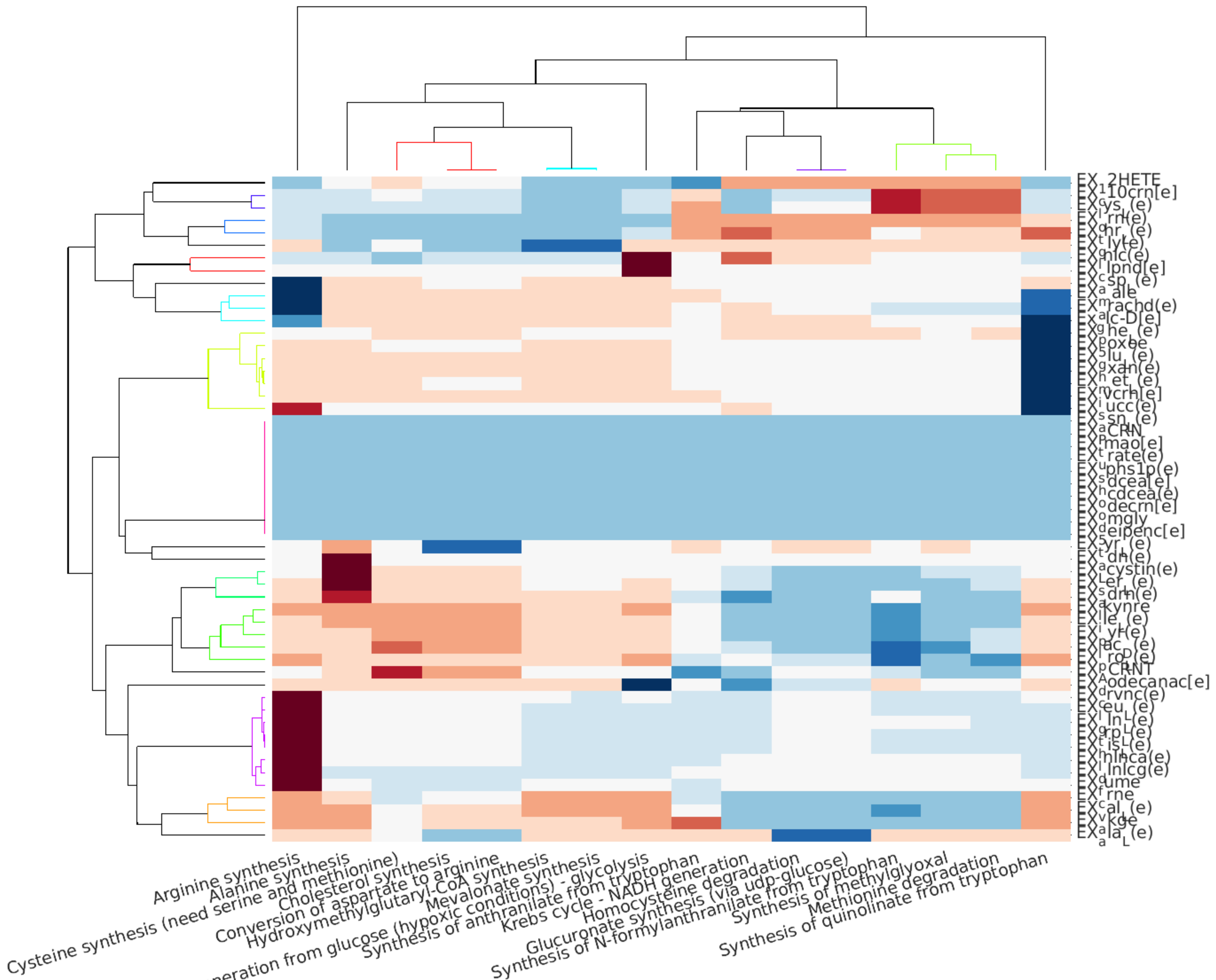

Arginine synthesis
Alanine synthesis
Cysteine synthesis (need serine and methionine)
Cholesterol synthesis
Conversion of aspartate to arginine
Hydroxymethylglutaryl-CoA synthesis
Mevalonate synthesis
Synthesis of anthranilate from tryptophan
Krebs cycle - NADH generation
Homocysteine degradation
Glucuronate synthesis (via udp-glucose)
Synthesis of N-formylanthranilate from tryptophan
Synthesis of methylglyoxal
Methionine degradation
Synthesis of quinolinate from tryptophan

Patient 62

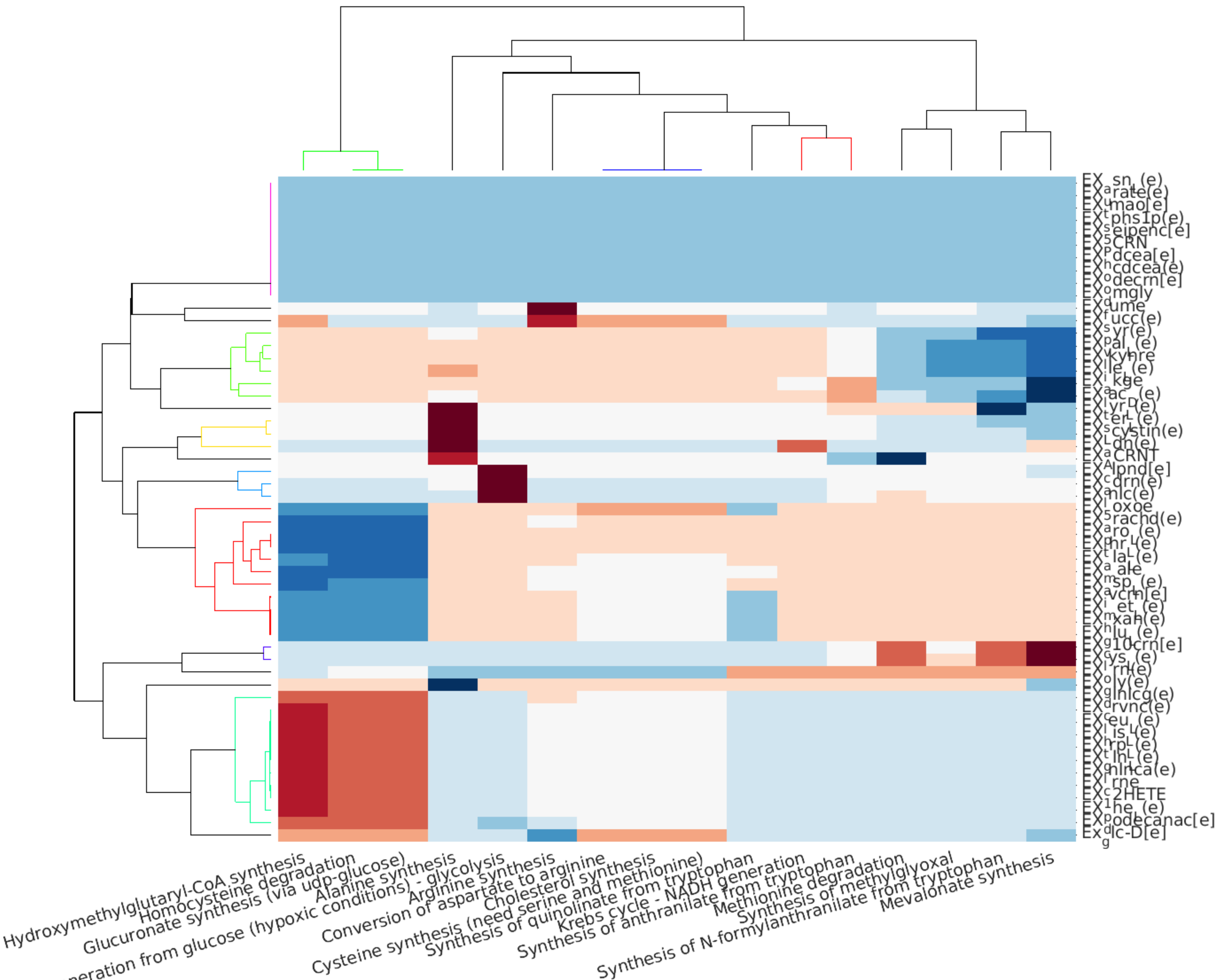
Hydroxymethylglutaryl-CoA synthesis
Homocysteine degradation
Glucuronate synthesis (via udp-glucose)
Alanine synthesis
Arginine synthesis
Conversion of aspartate to arginine
Cysteine synthesis (need serine and methionine)
Cholesterol synthesis
Synthesis of quinolinate from tryptophan
Krebs cycle - NADH generation
Synthesis of anthranilate from tryptophan
Methionine degradation
Synthesis of methylglyoxal
Synthesis of N-formylanthranilate from tryptophan
Mevalonate synthesis

Patient 63

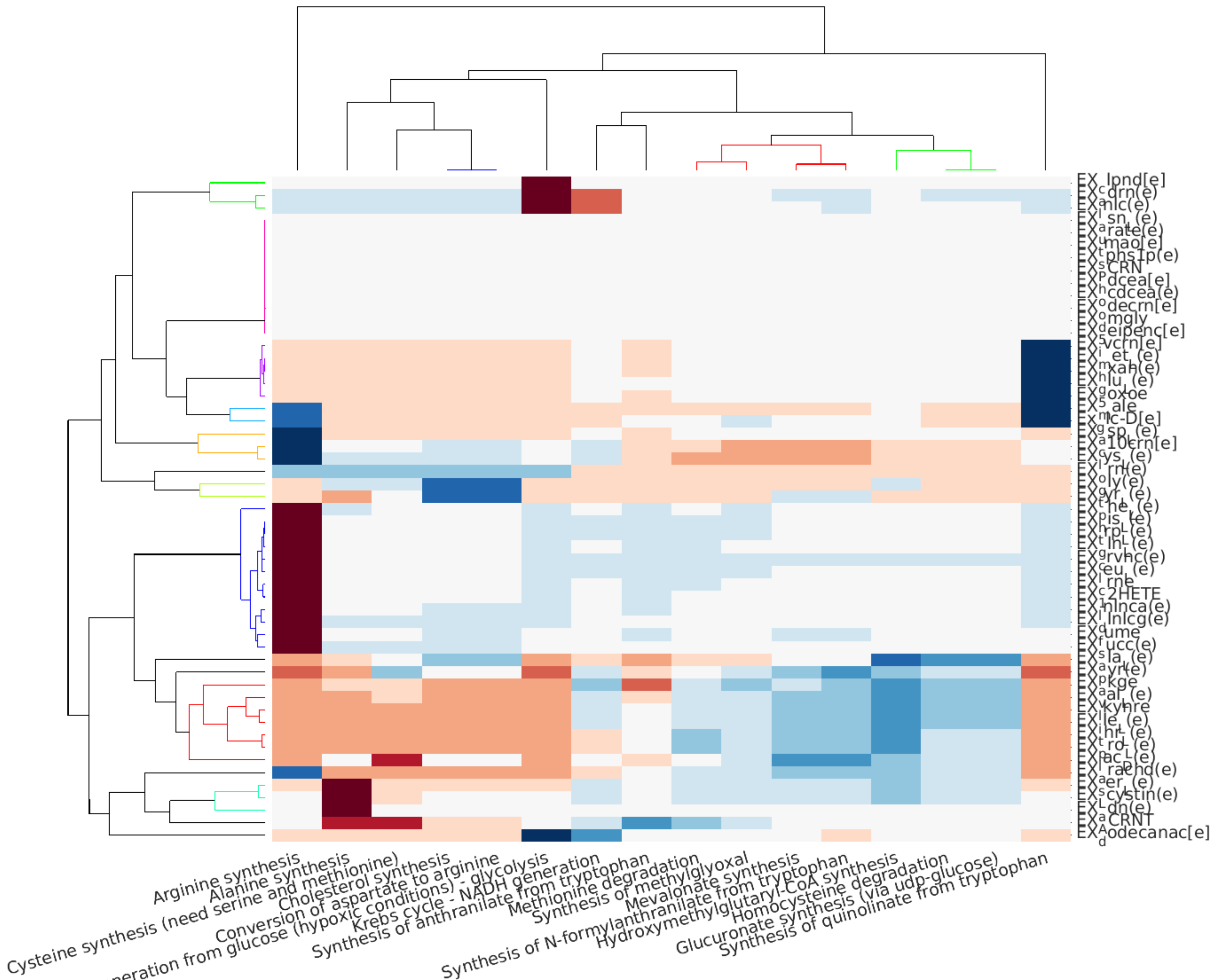

Arginine synthesis
Alanine synthesis
Cysteine synthesis (need serine and methionine)
Cholesterol synthesis
Conversion of aspartate to arginine
Krebs cycle - NADH generation
Synthesis of anthranilate from tryptophan
Methionine degradation
Synthesis of methylglyoxal
Mevalonate synthesis
Synthesis of N-formylanthranilate from tryptophan
Hydroxymethylglutaryl-CoA synthesis
Homocysteine degradation
Glucuronate synthesis (via udp-glucose)
Synthesis of quinolinate from tryptophan

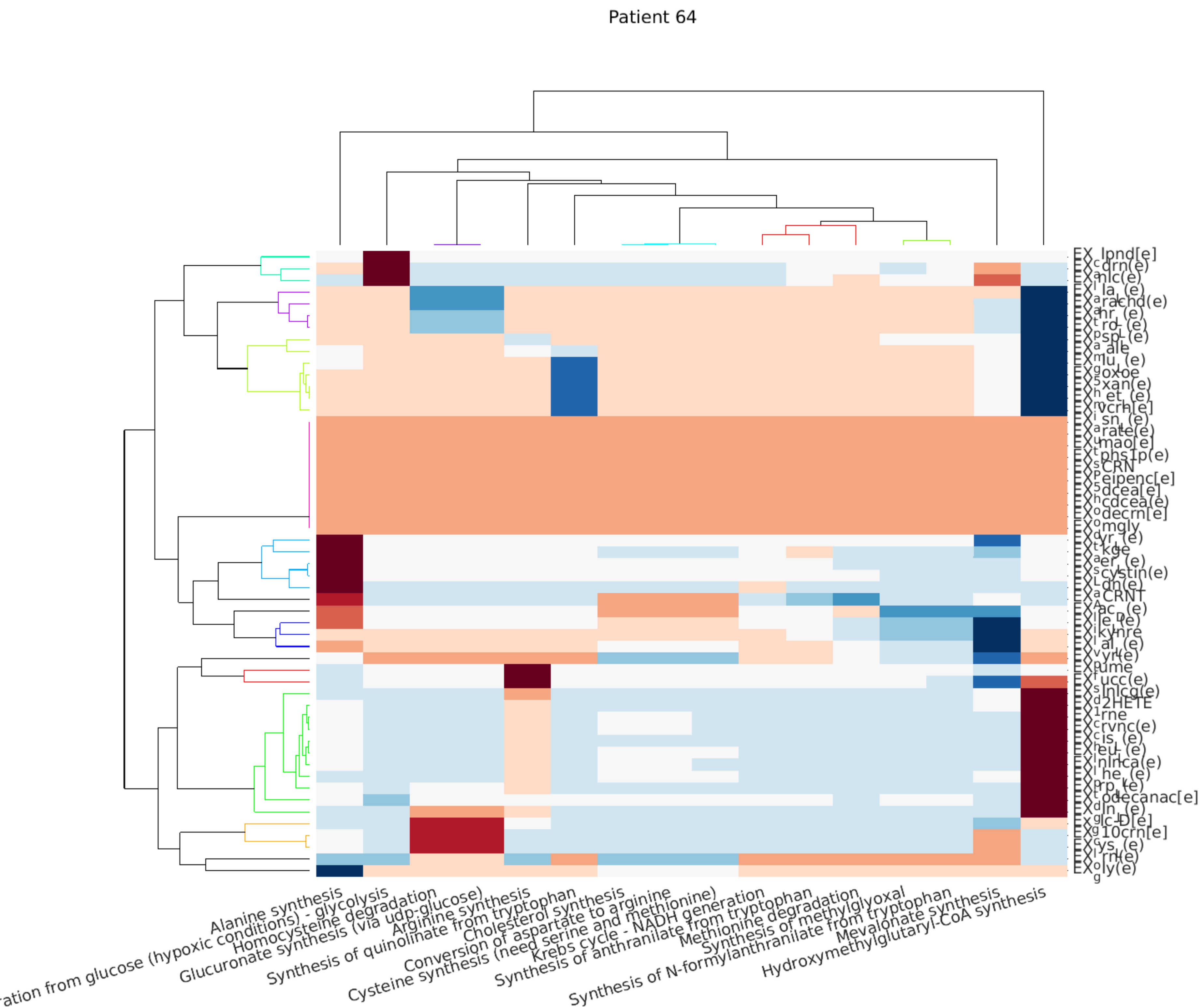
Patient 64
Alanine synthesis
ration from glucose (hypoxic conditions) - glycolysis
Homocysteine degradation
Glucuronate synthesis (via udp-glucose)
Arginine synthesis
Synthesis of quinolinate from tryptophan
Cholesterol synthesis
Conversion of aspartate to arginine
Cysteine synthesis (need serine and methionine)
Krebs cycle - NADH generation
Synthesis of anthranilate from tryptophan
Methionine degradation
Synthesis of N-formylanthranilate from tryptophan
Synthesis of methylglyoxal
Mevalonate synthesis
Hydroxymethylglutaryl-CoA synthesis

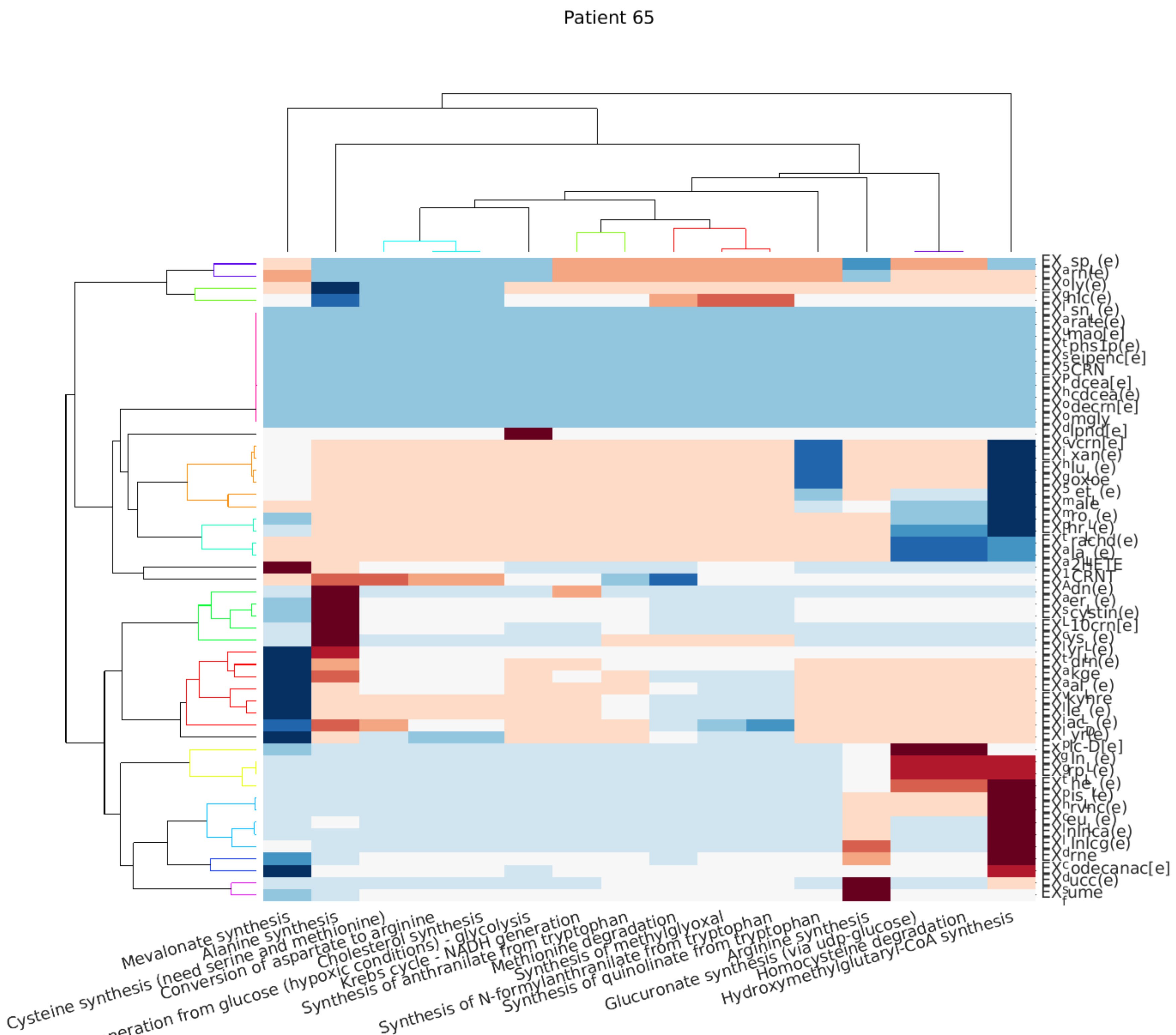

Patient 65
Mevalonate synthesis
Alanine synthesis
Cysteine synthesis (need serine and methionine)
Conversion of aspartate to arginine
neration from glucose (hypoxic conditions) - glycolysis
Cholesterol synthesis
Krebs cycle - NADH generation
Synthesis of anthranilate from tryptophan
Methionine degradation
Synthesis of N-formylanthranilate from tryptophan
Synthesis of methylglyoxal
Synthesis of quinolinate from tryptophan
Arginine synthesis
Glucuronate synthesis (via udp-glucose)
Homocysteine degradation
Hydroxymethylglutaryl-CoA synthesis

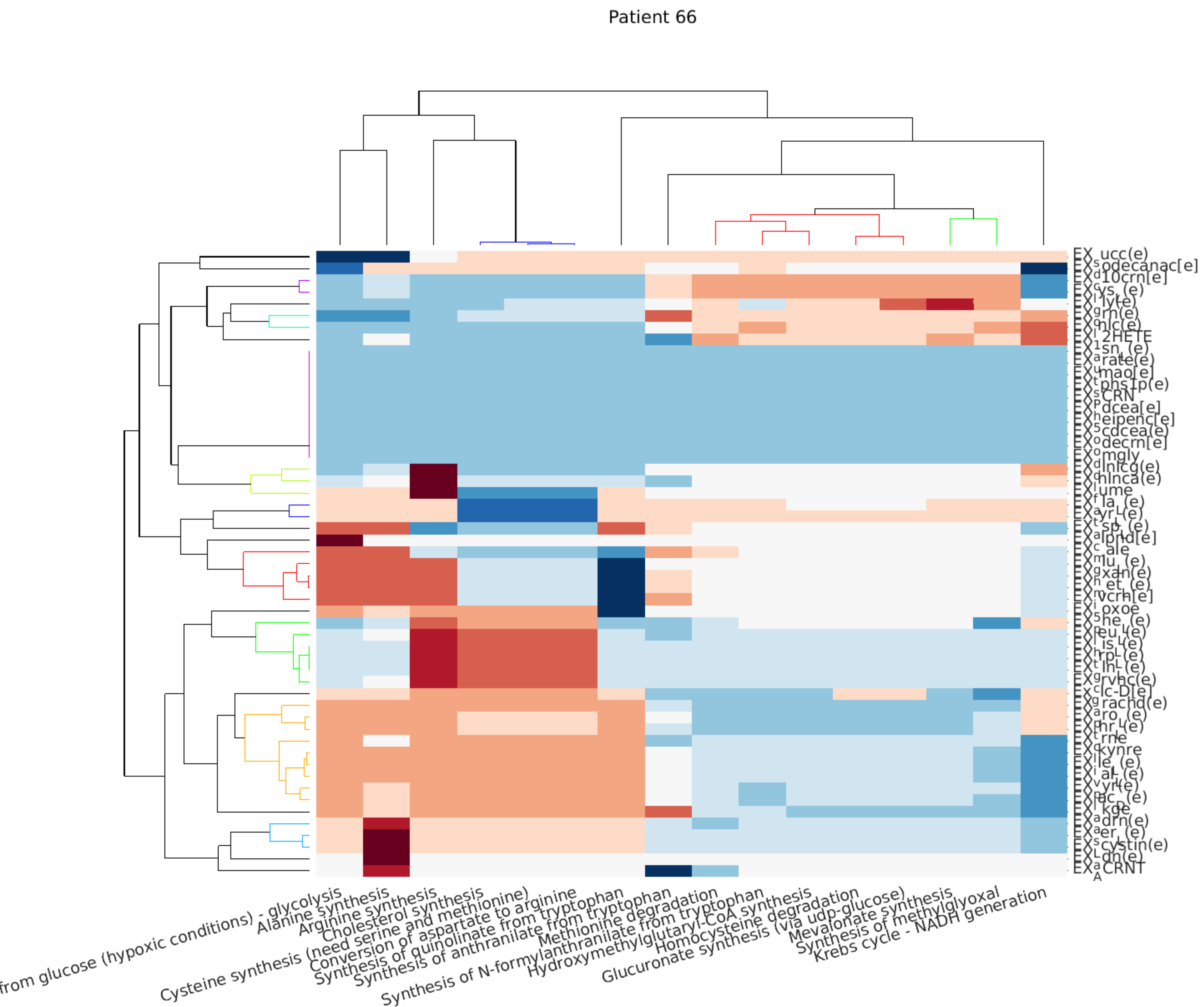
Patient 66
from glucose (hypoxic conditions) - glycolysis
Alanine synthesis
Arginine synthesis
Cholesterol synthesis
Cysteine synthesis (need serine and methionine)
Conversion of aspartate to arginine
Synthesis of quinolinate from tryptophan
Synthesis of anthranilate from tryptophan
Methionine degradation
Synthesis of N-formylanthranilate from tryptophan
Hydroxymethylglutaryl-CoA synthesis
Homocysteine degradation
Glucuronate synthesis (via udp-glucose)
Mevalonate synthesis
Synthesis of methylglyoxal
Krebs cycle - NADH generation

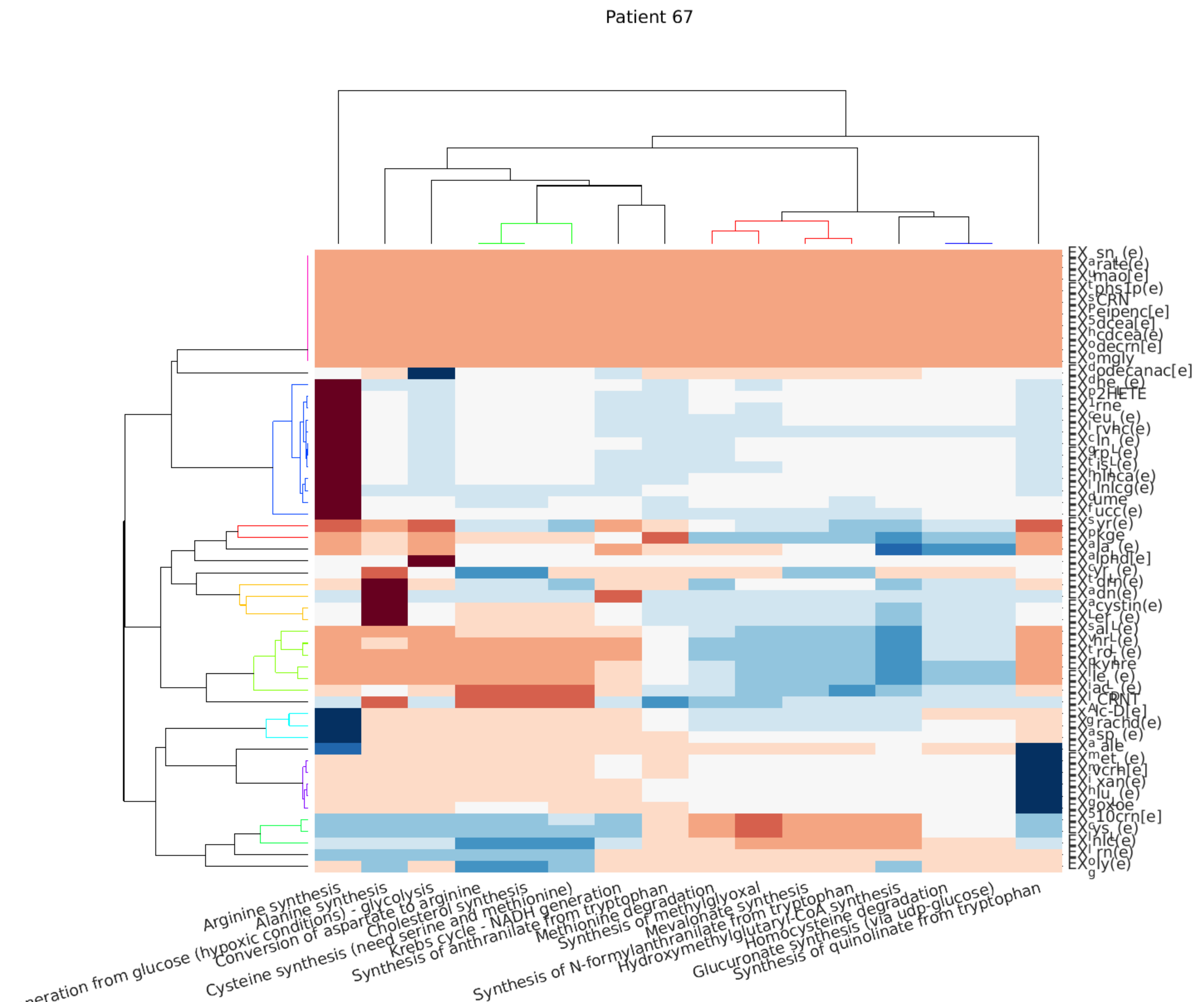

Patient 67
Arginine synthesis
Alanine synthesis
Glycolysis
Conversion of aspartate to arginine
Cholesterol synthesis
Cysteine synthesis (need serine and methionine)
Krebs cycle - NADH generation
Synthesis of anthranilate from tryptophan
Methionine degradation
Synthesis of N-formylanthranilate from tryptophan
Synthesis of methylglyoxal
Mevalonate synthesis
Hydroxymethylglutaryl-CoA synthesis
Homocysteine degradation
Glucuronate synthesis (via udp-glucose)
Synthesis of quinolinate from tryptophan

Patient 68

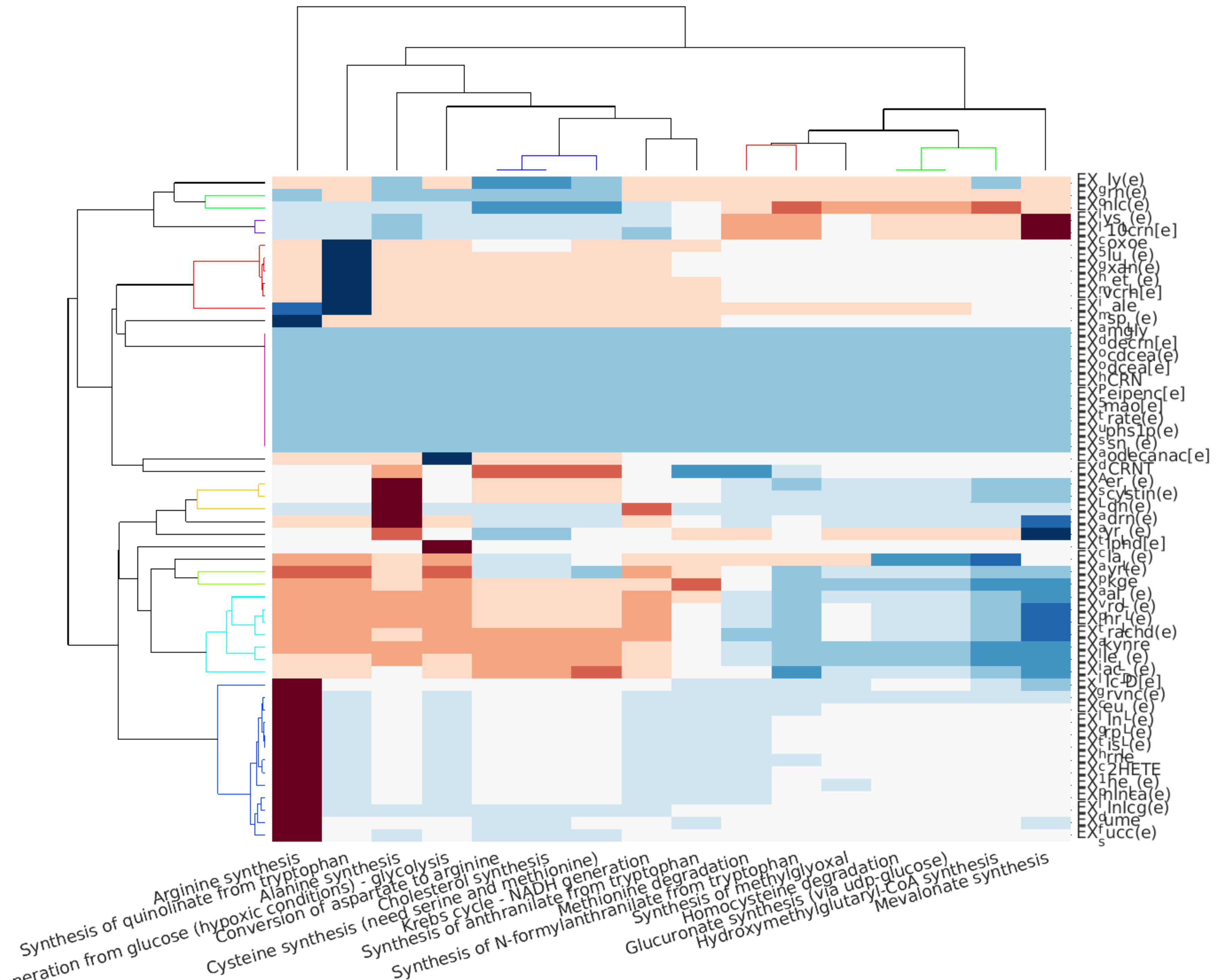
Arginine synthesis
Synthesis of quinolinate from tryptophan
Alanine synthesis
Conversion of aspartate to arginine
Cholesterol synthesis
Cysteine synthesis (need serine and methionine)
Krebs cycle - NADH generation
Synthesis of anthranilate from tryptophan
Methionine degradation
Synthesis of N-formylanthranilate from tryptophan
Synthesis of methylglyoxal
Homocysteine degradation
Glucuronate synthesis (via udp-glucose)
Hydroxymethylglutaryl-CoA synthesis
Mevalonate synthesis

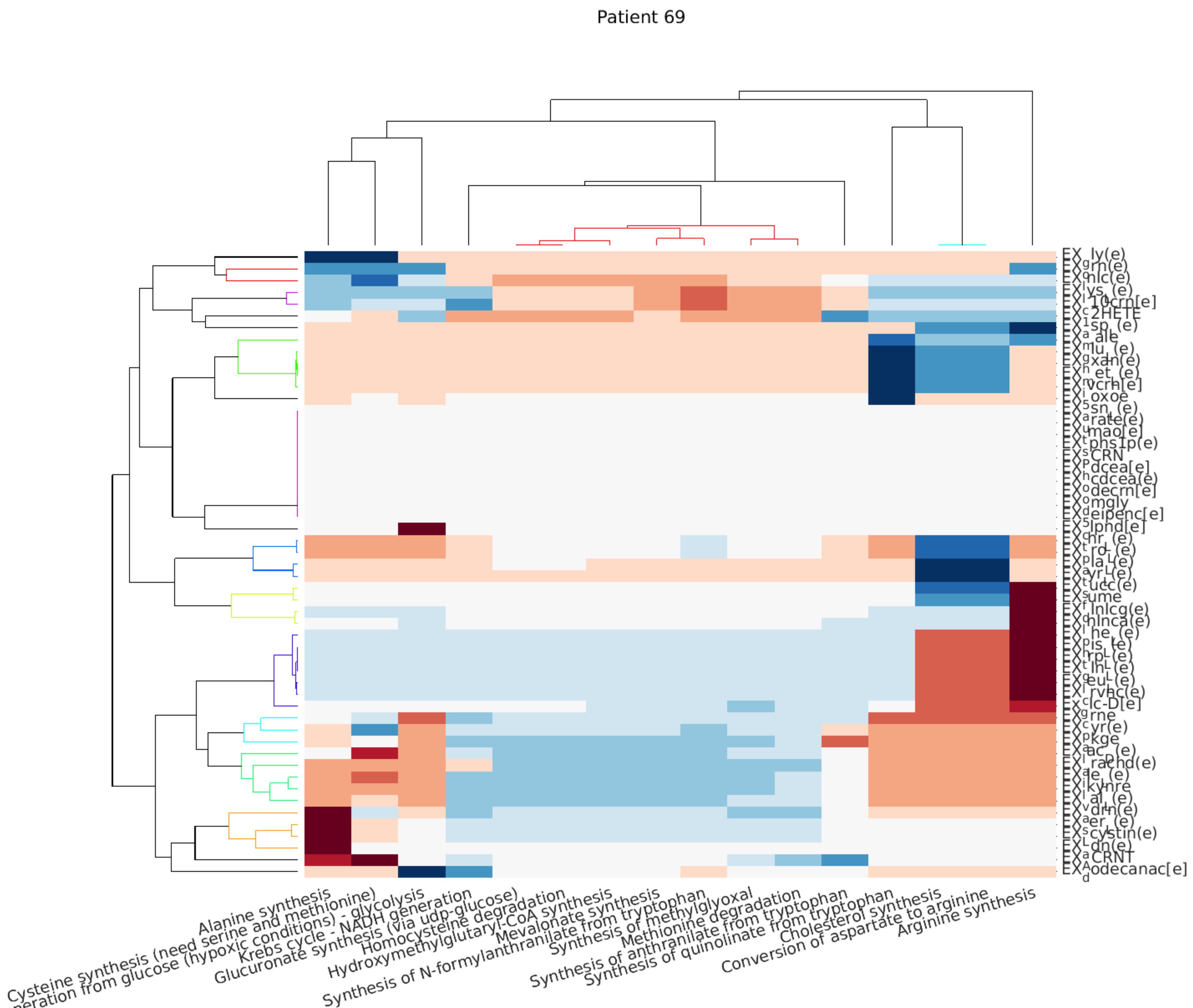
Patient 69
Alanine synthesis
Glucuronate synthesis (via udp-glucose)
Homocysteine degradation
Hydroxymethylglutaryl-CoA synthesis
Mevalonate synthesis
Synthesis of N-formylanthranilate from tryptophan
Synthesis of methylglyoxal
Methionine degradation
Synthesis of anthranilate from tryptophan
Synthesis of quinolinate from tryptophan
Cholesterol synthesis
Conversion of aspartate to arginine
Arginine synthesis
Krebs cycle - NADH generation

Patient 70

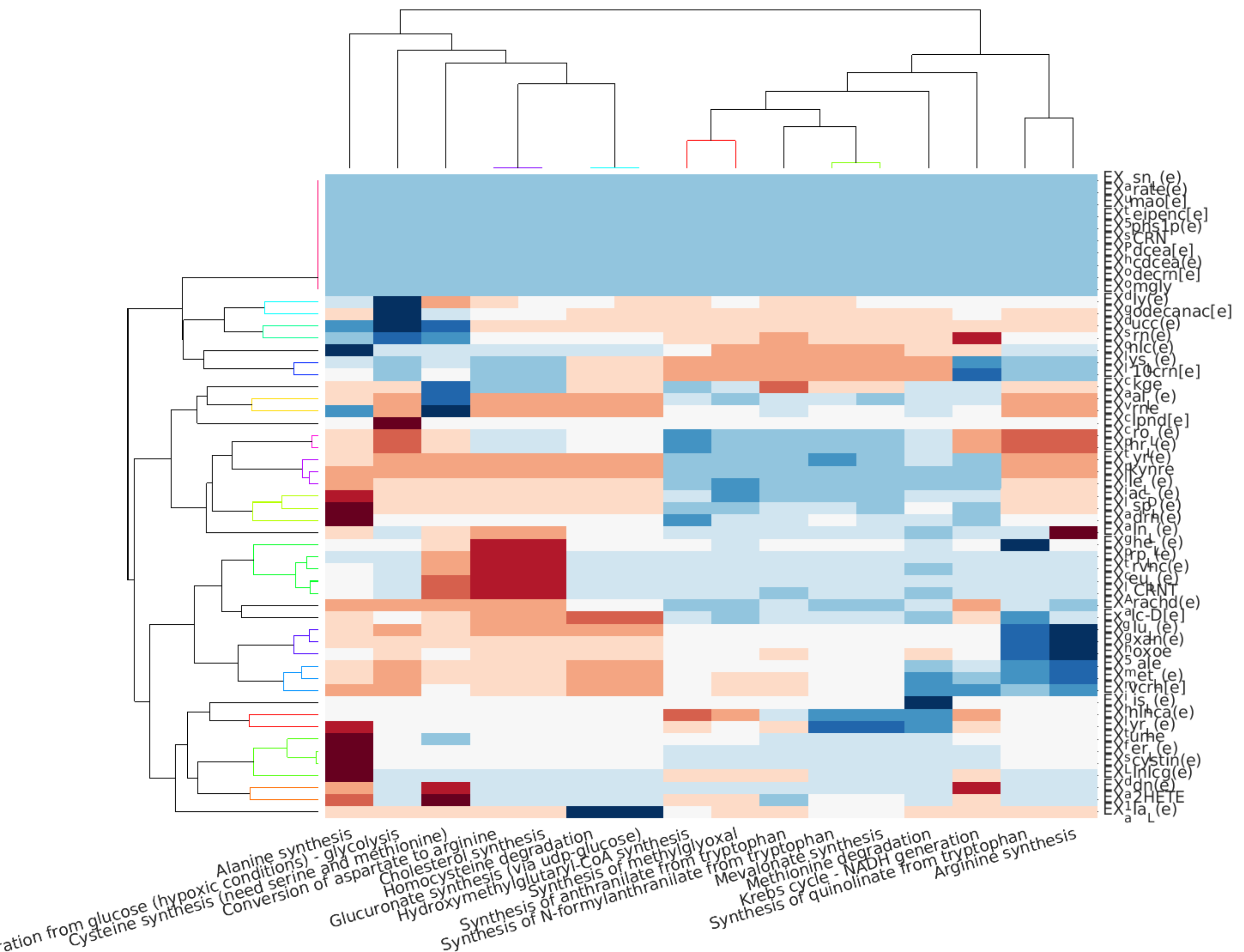
EX_sn_(e)
EX_arate(e)
EX_umao[e]
EX_teipenc[e]
EX_5phs1p(e)
EX_sCRN
EX_Pdcea[e]
EX_hcdcea(e)
EX_odecrn[e]
EX_omgly
EX_dly(e)
EX_godecanac[e]
EX_ducc(e)
EX_srn(e)
EX_qnlc(e)
EX_lys_(e)
EX_l 10crn[e]
EX_ckge
EX_aal_(e)
EX_vrne
EX_clpnd[e]
EX_cro_(e)
EX_phr_(e)
EX_tyr(e)
EX_pkynre
EX_lle_(e)
EX_iac_(e)
EX_lsp_(e)
EX_adrn(e)
EX_aln_(e)
EX_ghe_(e)
EX_prp_(e)
EX_trvnc(e)
EX_ceu_(e)
EX_l CRNT
EX_Arachd(e)
EX_alc-D[e]
EX_glu_(e)
EX_gxan(e)
EX_hoxoe
EX_5 ale
EX_met_(e)
EX_mvcrn[e]
EX_iis_(e)
EX_hmnca(e)
EX_lyr_(e)
EX_tume
EX_fer_(e)
EX_scystin(e)
EX_Lnlcg(e)
EX_ddn(e)
EX_a2HETE
EX_1la_(e)
Alanine synthesis
ration from glucose (hypoxic conditions) - glycolysis
Cysteine synthesis (need serine and methionine)
Conversion of aspartate to arginine
Cholesterol synthesis
Homocysteine degradation
Glucuronate synthesis (via udp-glucose)
Hydroxymethylglutaryl-CoA synthesis
Synthesis of methylglyoxal
Synthesis of anthranilate from tryptophan
Synthesis of N-formylanthranilate from tryptophan
Mevalonate synthesis
Methionine degradation
Krebs cycle - NADH generation
Synthesis of quinolinate from tryptophan
Arginine synthesis

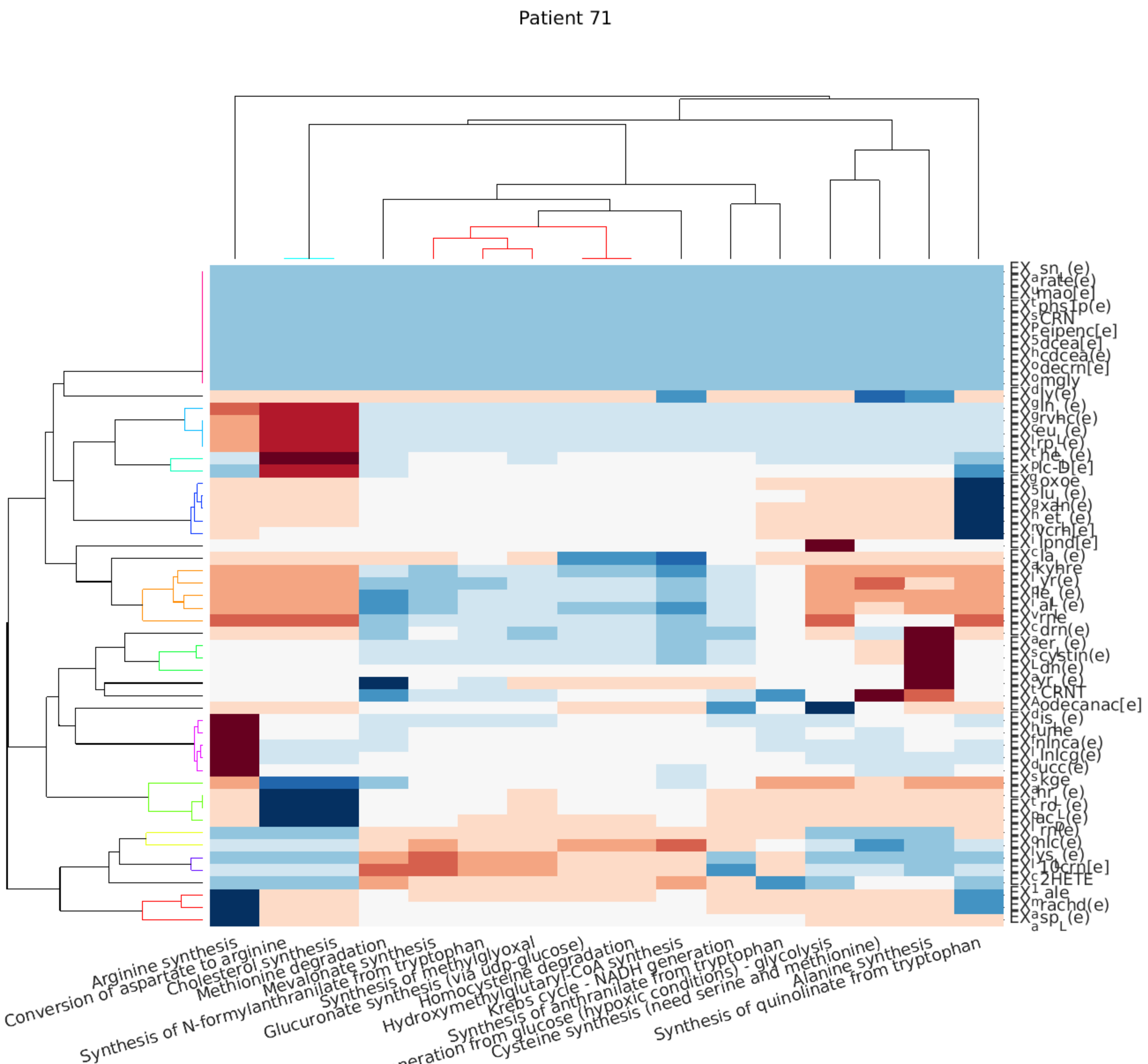
Patient 71
Arginine synthesis
Conversion of aspartate to arginine
Cholesterol synthesis
Methionine degradation
Mevalonate synthesis
Synthesis of N-formylanthranilate from tryptophan
Synthesis of methylglyoxal
Glucuronate synthesis (via udp-glucose)
Homocysteine degradation
Hydroxymethylglutaryl-CoA synthesis
Krebs cycle - NADH generation
Synthesis of anthranilate from tryptophan
eration from glucose (hypoxic conditions) - glycolysis
Cysteine synthesis (need serine and methionine)
Alanine synthesis
Synthesis of quinolinate from tryptophan

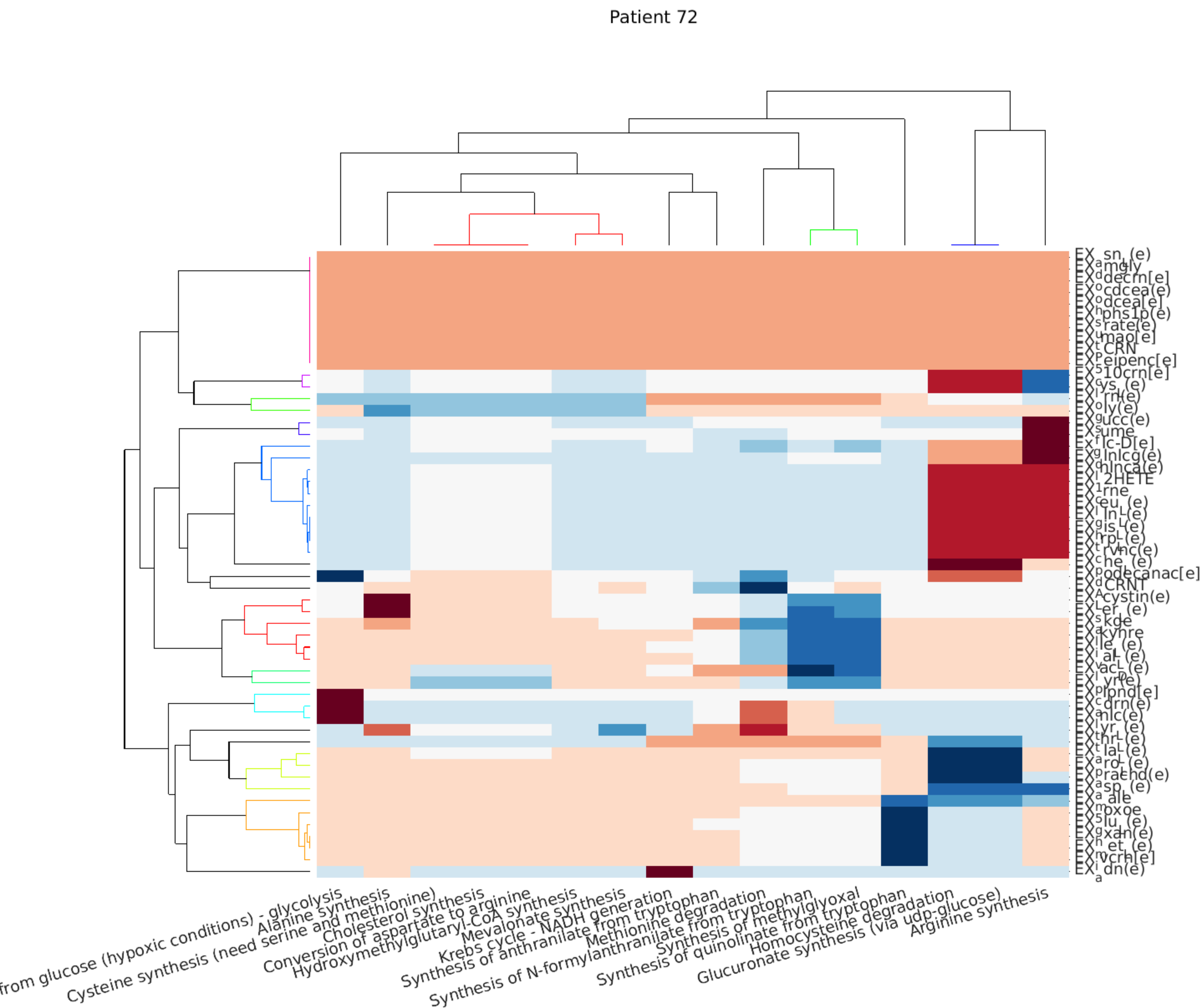
Patient 72
Alanine synthesis
Cholesterol synthesis
Mevalonate synthesis
Methionine degradation
Arginine synthesis

Patient 73

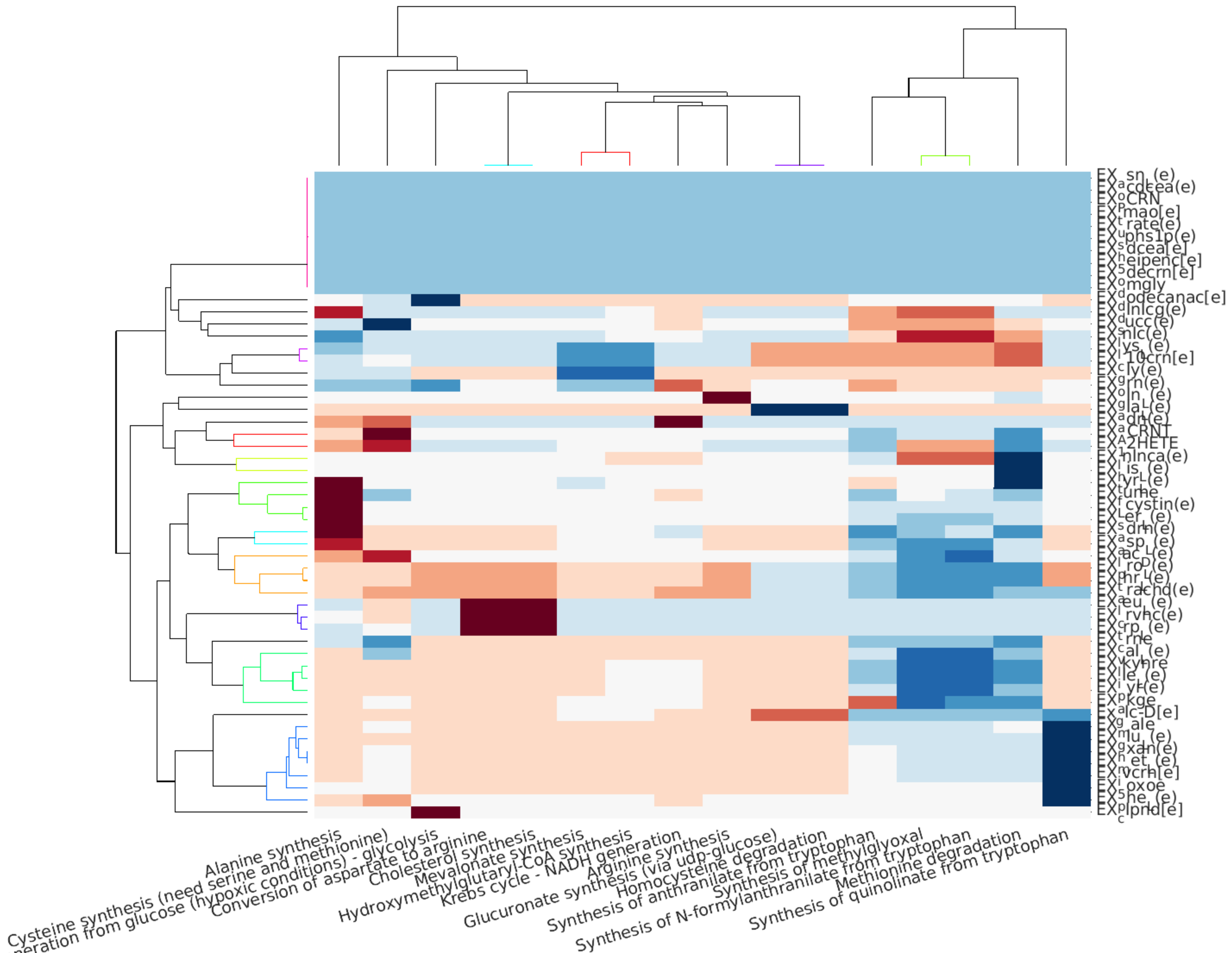
Alanine synthesis
Cysteine synthesis (need serine and methionine)
Generation from glucose (hypoxic conditions) - glycolysis
Conversion of aspartate to arginine
Cholesterol synthesis
Mevalonate synthesis
Hydroxymethylglutaryl-CoA synthesis
Krebs cycle - NADH generation
Arginine synthesis
Glucuronate synthesis (via udp-glucose)
Homocysteine degradation
Synthesis of anthranilate from tryptophan
Synthesis of methylglyoxal
Synthesis of N-formylanthranilate from tryptophan
Methionine degradation
Synthesis of quinolinate from tryptophan

Patient 74

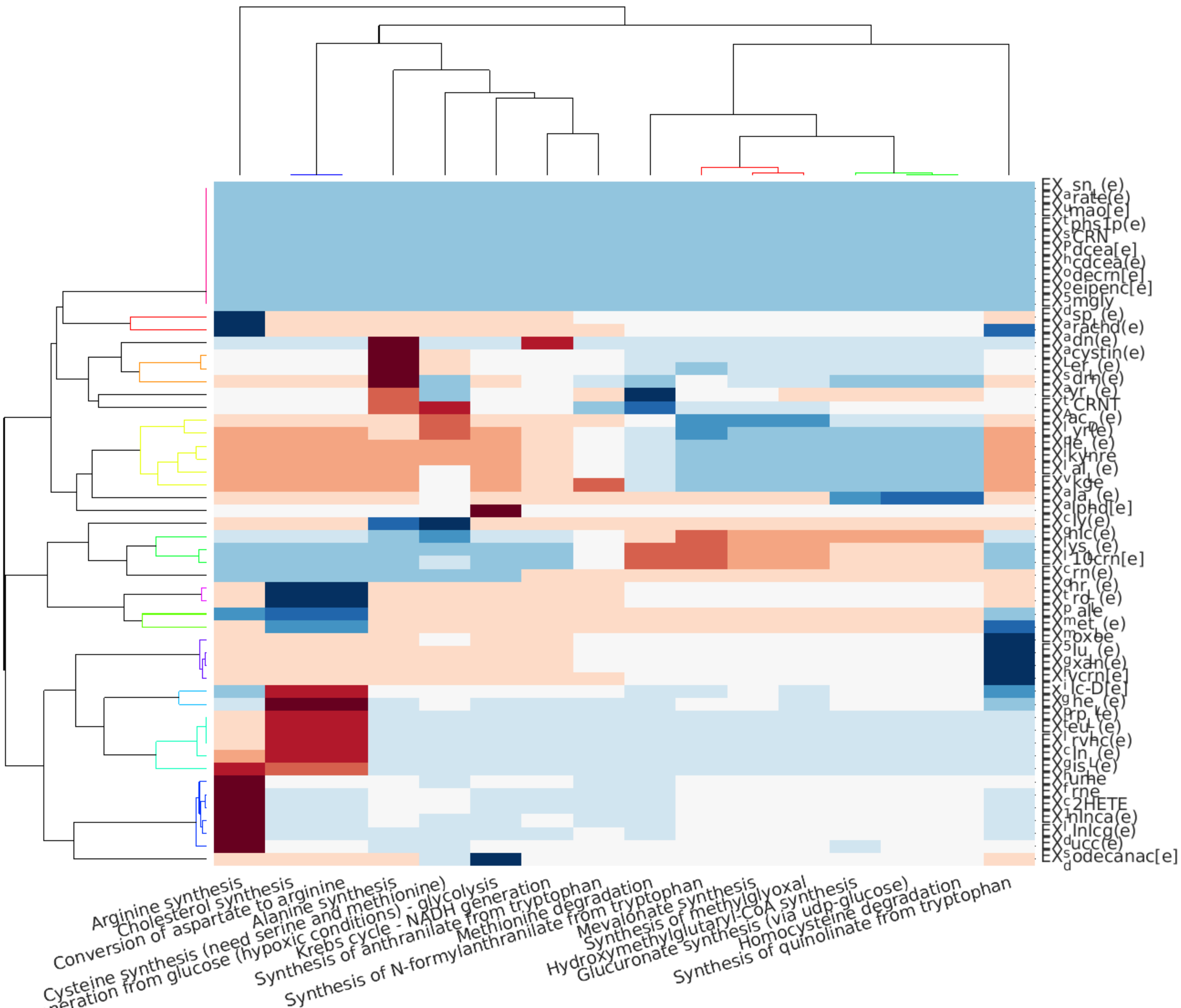
Arginine synthesis
Cholesterol synthesis
Conversion of aspartate to arginine
Alanine synthesis
Cysteine synthesis (need serine and methionine)
Krebs cycle - NADH generation
Synthesis of anthranilate from tryptophan
Methionine degradation
Synthesis of N-formylanthranilate from tryptophan
Mevalonate synthesis
Synthesis of methylglyoxal
Hydroxymethylglutaryl-CoA synthesis
Glucuronate synthesis (via udp-glucose)
Homocysteine degradation
Synthesis of quinolinate from tryptophan

Patient 75

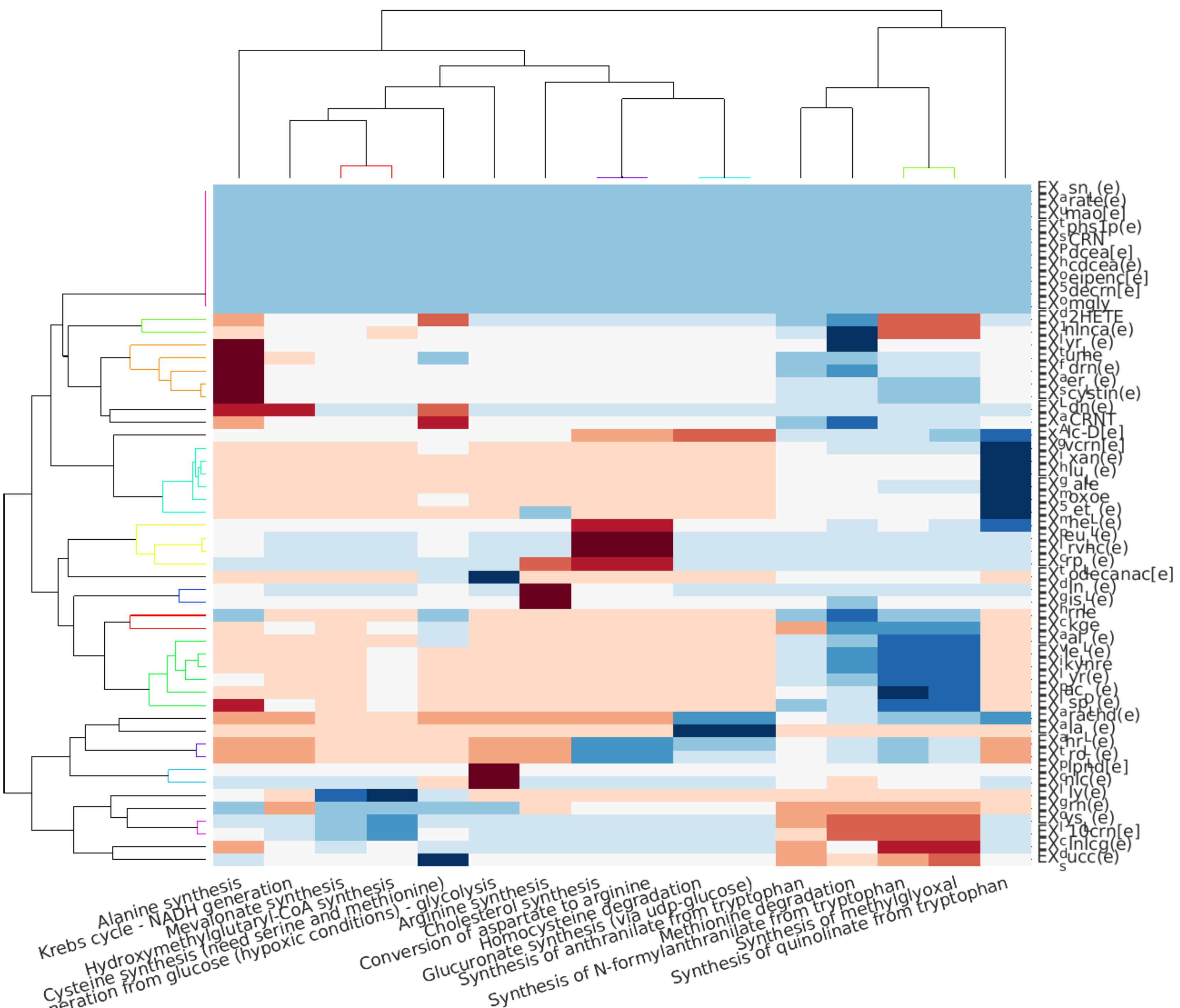
Alanine synthesis
Krebs cycle - NADH generation
Mevalonate synthesis
Hydroxymethylglutaryl-CoA synthesis
Cysteine synthesis (need serine and methionine)
Conversion of glucose (hypoxic conditions) - glycolysis
Arginine synthesis
Cholesterol synthesis
Synthesis of aspartate to arginine
Homocysteine degradation
Glucuronate synthesis (via udp-glucose)
Synthesis of anthranilate from tryptophan
Methionine degradation
Synthesis of N-formylanthranilate from tryptophan
Synthesis of methylglyoxal
Synthesis of quinolinate from tryptophan
EX_2HETE
EX_CRNT
EX_odecanac[e]
EX_10dcrn[e]

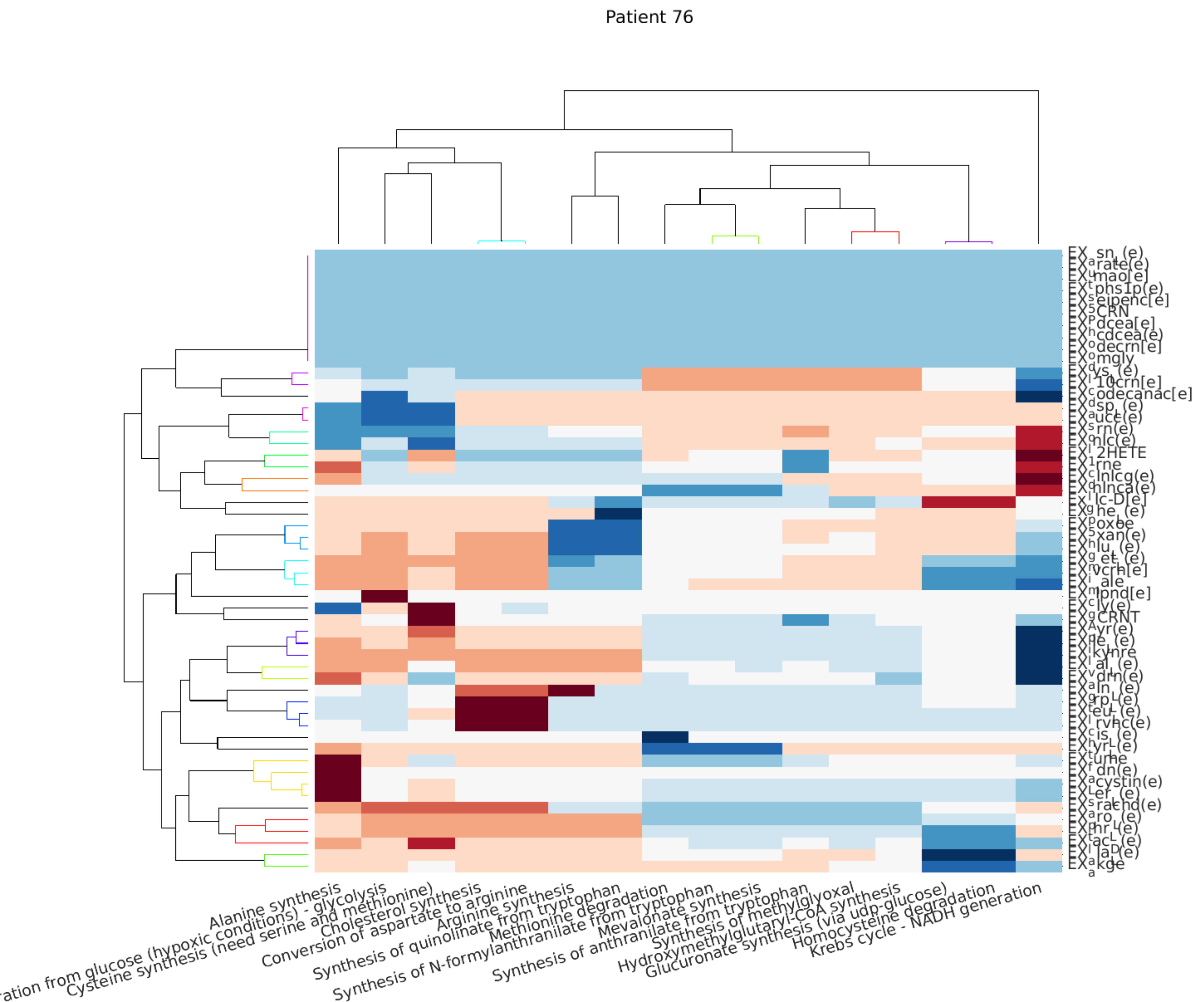
Patient 76
Alanine synthesis
Cysteine synthesis (need serine and methionine)
Cholesterol synthesis
Conversion of aspartate to arginine
Arginine synthesis
Synthesis of quinolinate from tryptophan
Methionine degradation
Synthesis of N-formylanthranilate from tryptophan
Mevalonate synthesis
Synthesis of anthranilate from tryptophan
Synthesis of methylglyoxal
Hydroxymethylglutaryl-CoA synthesis
Glucuronate synthesis (via udp-glucose)
Homocysteine degradation
Krebs cycle - NADH generation
EX_2HETE
EX_CRN

Patient 77

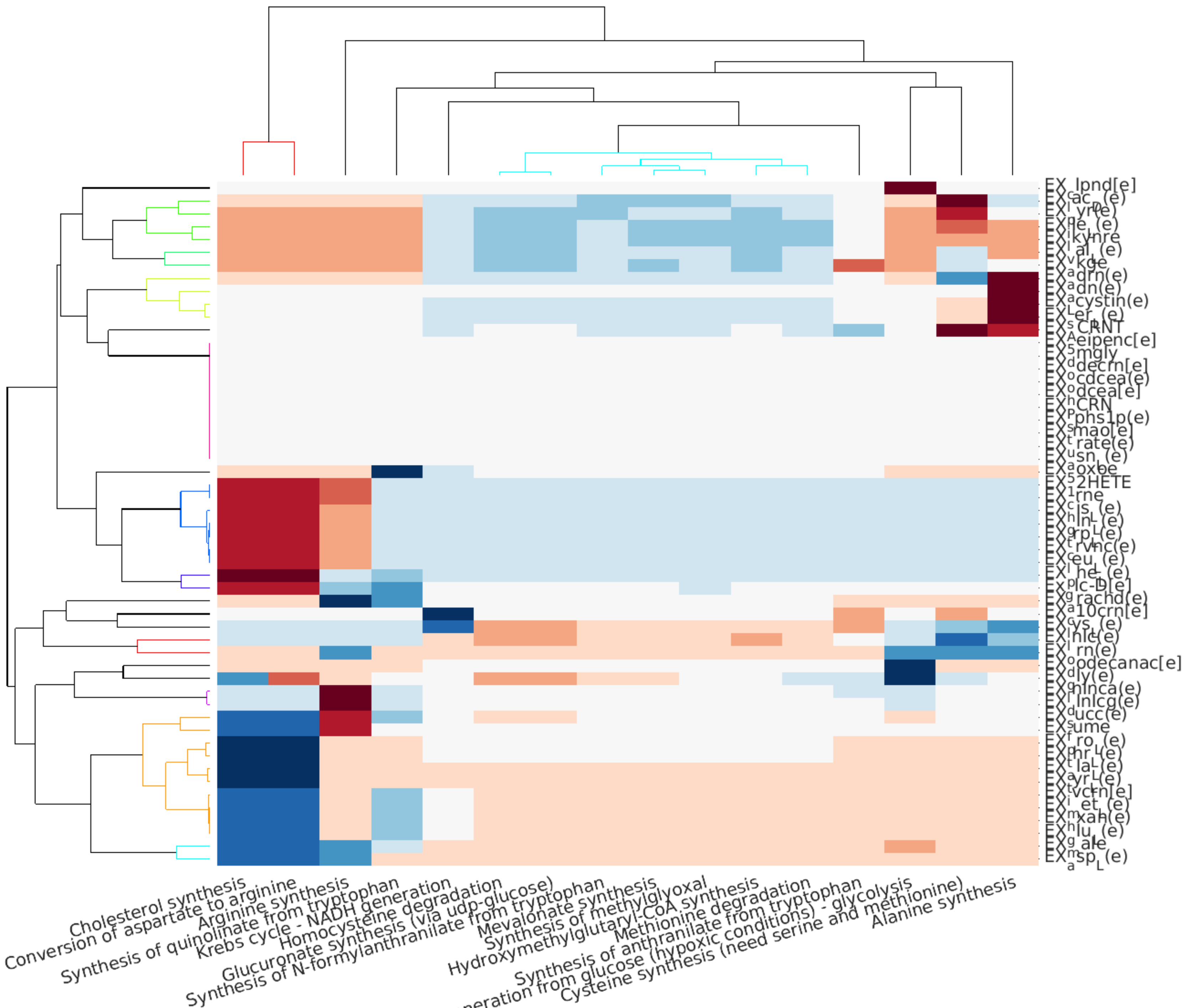

Cholesterol synthesis
Conversion of aspartate to arginine
Arginine synthesis
Synthesis of quinolinate from tryptophan
Krebs cycle - NADH generation
Homocysteine degradation
Glucuronate synthesis (via udp-glucose)
Synthesis of N-formylanthranilate from tryptophan
Mevalonate synthesis
Synthesis of methylglyoxal
Hydroxymethylglutaryl-CoA synthesis
Methionine degradation
Synthesis of anthranilate from tryptophan
glycolysis
Cysteine synthesis (need serine and methionine)
Alanine synthesis

Patient 78

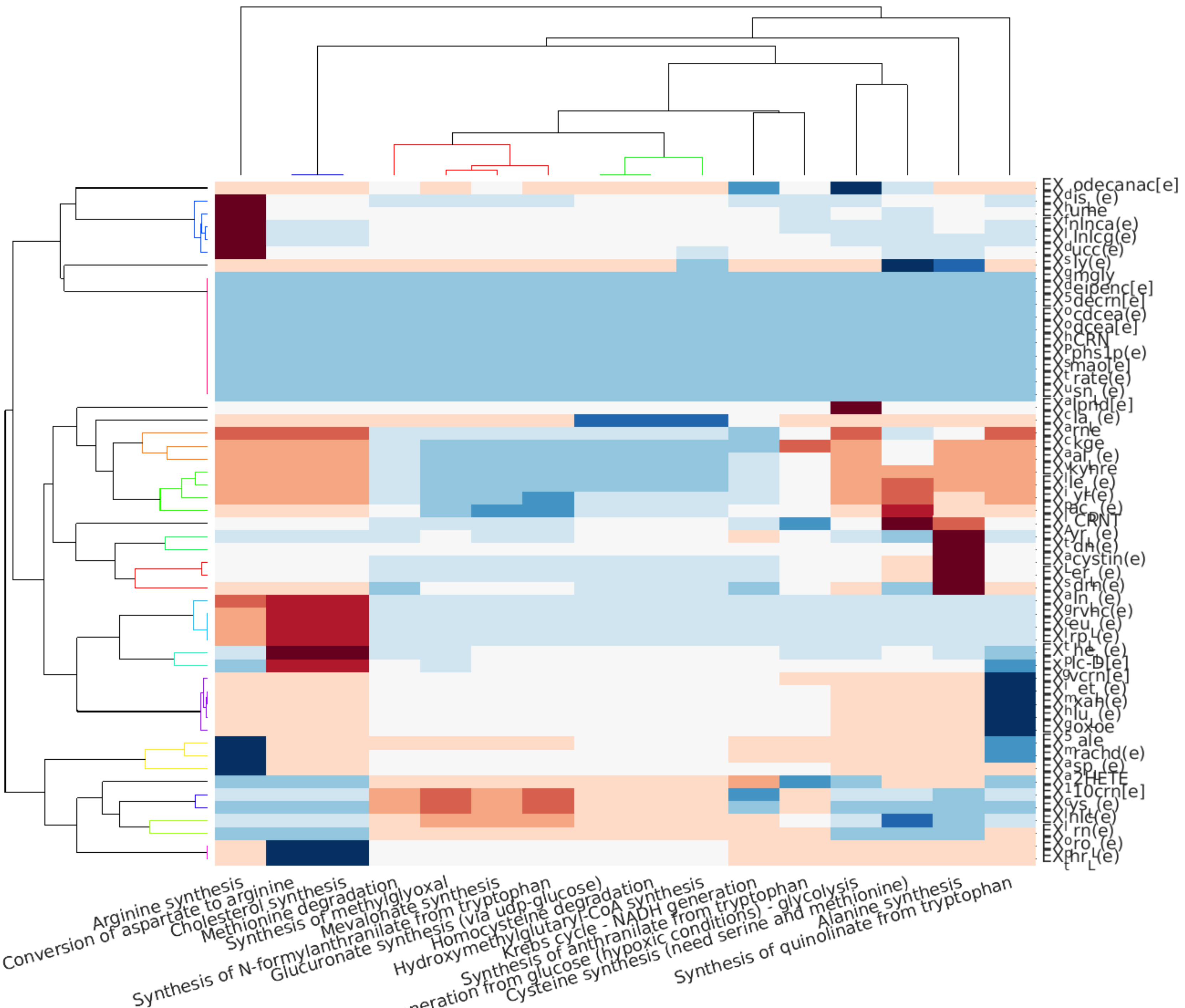

Arginine synthesis
Conversion of aspartate to arginine
Cholesterol synthesis
Methionine degradation
Synthesis of methylglyoxal
Synthesis of N-formylanthranilate from tryptophan
Mevalonate synthesis
Glucuronate synthesis (via udp-glucose)
Homocysteine degradation
Hydroxymethylglutaryl-CoA synthesis
Krebs cycle - NADH generation
Synthesis of anthranilate from tryptophan
Cysteine synthesis (need serine and methionine)
Alanine synthesis
Synthesis of quinolinate from tryptophan

Patient 79

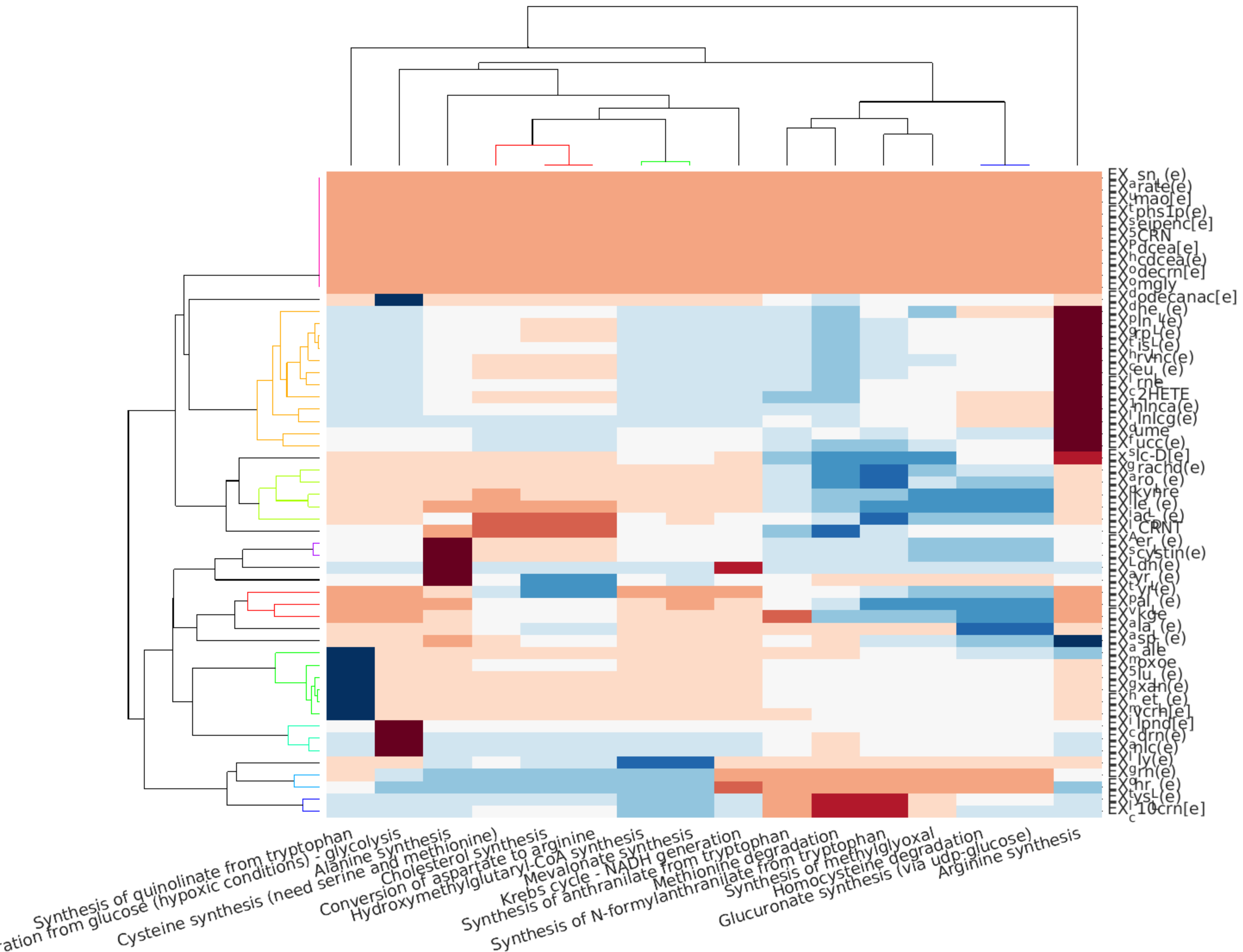
Synthesis of quinolinate from tryptophan
Alanine synthesis
Cysteine synthesis (need serine and methionine)
Cholesterol synthesis
Conversion of aspartate to arginine
Hydroxymethylglutaryl-CoA synthesis
Mevalonate synthesis
Krebs cycle - NADH generation
Synthesis of anthranilate from tryptophan
Methionine degradation
Synthesis of N-formylanthranilate from tryptophan
Synthesis of methylglyoxal
Homocysteine degradation
Glucuronate synthesis (via udp-glucose)
Arginine synthesis

Patient 80

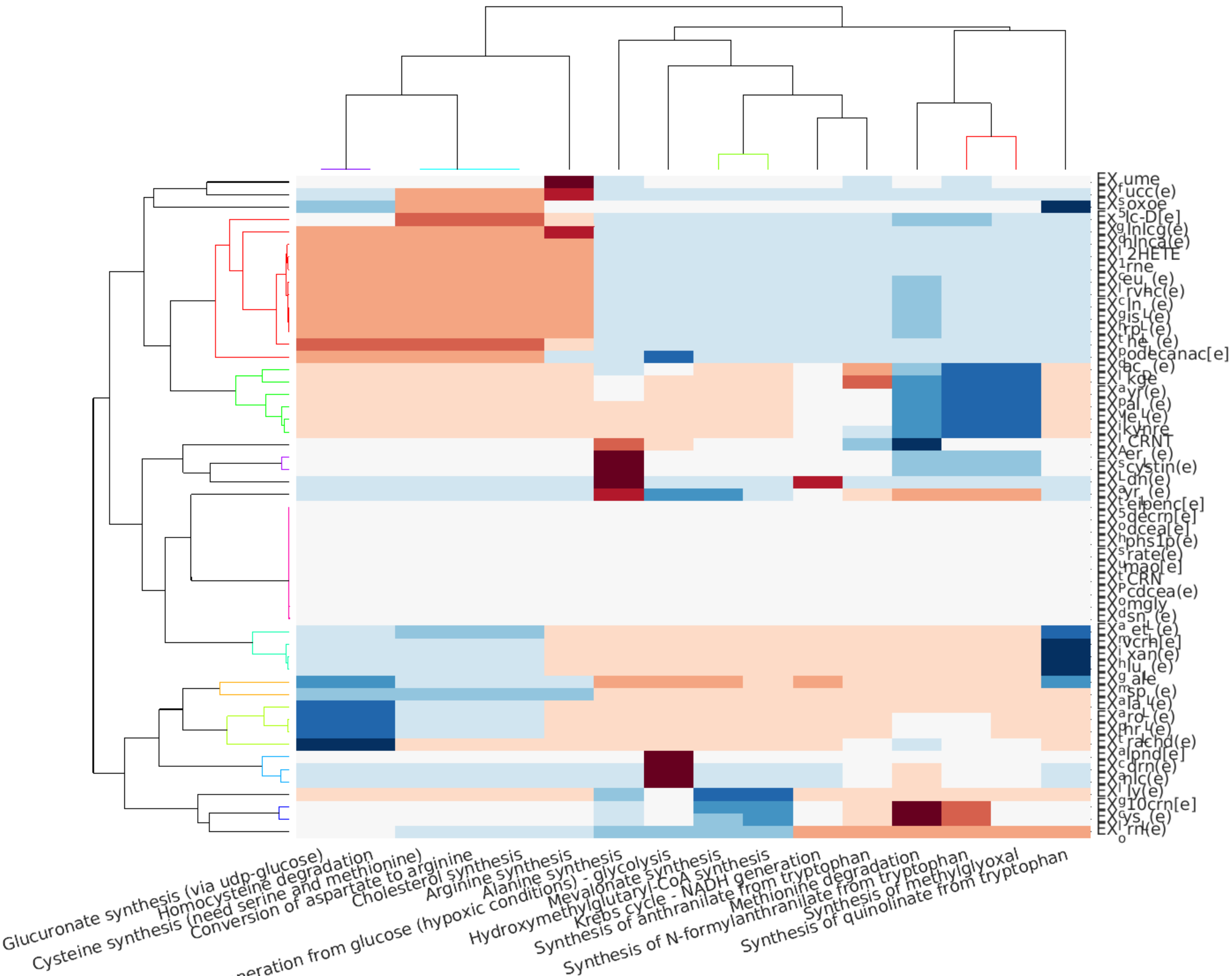
Glucuronate synthesis (via udp-glucose)
Homocysteine degradation
Cysteine synthesis (need serine and methionine)
Conversion of aspartate to arginine
Cholesterol synthesis
Arginine synthesis
Alanine synthesis
eneration from glucose (hypoxic conditions) - glycolysis
Mevalonate synthesis
Hydroxymethylglutaryl-CoA synthesis
Krebs cycle - NADH generation
Synthesis of anthranilate from tryptophan
Methionine degradation
Synthesis of N-formylanthranilate from tryptophan
Synthesis of methylglyoxal
Synthesis of quinolinate from tryptophan

Patient 81

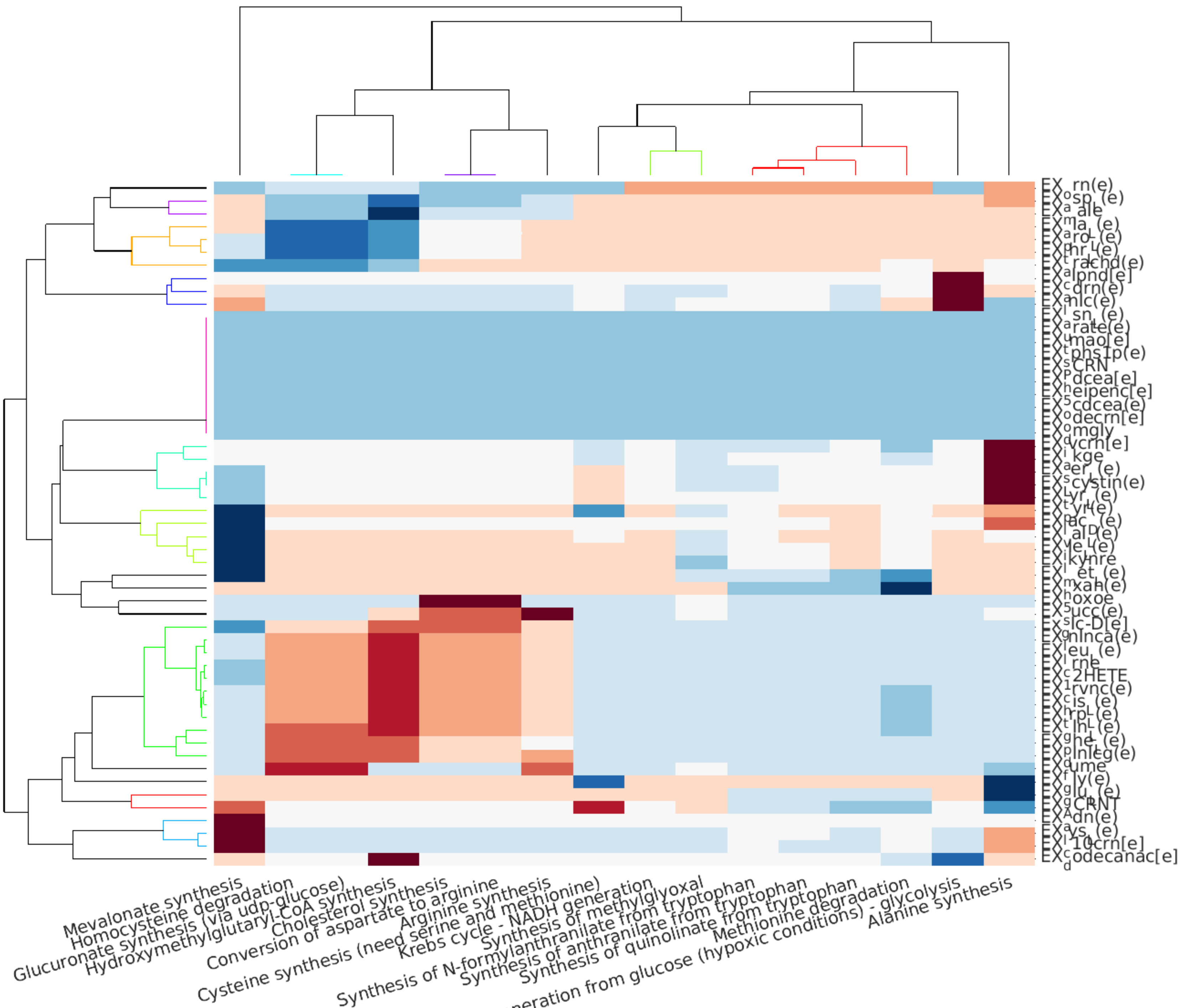

Mevalonate synthesis
Homocysteine degradation
Glucuronate synthesis (via udp-glucose)
Hydroxymethylglutaryl-CoA synthesis
Cholesterol synthesis
Conversion of aspartate to arginine
Arginine synthesis
Cysteine synthesis (need serine and methionine)
Krebs cycle - NADH generation
Synthesis of methylglyoxal
Synthesis of N-formylanthranilate from tryptophan
Synthesis of anthranilate from tryptophan
Synthesis of quinolinate from tryptophan
Methionine degradation
Alanine synthesis

Patient 82

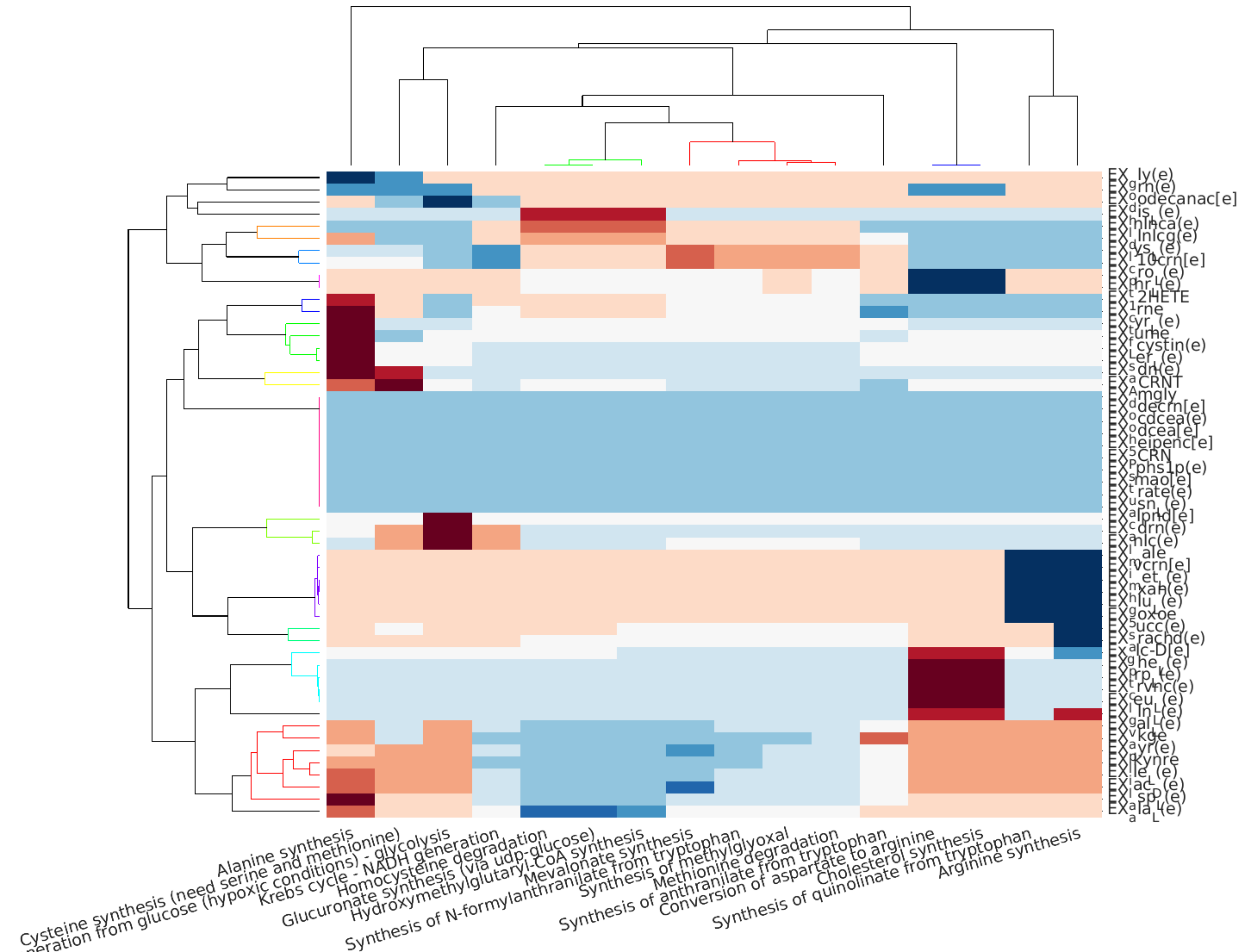

Alanine synthesis
Cysteine synthesis (need serine and methionine)
...eration from glucose (hypoxic conditions) - glycolysis
Krebs cycle - NADH generation
Homocysteine degradation
Glucuronate synthesis (via udp-glucose)
Hydroxymethylglutaryl-CoA synthesis
Mevalonate synthesis
Synthesis of N-formylanthranilate from tryptophan
Synthesis of methylglyoxal
Methionine degradation
Synthesis of anthranilate from tryptophan
Conversion of aspartate to arginine
Cholesterol synthesis
Synthesis of quinolinate from tryptophan
Arginine synthesis

Patient 83

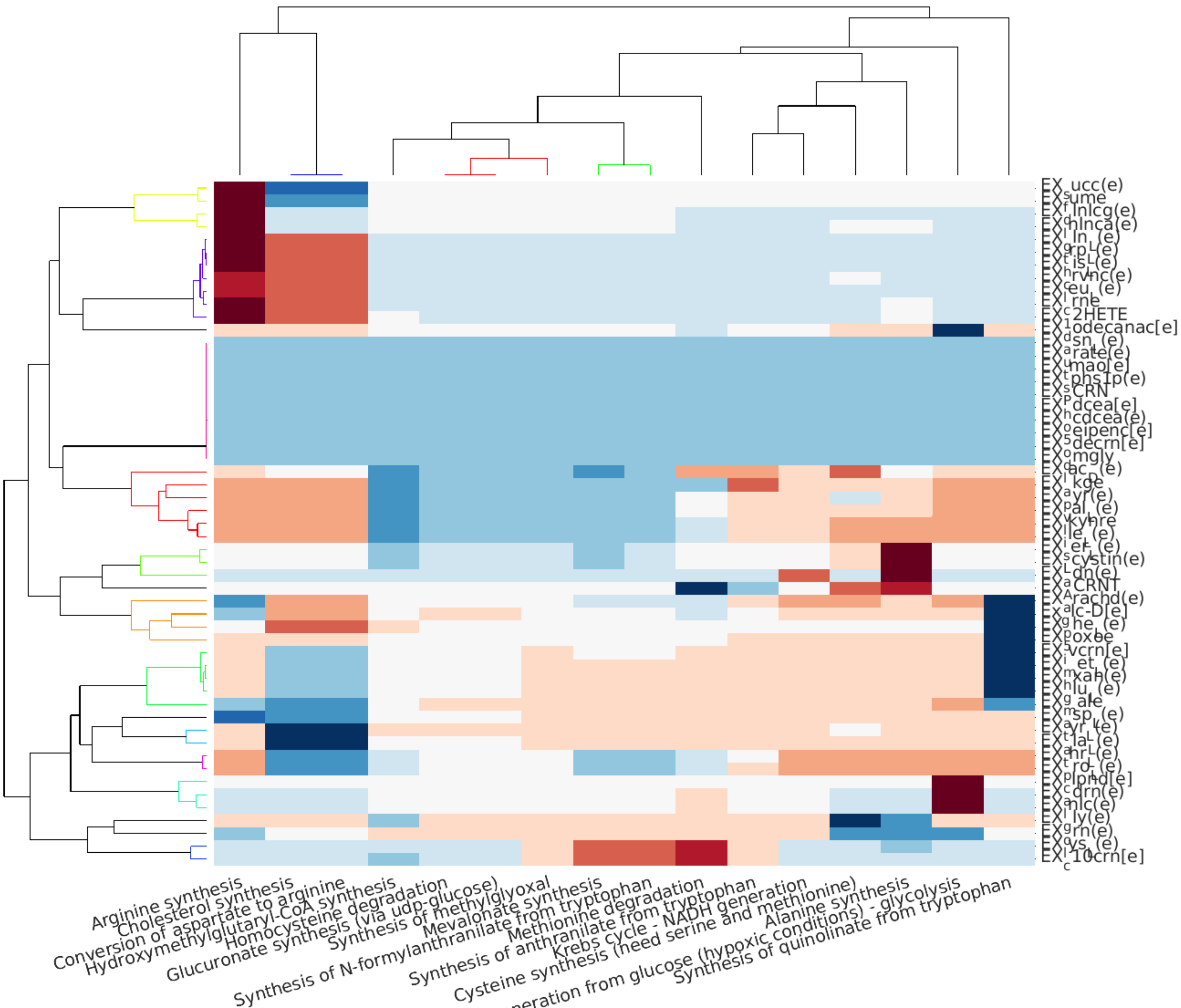
Arginine synthesis
Cholesterol synthesis
Conversion of aspartate to arginine
Hydroxymethylglutaryl-CoA synthesis
Homocysteine degradation
Glucuronate synthesis (via udp-glucose)
Synthesis of methylglyoxal
Mevalonate synthesis
Synthesis of N-formylanthranilate from tryptophan
Methionine degradation
Synthesis of anthranilate from tryptophan
Krebs cycle - NADH generation
Cysteine synthesis (need serine and methionine)
Alanine synthesis
(hypoxic conditions) - glycolysis
Synthesis of quinolinate from tryptophan

Patient 84

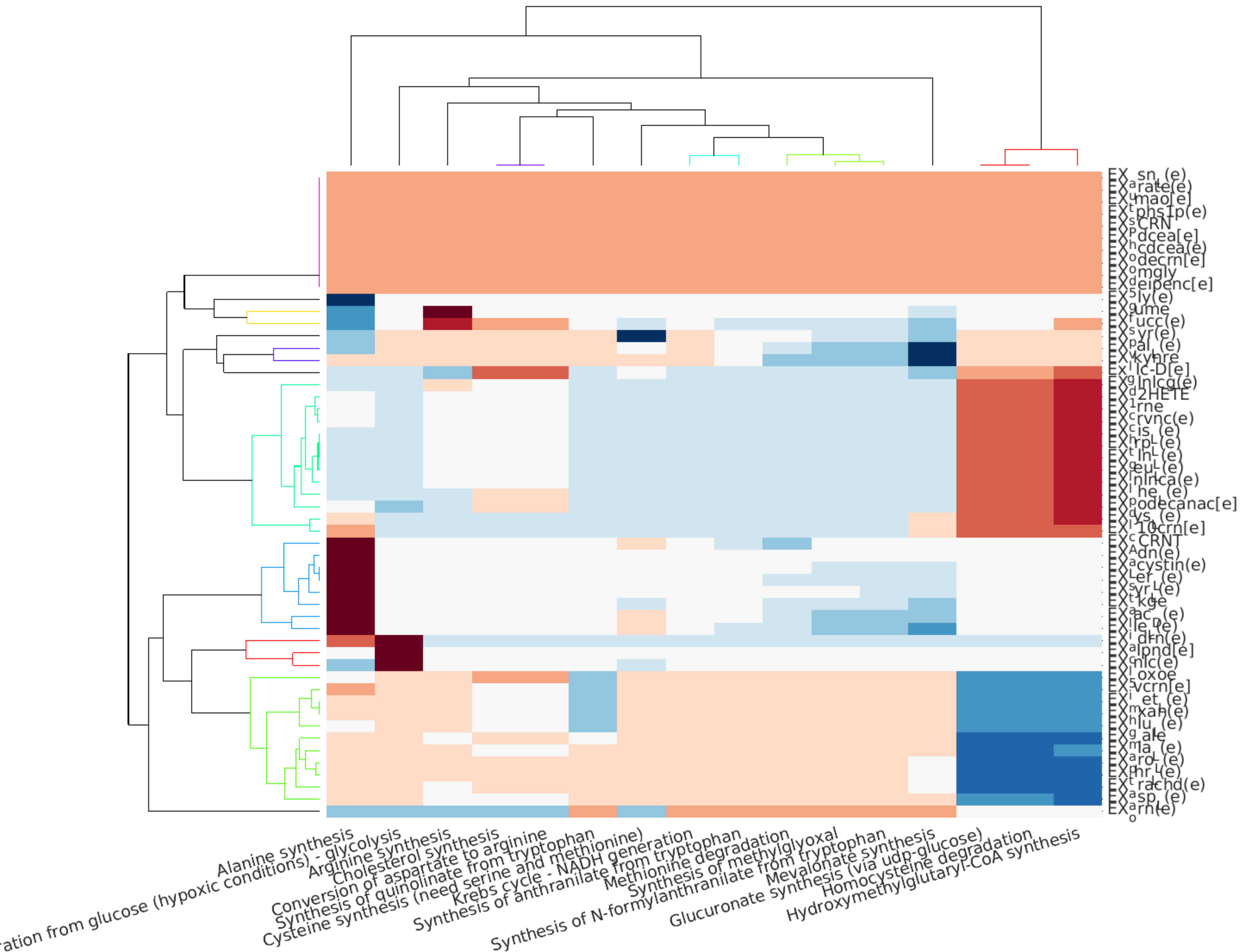
Alanine synthesis
ation from glucose (hypoxic conditions) - glycolysis
Arginine synthesis
Cholesterol synthesis
Conversion of aspartate to arginine
Synthesis of quinolinate from tryptophan
Cysteine synthesis (need serine and methionine)
Krebs cycle - NADH generation
Synthesis of anthranilate from tryptophan
Synthesis of N-formylanthranilate from tryptophan
Methionine degradation
Synthesis of methylglyoxal
Glucuronate synthesis (via udp-glucose)
Mevalonate synthesis
Homocysteine degradation
Hydroxymethylglutaryl-CoA synthesis

Patient 85

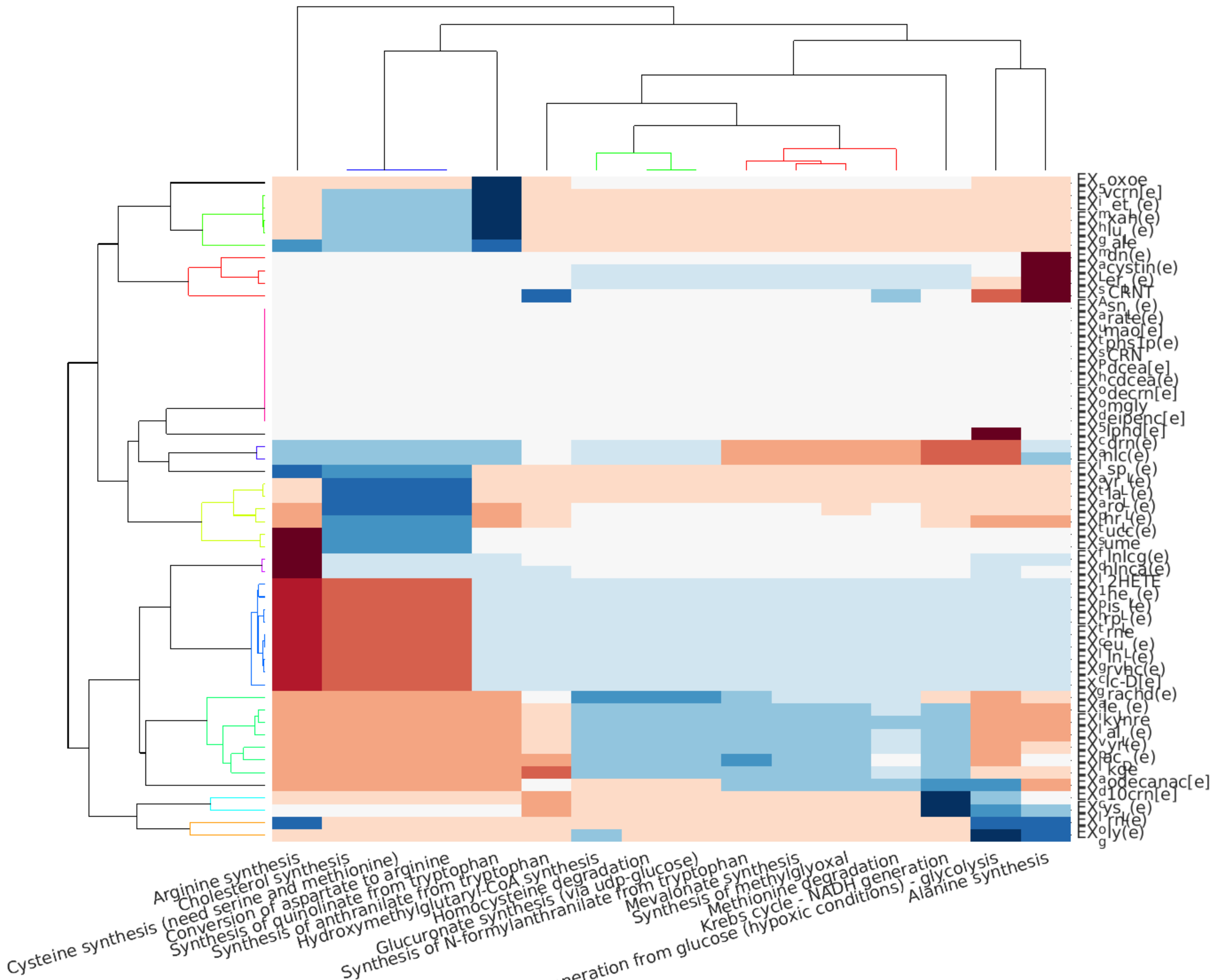
Arginine synthesis
Cholesterol synthesis
Cysteine synthesis (need serine and methionine)
Conversion of aspartate to arginine
Synthesis of quinolinate from tryptophan
Synthesis of anthranilate from tryptophan
Hydroxymethylglutaryl-CoA synthesis
Homocysteine degradation
Glucuronate synthesis (via udp-glucose)
Synthesis of N-formylanthranilate from tryptophan
Mevalonate synthesis
Synthesis of methylglyoxal
Methionine degradation
Krebs cycle - NADH generation
glycolysis
Alanine synthesis

Patient 86

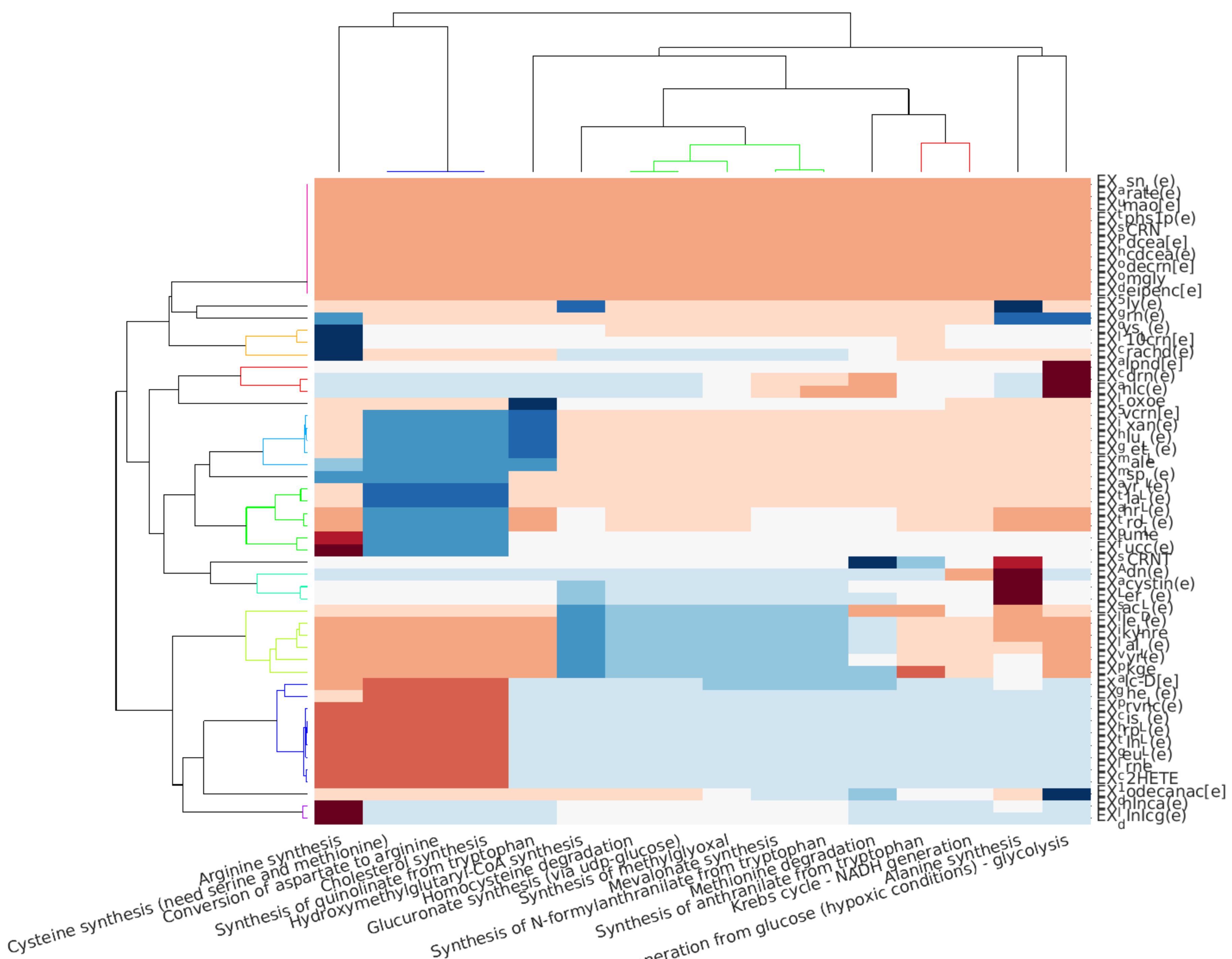
Arginine synthesis
Cysteine synthesis (need serine and methionine)
Conversion of aspartate to arginine
Cholesterol synthesis
Synthesis of quinolinate from tryptophan
Hydroxymethylglutaryl-CoA synthesis
Homocysteine degradation
Glucuronate synthesis (via udp-glucose)
Synthesis of methylglyoxal
Synthesis of N-formylanthranilate from tryptophan
Mevalonate synthesis
Methionine degradation
Synthesis of anthranilate from tryptophan
Krebs cycle - NADH generation
Alanine synthesis
from glucose (hypoxic conditions) - glycolysis

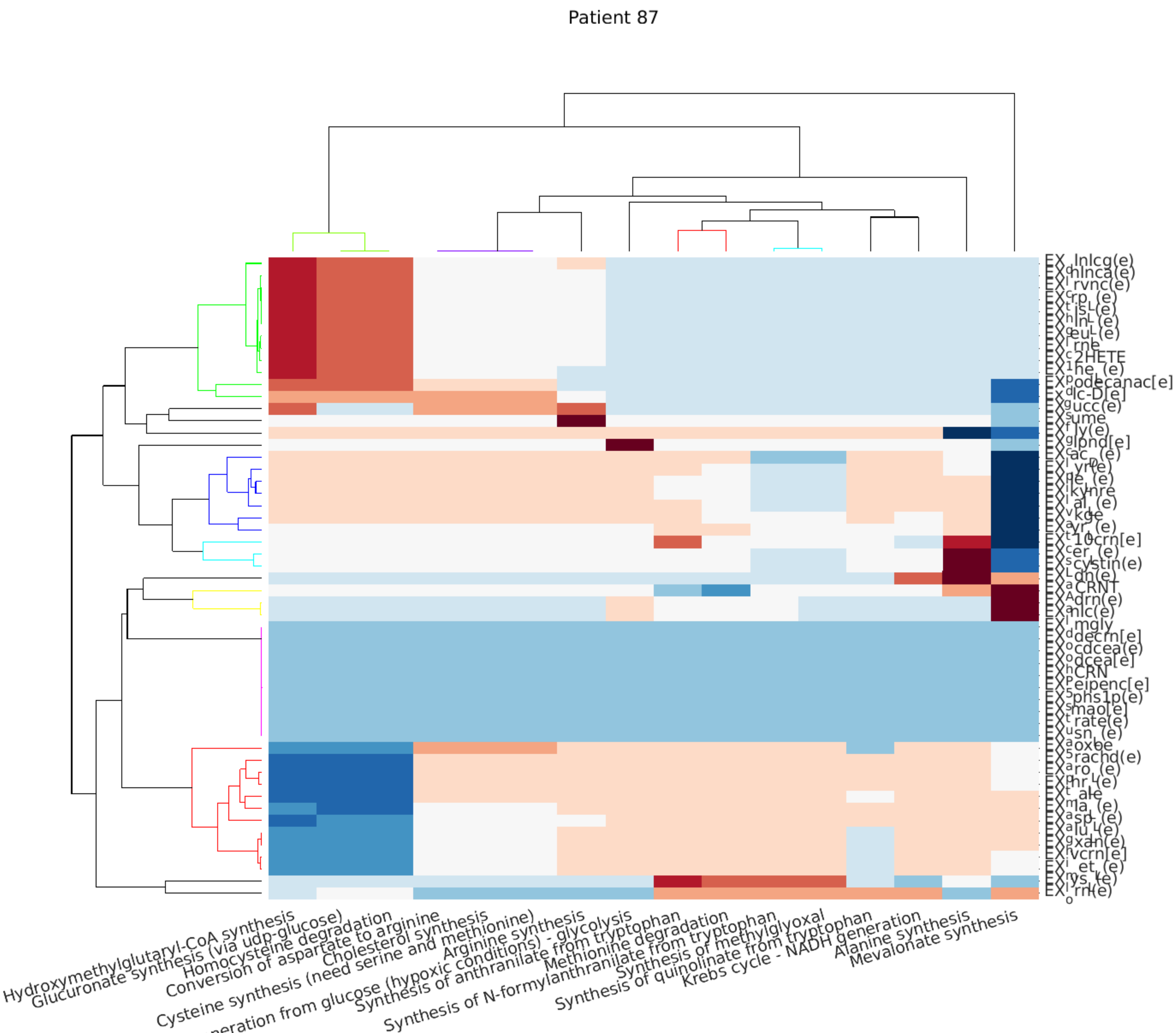

Patient 87
Hydroxymethylglutaryl-CoA synthesis
Glucuronate synthesis (via udp-glucose)
Homocysteine degradation
Conversion of aspartate to arginine
Cholesterol synthesis
Cysteine synthesis (need serine and methionine)
Arginine synthesis
from glucose (hypoxic conditions) - glycolysis
Synthesis of anthranilate from tryptophan
Methionine degradation
Synthesis of N-formylanthranilate from tryptophan
Synthesis of methylglyoxal
Synthesis of quinolinate from tryptophan
Krebs cycle - NADH generation
Alanine synthesis
Mevalonate synthesis

Patient 88

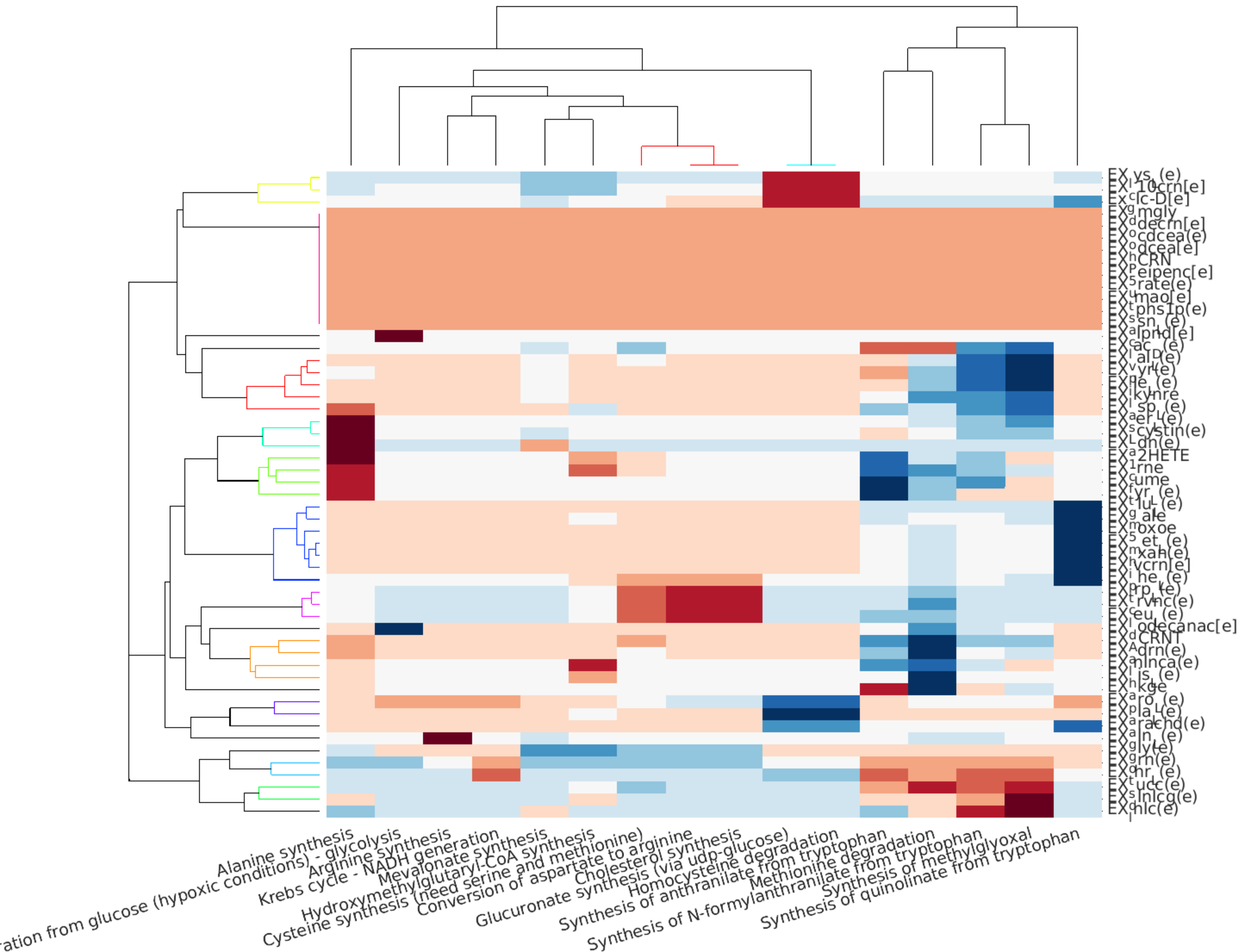

Alanine synthesis
ration from glucose (hypoxic conditions) - glycolysis
Arginine synthesis
Krebs cycle - NADH generation
Mevalonate synthesis
Hydroxymethylglutaryl-CoA synthesis
Cysteine synthesis (need serine and methionine)
Conversion of aspartate to arginine
Cholesterol synthesis
Glucuronate synthesis (via udp-glucose)
Homocysteine degradation
Synthesis of anthranilate from tryptophan
Methionine degradation
Synthesis of N-formylanthranilate from tryptophan
Synthesis of methylglyoxal
Synthesis of quinolinate from tryptophan

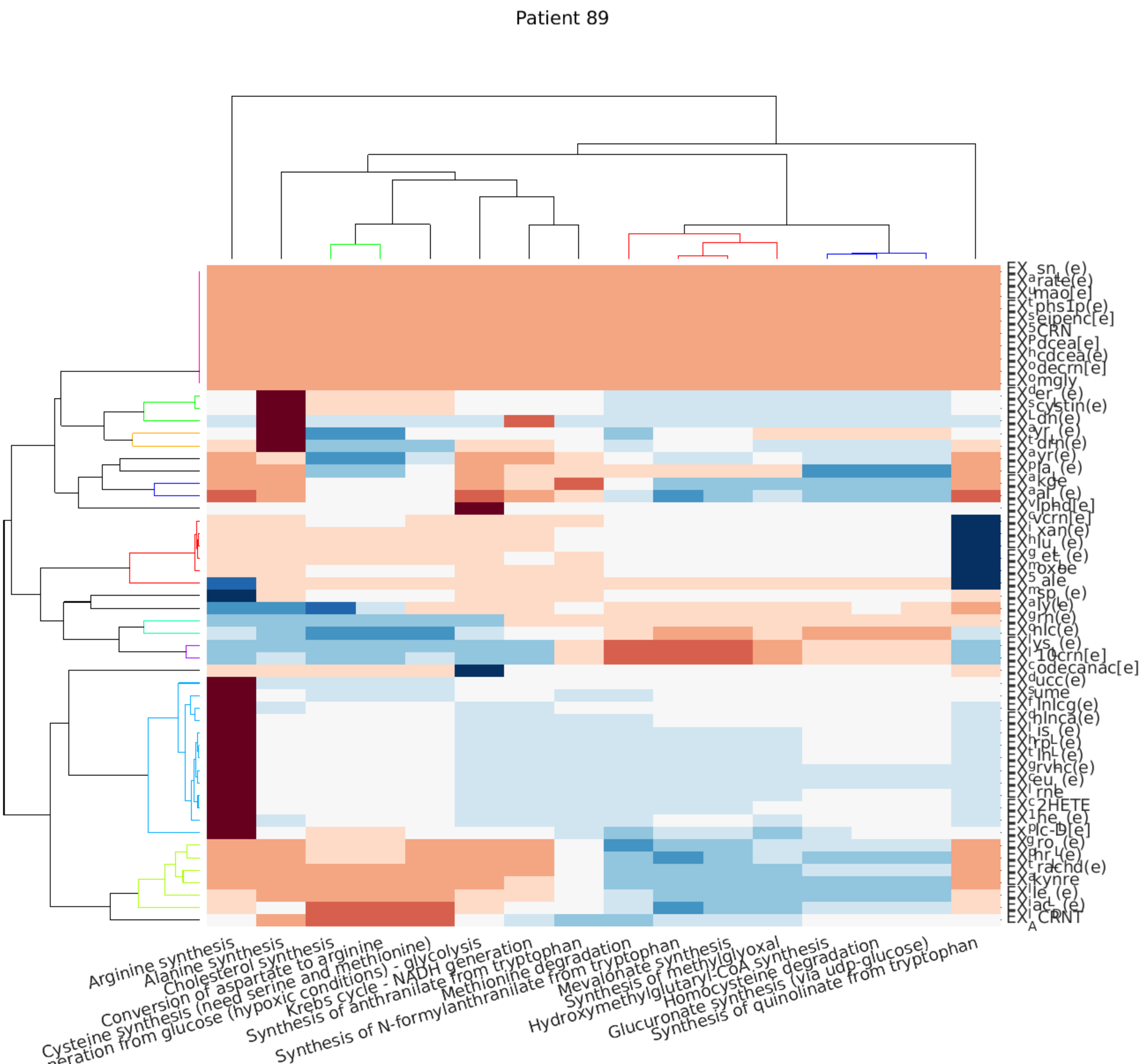
Patient 89
Arginine synthesis
Alanine synthesis
Cholesterol synthesis
Conversion of aspartate to arginine
Cysteine synthesis (need serine and methionine)
Krebs cycle - NADH generation
Synthesis of anthranilate from tryptophan
Methionine degradation
Synthesis of N-formylanthranilate from tryptophan
Mevalonate synthesis
Synthesis of methylglyoxal
Hydroxymethylglutaryl-CoA synthesis
Homocysteine degradation
Glucuronate synthesis (via udp-glucose)
Synthesis of quinolinate from tryptophan

Patient 90

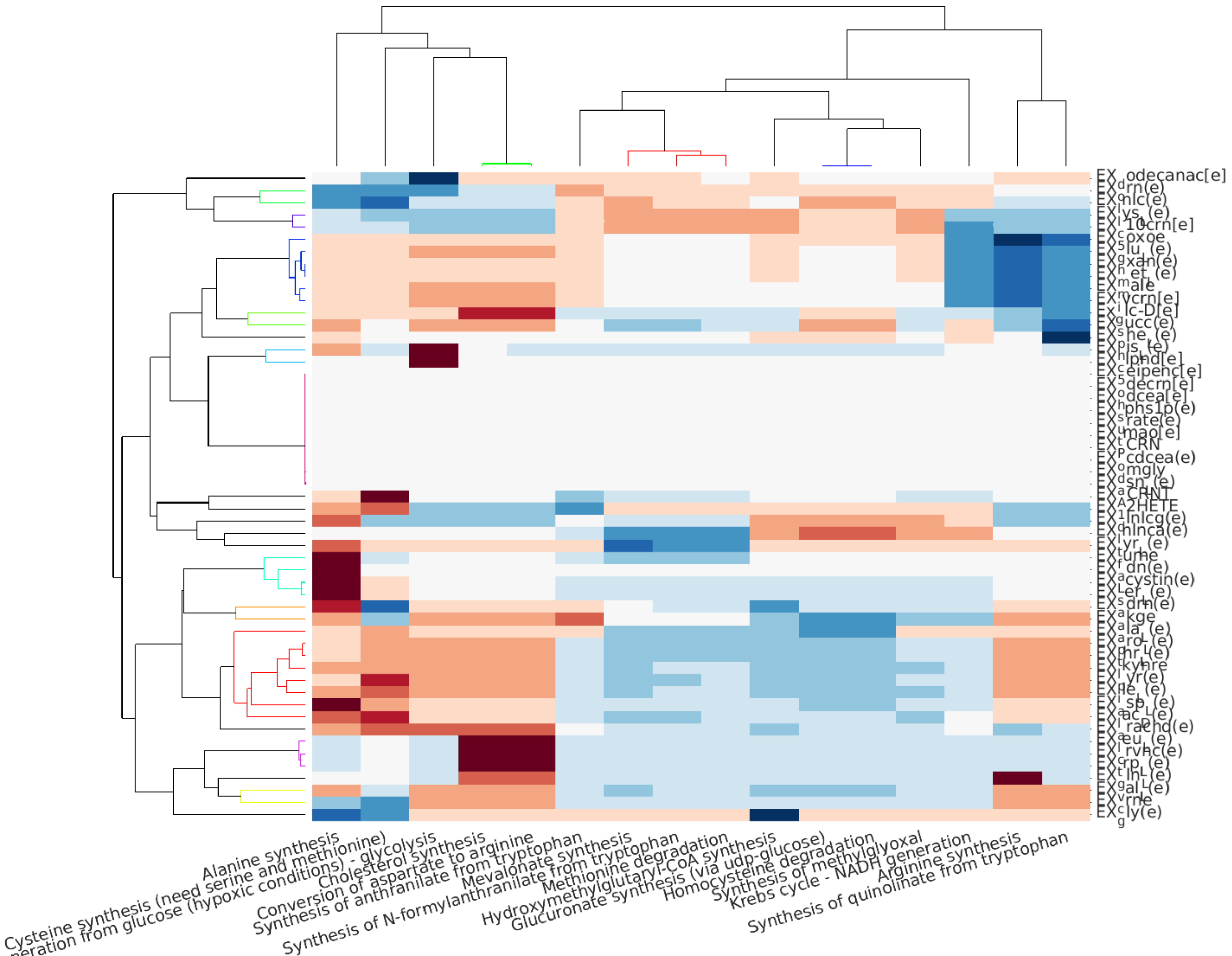

Alanine synthesis
Cysteine synthesis (need serine and methionine)
Cholesterol synthesis
Conversion of aspartate to arginine
Synthesis of anthranilate from tryptophan
Mevalonate synthesis
Synthesis of N-formylanthranilate from tryptophan
Methionine degradation
Hydroxymethylglutaryl-CoA synthesis
Glucuronate synthesis (via udp-glucose)
Homocysteine degradation
Synthesis of methylglyoxal
Krebs cycle - NADH generation
Arginine synthesis
Synthesis of quinolinate from tryptophan

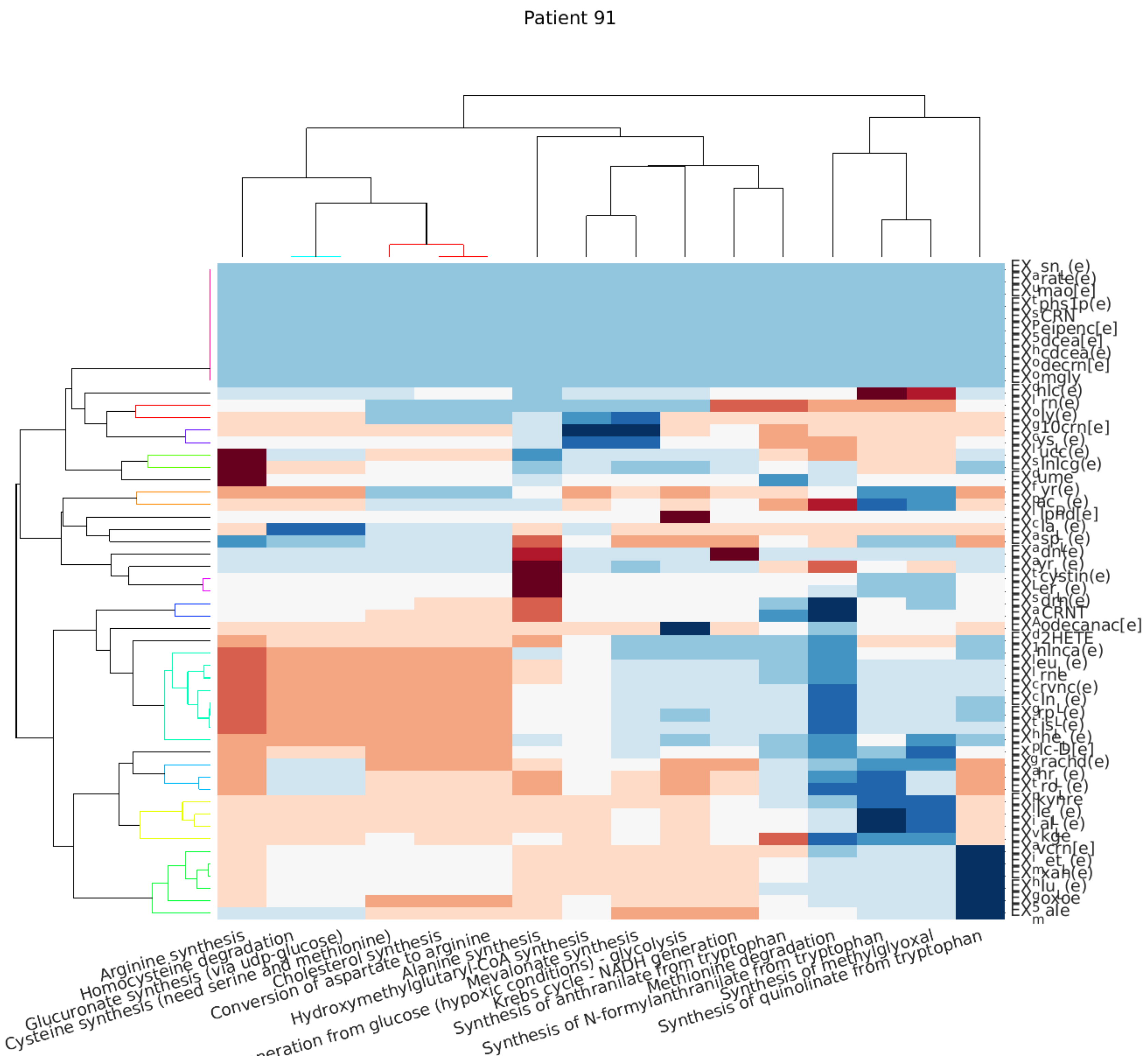
Patient 91
Arginine synthesis
Homocysteine degradation
Glucuronate synthesis (via udp-glucose)
Cysteine synthesis (need serine and methionine)
Cholesterol synthesis
Conversion of aspartate to arginine
Alanine synthesis
Hydroxymethylglutaryl-CoA synthesis
Mevalonate synthesis
Krebs cycle - NADH generation
Synthesis of anthranilate from tryptophan
Methionine degradation
Synthesis of N-formylanthranilate from tryptophan
Synthesis of methylglyoxal
Synthesis of quinolinate from tryptophan

Patient 92

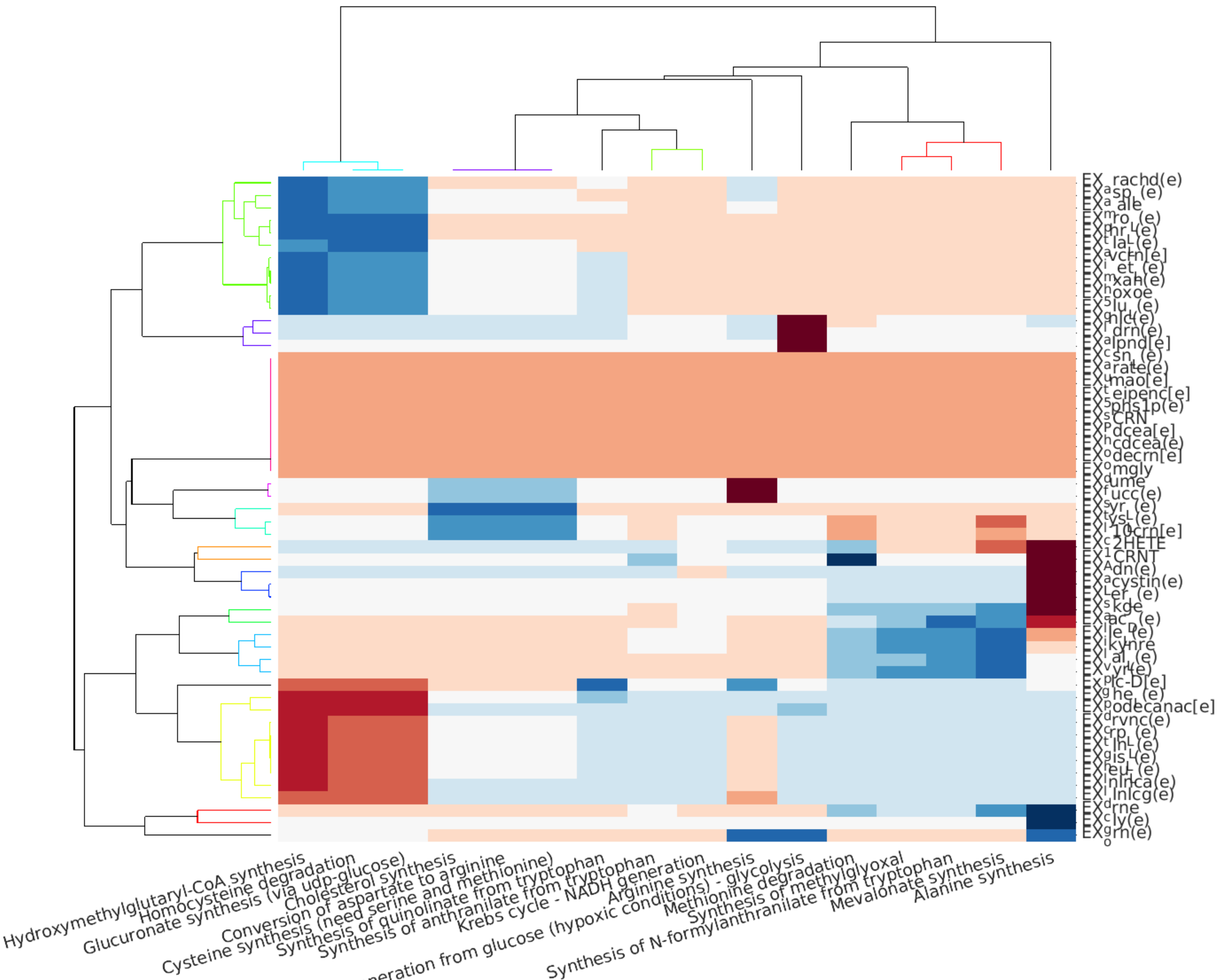
Hydroxymethylglutaryl-CoA synthesis
Homocysteine degradation
Glucuronate synthesis (via udp-glucose)
Cholesterol synthesis
Conversion of aspartate to arginine
Cysteine synthesis (need serine and methionine)
Synthesis of quinolinate from tryptophan
Synthesis of anthranilate from tryptophan
Krebs cycle - NADH generation
Arginine synthesis
Methionine degradation
Synthesis of methylglyoxal
Synthesis of N-formylanthranilate from tryptophan
Mevalonate synthesis
Alanine synthesis

Patient 93

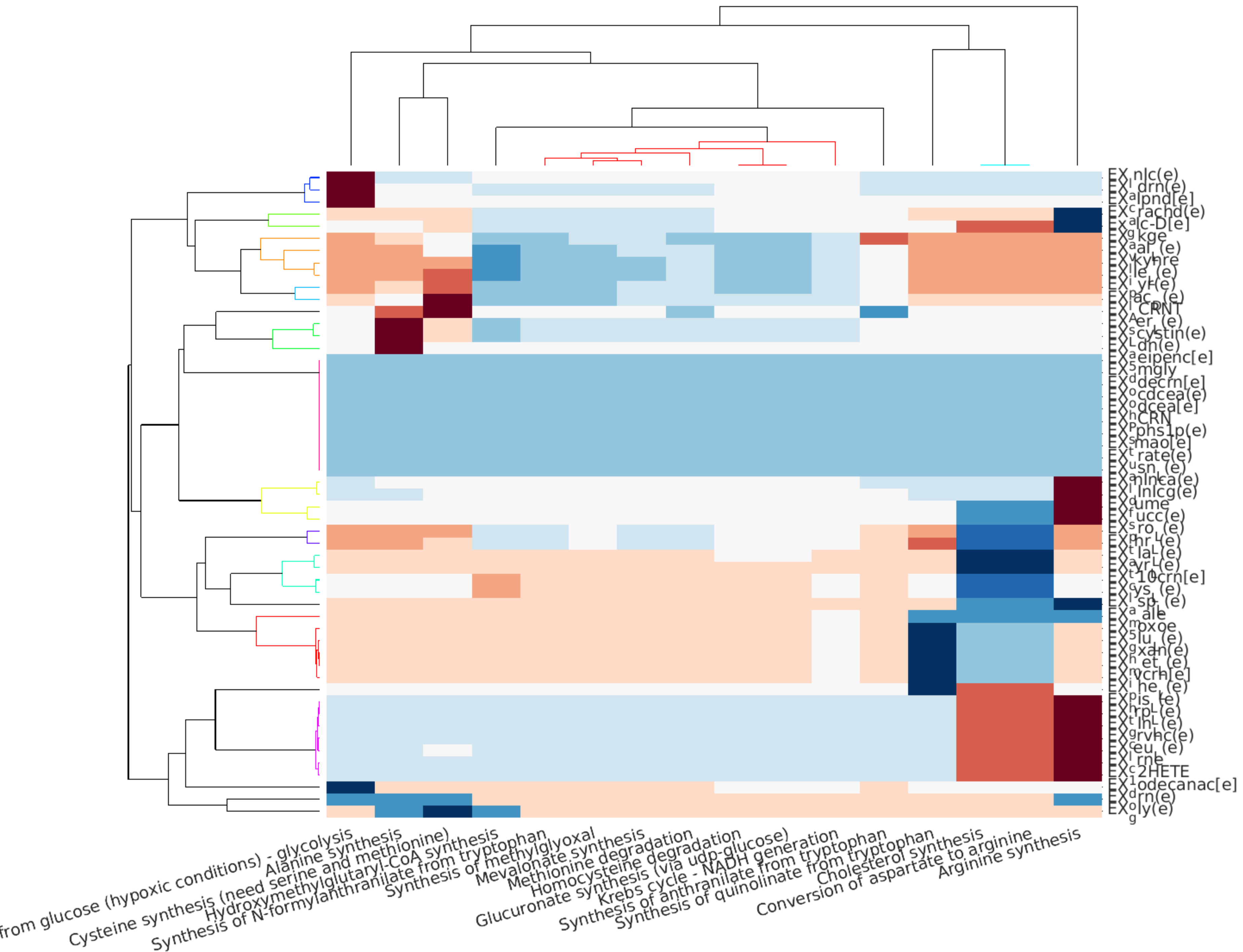
from glucose (hypoxic conditions) - glycolysis
Alanine synthesis
Cysteine synthesis (need serine and methionine)
Hydroxymethylglutaryl-CoA synthesis
Synthesis of N-formylanthranilate from tryptophan
Synthesis of methylglyoxal
Mevalonate synthesis
Methionine degradation
Homocysteine degradation
Glucuronate synthesis (via udp-glucose)
Krebs cycle - NADH generation
Synthesis of anthranilate from tryptophan
Synthesis of quinolinate from tryptophan
Cholesterol synthesis
Conversion of aspartate to arginine
Arginine synthesis

Patient 94

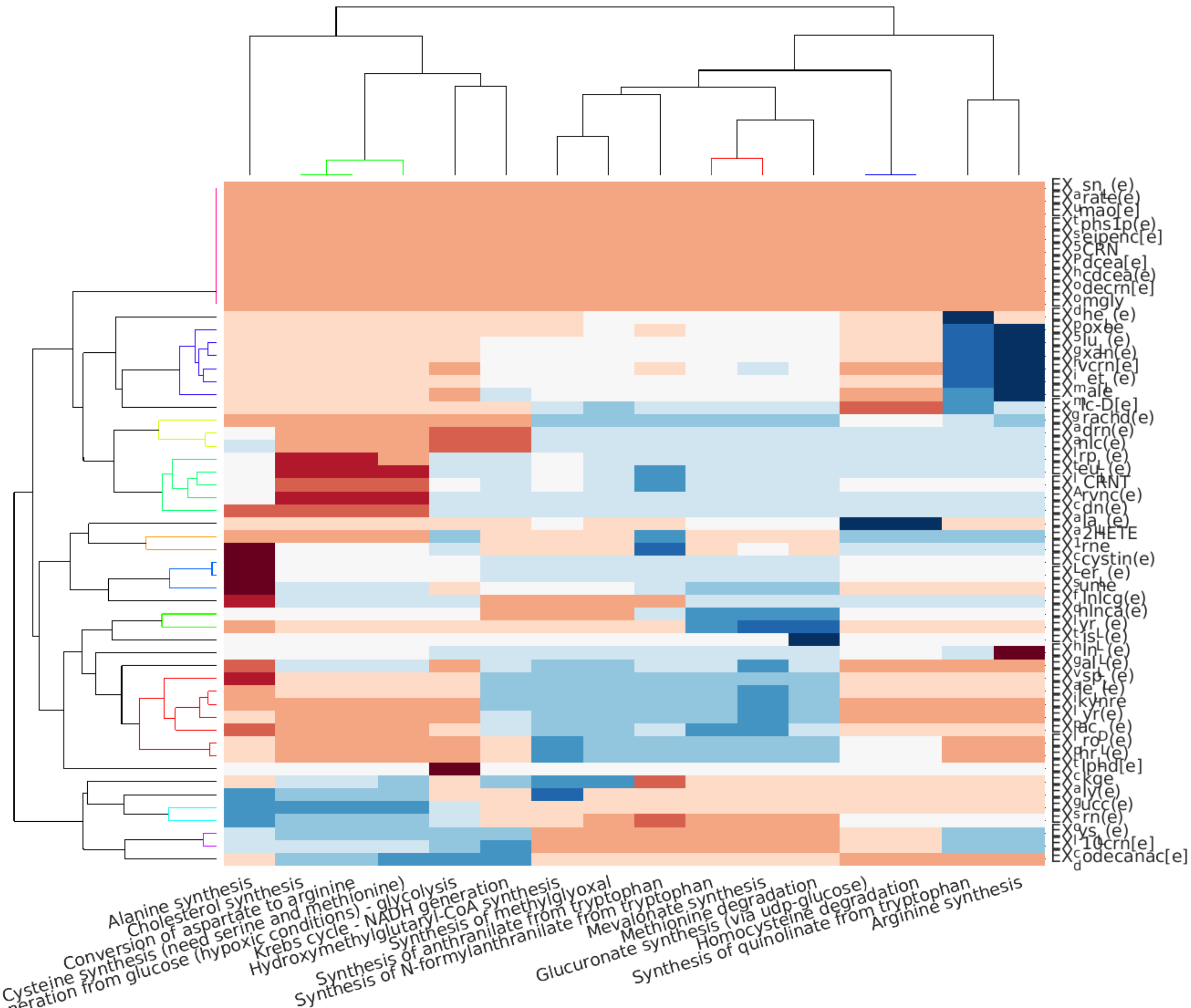
Alanine synthesis
Cholesterol synthesis
Conversion of aspartate to arginine
Cysteine synthesis (need serine and methionine)
eration from glucose (hypoxic conditions) - glycolysis
Krebs cycle - NADH generation
Hydroxymethylglutaryl-CoA synthesis
Synthesis of methylglyoxal
Synthesis of anthranilate from tryptophan
Synthesis of N-formylanthranilate from tryptophan
Mevalonate synthesis
Methionine degradation
Glucuronate synthesis (via udp-glucose)
Homocysteine degradation
Synthesis of quinolinate from tryptophan
Arginine synthesis
EX_CRN
EX_2HETE
EX_CRNT

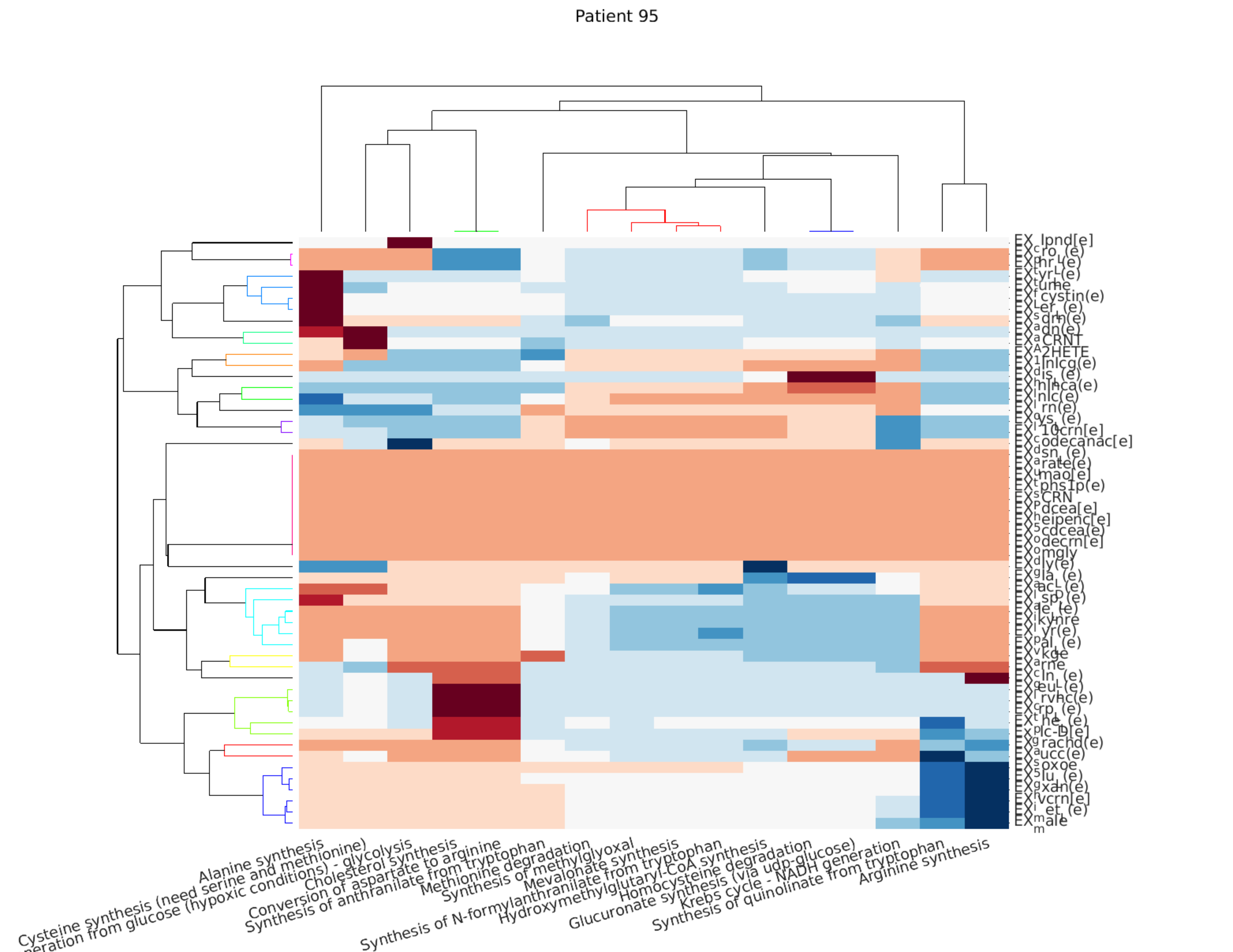
Patient 95
Alanine synthesis
Cysteine synthesis (need serine and methionine)
eration from glucose (hypoxic conditions) - glycolysis
Cholesterol synthesis
Conversion of aspartate to arginine
Synthesis of anthranilate from tryptophan
Methionine degradation
Synthesis of N-formylanthranilate from tryptophan
Synthesis of methylglyoxal
Mevalonate synthesis
Hydroxymethylglutaryl-CoA synthesis
Homocysteine degradation
Glucuronate synthesis (via udp-glucose)
Krebs cycle - NADH generation
Synthesis of quinolinate from tryptophan
Arginine synthesis
